# Carbocationoids, a concept for controlling highly reactive cationic species

**DOI:** 10.1038/s42004-024-01139-w

**Published:** 2024-03-13

**Authors:** Hikaru Fujita, Daichi Shimada, Jotaro Kudo, Kazuyuki Kosha, Satoshi Kakuyama, Hiromitsu Terasaki, Munetaka Kunishima

**Affiliations:** 1 Faculty of Pharmaceutical Sciences, Institute of Medical, Pharmaceutical, and Health Sciences, Kanazawa University, Kakuma-machi, Kanazawa, 920-1192 Japan; 2 Faculty of Pharmaceutical Sciences, Kobe Gakuin University, 1-1-3 Minatojima, Chuo-ku, Kobe, 650-8586 Japan

**Keywords:** Synthetic chemistry methodology, Synthetic chemistry methodology, Reaction mechanisms

## Abstract

Carbocations, which are positively charged highly electrophilic intermediates, are efficacious for the direct alkylation of low-reactive nucleophiles. The utilization of carbocations in S_N_1 reactions relies on the activation of their precursors in the presence of a nucleophile. However, undesirable interactions between the nucleophile and the leaving group activator limit the scope of acceptable nucleophiles. Here we report a strategy to conduct S_N_1 reactions involving unstable carbocations in an alternative stepwise procedure, which was demonstrated by the benzylation of various neutral nucleophiles. In the first step, carbocations were accumulated in a nucleophile-free solution in the form of carbocationoids utilizing the coordinative stabilization of triazinediones. Subsequently, the addition of these solutions in the second step enabled room-temperature alkylation without the need for acidic additives. This methodology overcomes the inherent challenges of carbocations in S_N_1 reactions.

## Abstract (web summary)

Carbocations are highly electrophilic intermediates involved in S_N_1 reactions, such as the direct alkylation of low-reactivity nucleophiles, however, in situ activation of leaving groups in such reactions remains challenging due to the unstable nature of the carbocations, leading to cross-reactivity between nucleophiles and leaving group activators. Here, the authors develop a two-step procedure to avoid such cross-reactivity: they generate carbocationoids as coordinatively stabilized carbocations in one nucleophile-free solution, and then complete the alkylation in another reaction vessel under mild conditions.

## Introduction

**Fig. 1:**
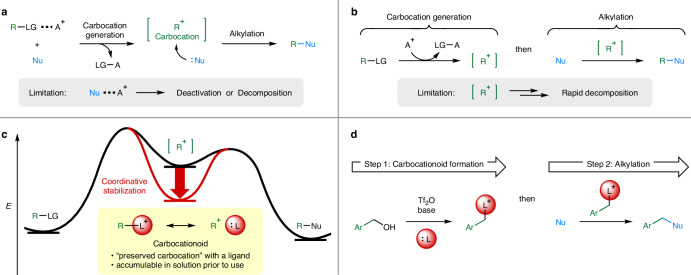
Direct alkylation of low-reactive nucleophiles. **a** Alkylation reaction via an S_N_1 mechanism involving carbocation generation in the presence of a nucleophile. LG leaving group, Nu nucleophile, A^+^ activator. **b** Stepwise S_N_1 reaction process limited by the inherent instability of carbocations. **c** Conceptual energy diagram illustrating the coordinative stabilization of carbocation intermediates with a ligand to form carbocationoids. L ligand. **d** Benzylation of nucleophiles through a two-step procedure of carbocationoid formation followed by alkylation.

Carbocations^1,2^ are positively charged, short-lived intermediates involved in S_N_1 reactions. Their high electrophilicity enables efficient transformation reactions that directly alkylate non-anionic, low-reactive nucleophiles. In typical S_N_1 reactions, a leaving group (LG) present in a suitable precursor (R–LG), such as alcohols (LG = OH)^3–6^ and alkyl halides (LG = X)^7,8^ is activated through interaction with an activator (A^+^) such as Brønsted or Lewis acids (Fig. 1a)^9^. LG activation by one-electron oxidation^10^ or photo-irradiation^11^ has also been reported. The generated carbocation reacts immediately with a co-existing nucleophile to afford the corresponding alkylation product. However, the scope of this reaction methodology is limited owing to competing interactions between the nucleophile and A^+^ that deactivate/decompose one or both. Only compatible combinations of the nucleophile and A^+^ allow the desired alkylation to proceed. In particular, unstable carbocations tend to require a strongly acidic activator with high-temperature heating for their generation, which leads to the decomposition of nucleophiles bearing sensitive functionalities. This limitation can be reasonably resolved if the S_N_1 reactions are conducted stepwise, completing the carbocation generation in another reaction vessel prior to alkylation. However, carbocations generally cannot be accumulated in solution due to their rapid decomposition (Fig. 1b). Whereas exceptionally stable carbocations such as the triphenylmethyl cation^12^ can be isolated, unstable carbocations, such as primary benzyl cations that lack the conjugation from heteroatom lone pairs, require the use of superacidic media for accumulation at low temperature^13,14^, which is not suitable for synthetic applications. Thus, the inherent instability of carbocations is a longstanding problem for the synthetic methodology of the S_N_1 reactions.

Yoshida developed the cation pool method for the accumulation of electrochemically generated carbocations^15,16^ as a methodologically different approach to the S_N_1 reactions involving LG activation. This method has been applied to relatively stable carbocations (e.g., heteroatom-stabilized carbocations and secondary benzyl cations) that can be generated by the selective oxidation of precursors.

Stabilization by complex formation allows for the accumulation of highly reactive intermediates without compromising their characteristic reactivity, as exemplified by carbenoids^17^, used as “preserved carbenes” for synthetic purposes. Applying this idea to the carbocation intermediates in the S_N_1 reactions leads to “preserved carbocations” formed by coordinative stabilization with an external ligand (Fig. 1c). If these accumulable complexes retain carbocation-like alkylating ability, they can likewise be termed carbocationoids. We herein report a distinct synthetic methodology to conduct S_N_1 reactions in an alternative two-step procedure using carbocationoids, which is demonstrated by the benzylation of neutral nucleophiles (Fig. 1d). In step 1, these benzylic carbocationoids are formed from the corresponding benzyl alcohols in a nucleophile-free solution. In step 2, the alkylation of nucleophiles proceeds at ambient temperature, even under mildly basic conditions, because the addition of acidic LG activators is not required.

## Results and Discussion

### Design and preparation

**Fig. 2:**
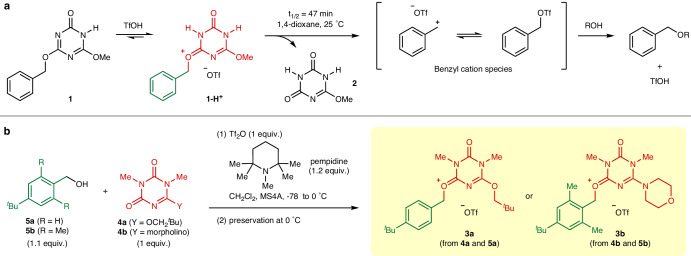
Design and preparation of carbocationoids. **a** Acid-catalyzed *O*-benzylation reaction of reagent **1** involving **1-H**^+^ that generates benzyl cation species. **b** Formation of carbocationoids **3** from ligands **4** and benzyl alcohols **5** by trifluoromethanesulfonic anhydride (Tf_2_O)-mediated dehydrative condensation.

The carbocationoids employed in this study were designed based on our previously developed acid-catalyzed *O*-benzylating reagent **1** (Fig. 2a)^18^. Upon the protonation of reagent **1** with trifluoromethanesulfonic acid (TfOH) in 1,4-dioxane, the resulting **1-H**^**+**^ released benzyl cation species (benzyl trifluoromethanesulfonate)^19^ and triazinedione **2** with a half-life of 47 min at 25 °C. Using **2** as the ligand for the benzyl cation, the structural modification of **1-H**^**+**^ led to the benzylic carbocationoids **3a** and **3b** (Fig. 2b), derived from triazinedione ligands **4a** and **4b**, respectively, with the following features: (1) *N*,*N’*-Dimethyl groups were introduced to the triazinedione skeleton^20^ to prevent deactivation by *N*-deprotonation. (2) Ligand **4a** possesses an *O*-neopentyl group that is resistant to dealkylation in place of the *O*-Me group in **1-H**^**+**^ that is susceptible to demethylation^21^. (3) A 4-*t*-butyl group has been introduced to the benzyl groups of **3a** and **3b** for experimental convenience, as it increases their solubility in organic solvents and reduces the volatility of the alkylation products of nucleophiles. (4) The benzyl group of **3b** bears electron-donating 2,6-dimethyl groups that facilitate C–O bond cleavage at the benzylic position. This electronic effect can be compensated for by the presence of the morpholino group in **4b**, whose strong electron donating ability enhances the coordinating ability of the ligand.

CH_2_Cl_2_ solutions of carbocationoids **3** were prepared by dehydrative condensation of the corresponding ligands **4** (1 equiv.) and benzyl alcohols **5** (1.1 equiv.) using trifluoromethanesulfonic anhydride (Tf_2_O, 1 equiv.) in the presence of a sterically hindered *tert*-amine base, 1,2,2,6,6-pentamethylpiperidine (pempidine, 1.2 equiv.). These materials were combined at −78 °C and then warmed to 0 °C to complete the carbocationoid formation.

### Preservability and alkylating ability

To evaluate the stability and reactivity of these carbocationoids, we conducted the *O*-alkylation of (10-acetoxy)decanol (**6**, 1 equiv.) in the presence of pempidine using **3a** and **3b** (prepared from 2.2 and 2.0 equiv. of **4a** and **4b**, respectively) by changing the preservation time at 0 °C prior to use (Table 1). 1,4-Dioxane was used as a co-solvent, as our previous studies indicated that ethereal solvents have a favorable effect on the product yields of *O*-alkylation reactions involving benzylic carbocation species^22–24^. In entry 1, the *O*-alkylation of **6** using **3a** (preserved for 1 h) proceeded at room temperature to afford ether **7a** in 89% nuclear magnetic resonance (NMR) yield and 88% isolated yield. Significantly, the yield remained at 85% even with an extended preservation time of 20 h (entry 2). By contrast, a control experiment conducted without the use of ligand **4a** did not form **7a** (entry 3). These results indicate the critical role of **4a** and the sufficient stability of **3a** in the absence of suitable nucleophiles. The reaction shown in entry 4 used **3b** (preserved for 1 h) as the electrophile to afford **7b** in 89% yield (or 87% yield using *N*-isobutylmorpholine instead of pempidine). Furthermore, the satisfactory stability of **3b** was confirmed by extending the preservation time to 20 h (89% NMR yield and 87% isolated yield, entry 5). Entry 6 was an attempt to synthesize **7b** without the use of ligand **4b**. In this case, benzyl alcohol **5b** was treated directly with Tf_2_O and pempidine at −78 °C for 1 h before the addition of **6**. As the in situ-formed carbocation species reacted rapidly with **5b** even at the low temperature, symmetric ether **8b** was the major product (95% yield based on **5b**), with only a trace quantity of **7b** detected (<3% based on **6**). Thus, the coordinative stabilization of the ligand **4b** to form **3b** was crucial for the successful alkylation of **6**.

**Table 1:** *O*-Alkylation of alcohol using carbocationoids **3**


Entry	Carbocationoid 3	Preservation time (h)^*^	Product	Yield (%)^†^
1	**3a**	1	**7a**	89 (88)
2	**3a**	20	**7a**	85
3^‡^	–	1	**7a**	not detected
4	**3b**	1	**7b**	89, 87^§^
5	**3b**	20	**7b**	89 (87)
6^||^	–	–	**7b**	<3

^*^Preservation time at 0 °C for carbocationoids **3** prior to use. ^†^Calculated by ^1^H nuclear magnetic resonance (NMR) spectroscopic analysis using an internal standard. Isolated yields are given in parentheses. ^‡^Reaction was conducted without the use of **4a**. ^§^Alkylation was conducted using *N*-isobutylmorpholine instead of pempidine. ^||^After treating **5b** (2 equiv.) with Tf_2_O (2 equiv.) and pempidine (2.2 equiv.) at −78 °C for 1 h, **6** (1 equiv.) and pempidine (2.1 equiv.) were added. The reaction mixture was stirred at room temperature for 3 h.

### Characterization by NMR spectroscopic analysis

The ^1^H and ^13^C{^1^H} NMR spectra (together with the 2D NMR data) of carbocationoids **3** in CDCl_3_ supported their structures (Supplementary Tables 1 and 2). The ^1^H NMR spectral comparisons between carbocationoids **3**, benzyl alcohol **5**, and ligands **4** are shown in Supplementary Fig. 1. The benzylic proton signal of **3b** (5.60 ppm) is downfield shifted than that of benzyl alcohol **5b** (4.72 ppm), while it is largely upfield shifted than that of the corresponding carbocation (8.67 ppm, measured in SbF_5_–SO_2_)^14^, reflecting the coordinatively stabilized carbocation character of **3b**. Upon the addition of nucleophile **6** and pempidine at room temperature, the ^1^H NMR signals of the carbocationoids **3** decreased and disappeared with time (~4 h), accompanied by increase of the corresponding ethers **7** and ligands **4**, respectively (Supplementary Fig. 2). The yields of **3a** and **3b** freshly prepared in CDCl_3_ were 69% and 62% (based on **4a** and **4b**), respectively (Supplementary Fig. 3). Symmetric ethers **8** were also formed in a mole percentage of 15–24% relative to the quantity of ligands **4** used. The majority (~70%) of the initially-formed carbocationoids **3** were still present after 20 h at 0 °C, indicating their preservability at this temperature.

### Reaction generality

**Fig. 3:**
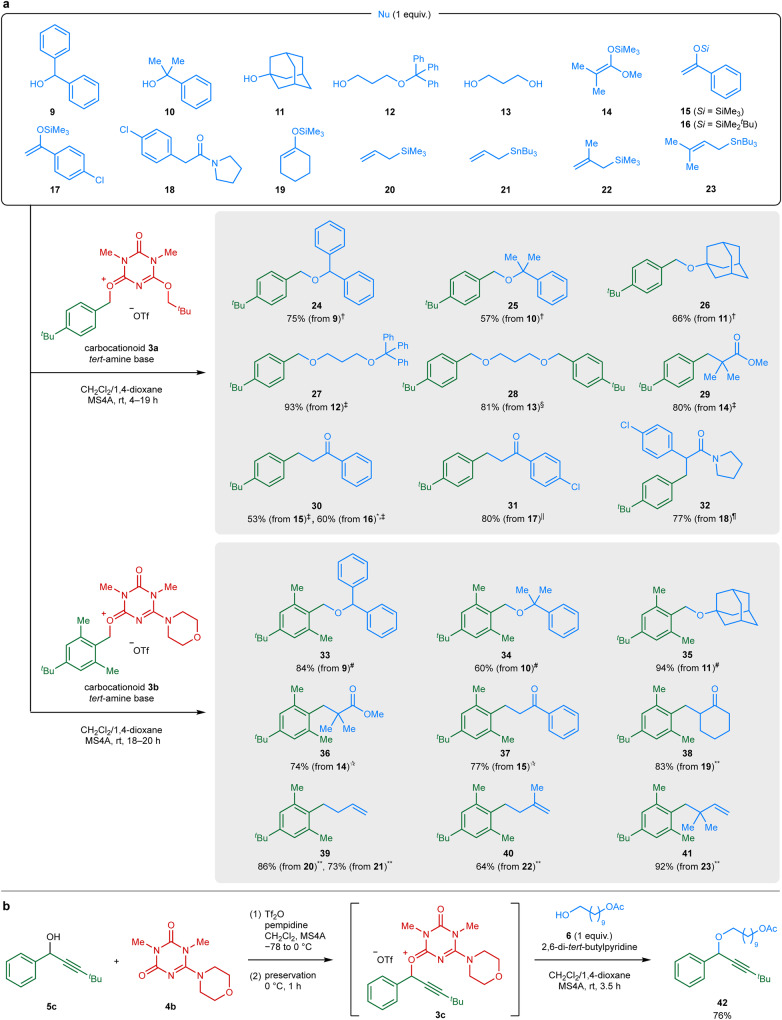
Alkylation reactions using carbocationoids. **a** Reactions using carbocationoids **3a** and **3b**. Isolated yields are presented unless otherwise noted. * indicates a yield calculated by ^1^H NMR spectroscopy using an internal standard. The symbols indicate the reaction conditions: ^†^ **3a** [from **4a** (3.3 equiv.)] and *N*-isobutylmorpholine (3.1 equiv.); ^‡^ **3a** [from **4a** (2.2 equiv.)] and pempidine (2.1 equiv.); ^§^ **3a** [from **4a** (6.6 equiv.)] and *N*-isobutylmorpholine (6.3 equiv.); ^||^ **3a** [from **4a** (3.3 equiv.)] and pempidine (3.1 equiv.); ^¶^ **3a** [from **4a** (4.4 equiv.)] and pempidine (4.2 equiv.); ^#^ **3b** [from **4b** (3.0 equiv.)] and *N*-isobutylmorpholine (3.5 equiv.); ^✩^ **3b** [from **4b** (2.0 equiv.)] and pempidine (2.3 equiv.); ^**^ **3b** [from **4b** (3.0 equiv.)] and *N*-isobutylmorpholine (1.5 equiv.). **b** Secondary alkylation reaction using carbocationoid **3c**.

Carbocationoids **3** were utilized for the alkylation of various nucleophiles (**9**–**23**, Fig. 3a). Benzhydrol (**9**), *tert*-alcohol **10**, and 1-adamantanol (**11**) are suitable electrophiles for S_N_1 reactions because they produce the corresponding carbocations in the presence of acidic activators^25–27^. Nevertheless, these acid-labile alcohols **9**–**11** were successfully converted to the ether products **24**–**26** in 57–75% yield when treated with carbocationoid **3a** under mildly basic conditions. Furthermore, the acid-sensitive triphenylmethyl ether in alcohol **12** remained intact during the alkylation reaction with **3a**, resulting in 93% yield of the ether product **27**. In contrast, such a triphenylmethyl ether functionality was completely decomposed under the acidic reaction conditions reported for the S_N_1-type alkylation using **9** as the electrophile^26,27^ (see Supplementary Fig. 4 for details). Acid-catalyzed S_N_1 reactions using an alcohol (1 equiv.) as the carbocation precursor typically require an excess amount (2 equiv. or more) of a nucleophile to suppress the competing side reaction that forms the symmetric ether derived from the precursor alcohol. Consequently, completing multiple alkylation of polyol nucleophiles using this method is difficult. In contrast, the dialkylation of diol **13** was achieved with **3a**, resulting in 81% yield of the product **28**. The *C*-alkylation of silyl ketene acetal **14** and silyl enol ethers **15**–**17** with **3a** afforded the corresponding carbonyl compounds **29**–**31** in 53–80% yield. The smooth reactions of the neutral nucleophiles **14**–**17** underscore the carbocation-like alkylating ability of **3a**. Notably, amide **18** underwent *C*-alkylation at the α-position of the carbonyl group to afford **32** in 77% yield (reaction mechanism predicted in Supplementary Fig. 5). This reaction process may be initiated by the *O*-alkylation of the weakly nucleophilic carbonyl oxygen atom within the amide, similar to the *O*-alkylation of amides using benzyl cation equivalents that we have previously reported in the study of an amide cleavage reaction^28^. When the alkylation reactions using **3b** were conducted for alcohols **9**–**11**, silyl ketene acetal **14**, and silyl enol ethers **15** and **19**, the corresponding *O*- and *C*-alkylated products **33**–**38** were obtained in 60–94% yield. Furthermore, allylsilane and allylstannane compounds **20**–**23** reacted with **3b** to afford the alkylated products **39**–**41** in 64–92% yield. Again, the efficient alkylation of these neutral nucleophiles highlights the carbocationic reactivity of **3b**.

Our two-step alkylation procedure successfully achieved the secondary *O*-alkylation of nucleophile **6** at room temperature (Fig. 3b). The treatment of nucleophile **6** with a solution of carbocationoid **3c**, which was prepared from secondary alcohol **5c** and ligand **4b**, afforded the ether product **42** in 76% yield.

### Comparison to other alkylation methods

**Fig. 4:**
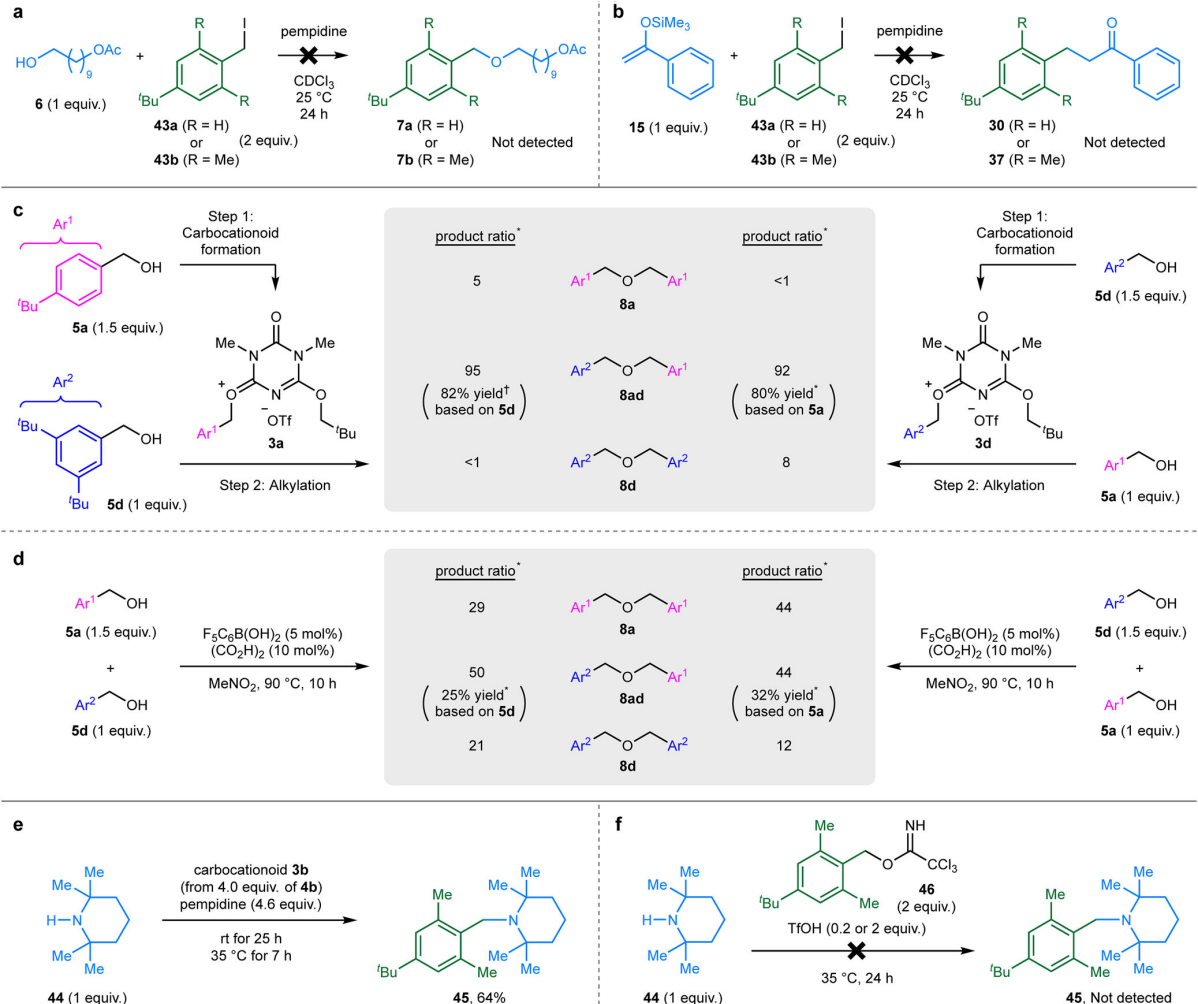
Comparison of alkylation methods. **a** Attempts at the alkylation of alcohol **6** using benzyl iodides **43a** or **43b**. **b** Attempts at the alkylation of silyl enol ether **15** using benzyl iodides **43a** or **43b**. **c** Selective formation of asymmetric ether **8ad** through a stepwise procedure of carbocationoid formation followed by alkylation. **d** Unselective ether formation reactions promoted by an acidic catalyst system. * indicates a ratio or a yield calculated by ^1^H NMR spectroscopy. ^†^ indicates an isolated yield. **e** The *N*-alkylation of sterically hindered secondary amine **44** using carbocationoid **3b**. **f** Attempts at the acid-catalyzed *N*-alkylation of **44** using trichloroacetimidate **46** and trifluoromethanesulfonic acid.

To investigate the reactivity of neutral nucleophiles toward alkyl halides, the reaction of alcohol **6** with benzyl iodide **43a** or **43b** in the presence of pempidine was monitored by ^1^H NMR spectroscopy in CDCl_3_ at 25 °C (Fig. 4a). The observations revealed that no product ether **7a** or **7b** was detected after 24 h (Supplementary Fig. 6), whereas **6** and **43a** or **43b** remained unreacted. Likewise, no formation of **30** or **37** was observed when silyl enol ether **15** was used as the nucleophile (Fig. 4b and Supplementary Fig. 7). Therefore, under the reaction conditions employed, nucleophiles **6** and **12** lacked reactivity with alkyl iodides, which are common reagents for alkylation via an S_N_2 mechanism. These results support the carbocationic reactivity of **3a** and **3b** observed in Fig. 3a.

Next, we conducted the selective preparation of asymmetric ether **8ad** from benzylic alcohols **5a** and **5d** (Fig. 4c) to emphasize the synthetic advantages of our stepwise alkylation methodology utilizing carbocationoids. When **5a** (1.5 equiv.) was used as the precursor of carbocationoid **3a** and **5d** (1 equiv.) as the nucleophile, the product ratio of **5a**-derived ether **8a**, asymmetric ether **8ad**, and **5d**-derived ether **8d** was 5:95: < 1. The isolated yield of **8ad** was 82% based on **5d**. Exchanging the roles of the benzylic alcohols (**5d** used as the precursor of carbocationoid **3d** and **5a** as the nucleophile) produced similar results. The product ratio of **8a**:**8ad**:**8d** was <1:92:8, and the yield of **8ad** was 80% based on **5a**. In contrast, when the ether formation was conducted under S_N_1-type reaction conditions using a boronic acid–oxalic acid catalyst system^29^ (Fig. 4d), the product ratio of **8a**:**8ad**:**8d** was 29:50:21 [with **5a** (1.5 equiv.) and **5d** (1 equiv.); 25% yield of **8ad** based on **5d**] or 44:44:12 [with **5a** (1 equiv.) and **5d** (1.5 equiv.); 32% yield of **8ad** based on **5a**]. These results indicate that the formation of carbocationoids **3a** or **3d** was crucial for the selective formation of **8ad**.

Finally, we carried out the *N*-alkylation of a hindered secondary amine, 2,2,6,6-tetramethylpiperidine (**44**), to demonstrate the effectiveness of our alkylation methodology in contrast to acid-catalyzed alkylation using a trichloroacetimidate reagent. The *N*-alkylation of **44** using **3b** afforded the desired product **45** in 64% yield, despite the significant steric hindrance around the reaction site (Fig. 4e). We subsequently attempted the acid-catalyzed alkylation of **44** to **45** using trichloroacetimidate **46** (Fig. 4f). Benzylic trichloroacetimidates are expected to release carbocation species via an S_N_1 mechanism upon activation of the LG by protonation^30,31^. However, the use of TfOH (0.2 equiv.) as an acid catalyst for **46** did not yield any product. This can be attributed to the neutralization of TfOH by amine **44**. Increasing the amount of TfOH to 2 equiv. led to the decomposition of **46**. However, the desired product **45** could not be obtained because amine **44** was completely protonated by the excess TfOH, and thus, lost its nucleophilicity. These results clearly delineate the limitations of the conventional acid-catalyzed alkylation, where the LG requires activation in the presence of a nucleophile.

## Conclusions

We demonstrated that the methodological limitations of S_N_1 reactions can be overcome by conceptually extracting their highly reactive intermediates in the form of carbocationoids that can be accumulated and preserved in a nucleophile-free solution. The methodology presented in this study has the potential to be applied to other carbocations by modifying the coordinating ability of the ligand, and has the potential to provide new opportunities in carbocation chemistry.

## Methods

### General procedure for the preparation of carbocationoid 3a in CH_2_Cl_2_ (GP-1)

Tf_2_O (37.0 μL, 0.22 mmol, 1.0 equiv.) was added dropwise to a suspension of ligand **4a** (50.0 mg, 0.22 mmol, 1.0 equiv.), benzyl alcohol **5a** (40.6 μL, 0.24 mmol, 1.1 equiv.), pempidine (47.0 μL, 0.26 mmol, 1.2 eqiuv.), and powdered molecular sieves 4 A (33.3 mg, a dehydrating agent to remove residual moisture in reaction mixtures) in CH_2_Cl_2_ (1.33 mL) at –78 °C. After 10 min, the reaction mixture was warmed to 0 °C and stirred for a period of the indicated preservation time. The supernatant was used for the alkylation reactions of nucleophiles.

### Synthesis of (10-Acetoxy)decyl 4-(*tert*-butyl)benzyl ether (7a)

A solution of carbocationoid **3a** in CH_2_Cl_2_ [prepared from ligand **4a** (50.0 mg, 0.22 mmol) following GP-1, the preservation time of 1 h] was added to a suspension of nucleophile **6**^32^ (21.6 mg, 0.10 mmol), pempidine (37.9 μL, 0.21 mmol), and powdered molecular sieves 4 A (41.7 mg) in 1,4-dioxane (0.33 mL) at room temperature. After 19 h, the reaction mixture was passed through a silica pad (EtOAc as an eluent). The eluent was concentrated under reduced pressure. The residue was purified by silica gel column chromatography (hexane/EtOAc = 9:1) and preparative thin layer chromatography (hexane/EtOAc = 19:1) to afford a clear colorless oil (32.0 mg, 88%). ^1^H NMR (600 MHz, CDCl_3_): *δ* 7.39–7.35 (m, 2H), 7.29–7.25 (m, 2H), 4.47 (s, 2H), 4.05 (t, *J* = 6.9 Hz, 2H), 3.46 (t, *J* = 6.7 Hz, 2H), 2.04 (s, 3H), 1.65–1.57 (m, 4H), 1.40–1.22 (m, 21H); ^13^C{^1^H} NMR (150 MHz, CDCl_3_): *δ* 171.4, 150.5, 135.8, 127.6, 125.4, 72.8, 70.6, 64.8, 34.6, 31.5, 29.9, 29.62, 29.58, 29.4, 28.7, 26.3, 26.0, 21.2; HRMS (DART): calcd for C_23_H_39_O_3_ [M + H]^+^: 363.2899; found: 363.2905.

### General procedure for the preparation of carbocationoid 3b in CH_2_Cl_2_ (GP-2)

Tf_2_O (65.4 µL, 0.40 mmol, 1.0 equiv.) was added dropwise to a suspension of ligand **4b** (90.5 mg, 0.40 mmol, 1.0 equiv.) and powdered molecular sieves 4 A (53.2 mg) in CH_2_Cl_2_ (1.20 mL) at –78 °C. After 30 min, a solution of pempidine (86.6 µL, 0.48 mmol, 1.2 equiv.) and benzyl alcohol **5b** (84.6 mg, 0.44 mmol, 1.1 equiv.) in CH_2_Cl_2_ (1.46 mL) was added dropwise at –78 °C. After 5 min, the reaction mixture was warmed to 0 °C and stirred for a period of the indicated preservation time. The supernatant was used for the alkylation reactions of nucleophiles.

### Synthesis of (10-Acetoxy)decyl (4-*tert*-butyl-2,6-dimethyl)benzyl ether (7b)

A solution of carbocationoid **3b** in CH_2_Cl_2_ [prepared from ligand **4b** (90.5 mg, 0.40 mmol) following GP-2, the preservation time of 20 h] was added to a suspension of nucleophile **6**^32^ (43.2 mg, 0.20 mmol), pempidine (83.0 μL, 0.46 mmol), and powdered molecular sieves 4 A (66.6 mg) in 1,4-dioxane (0.67 mL) at room temperature. After 22 h, the reaction mixture was treated with H_2_O (0.1 mL). After 10 min, the mixture was passed through a silica pad (EtOAc as an eluent). The eluent was concentrated under reduced pressure. The residue was purified by silica gel column chromatography (hexane/EtOAc = 9:1) and preparative thin layer chromatography (hexane/EtOAc = 9:1) to afford a clear colorless oil (68.1 mg, 87%). ^1^H NMR (600 MHz, CDCl_3_): *δ* 7.03 (s, 2H), 4.47 (s, 2H), 4.05 (t, *J* = 6.8 Hz, 2H), 3.49 (t, *J* = 6.5 Hz, 2H), 2.39 (s, 6H), 2.05 (s, 3H), 1.65–1.55 (m, 4H), 1.40–1.22 (m, 21H); ^13^C{^1^H} NMR (150 MHz, CDCl_3_): *δ* 171.4, 150.7, 137.5, 131.8, 125.4, 70.8, 67.1, 64.8, 34.4, 31.4, 30.0, 29.65, 29.59, 29.57, 29.4, 28.7, 26.4, 26.0, 21.2, 20.0; HRMS (DART): calcd for C_25_H_43_O_3_ [M + H]^+^: 391.3212; found: 391.3204.

### General information, experimental procedure and characterization data

For general information, see Supplementary Methods (page S11). For experimental procedure and characterization data, see Supplementary Methods (pages S12–S27).

### NMR spectra

For ^1^H, ^13^C{^1^H}, and 2D NMR spectra, see Supplementary Data 1.

### Supplementary information

Supplemental Information

Description of Additional Supplementary Files

Supplementary Data

## Data Availability

All data supporting the findings of this study are available within this article and its Supplementary Information, or from the corresponding author upon reasonable request. NMR spectra are available in Supplementary Data 1.
