# Supplementary material for: Carbocationoids, a concept for controlling highly reactive cationic species
Source: Commun Chem. 2024 Mar 13;7:55. doi: 10.1038/s42004-024-01139-w (PMC10937719; doi:10.1038/s42004-024-01139-w)
Supplement: Supplementary file 1 — Supplemental Information [file 42004_2024_1139_MOESM1_ESM.pdf]

Supplementary Information

Carbocationoids, a concept for controlling highly reactive cationic species

Hikaru Fujita[1], Daichi Shimada[1], Jotaro Kudo[1], Kazuyuki Kosha[1], Satoshi Kakuyama[1], Hiromitsu Terasaki[1], Munetaka Kunishima[1,2]*

[1]Faculty of Pharmaceutical Sciences, Institute of Medical, Pharmaceutical, and Health Sciences, Kanazawa University, Kakuma-machi, Kanazawa 920-1192, Japan
[2]Faculty of Pharmaceutical Sciences, Kobe Gakuin University, 1-1-3 Minatojima, Chuo-ku, Kobe 650-8586, Japan

E-mail for Correspondence
kunisima@pharm.kobegakuin.ac.jp

Table of Contents

1. Supplementary Tables 1 and 2 S2
2. Supplementary Figures 1–7 S4
3. Supplementary Methods S11
General information S11
Experimental procedures and characterization data S12
4. Supplementary References S28

1. Supplementary Tables 1 and 2

Supplementary Table 1 | Summary of ^{1}H NMR (600 MHz, $CDCl_3$) and $^{13}C\{^1H\}$ NMR (150 MHz, $CDCl_3$) spectroscopic data for carbocationoid 3a.

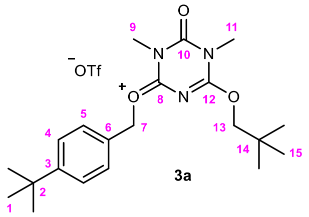

Chemical shifts* (ppm)		
Position	δ_H	δ_C
1	1.33 (s, 9H)	31.3
2		34.6
3		153.0
4	7.43 (d, *J* = 8.4 Hz, 2H)	125.9
5	7.42 (d, *J* = 8.4 Hz, 2H)	129.2
6		129.4
7	5.64 (s, 2H)	75.5
8		162.7
9	3.45 (s, 3H)	30.8
10		148.0
11	3.52 (s, 3H)	30.6
12		162.8
13	4.39 (s, 2H)	82.9
14		31.7
15	1.07 (s, 9H)	26.2

*Signals were assigned with reference to HMQC and HMBC spectra.

Supplementary Table 2 | Summary of ^{1}H NMR (600 MHz, $CDCl_3$) and ^{13}C{^{1}H} NMR (150 MHz, $CDCl_3$) spectroscopic data for carbocationoid 3b.

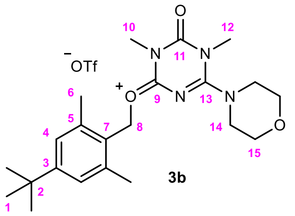

3b

Chemical shifts* (ppm)		
Position	δ_H	δ_C
1	1.31 (s, 9H)	31.2
2		34.5
3		152.8
4	7.09 (s, 2H)	125.6
5		138.6
6	2.40 (s, 6H)	19.8
7		126.2
8	5.59 (s, 2H)	69.0
9		159.0
10	3.32 (s, 3H)	30.1
11		150.2
12	3.52 (s, 3H)	37.7
13		159.4
14	3.91 (br s, 4H)	49.5
15	3.91 (br s, 4H)	66.2

*Signals were assigned with reference to HMQC and HMBC spectra.

2. Supplementary Figures 1–7

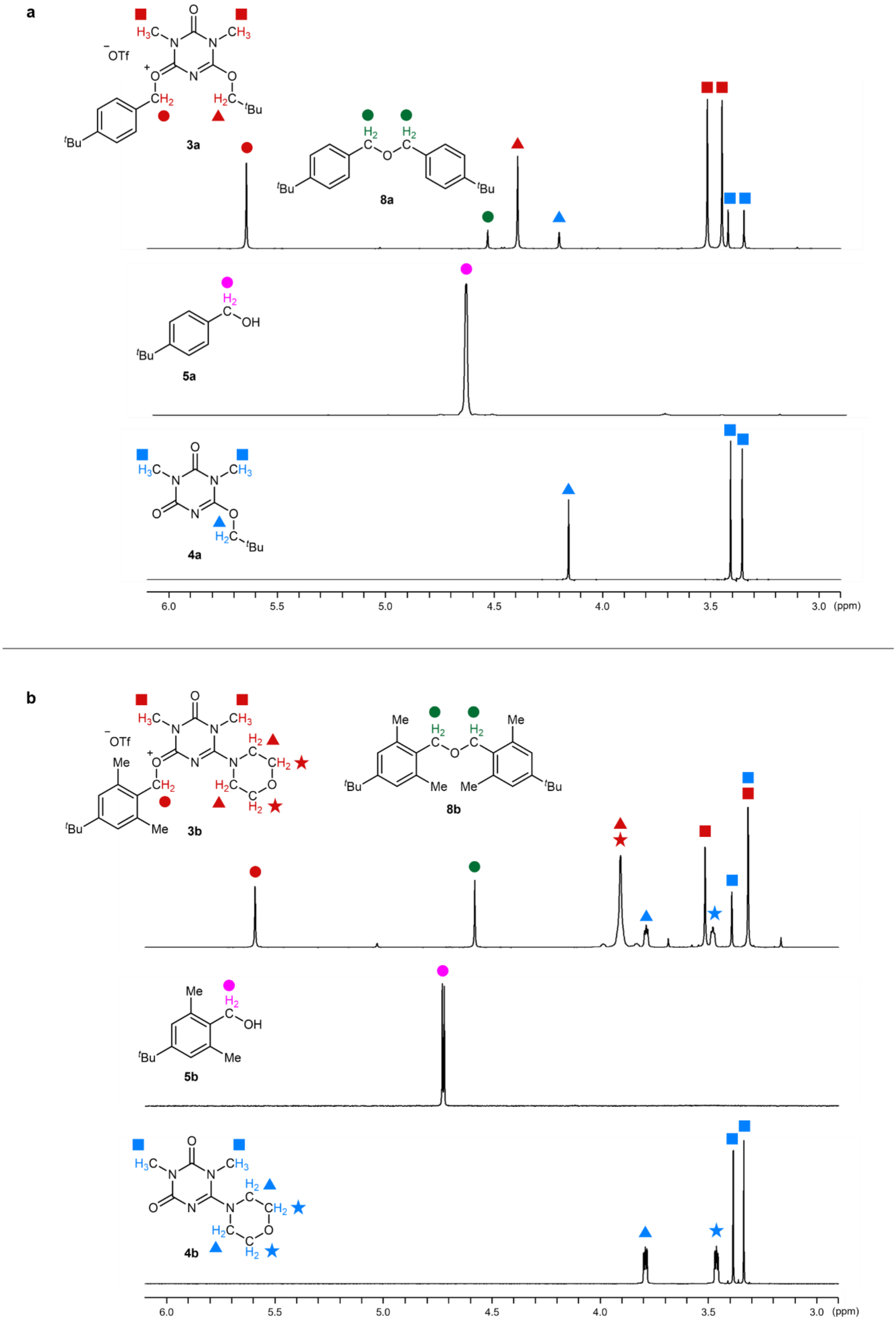

Supplementary Figure 1 | Comparison of ^{1}H NMR spectral profiles (600 MHz, $CDCl_3$, 20 °C). a, ^{1}H NMR spectral profiles recorded for carbocationoid **3a**, benzyl alcohol **5a**, and ligand **4a**. **b**, ^{1}H NMR spectral profiles recorded for carbocationoid **3b**, benzyl alcohol **5b**, and ligand **4b**.

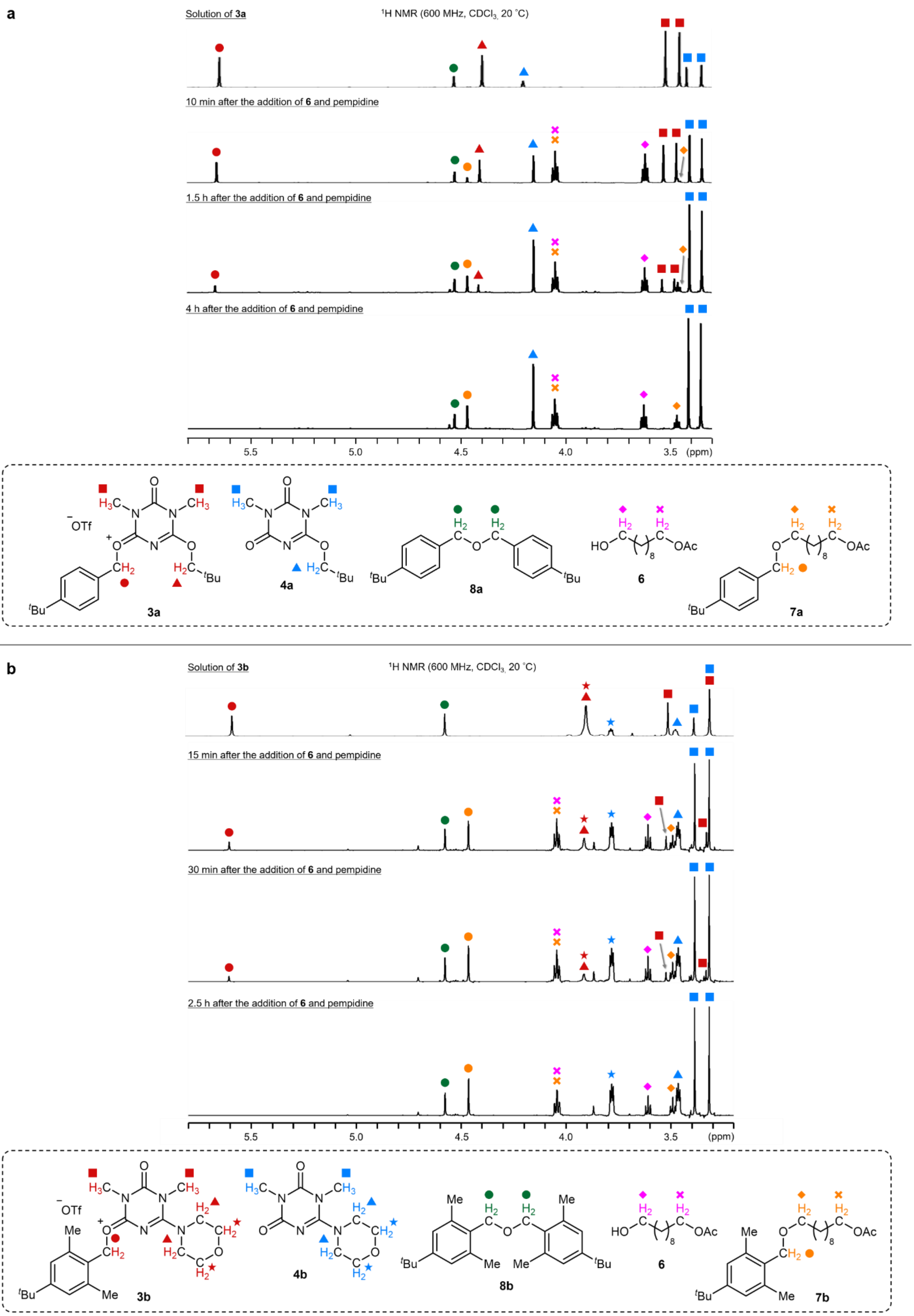

Supplementary Figure 2 | Progress of reactions between carbocationoid 3 and nucleophile 6 at room temperature. a, ^1H NMR monitoring (600 MHz, $CDCl_3$, 20 °C) of reaction of carbocationoid **3a**. **b**, ^1H NMR monitoring (600 MHz, $CDCl_3$, 20 °C) of reaction of carbocationoid **3b**.

a

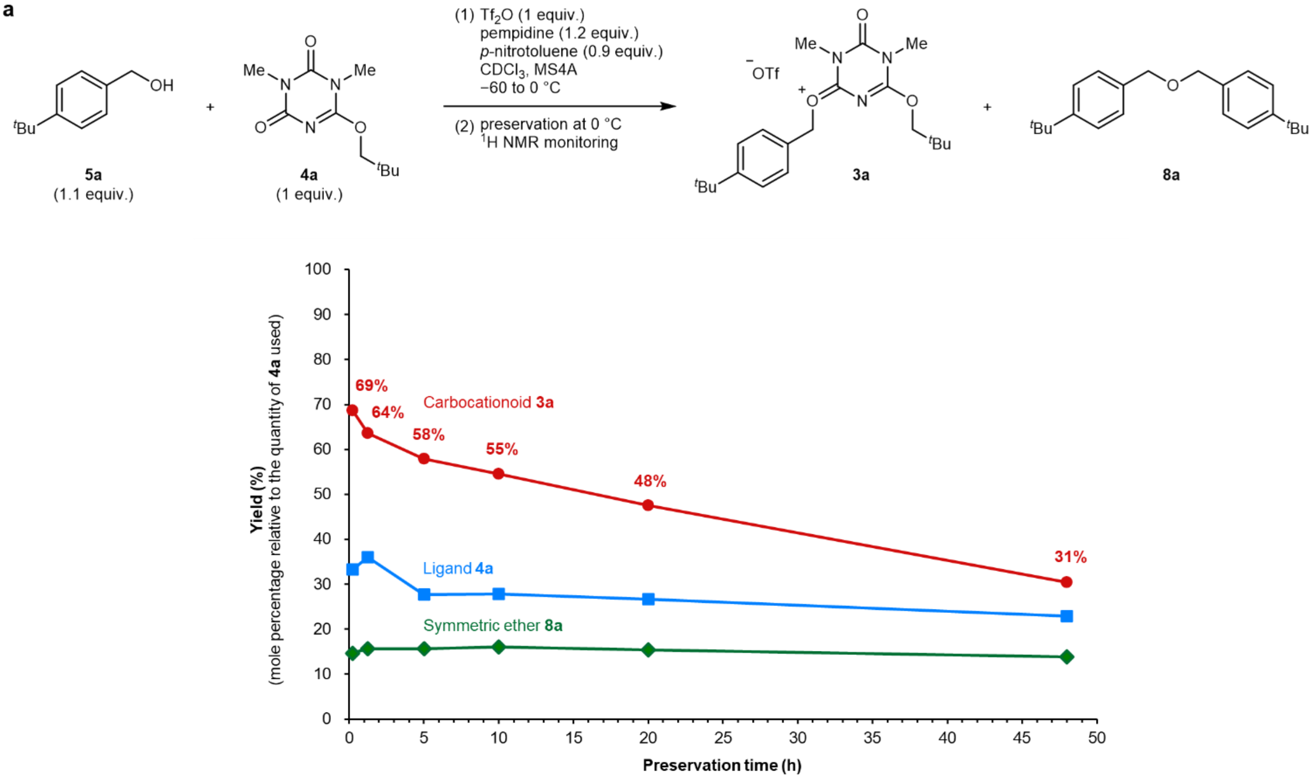

b

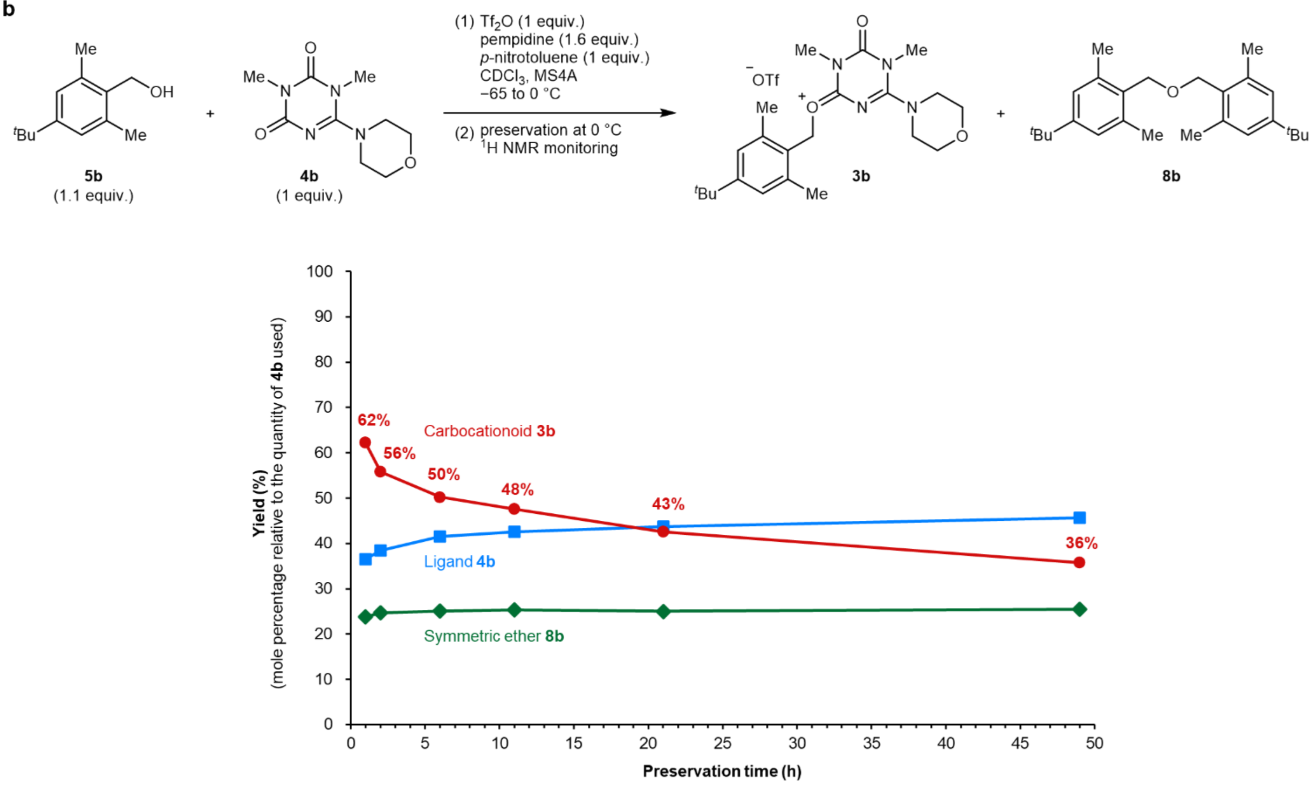

Supplementary Figure 3 | Time course of $CDCl_3$ solution containing carbocationoid 3. a, Carbocationoid **3a**. **b**, Carbocationoid **3b**.

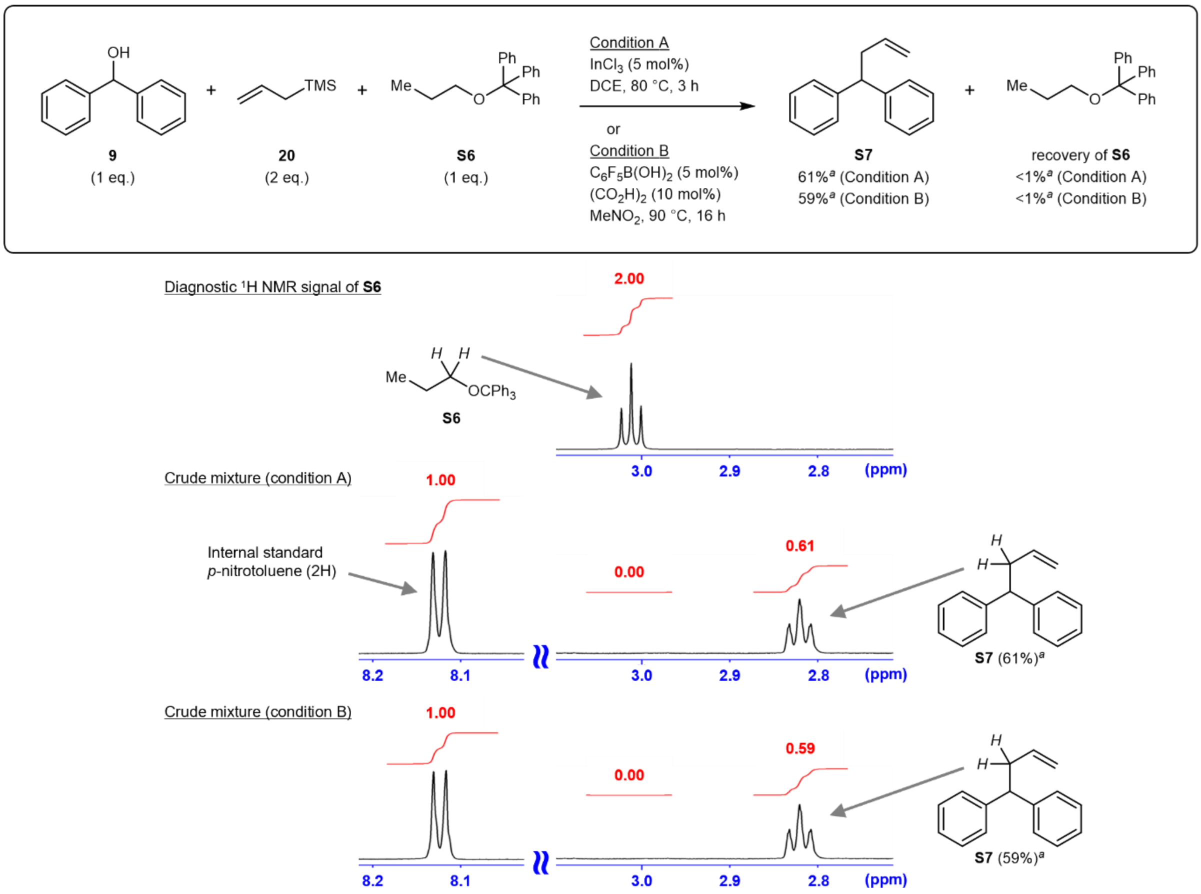

Supplementary Figure 4 | Decomposition of triphenylmethyl ether S6 under the reaction conditions for S_N1-type acid-catalyzed alkylation using alcohol 9 as an electrophile.

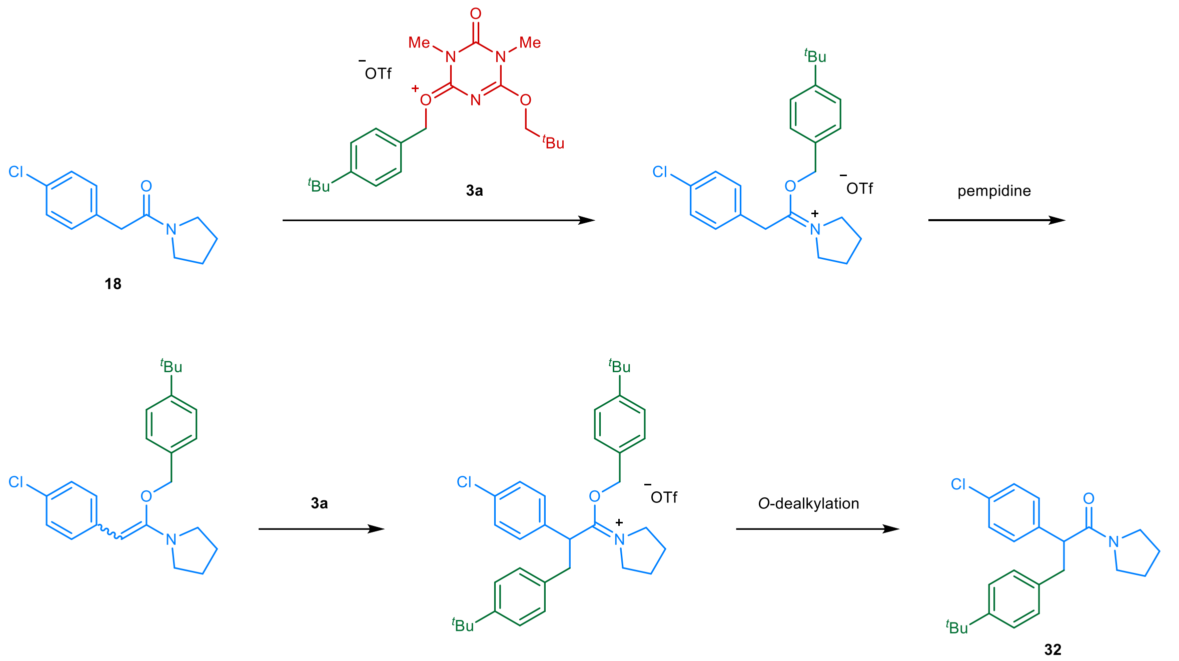

Supplementary Figure 5 | Possible reaction mechanism for the alkylation of amide 18 with carbocationoid 3a.

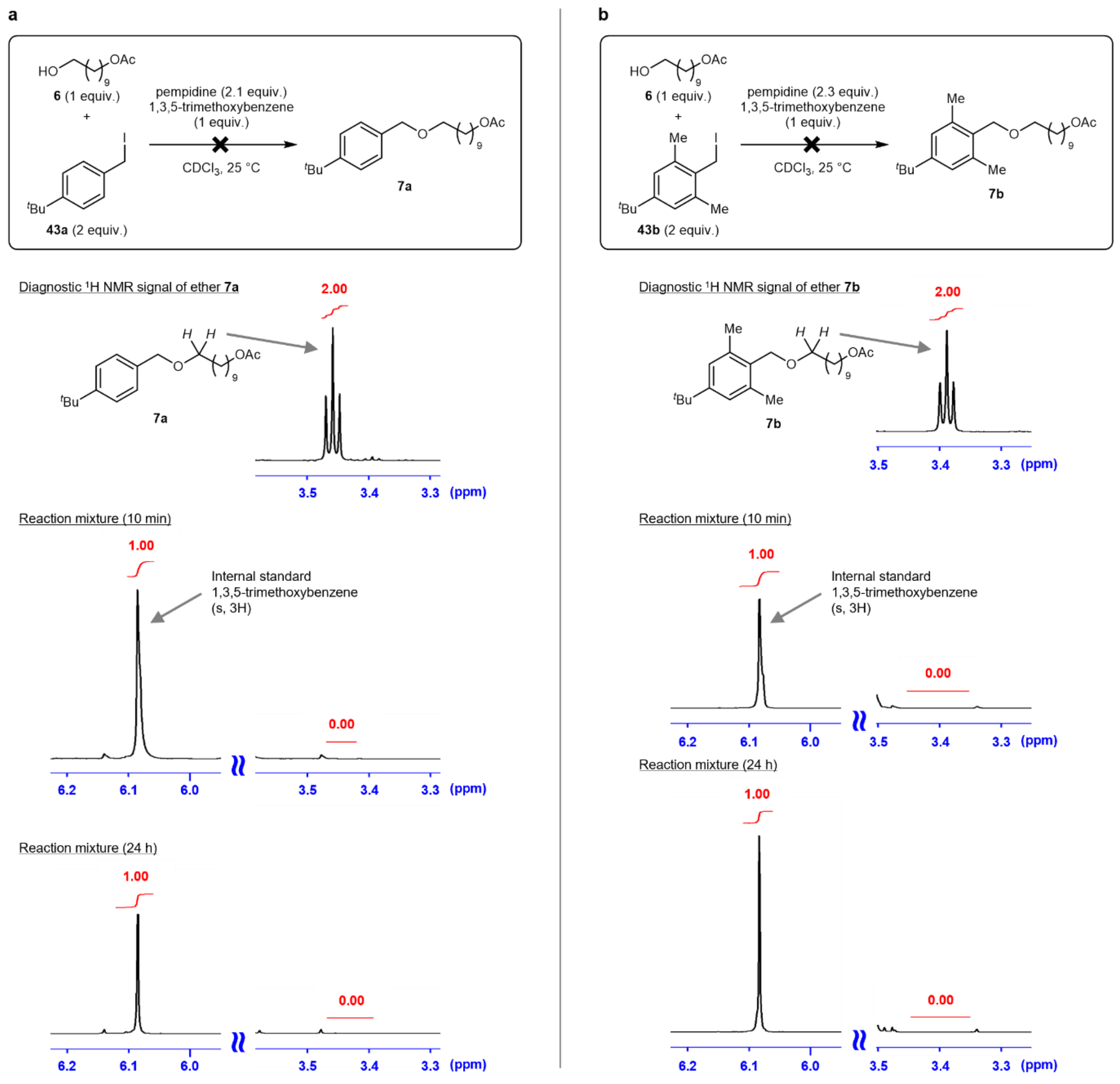

Supplementary Figure 6 | ^{1}H NMR monitoring (600 MHz, $CDCl_3$, 20 °C) of attempted reactions between nucleophile 6 and benzyl iodide 43. a, Benzyl iodide **43a. b,** Benzyl iodide **43b.**

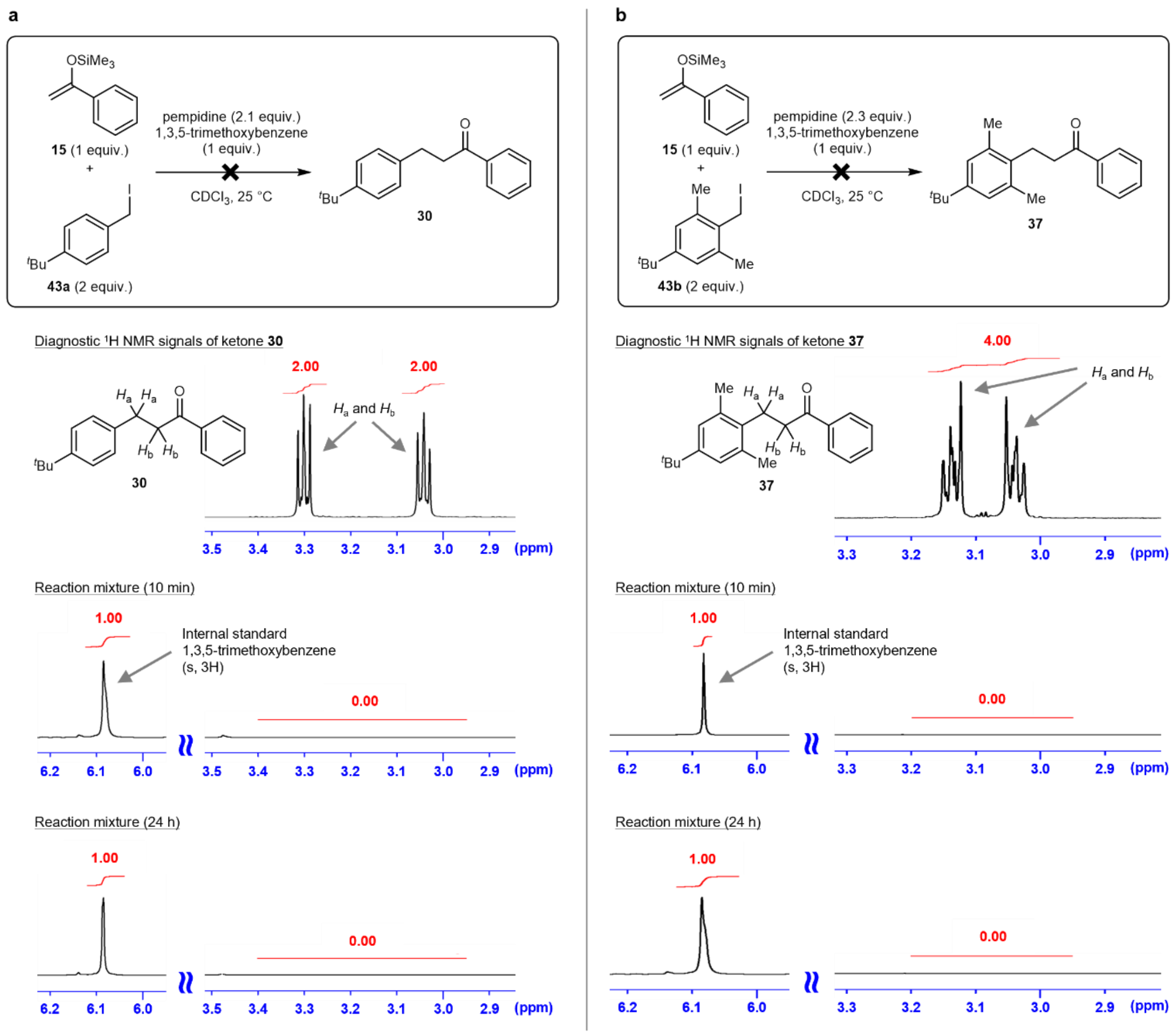

Supplementary Figure 7 |^{1}H NMR monitoring (600 MHz, CDCl$_3$, 20 °C) of attempted reactions between nucleophile 15 and benzyl iodide 43. a, Benzyl iodide **43a. b,** Benzyl iodide **43b.**

3. Supplementary Methods

General information

NMR spectra were recorded on a JEOL JNM-ECS400 spectrometer [^{1}H NMR (400 MHz)], a JEOL JNM-ECA600 spectrometer [^{1}H NMR (600 MHz), ^{13}C{^{1}H} NMR (150 MHz)], or a JEOL JNM-ECZ600R [^{1}H NMR (600 MHz), ^{13}C{^{1}H} NMR (150 MHz)] at 20 °C unless otherwise noted. Chemical shifts for ^{1}H NMR are reported in parts per million (δ) relative to tetramethylsilane (TMS, δ 0.00) as the internal standard. Coupling constants (J) are reported in hertz (Hz). The following abbreviations are used for spin multiplicity: s = singlet, d = doublet, t = triplet, q = quartet, quin = quintet, m = multiplet, br = broad. Chemical shifts for ^{13}C{^{1}H} NMR are reported in parts per million (δ) relative to the solvent ($CDCl_3$, δ 77.16). Mass spectra were measured on a Micromass Zq2000 spectrometer (ESI-MS), an LCMS-2050 spectrometer (ESI-MS), a JMS-T100TD AccuTOF TLC spectrometer (DART-MS and ESI-MS), or JMS-700 spectrometer (FAB). IR spectra were measured with a Fisher Scientific Nicolet IS5 FT-IR spectrometer equipped with an iD7 diamond ATR accessory. Melting points were determined with a Yanagimoto melting point apparatus and were uncorrected. Analytical thin layer chromatography (TLC) was performed using glass plates precoated with 0.25 mm silica gel impregnated with a fluorescent indicator (254 nm). Preparative TLC separations were performed using glass plates precoated with 0.50 mm silica gel impregnated with a fluorescent indicator (254 nm). Column chromatography was performed using silica (spherical, neutral, 40–100 μm), amine-functionalized silica (60 μm), carboxylic acid-functionalized silica (40–75 μm), diol-functionalized silica (40–75 μm), or reverse phase silica (C18, 40–75 μm). Recycling preparative HPLC was performed with Japan Analytical Industry LC-928 equipped with gel permeation chromatography columns Jaigel-1H and 2H. All reactions sensitive to oxygen or moisture were conducted under a nitrogen atmosphere. Reagents were commercial grades and were used without any purification unless otherwise noted. Dehydrated CH_2Cl_2, 1,4-dioxane, MeOH, and MeCN were purchased from commercial sources. $^{i}Pr_2EtN$, *N*-methylpyrrolidine, morpholine, trifluoromethanesulfonic anhydride (Tf_2O), pyrrolidine, 1,8-diazabicyclo[5.4.0]undec-7-ene (DBU), and nucleophile **13** were purchased from commercial sources and distilled before use. Molecular sieves 4A (a dehydrating agent to remove residual moisture in reaction mixtures) were used in powder form unless otherwise noted and were activated by heating under vacuum before use.

Experimental procedures and characterization data

2,4-Dimethoxy-6-(neopentyloxy)-1,3,5-triazine (S1)

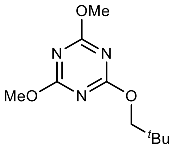

2,4,6-Trichloro-1,3,5-triazine (4.04 g, 21.9 mmol) was added to a suspension of neopentyl alcohol (2.90 g, 32.9 mmol), 1,10-phenanthroline (8.96 g, 43.9 mmol), and molecular sieves 4A (1.82 g) in CH_2Cl_2 (73.1 mL) at room temperature. After 18 h, the reaction mixture was cooled to 0 °C. Subsequently, $^{i}Pr_2EtN$ (13.4 mL, 78.8 mmol), MeOH (73.1 mL), and *N*-methylpyrrolidine (0.230 mL, 2.19 mmol) were added. The reaction mixture was stirred for 1 h at room temperature and then filtered. The filtrate was diluted with CH_2Cl_2 (40 mL) and washed with aqueous HCl (1 M, 60 mL) and brine (30 mL). The organic layer was dried (Na_2SO_4), filtered, and concentrated under reduced pressure to afford a white solid (5.04 g, 94%). ^{1}H NMR (600 MHz, $CDCl_3$): δ 4.09 (s, 2H), 4.03 (s, 6H), 1.04 (s, 9H); $^{13}C\{^{1}H\}$ NMR (150 MHz, $CDCl_3$): δ 173.64, 173.61, 77.9, 55.4, 31.7, 26.6; HRMS (DART): calcd for $C_{10}H_{18}N_3O_3$ $[M + H]^+$: 228.1348; found: 228.1340.

1,3-Dimethyl-6-(neopentyloxy)-1,3,5-triazine-2,4(1*H*,3*H*)-dione (4a)

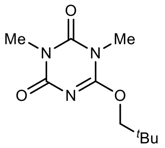

Following a reported procedure for the methyl group rearrangement of 2,4,6-trimethoxy-1,3,5-triazine[1], a solution of **S1** (2.00 g, 8.80 mmol) in 1,4-dioxane (11.0 mL) and iodomethane (11.0 mL) was heated at 100 °C in an autoclave. After 14 h, the reaction mixture was cooled to room temperature and then concentrated under reduced pressure. The residue was purified by column chromatography (silica, hexane/EtOAc = 2:3) to afford a white solid (1.79 g, 90%). Mp: 47.1–47.9 °C; ^{1}H NMR (600 MHz, $CDCl_3$): δ 4.16 (s, 2H), 3.41 (s, 3H), 3.35 (s, 3H), 1.03 (s, 9H); $^{13}C\{^{1}H\}$ NMR (150 MHz, $CDCl_3$): δ 159.2, 154.8, 151.2, 79.7, 31.6, 29.24, 29.21, 26.4; HRMS (DART): calcd for $C_{10}H_{18}N_3O_3$ $[M + H]^+$: 228.1348; found: 228.1351; IR (ATR): 2968, 1738, 1681, 1595, 1470, 1429, 1347, 1224, 955, 776 cm^{-1}.

2,4-Dimethoxy-6-phenoxy-1,3,5-triazine (S2)[2]

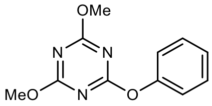

Phenol (2.26 g, 24.0 mmol), $^{i}Pr_2EtN$ (5.23 mL, 30.0 mmol), and *N*-methylpyrrolidine (0.0208 mL, 0.20 mmol) were added to a solution of 2-chloro-4,6-dimethoxy-1,3,5-triazine (3.51 g, 20.0 mmol) in CH_2Cl_2 (40.0 mL) at 0 °C. The reaction mixture was stirred for 1 h at 0 °C and then diluted with CH_2Cl_2 (50 mL). The solution was washed with aqueous HCl (1 M, 50 mL) and brine (25 mL). The organic layer was dried (Na_2SO_4) and filtered. The filtrate was concentrated under reduced pressure. The residue was purified by column chromatography (silica, hexane/EtOAc = 4:1) to afford a white solid (3.99 g, 86%). ^{1}H NMR (400 MHz, $CDCl_3$): δ 7.47–7.36 (m, 2H), 7.31–7.22 (m, 1H), 7.22–7.14 (m, 2H), 4.00 (s, 6H); $^{13}C\{^{1}H\}$ NMR (150 MHz, $CDCl_3$): δ 174.0, 173.4, 151.9, 129.7, 126.1, 121.7, 55.6; LRMS (ESI): *m*/*z* 256 ($[M + Na]^+$).

1,3-Dimethyl-6-phenoxy-1,3,5-triazine-2,4(1*H*,3*H*)-dione (S3)

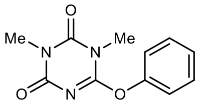

Following a reported procedure for the methyl group rearrangement of 2,4,6-trimethoxy-1,3,5-triazine[1], a suspension of **S2** (3.99 g, 17.1 mmol) in 1,4-dioxane (11.4 mL) and iodomethane (11.4 mL) was heated at 100 °C in an autoclave. After 72 h, the reaction mixture was cooled to room temperature and then concentrated under reduced pressure. The residue was purified by column chromatography (silica, hexane/EtOAc = 1:1) to afford a white solid (3.47 g, 87%). ^{1}H NMR (600 MHz, $CDCl_3$): *δ* 7.47–7.39 (m, 2H), 7.33–7.27 (m, 1H), 7.19–7.14 (m, 2H), 3.58 (s, 3H), 3.36 (s, 3H); ^{13}C{^{1}H} NMR (150 MHz, $CDCl_3$): *δ* 159.1, 154.3, 151.2, 150.7, 130.0, 127.1, 121.5, 30.1, 29.4; HRMS (DART): calcd for $C_{11}H_{12}N_3O_3$ [M + H]$^+$: 234.0879; found: 234.0888.

1,3-Dimethyl-6-morpholino-1,3,5-triazine-2,4(1*H*,3*H*)-dione (4b)

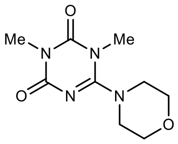

Morpholine (1.15 mL, 13.3 mmol) was added to a solution of **S3** (1.00 g, 4.42 mmol) in MeCN (6.31 mL) at room temperature. After 1.5 h, the reaction mixture was quenched by the addition of AcOH (0.885 mL, 15.5 mmol) and then concentrated under reduced pressure. The residue was purified by column chromatography (silica, hexane/acetone = 1:1) afforded a white solid (871 mg, 90%). Mp: 102.8–103.1 °C; ^{1}H NMR (600 MHz, $CDCl_3$): *δ* 3.79 (t, *J* = 4.7 Hz, 4H), 3.46 (t, *J* = 4.7 Hz, 4H), 3.38 (s, 3H), 3.33 (s, 3H); ^{13}C{^{1}H} NMR (150 MHz, $CDCl_3$): *δ* 160.1, 154.8, 153.0, 66.3, 48.9, 35.4, 29.1; HRMS (DART): calcd for $C_9H_{15}N_4O_3$ [M + H]$^+$: 227.1144; found: 227.1134; Anal. calcd for $C_9H_{14}N_4O_3$; C, 47.78; H, 6.24; N, 24.77; found: C, 47.71; H, 6.23; N, 24.79; IR (ATR): 2971, 2913, 1728, 1653, 1540, 1425, 1202, 1111, 1028, 946, 860, 775 cm^{-1}.

(4-*tert*-Butyl-2,6-dimethyl)benzyl chloride (S4)[3]

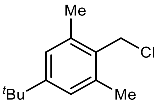

A solution of 1-(*tert*-butyl)-3,5-dimethylbenzene (1.90 mL, 10.2 mmol), aqueous formaldehyde (37 w/w % in H_2O, 812 μL, 11.0 mmol), aqueous HCl (12 M, 2.9 mL, 35 mmol) in AcOH (8.3 mL) was heated at 60 °C for 3 days and then cooled to room temperature. The reaction mixture was diluted with H_2O (25 mL) and extracted with CH_2Cl_2 (25 mL). The organic layer was washed with brine, dried (Na_2SO_4), and filtered. The filtrate was concentrated under reduced pressure. The residue was purified by column chromatography (silica, hexane) to afford a colorless oil (2.04 g, 95%). ^{1}H NMR (400 MHz, $CDCl_3$): *δ* 7.06 (s, 2H), 4.66 (s, 2H), 2.43 (s, 6H), 1.29 (s, 9H; ^{13}C{^{1}H} NMR (150 MHz, $CDCl_3$): *δ* 151.7, 137.2, 131.2, 125.6, 41.4, 34.5, 31.4, 19.7; LRMS (FAB): *m*/*z* 175 ([M − Cl]$^+$).

(4-*tert*-Butyl-2,6-dimethyl)benzyl alcohol (5b)[4]

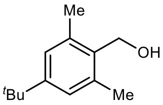

A mixture of **S4** (2.10 g, 9.96 mmol) in 1,4-dioxane (228 mL) and aqueous NaOH (2.1 M, 66 mL) was heated at 100 °C for 3 days and then cooled to room temperature. The reaction

mixture was diluted with H_2O (200 mL) and extracted with CH_2Cl_2 (200 mL). The organic layer was washed with brine (600 mL), dried (Na_2SO_4), and filtered. The filtrate was concentrated under reduced pressure. The residue was purified by column chromatography (silica, hexane/EtOAc = 4:1) to afford a white solid (1.70 g, 89%). ^{1}H NMR (600 MHz, $CDCl_3$): δ 7.06 (s, 2H), 4.72 (d, J = 5.4 Hz, 2H), 2.44 (s, 6H), 1.30 (s, 9H), 1.19 (t, J = 5.4 Hz, 1H); ^{13}C{^{1}H} NMR (150 MHz, $CDCl_3$): δ 151.1, 137.1, 133.9, 125.6, 59.5, 34.5, 31.4, 19.9; LRMS (ESI): *m*/*z* 215.2 ([M + Na]$^+$).

Attempted synthesis of ether 7a without the use of ligand 4a (Table 1, entry 3)

Tf_2O (37.0 μL, 0.22 mmol) was added dropwise to a suspension of benzyl alcohol **5a** (40.6 μL, 0.24 mmol), pempidine (47.0 μL, 0.26 mmol), and molecular sieves 4A (33.3 mg) in CH_2Cl_2 (1.33 mL) at –78 °C. After 10 min, the reaction mixture was warmed to 0 °C and stirred for 1 h. The supernatant was added to a suspension of nucleophile **6** (21.6 mg, 0.10 mmol), pempidine (37.9 μL, 0.21 mmol), and molecular sieves 4A (41.7 mg) in 1,4-dioxane (0.33 mL) at room temperature. After 19 h, the reaction mixture was passed through a silica pad (EtOAc as an eluent). The eluent was concentrated under reduced pressure. The alkylated product **7a** was not detected by ^{1}H NMR spectroscopic analysis of the crude mixture.

Attempted synthesis of ether 7b without the use of ligand 4b (Table 1, entry 6)

Tf_2O (32.7 μL, 0.20 mmol) was added to a solution of benzyl alcohol **5b** (38.4 mg, 0.20 mmol) and pempidine (39.7 μL, 0.22 mmol) in CH_2Cl_2 (1.33 mL) at −78 °C. After 1 h, a solution of nucleophile **6** (21.6 mg, 0.10 mmol) and pempidine (37.9 μL, 0.21 mmol) in CH_2Cl_2 (0.33 mL) was added at −78 °C. The reaction mixture was allowed to warm to room temperature. After 3 h, the reaction mixture was quenched by the addition of pyridine (40.2 μL). After 10 min, the mixture was passed through a silica pad (EtOAc as an eluent). The eluent was concentrated under reduced pressure. The yields of ether **7b** (<3% based on **6**) and symmetric ether **8b** (95% based on **5b**) were calculated by analyzing the ^{1}H quantitative NMR spectral profile of the crude mixture using coumarin as the internal standard. The crude mixture was purified by column chromatography (silica, hexane/EtOAc = 19:1) and preparative TLC (hexane/EtOAc = 19:1) to afford symmetric ether **8b** as a pale yellow solid (31.5 mg, 86% based on **5b**).

Bis(4-*tert*-butyl-2,6-dimethyl)benzyl ether (8b)

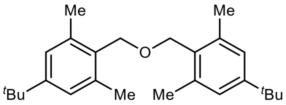

^{1}H NMR (400 MHz, $CDCl_3$): δ 7.01 (s, 4H), 4.58 (s, 4H), 2.39 (s, 12H), 1.26 (s, 18H); ^{13}C{^{1}H} NMR (150 MHz, $CDCl_3$): δ 150.8, 137.6, 131.6, 125.3, 66.9, 34.4, 31.4, 19.9; HRMS (DART): calcd for $C_{26}H_{39}O$ [M + H]$^+$: 367.3001; found: 367.2994.

1,2,2,6,6-Pentamethylpiperidin-1-ium trifluoromethanesulfonate (S5)

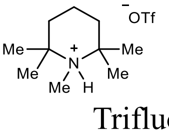

Trifluoromethanesulfonic acid (TfOH, 88.5 μL, 1.0 mmol) was added to a solution of pempidine (190.0 μL, 1.05 mmol) in Et_2O (3.3 mL) at 0 °C. After 2 h, the precipitate was separated by decantation to afford a white solid (266.2 mg, 87%). Mp: 159.3–161.1 °C; ^{1}H NMR(600 MHz, $CDCl_3$): δ 8.19 (br s, 1H), 2.76 (d, J = 5.0 Hz, 3H), 2.20–2.08 (m, 2H), 1.82–1.67 (m, 4H), 1.54 (s, 6H), 1.37 (s, 6H); ^{13}C{^{1}H} NMR (150 MHZ, $CDCl_3$): δ 120.5 (q, J = 317.4 Hz), 64.9, 37.5, 29.5, 29.4, 20.7, 16.0; HRMS (ESI): calcd for $C_{10}H_{22}N$ [M − CF_3SO_3]$^+$:

156.1752; found:156.1756.

Bis(4-*tert*-butylbenzyl) ether (8a)[5]

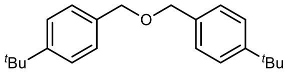

An authentic sample of **8a** was obtained from the alkylation reactions using carbocationoid **3a**. ^{1}H NMR (600 MHz, $CDCl_3$): δ 7.40–7.35 (m, 4H), 7.32–7.28 (m, 4H), 4.52 (s, 4H), 1.32 (s, 18H); ^{13}C{^{1}H} NMR (150 MHz, $CDCl_3$): δ 150.7, 135.5, 127.8, 125.4, 72.0, 34.7, 31.5; LRMS (ESI): *m*/*z* 333 ([M + Na]$^+$).

Preparation of carbocationoid 3a in $CDCl_3$ for NMR measurements

Tf_2O (72.2 µL, 0.44 mmol) was added dropwise to a mixture of ligand **4a** (100.0 mg, 0.44 mmol), benzyl alcohol **5a** (85.0 µL, 0.50 mmol), pempidine (94.1 µL, 0.52 mmol), and molecular sieves 4A (pellets, 293.0 mg) in $CDCl_3$ (2.90 mL) at −60 °C. After 10 min, the reaction mixture was warmed to 0 °C and stirred at this temperature for 10 min. An aliquot of the supernatant (0.60 mL) was transferred to an NMR tube and immediately used for NMR measurements [^{1}H NMR (600 MHz) and ^{13}C{^{1}H} NMR (150 MHz)]. Two additional aliquots of the supernatant (2 × 0.60 mL) were also taken and used for HMQC and HMBC experiments, respectively. The ^{1}H and ^{13}C{^{1}H} NMR data are summarized in Table S1.

Preparation of carbocationoid 3b in $CDCl_3$ for NMR measurements

Tf_2O (131.2 µL, 0.80 mmol) was added dropwise to a mixture of ligand **4b** (181.0 mg, 0.80 mmol) and molecular sieves 4A (pellets, 240 mg) in $CDCl_3$ (2.40 mL) at −60 °C. After 30 min, a solution of benzyl alcohol **5b** (169.2 mg, 0.88 mmol) and pempidine (173.7 µL, 0.96 mmol) in $CDCl_3$ (3.00 mL) was added dropwise at −60 °C. After 5 min, the reaction mixture was warmed to 0 °C and stirred at this temperature for 1 h. An aliquot of the supernatant (0.60 mL) was transferred to an NMR tube and immediately used for NMR measurements [^{1}H NMR (600 MHz) and ^{13}C{^{1}H} NMR (150 MHz)]. Two additional aliquots of the supernatant (2 × 0.60 mL) were also taken and used for HMQC and HMBC experiments, respectively. The ^{1}H and ^{13}C{^{1}H} NMR data are summarized in Table S2.

^{1}H NMR monitoring of the reaction between carbocationoid 3a and nucleophile 6 (Supplementary Fig. 2a)

Tf_2O (72.2 µL, 0.44 mmol) was added dropwise to a mixture of ligand **4a** (100.0 mg, 0.44 mmol), benzyl alcohol **5a** (85.0 µL, 0.50 mmol), pempidine (94.1 µL, 0.52 mmol), and molecular sieves 4A (pellets, 293.0 mg) in $CDCl_3$ (2.90 mL) at −60 °C. After 10 min, the reaction mixture was warmed to 0 °C and stirred at this temperature for 10 min. Aliquots of the supernatant (2 × 0.60 mL) were taken and immediately used for a ^{1}H NMR measurement (600 MHz). A solution of nucleophile **6** (84.3 mg, 0.39 mmol) and pempidine (162.9 µL, 0.90 mmol) in $CDCl_3$ (1.20 mL) was added to the reaction mixture at 0 °C. An aliquot of the supernatant (0.60 mL) was transferred to an NMR tube, which was kept at room temperature. ^{1}H NMR measurements (600 MHz, 20 °C) were performed at intervals to monitor the reaction at room temperature.

^{1}H NMR monitoring of the reaction between carbocationoid 3b and nucleophile 6 (Supplementary Fig. 2b)

Tf_2O (32.7 µL, 0.20 mmol) was added dropwise to a mixture of ligand **4b** (45.2 mg, 0.20 mmol) and molecular sieves 4A (pellets, 26.6 mg) in $CDCl_3$ (0.60 mL) at −60 °C. After 30 min, a solution of benzyl alcohol **5b** (42.3 mg, 0.22 mmol) and pempidine (43.3 µL, 0.24 mmol) in $CDCl_3$ (0.73 mL) was added dropwise at −60 °C. After 5 min, the reaction mixture was warmed to 0 °C and stirred at this temperature for 1 h. An aliquot of the supernatant (0.60 mL) was transferred to an NMR tube and immediately used for a ^{1}H NMR measurement (600 MHz). A solution of nucleophile **6** (34.6 mg, 0.16 mmol) and pempidine (66.4 µL, 0.37 mmol) in $CDCl_3$ (0.53 mL) was added to the reaction mixture at 0 °C. An aliquot of the supernatant (0.60 mL) was transferred to an NMR tube, which was kept at room temperature. ^{1}H NMR measurements (600 MHz, 20 °C) were performed at intervals to monitor the reaction at room temperature.

^{1}H NMR monitoring of the $CDCl_3$ solution containing carbocationoid 3a (Supplementary Fig. 3a)

Tf_2O (108 µL, 0.66 mmol) was added dropwise to a mixture of ligand **4a** (149.9 mg, 0.66 mmol), benzyl alcohol **5a** (122 µL, 0.72 mmol), pempidine (141 µL, 0.78 mmol), *p*-nitrotoluene (82.3 mg, 0.60 mmol), and molecular sieves 4A (pellets, 100 mg) in $CDCl_3$ (4.00 mL) at −60 °C. After 10 min, the reaction mixture was warmed to 0 °C and stirred at this temperature for 10 min. An aliquot of the supernatant (0.60 mL) was transferred to an NMR tube. The temperature of this solution was maintained at 0 °C. ^{1}H NMR measurements (600 MHz) were performed at intervals. The yields of carbocationoid **3a** and remaining **4a** and the mole percentage of symmetric ether **8a** (relative to the quantity of **4a** used) were calculated using *p*-nitrotoluene as the internal standard.

^{1}H NMR monitoring of the $CDCl_3$ solution containing carbocationoid 3b (Supplementary Fig. 3b)

A solution of ligand **4b** (135.7 mg, 0.60 mmol) and *p*-nitrotoluene (82.2 mg, 0.60 mmol) in $CDCl_3$ (2.00 mL) was added dropwise to a mixture of Tf_2O (98.4 µL, 0.60 mmol) and molecular sieves 4A (pellets, 195 mg) in $CDCl_3$ (1.50 mL) at −65 °C. After 30 min, a solution of benzyl alcohol **5b** (126.9 mg, 0.66 mmol) and pempidine (173 µL, 0.96 mmol) in $CDCl_3$ (3.00 mL) was added dropwise at −65 °C. After 5 min, the reaction mixture was warmed to 0 °C and stirred at this temperature for 1 h. An aliquot of the supernatant (0.60 mL) was transferred to an NMR tube. The temperature of this solution was maintained at 0 °C. ^{1}H NMR measurements (600 MHz) were performed at intervals. The yields of carbocationoid **3b** and

remaining **4b** and the mole percentage of symmetric ether **8b** (relative to the quantity of **4b** used) were calculated using *p*-nitrotoluene as the internal standard.

Benzhydryl 4-*tert*-butylbenzyl ether (24)

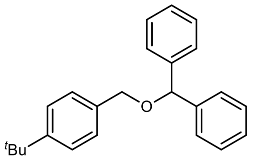

A solution of **3a** in CH_2Cl_2 [prepared from ligand **4a** (0.66 mmol) following GP-1, the preservation time of 10 min] was added to a suspension of nucleophile **9** (36.8 mg, 0.20 mmol), *N*-isobutylmorpholine (99.8 µL, 0.62 mmol), and molecular sieves 4A (125.0 mg) in 1,4-dioxane (1.00 mL) at room temperature. After 4 h, the reaction mixture was treated with H_2O (108 µL). After 10 min, the reaction mixture was passed through a silica pad (EtOAc as an eluent). The eluent was concentrated under reduced pressure. The residue was purified by column chromatography (silica, hexane/EtOAc = 19:1) and recycling preparative HPLC ($CHCl_3$) to afford a clear colorless oil (49.2 mg, 75%). ^1H NMR (600 MHz, $CDCl_3$): δ 7.39–7.35 (m, 6H), 7.35–7.28 (m, 6H), 7.27–7.23 (m, 2H), 5.45 (s, 1H), 4.51 (s, 2H), 1.32 (s, 9H); $^{13}C\{^1H\}$ NMR (150 MHz, $CDCl_3$): δ 150.6, 142.4, 135.5, 128.5, 127.7, 127.6, 127.3, 125.4, 82.5, 70.5, 34.7, 31.5; HRMS (DART): calcd for $C_{24}H_{30}NO$ $[M + NH_4]^+$: 348.2327; found: 348.2330.

4-*tert*-Butylbenzyl 2-phenylpropan-2-yl ether (25)

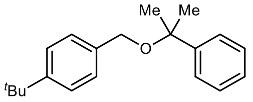

A solution of **3a** in CH_2Cl_2 [prepared from ligand **4a** (0.66 mmol) following GP-1, the preservation time of 10 min] was added to a suspension of nucleophile **10** (27.2 mg, 0.20 mmol), *N*-isobutylmorpholine (99.8 µL, 0.62 mmol), and molecular sieves 4A (125.0 mg) in 1,4-dioxane (1.00 mL) at room temperature. After 4 h, the reaction mixture was treated with H_2O (108 µL). After 10 min, the reaction mixture was passed through a silica pad (EtOAc as an eluent). The eluent was concentrated under reduced pressure. The residue was purified by column chromatography (silica, hexane/EtOAc = 9:1) and recycling preparative HPLC ($CHCl_3$) to afford a clear colorless oil (32.0 mg, 57%). ^1H NMR (600 MHz, $CDCl_3$): δ 7.53–7.49 (m, 2H), 7.40–7.33 (m, 4H), 7.30–7.25 (m, 3H), 4.20 (s, 2H), 1.63 (s, 6H), 1.31 (s, 9H); $^{13}C\{^1H\}$ NMR (150 MHz, $CDCl_3$): δ 150.3, 146.5, 136.5, 128.4, 127.5, 127.1, 126.1, 125.4, 65.0, 34.6, 31.5, 28.7; HRMS (DART): calcd for $C_{20}H_{26}NaO$ $[M + Na]^+$: 305.1881; found: 305.1888.

1-Adamantyl 4-*tert*-butylbenzyl ether (26)

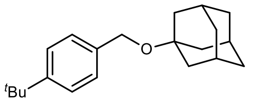

A solution of **3a** in CH_2Cl_2 [prepared from ligand **4a** (75.0 mg, 0.33 mmol) following GP-1, the preservation time of 10 min] was added to a suspension of nucleophile **11** (15.2 mg, 0.10 mmol), *N*-isobutylmorpholine (49.9 µL, 0.31 mmol), and molecular sieves 4A (12.5 mg) in 1,4-dioxane (0.50 mL) at room temperature. After 5 h, the reaction mixture was treated with H_2O (81 µL). After 10 min, the reaction mixture was passed through a silica pad (EtOAc as an eluent). The eluent was concentrated under reduced pressure. The residue was purified by column chromatography (silica, hexane/EtOAc = 19:1) and recycling preparative HPLC ($CHCl_3$) to afford a white solid (19.7 mg, 66%). Mp: 67.7–68.7 °C; ^1H NMR (600 MHz,

$CDCl_3$): δ 7.37–7.32 (m, 2H), 7.30–7.26 (m, 2H), 4.46 (s, 2H), 2.17 (br s, 3H), 1.88–1.83 (m, 6H), 1.70–1.59 (m, 6H), 1.30 (s, 9H); $^{13}C\{^1H\}$ NMR (150 MHz, $CDCl_3$): δ 150.1, 137.1, 127.6, 125.4, 72.7, 62.3, 41.9, 36.7, 34.6, 31.5, 30.7; HRMS (FAB): calcd for $C_{21}H_{31}O$ $[M + H]^+$: 299.2375; found: 299.2361.

4-*tert*-Butylbenzyl 3-(trityloxy)propyl ether (27)

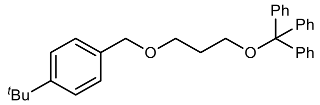

A solution of **3a** in CH_2Cl_2 [prepared from ligand **4a** (149.9 mg, 0.66 mmol) following GP-1, the preservation time of 10 min] was added to a suspension of nucleophile **9**[6] (95.5 mg, 0.30 mmol), pempidine (114 µL, 0.63 mmol), and molecular sieves 4A (125 mg) in 1,4-dioxane (1.00 mL) at room temperature. After 19 h, the reaction mixture was treated with saturated aqueous $NaHCO_3$ (162 µL). After 10 min, the reaction mixture was passed through a pad of diol-functionalized silica (EtOAc as an eluent). The eluent was concentrated under reduced pressure. The residue was purified by column chromatography (silica, hexane/EtOAc = 19:1) and recycling preparative HPLC ($CHCl_3$) to afford a white solid (130 mg, 93%). ^1H NMR (600 MHz, $CDCl_3$): δ 7.45–7.41 (m, 6H), 7.36–7.31 (m, 2H), 7.30–7.24 (m, 6H), 7.24–7.19 (m, 2H), 7.22–7.19 (m, 3H), 4.45 (s, 2H), 3.63 (t, J = 6.4 Hz, 2H), 3.19 (t, J = 6.3 Hz, 2H), 1.92 (tt, J_1 = 6.4 Hz, J_2 = 6.3 Hz, 2H), 1.31 (s, 9H); $^{13}C\{^1H\}$ NMR (150 MHz, $CDCl_3$): δ 150.6, 144.5, 135.7, 128.9, 127.9, 127.6, 127.0, 125.4, 86.5, 72.9, 67.7, 60.7, 34.6, 31.5, 30.6; HRMS (ESI): calcd for $C_{33}H_{36}NaO_2$ $[M + Na]^+$: 487.2613; found: 487.2610.

Acid-catalyzed alkylation of nucleophile 20 using alcohol 9 in the presence of triphenylmethyl ether S6 (Supplementary Figure 4)

Condition A: Following a reported procedure with modification,[26] a mixture of alcohol **9** (40.9 mg, 0.22 mmol), nucleophile **20** (80.0 µL, 0.44 mmol), triphenylmethyl ether **S6**[7] (67.1 mg, 0.22 mmol), and $InCl_3$ (2.4 mg, 0.011 mmol) in 1,2-dichloroethane (0.22 mL) was heated to 80 °C for 3 h. The reaction mixture was cooled to room temperature and diluted with Et_2O (10 mL). The mixture was washed with saturated aqueous $NaHCO_3$ and brine. The organic layer was dried ($MgSO_4$) and filtered. The filtrate was concentrated under reduced pressure. The yield of product **S7** (61%) was calculated by ^1H quantitative NMR spectroscopic analysis using *p*-nitrotoluene (30.8 mg) as the internal standard. Triphenylmethyl ether **S6** was not detected in the crude mixture.

Condition B: Following a reported procedure with modification,[27] a mixture of (pentafluorophenyl)boronic acid (1.1 mg, 0.005 mmol), oxalic acid (0.9 mg, 0.01 mmol), nucleophile **20** (36.0 µL, 0.20 mmol), and $MeNO_2$ (2.00 mL) was stirred for 5 min at room temperature. After adding alcohol **9** (18.3 mg, 0.10 mmol) and triphenylmethyl ether **S6**[7] (30.3 mg, 0.10 mmol), the reaction mixture was heated to 90 °C for 16 h. The reaction mixture was cooled to room temperature and diluted with CH_2Cl_2 (5 mL). The mixture was washed with saturated aqueous $NaHCO_3$ and brine. The organic layer was dried ($MgSO_4$) and filtered. The filtrate was concentrated under reduced pressure. The yield of product **S7** (59%) was calculated by ^1H quantitative NMR spectroscopic analysis using *p*-nitrotoluene (13.4 mg) as the internal standard. Triphenylmethyl ether **S6** was not detected in the crude mixture.

1,3-Bis[(4-*tert*-butylbenzyl)oxy]propane (28)

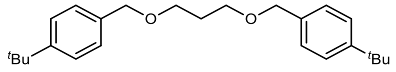

A solution of **3a** in CH_2Cl_2 [prepared from ligand **4a** (299.9 mg, 1.32 mmol) following GP-1, the preservation time of 10 min] was added to a suspension of nucleophile **13** (14.5 µL,

0.20 mmol), *N*-isobutylmorpholine (203 μL, 1.26 mmol), and molecular sieves 4A (250 mg) in 1,4-dioxane (2.00 mL) at room temperature. After 18 h, the reaction mixture was treated with saturated aqueous $NaHCO_3$ (216 μL). After 10 min, the reaction mixture was passed through a silica pad (EtOAc as an eluent). The eluent was concentrated under reduced pressure. The residue was purified by column chromatography (silica, hexane/EtOAc = 9:1) and preparative TLC (hexane/EtOAc = 9:1) to afford a white solid (59.7 mg, 81%). ^{1}H NMR (600 MHz, $CDCl_3$): δ 7.38–7.33 (m, 4H), 7.28–7.23 (m, 4H), 4.46 (s, 4H), 3.59 (t, J = 6.3 Hz, 4H), 3.59 (quin, J = 6.3 Hz, 2H), 1.31 (s, 18H); ^{13}C{^{1}H} NMR (150 MHz, $CDCl_3$): δ 150.6, 135.7, 127.7, 125.4, 73.0, 67.5, 34.7, 31.5, 30.4; HRMS (DART): calcd for $C_{25}H_{37}O_2$ [M + H]$^+$: 369.2794; found: 369.2803.

Methyl 3-(4-*tert*-butylphenyl)-2,2-dimethylpropanoate (29)

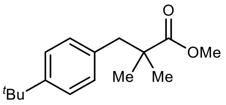

A solution of **3a** in CH_2Cl_2 [prepared from ligand **4a** (50.0 mg, 0.22 mmol) following GP-1, the preservation time of 10 min] was added to a suspension of nucleophile **14** (20.3 μL, 0.10 mmol), pempidine (37.9 μL, 0.21 mmol), and molecular sieves 4A (41.7 mg) in 1,4-dioxane (0.33 mL) at room temperature. After 4 h, the reaction mixture was passed through a silica pad (EtOAc as an eluent). The eluent was concentrated under reduced pressure. The residue was purified by silica gel column chromatography (hexane/EtOAc= 19:1) and preparative TLC (hexane/EtOAc = 19:1) to afford a white solid (19.8 mg, 80%). ^{1}H NMR (600 MHz, $CDCl_3$): δ 7.29–7.25 (m, 2H), 7.04–7.00 (m, 2H), 3.67 (s, 3H), 2.82 (s, 2H), 1.30 (s, 9H), 1.18 (s, 6H); ^{13}C{^{1}H} NMR (150 MHz, $CDCl_3$): δ 178.2, 149.3, 134.9, 129.9, 125.0, 51.8, 45.9, 43.8, 34.5, 31.5, 25.1; HRMS (DART): calcd for $C_{16}H_{25}O_2$ [M + H]$^+$: 249.1855; found: 249.1847.

3-(4-*tert*-Butylphenyl)-1-phenylpropan-1-one (30)

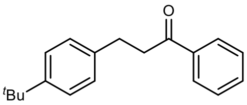

Synthesis from nucleophile ***15***: A solution of **3a** in CH_2Cl_2 [prepared from ligand **4a** (50.0 mg, 0.22 mmol) following GP-1, the preservation time of 10 min] was added to a suspension of nucleophile **15** (20.5 μL, 0.10 mmol), pempidine (37.9 μL, 0.21 mmol), and molecular sieves 4A (41.7 mg) in 1,4-dioxane (0.33 mL) at room temperature. After 15 h, the reaction mixture was passed through a silica pad (EtOAc as an eluent). The eluent was concentrated under reduced pressure. The residue was purified by silica gel column chromatography (hexane/EtOAc = 19:1) and preparative TLC (hexane/EtOAc = 19:1) to afford a white solid (14.2 mg, 53%).

Synthesis from nucleophile ***16***: A solution of **3a** in CH_2Cl_2 [prepared from ligand **4a** (50.0 mg, 0.22 mmol) following GP-1, the preservation time of 10 min] was added to a suspension of nucleophile **16**[8] (25.5 μL, 0.10 mmol), pempidine (37.9 μL, 0.21 mmol), and molecular sieves 4A (41.7 mg) in 1,4-dioxane (0.33 mL) at room temperature. After 6 h, the reaction mixture was quenched by the addition of MeOH (41 μL) and then passed through a silica pad (EtOAc as an eluent). The eluent was concentrated under reduced pressure. The yield of **24** (60%) was calculated by analyzing the ^{1}H quantitative NMR spectral profile of the crude mixture using coumarin as the internal standard.

^{1}H NMR (600 MHz, $CDCl_3$): δ 8.00–7.94 (m, 2H), 7.58–7.52 (m, 1H), 7.48–7.42 (m, 2H), 7.36–7.29 (m, 2H), 7.23–7.17 (m, 2H), 3.33–3.28 (m, 2H), 3.08–3.01 (m, 2H), 1.31 (s, 9H); ^{13}C{^{1}H} NMR (150 MHz, $CDCl_3$): δ 199.5, 149.1, 138.3, 137.0, 133.2, 128,7, 128.21,

128.18, 125.6, 40.6, 34.5, 31.5, 29.7; HRMS (DART): calcd for $C_{19}H_{23}O$ [M + H]$^+$: 267.1749; found: 267.1760.

3-(4-*tert*-Butylphenyl)-1-(4-chlorophenyl)propan-1-one (31)

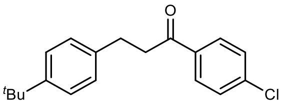

A solution of **3a** in CH_2Cl_2 [prepared from ligand **4a** (75.0 mg, 0.33 mmol) following GP-1, the preservation time of 10 min] was added to a suspension of nucleophile **17**[9] (22.7 mg, 0.10 mmol), pempidine (56.0 μL, 0.31 mmol), and molecular sieves 4A (62.5 mg) in 1,4-dioxane (0.50 mL) at room temperature. After 7 h, the reaction mixture was passed through a silica pad (EtOAc as an eluent). The eluent was concentrated under reduced pressure. The residue was purified by column chromatography (silica, hexane/EtOAc = 19:1), preparative TLC (hexane/EtOAc = 19:1), and column chromatography (carboxylic acid-functionalized silica, hexane) to afford a white solid (24.2 mg, 80%). ^{1}H NMR (600 MHz, $CDCl_3$): δ 7.92–7.87 (m, 2H), 7.44–7.39 (m, 2H), 7.35–7.30 (m, 2H), 7.21–7.16 (m, 2H), 3.30–3.22 (m, 2H), 3.07–2.99 (m, 2H), 1.31 (s, 9H); ^{13}C{^{1}H} NMR (150 MHz, $CDCl_3$): δ 198.3, 149.2, 139.6, 138.1, 135.4, 129.6, 129.1, 128.2, 125.6, 40.6, 34.5, 31.5, 29.7; HRMS (DART): calcd for $C_{19}H_{22}ClO$ [M + H]$^+$: 301.1359; found: 310.1350.

2-(4-Chlorophenyl)-1-(pyrrolidin-1-yl)ethan-1-one (18)[10]

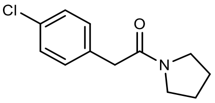

Following a standard procedure[11], a mixture of 2-(4-chlorophenyl)acetic acid (86.1 mg, 0.50 mmol), pyrrolidine (45.5 μL, 0.55 mmol), and DMT-MM (154 mg, 0.556 mmol) in MeOH (5.0 mL) was stirred for 3 h at room temperature. The reaction mixture was concentrated under reduced pressure. The residue was suspended in Et_2O, which was washed with aqueous HCl (1 M, 10 mL) and brine (30 mL). The organic layer was dried (Na_2SO_4) and filtered. The filtrate was concentrated under reduced pressure. The residue was purified by column chromatography (silica, hexane/EtOAc = 2:3) to afford a white solid (72.4 mg, 64%). ^{1}H NMR (600 MHz, $CDCl_3$): δ 7.31–7.25 (m, 2H), 7.24–7.19 (m, 2H), 3.61 (s, 2H), 3.48 (t, J = 6.8 Hz, 2H), 3.42 (t, J = 6.8 Hz, 2H), 1.93 (tt, J_1 = 6.8 Hz, J_2 = 6.8 Hz, 2H), 1.85 (tt, J_1 = 6.8 Hz, J_2 = 6.8 Hz, 2H); ^{13}C{^{1}H} NMR (150 MHz, $CDCl_3$): δ 169.1, 133.6, 132.8, 130.6, 128.8, 47.0, 46.1, 41.6, 26.3, 24.5; HRMS (DART): calcd for $C_{12}H_{15}ClNO$ [M + H]$^+$: 224.0842; found: 224.0841.

3-(4-*tert*-Butylphenyl)-2-(4-chlorophenyl)-1-(pyrrolidin-1-yl)propan-1-one (32)

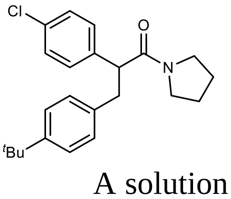

A solution of **3a** in CH_2Cl_2 [prepared from ligand **4a** (50.0 mg, 0.22 mmol) following GP-1, the preservation time of 10 min] was added to a suspension of nucleophile **18** (11.2 mg, 0.05 mmol), pempidine (37.9 μL, 0.21 mmol), and molecular sieves 4A (41.7 mg) in 1,4-dioxane (0.33 mL) at room temperature. After 5 h, the reaction mixture was treated with MeOH (41 μL) and then passed through a silica pad (EtOAc as an eluent). The eluent was concentrated under reduced pressure. The residue was purified by column chromatography (silica, hexane/EtOAc = 4:1) and preparative TLC (hexane/EtOAc = 4:1) to afford a white solid (14.3 mg, 77%). ^{1}H NMR (600 MHz, $CDCl_3$): δ 7.27–7.22 (m, 6H), 7.05–6.99 (m, 2H), 3.79 (dd, J_1 = 8.2 Hz, J_2 = 6.5 Hz, 1H), 3.49–3.34 (m, 3H), 3.21–3.10 (m, 2H), 2.89 (dd, J_1 = 13.7 Hz, J_2 =

6.5 Hz, 1H), 1.78–1.68 (m, 4H), 1.28 (s, 9H); $^{13}C\{^1H\}$ NMR (150 MHz, $CDCl_3$): δ 171.0, 149.2, 138.3, 136.7, 132.9, 129.8, 128.82, 128.80, 125.2, 52.4, 46.4, 46.1, 40.5, 34.5, 31.5, 26.0, 24.3; HRMS (DART): calcd for $C_{23}H_{29}ClNO$ [M + H]$^+$: 370.1938; found: 370.1941.

Benzhydryl 4-(*tert*-butyl-2,6-dimethyl)benzyl ether (33)

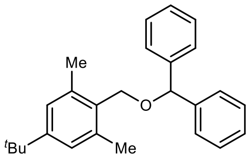

A solution of **3b** in CH_2Cl_2 [prepared from ligand **4b** (0.60 mmol) following GP-2, the preservation time of 1 h] was added to a suspension of nucleophile **9** (36.8 mg, 0.20 mmol), *N*-isobutylmorpholine (111 μL, 0.69 mmol), and molecular sieves 4A (150.0 mg) in 1,4-dioxane (1.00 mL) at room temperature. After 16 h, the reaction mixture was treated with H_2O (108 μL). After 10 min, the reaction mixture was passed through a silica pad (EtOAc as an eluent). The eluent was concentrated under reduced pressure. The residue was purified by column chromatography (silica, hexane/EtOAc = 9:1) and recycling preparative HPLC ($CHCl_3$) to afford a clear colorless oil (60.0 mg, 84%). ^1H NMR (600 MHz, $CDCl_3$): δ 7.39–7.35 (m, 4H), 7.35–7.29 (m, 4H), 7.27–7.23 (m, 2H), 7.03 (s, 2H), 5.44 (s, 1H), 4.50 (s, 2H), 2.31 (s, 6H), 1.29 (s, 9H); $^{13}C\{^1H\}$ NMR (150 MHz, $CDCl_3$): δ 150.9, 142.5, 137.8, 131.6, 128.4, 127.6, 127.4, 125.3, 83.4, 65.7, 34.4, 31.4, 20.1; HRMS (DART): calcd for $C_{26}H_{31}O$ [M + H]$^+$: 359.2375; found: 359.2367.

4-(*tert*-Butyl-2,6-dimethyl)benzyl 2-phenylpropan-2-yl ether (34)

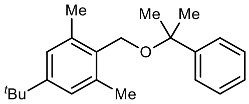

A solution of **3b** in CH_2Cl_2 [prepared from ligand **4b** (0.60 mmol) following GP-2, the preservation time of 1 h] was added to a suspension of nucleophile **10** (27.2 mg, 0.20 mmol), *N*-isobutylmorpholine (111 μL, 0.69 mmol), and molecular sieves 4A (150.0 mg) in 1,4-dioxane (1.00 mL) at room temperature. After 16 h, the reaction mixture was treated with H_2O (108 μL). After 10 min, the reaction mixture was passed through a silica pad (EtOAc as an eluent). The eluent was concentrated under reduced pressure. The residue was purified by column chromatography (silica, hexane/EtOAc = 9:1) and recycling preparative HPLC ($CHCl_3$) to afford a white solid (37.6 mg, 60%). ^1H NMR (600 MHz, $CDCl_3$): δ 7.55–7.51 (m, 2H), 7.41–7.36 (m, 2H), 7.31–7.26 (m, 1H), 7.01 (s, 2H), 4.14 (s, 2H), 2.31 (s, 6H), 1.66 (s, 6H), 1.24 (s, 9H); $^{13}C\{^1H\}$ NMR (150 MHz, $CDCl_3$): δ 150.5, 146.2, 137.6, 132.1, 128.3, 127.2, 126.2, 125.4, 76.4, 59.2, 34.4, 31.5, 28.3, 20.0; HRMS (DART): calcd for $C_{22}H_{34}NO$ [M + NH_4]$^+$: 328.2640; found: 328.2649.

1-Adamantyl 4-(*tert*-butyl-2,6-dimethyl)benzyl ether (35)

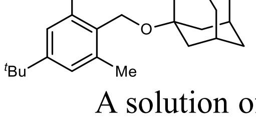

A solution of **3b** in CH_2Cl_2 [prepared from ligand **4b** (203 mg, 0.90 mmol) following GP-2, the preservation time of 1 h] was added to a suspension of nucleophile **11** (45.6 mg, 0.30 mmol), *N*-isobutylmorpholine (166.6 μL, 1.04 mmol), and molecular sieves 4A (225 mg) in 1,4-dioxane (1.50 mL) at room temperature. After 18 h, the reaction mixture was treated with H_2O (162 μL). After 10 min, the reaction mixture was passed through a silica pad (EtOAc as an eluent). The eluent was concentrated under reduced pressure. The residue was purified by column chromatography (silica, hexane/EtOAc = 19:1) and recycling preparative HPLC

($CHCl_3$) to afford a white solid (91.9 mg, 94%).^{1}H NMR (600 MHz, $CDCl_3$): δ 7.02 (s, 2H), 4.46 (s, 2H), 2.39 (s, 6H), 2.18 (br s, 3H), 1.92–1.86 (m, 6H), 1.70–1.62 (m, 6H), 1.27 (s, 9H); $^{13}C\{^1H\}$ NMR (150 MHz, $CDCl_3$): δ 150.4, 137.5, 132.2, 125.4, 72.3, 56.3, 41.7, 36.7, 34.4, 31.5, 30.8, 19.9; HRMS (DART): calcd for $C_{23}H_{34}NaO$ $[M + Na]^+$: 349.2507; found: 349.2500.

Methyl 3-(4-*tert*-butyl-2,6-dimethylphenyl)-2,2-dimethylpropanoate (36)

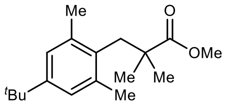

A solution of **3b** in CH_2Cl_2 [prepared from ligand **4b** (90.4 mg, 0.40 mmol) following GP-2, the preservation time of 1 h] was added to a suspension of nucleophile **14** (40.6 µL, 0.20 mmol), pempidine (83.0 µL, 0.46 mmol), and molecular sieves 4A (66.6 mg) in 1,4-dioxane (0.67 mL) at room temperature. After 20 h, the reaction mixture was treated with pyridine (48.4 µL) and then passed through a silica pad (EtOAc as an eluent). The eluent was concentrated under reduced pressure. The residue was purified by column chromatography (silica, hexane/EtOAc = 9:1) and preparative TLC (hexane/EtOAc = 9:1) to afford a white solid (40.9 mg, 74%). ^{1}H NMR (600 MHz, $CDCl_3$): δ 7.00 (s, 2H), 3.66 (s, 3H), 3.02 (s, 2H), 2.28 (s, 6H), 1.28 (s, 9H), 1.19 (s, 6H); $^{13}C\{^1H\}$ NMR (150 MHz, $CDCl_3$): δ 179.1, 148.7, 137.4, 132.5, 125.5, 52.0, 44.2, 38.0, 34.2, 31.5, 25.8, 21.6; HRMS (DART): calcd for $C_{18}H_{29}O_2$ $[M + H]^+$: 277.2168; found: 277.2173.

3-(4-*tert*-Butyl-2,6-dimethylphenyl)-1-phenylpropan-1-one (37)

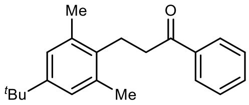

A solution of **3b** in CH_2Cl_2 [prepared from ligand **4b** (90.4 mg, 0.40 mmol) following GP-2, the preservation time of 1 h] was added to a suspension of nucleophile **15** (40.9 µL, 0.20 mmol), pempidine (83.0 µL, 0.46 mmol), and molecular sieves 4A (66.6 mg) in 1,4-dioxane (0.67 mL) at room temperature. After 20 h, the reaction mixture was treated with pyridine (48.4 µL) and then passed through a silica pad (EtOAc as an eluent). The eluent was concentrated under reduced pressure. The residue was purified by column chromatography (silica, hexane/EtOAc = 9:1) and preparative TLC (hexane/EtOAc = 19:1) to afford a white solid (45.1 mg, 77%). ^{1}H NMR (600 MHz, $CDCl_3$): δ 8.00–7.94 (m, 2H), 7.60–7.54 (m, 1H), 7.50–7.42 (m, 2H), 7.06 (s, 2H), 3.17–3.10 (m, 2H), 3.07–3.00 (m, 2H), 2.34, (s, 6H), 1.31 (s, 9H); $^{13}C\{^1H\}$ NMR (150 MHz, $CDCl_3$): δ 199.9, 148.9, 137.0, 135.9, 135.0, 133.2, 128.8, 128.2, 125.4, 38.0, 34.3, 31.5, 23.9, 20.3; HRMS (DART): calcd for $C_{21}H_{27}O$ $[M + H]^+$: 295.2062; found: 295.2071.

2-(4-*tert*-Butyl-2,6-dimethylbenzyl)cyclohexan-1-one (38)

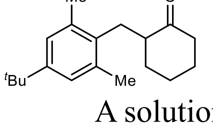

A solution of **3b** in CH_2Cl_2 [prepared from ligand **4b** (0.90 mmol) following GP-2, the preservation time of 1 h] was added to a suspension of nucleophile **19** (58.4 µL, 0.30 mmol), *N*-isobutylmorpholine (72.4 µL, 0.45 mmol), and molecular sieves 4A (225 mg) in 1,4-dioxane (1.50 mL) at room temperature. After 18 h, the reaction mixture was treated with H_2O (162 µL). After 10 min, the mixture was passed through a silica pad (EtOAc as an eluent). The eluent was concentrated under reduced pressure. The residue was purified by column chromatography (silica, hexane) and preparative TLC (hexane) to afford a clear colorless oil (69.2 mg, 83%). ^{1}H NMR (600 MHz, $CDCl_3$): δ 7.01 (s, 2H), 3.15 (dd, J_1 = 14.2 Hz, J_2 = 3.1 Hz, 1H), 2.60 (dd,

J_1 = 14.2 Hz, J_2 = 10.2 Hz, 1H), 2.57–2.48 (m, 1H), 2.46–2.43 (m, 1H), 2.34 (ddd, J_1 = 8.6 Hz, J_2 = 8.6 Hz, J_3 = 1.1 Hz, 1H), 2.27 (s, 6H), 2.11–2.04 (m, 1H), 2.01–1.94 (m, 1H), 1.85–1.77 (m, 1H), 1.69 (ddddd, J_1 = 13.0 Hz, J_2 = 13.0 Hz, J_3 = 13.0 Hz, J_4 = 4.1 Hz, J_5 = 4.1 Hz, 1H), 1.53 (ddddd, J_1 = 12.5 Hz, J_2 = 12.5 Hz, J_3 = 12.5 Hz, J_4 = 3.3 Hz, J_5 = 3.3 Hz, 1H), 1.44 (dddd, J_1 = 12.5 Hz, J_2 = 12.5 Hz, J_3 = 12.5 Hz, J_4 = 3.3 Hz, 1H), 1.29 (s, 9H); $^{13}C\{^1H\}$ NMR (150 MHz, $CDCl_3$): δ 213.1, 148.5, 136.4, 134.3, 125.4, 51.2, 42.3, 34.2, 33.6, 31.5, 28.22, 28.16, 25.6, 20.9; HRMS (DART): calcd for $C_{19}H_{29}O$ $[M + H]^+$: 273.2218; found: 273.2206.

2-(But-3-en-1-yl)-5-*tert*-butyl-1,3-dimethylbenzene (39)

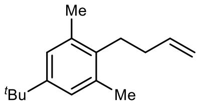

*Synthesis from nucleophile **20***: A solution of **3b** in CH_2Cl_2 [prepared from ligand **4b** (0.90 mmol) following GP-2, the preservation time of 1 h] was added to a suspension of nucleophile **20** (47.7 µL, 0.30 mmol), *N*-isobutylmorpholine (72.4 µL, 0.45 mmol), and molecular sieves 4A (225 mg) in 1,4-dioxane (1.50 mL) at room temperature. After 18 h, the reaction mixture was treated with H_2O (162 µL). After 10 min, the mixture was passed through a silica pad (EtOAc as an eluent). The eluent was concentrated under reduced pressure. The residue was purified by column chromatography (silica, hexane) and preparative TLC (hexane) to afford a clear colorless oil (55.6 mg, 86%).

*Synthesis from nucleophile **21***: A solution of **3b** in CH_2Cl_2 [prepared from ligand **4b** (0.90 mmol) following GP-2, the preservation time of 1 h] was added to a suspension of nucleophile **21** (93.0 µL, 0.30 mmol), *N*-isobutylmorpholine (72.4 µL, 0.45 mmol), and molecular sieves 4A (225 mg) in 1,4-dioxane (1.50 mL) at room temperature. After 20 h, the reaction mixture was treated with H_2O (162 µL). After 10 min, the mixture was passed through a silica pad (EtOAc as an eluent). The eluent was concentrated under reduced pressure. The residue was purified by column chromatography (silica, hexane) and preparative TLC (hexane) to afford a clear colorless oil (48.2 mg, 73%).

^1H NMR (600 MHz, $CDCl_3$): δ 7.02 (s, 2H), 5.98–5.88 (m, 1H), 5.12–5.06 (m, 1H), 5.02–4.98 (m, 1H), 2.70–2.65 (m, 2H), 2.32 (s, 6H), 2.24–2.17 (m, 2H), 1.29 (s, 9H); $^{13}C\{^1H\}$ NMR (150 MHz, $CDCl_3$): δ 148.4, 138.8, 135.8, 135.7, 125.2, 114.6, 34.2, 33.3, 31.5, 29.2, 20.3; HRMS (DART): calcd for $C_{16}H_{25}$ $[M + H]^+$: 217.1956; found: 217.1958.

5-*tert*-Butyl-1,3-dimethyl-2-(3-methylbut-3-en-1-yl)benzene (40)

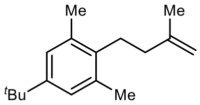

A solution of **3b** in CH_2Cl_2 [prepared from ligand **4b** (0.90 mmol) following GP-2, the preservation time of 1 h] was added to a suspension of nucleophile **22** (52 µL, 0.30 mmol), *N*-isobutylmorpholine (72.4 µL, 0.45 mmol), and molecular sieves 4A (225 mg) in 1,4-dioxane (1.50 mL) at room temperature. After 18 h, the reaction mixture was treated with H_2O (162 µL). After 10 min, the mixture was passed through a silica pad (EtOAc as an eluent). The eluent was concentrated under reduced pressure. The residue was purified by column chromatography (silica, hexane) to afford a clear colorless oil (44.1 mg, 64%). ^1H NMR (600 MHz, $CDCl_3$): δ 7.02 (s, 2H), 4.83–4.73 (m, 2H), 2.73–2.69 (m, 2H), 2.33 (s, 6H), 2.15–2.09 (m, 2H), 1.82 (s, 3H), 1.29 (s, 9H); $^{13}C\{^1H\}$ NMR (150 MHz, $CDCl_3$): δ 148.4, 146.4, 136.1, 135.6, 125.2, 109.8, 37.2, 34.3, 31.5, 28.4, 22.7, 20.1; HRMS (DART): calcd for $C_{17}H_{27}$ $[M + H]^+$: 231.2113; found: 231.2121.

5-*tert*-Butyl-2-(2,2-dimethylbut-3-en-1-yl)-1,3-dimethylbenzene (41)

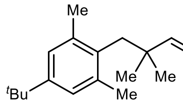

A solution of **3b** in CH_2Cl_2 [prepared from ligand **4b** (407 mg, 0.90 mmol) following GP-2, the preservation time of 1 h] was added to a suspension of nucleophile **23** (100.8 µL, 0.30 mmol), *N*-isobutylmorpholine (72.4 µL, 0.45 mmol), and molecular sieves 4A (225 mg) in 1,4-dioxane (1.50 mL) at room temperature. After 18 h, the reaction mixture was treated with H_2O (162 µL). After 10 min, the mixture was passed through a silica pad (EtOAc as an eluent). The eluent was concentrated under reduced pressure. The residue was purified by column chromatography (silica, hexane) to afford a clear colorless oil (67.8 mg, 92%). ^{1}H NMR (600 MHz, $CDCl_3$): δ 7.00 (s, 2H), 5.89 (dd, J_1 = 17.6 Hz, J_2 = 10.7 Hz 1H), 4.90 (dd, J_1 = 17.6 Hz, J_2 = 1.5 Hz, 1H), 4.84 (dd, J_1 = 10.7 Hz, J_2 = 1.5 Hz, 1H), 2.71 (s, 2H), 2.32 (s, 6H), 1.29 (s, 9H), 1.07 (s, 6H); ^{13}C{^{1}H} NMR (150 MHz, $CDCl_3$): δ 149.1, 148.2, 137.5, 133.3, 125.4, 109.6, 40.6, 39.9, 34.2, 31.5, 27.7, 22.2; HRMS (DART): calcd for $C_{18}H_{29}$ $[M + H]^+$: 245.2269; found: 245.2271.

(10-Acetoxy)decyl (4,4-dimethyl-1-phenylpent-2-yn-1-yl) ether (42)

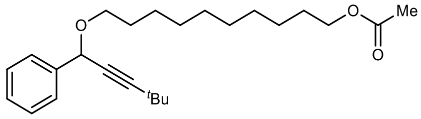

Tf_2O (65.6 µL, 0.40 mmol) was added dropwise to a suspension of ligand **4b** (90.5 mg, 0.40 mmol) and powdered molecular sieves 4A (80.1 mg) in CH_2Cl_2 (1.00 mL) at –78 °C. After 30 min, a solution of pempidine (86.7 µL, 0.48 mmol) and benzyl alcohol **5c**[12] (85.4 µL, 0.44 mmol) in CH_2Cl_2 (1.66 mL) was added dropwise at –78 °C. After 5 min, the reaction mixture was warmed to 0 °C and stirred for 1 h. The supernatant was added to a suspension of nucleophile **6** (21.6 mg, 0.10 mmol), 2,6-di-*tert*-butylpyridine (101.2 µL, 0.46 mmol), and powdered molecular sieves 4A (100.0 mg) in 1,4-dioxane (0.67 mL) at room temperature. After 3.5 h, the reaction mixture was treated with saturated aqueous $NaHCO_3$ (54 µL). After 10 min, the mixture was filtered, diluted with CH_2Cl_2 (15 mL), and washed with saturated aqueous $NaHCO_3$ (15 mL) and brine (40 mL). The organic layer was dried (Na_2SO_4) and filtered. The filtrate was concentrated under reduced pressure. The residue was purified by silica gel column chromatography (hexane/CH_2Cl_2 = 1:1) to afford a clear colorless oil (29.5 mg, 76%). ^{1}H NMR (600 MHz, $CDCl_3$) δ: 7.53–7.49 (m, 2H), 7.38–7.33 (m, 2H), 7.32–7.27 (m, 1H), 5.16 (s, 1H), 4.05 (t, J = 6.8 Hz, 2H), 3.63–3.58 (m, 1H), 3.50–3.44 (m, 1H), 2.05 (s, 3H), 1.66–1.57 (m, 4H), 1.40–1.25 (m, 21H). ^{13}C{^{1}H} NMR (100 MHz, $CDCl_3$) δ: 171.4, 139.7, 128.4, 128.1, 127.6, 96.6, 76.5, 71.6, 68.0, 64.8, 31.1, 29.8, 29.62, 29.59, 29.5, 29.4, 28.7, 27.7, 26.3, 26.0, 21.2. HRMS (DART): calcd for $C_{25}H_{39}O_3$ $[M + H]^+$: 387.2899; found: 387.2895.

^{1}H NMR monitoring of the attempted reaction between nucleophile 6 or 15 and benzyl iodide 43a (Figs. 4a,b and Supplementary Figs. 6a and 7a)

Benzyl iodide **43a**[13] (98.8 mg, 0.36 mmol), pempidine (68.3 µL, 0.38 mmol), and 1,3,5-trimethoxybenzene (30 mg, 0.18 mmol, used as an internal standard) were dissolved in $CDCl_3$ (1.80 mL). Nucleophile **6** (12.9 mg, 0.060 mmol) or **15** (12.3 µL, 0.060 mmol) in an NMR tube was dissolved in an aliquot (0.60 mL) of this $CDCl_3$ solution. The temperature of the reaction mixture was maintained at 25 °C. ^{1}H NMR measurements (600 MHz) were performed at intervals to monitor the reaction.

^{1}H NMR monitoring of the attempted reaction between nucleophile 6 and benzyl iodide 43b (Fig. 4a and Supplementary Fig. 6b)

In an NMR tube, nucleophile **6** (12.9 mg, 0.060 mmol), benzyl iodide **43b**[3] (36.3 mg,

0.12 mmol), pempidine (24.9 μL, 0.14 mmol), and 1,3,5-trimethoxybenzene (10 mg, 0.059 mmol, used as an internal standard) were dissolved in $CDCl_3$ (0.60 mL). The temperature of the reaction mixture was maintained at 25 °C. ^{1}H NMR measurements (600 MHz) were performed at intervals to monitor the reaction.

^{1}H NMR monitoring of the attempted reaction between nucleophile 15 and benzyl iodide 43b (Fig. 4b and Supplementary Fig. 7b)

Benzyl iodide **43b**[3] (108 mg, 0.36 mmol), pempidine (74.7 μL, 0.41 mmol), and 1,3,5-trimethoxybenzene (30 mg, 0.18 mmol, used as an internal standard) were dissolved in $CDCl_3$ (1.80 mL). Nucleophile **15** (12.3 μL, 0.060 mmol) in an NMR tube was dissolved in an aliquot (0.60 mL) of this $CDCl_3$ solution. The temperature of the reaction mixture was maintained at 25 °C. ^{1}H NMR measurements (600 MHz) were performed at intervals to monitor the reaction.

Bis(3,5-di-*tert*-butylbenzyl) ether (8d)

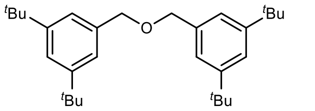

Following a standard procedure,[29] a mixture of (pentafluorophenyl)boronic acid (4.2 mg, 0.02 mmol), oxalic acid (3.6. mg, 0.04 mmol), and alcohol **5d** (88.1 mg, 0.40 mmol) was heated to 90 °C for 17 h and then cooled to room temperature. The reaction mixture was diluted with toluene (2 mL) and concentrated under reduced pressure. The residue was purified by column chromatography (silica, hexane/EtOAc =7:3) and preparative TLC (hexane/EtOAc = 7:3) to afford a clear colorless oil (35.3 mg, 42%). ^{1}H NMR (600 MHz, $CDCl_3$): δ 7.36 (s, 2H), 7.23 (s, 4H), 4.56 (s, 4H), 1.33 (s, 36H); $^{13}C\{^1H\}$ NMR (150 MHz, $CDCl_3$): δ 150.9, 137.7, 122.2, 121.8, 72.9, 35.0, 31.6; HRMS (DART): calcd for $C_{30}H_{46}NaO$ $[M + Na]^+$: 445.3446; found: 445.3459.

***O*-(4-*tert*-Butyl)benzylation of alcohol 5d using carbocationoid 3a (Fig. 4c)**

Tf_2O (49.2 μL, 0.30 mmol) was added dropwise to a suspension of ligand **4a** (75.0 mg, 0.33 mmol), benzyl alcohol **5a** (50.8 μL, 0.30 mmol), pempidine (65.0 μL, 0.36 mmol), and powdered molecular sieves 4A (37.5 mg) in CH_2Cl_2 (1.50 mL) at –78 °C. After 10 min, the reaction mixture was warmed to 0 °C and stirred for 10 min. The supernatant was added to a suspension of alcohol **5d** (44.1 mg, 0.20 mmol), 2,6-di-*tert*-butylpyridine (57.2 μL, 0.26 mmol), and powdered molecular sieves 4A (46.9 mg) in 1,4-dioxane (0.38 mL) at room temperature. After 8 h, the reaction mixture was treated with saturated aqueous $NaHCO_3$ (108 μL). After 10 min, the mixture was filtered, diluted with CH_2Cl_2, and washed with saturated aqueous $NaHCO_3$ and brine. The organic layer was dried (Na_2SO_4) and filtered. The filtrate was concentrated under reduced pressure. The ratio of **8a**:**8ad**:**8d** (5:95:<1) was calculated by ^{1}H NMR spectroscopic analysis of the crude mixture. The residue was purified by column chromatography (silica, hexane/EtOAc = 19:1) and recycling preparative HPLC ($CHCl_3$) to afford **8ad** as a clear colorless oil (60.5 mg, 82%). ^{1}H NMR (600 MHz, $CDCl_3$): δ 7.40–7.37 (m, 2H), 7.35 (t, J = 1.9 Hz, 1H), 7.33–7.29 (m, 2H), 7.20 (d, J = 1.9 Hz, 2H), 4.55 (s, 2H), 4.54 (s, 2H), 1.33 (s, 18H), 1.32 (s, 9H); $^{13}C\{^1H\}$ NMR (150 MHz, $CDCl_3$): δ 150.9, 150.7, 137.6, 135.6, 127.9, 125.5, 122.3, 121.8, 72.9, 72.1, 35.0, 34.7, 31.6, 31.5; HRMS (DART): calcd for $C_{26}H_{38}NaO$ $[M + Na]^+$: 389.2820; found: 389.2838.

***O*-(3,5-Di-*tert*-butyl)benzylation of alcohol 5a using carbocationoid 3d (Fig. 4c)**

Tf_2O (49.2 μL, 0.30 mmol) was added dropwise to a suspension of ligand **4a** (75.0 mg, 0.33 mmol), benzyl alcohol **5d** (66.1 mg, 0.30 mmol), pempidine (65.0 μL, 0.36 mmol), and powdered molecular sieves 4A (37.5 mg) in CH_2Cl_2 (1.50 mL) at –78 °C. After 10 min, the

reaction mixture was warmed to 0 °C and stirred for 10 min. The supernatant was added to a suspension of alcohol **5a** (33.9 μL, 0.20 mmol), 2,6-di-*tert*-butylpyridine (57.2 μL, 0.26 mmol), and powdered molecular sieves 4A (46.9 mg) in 1,4-dioxane (0.38 mL) at room temperature. After 9 h, the reaction mixture was treated with saturated aqueous $NaHCO_3$ (108 μL). After 10 min, the mixture was filtered, diluted with CH_2Cl_2, and washed with saturated aqueous $NaHCO_3$ and brine. The organic layer was dried (Na_2SO_4) and filtered. The filtrate was concentrated under reduced pressure. The ratio of **8a**:**8ad**:**8d** (<1:92:8) was calculated by ^1H NMR spectroscopic analysis of the crude mixture. The residue was purified by column chromatography (silica, hexane/EtOAc = 19:1) and recycling preparative HPLC ($CHCl_3$) to afford a mixture composed of **8ad** (58.2 mg, 80%) and **8d** (6.6 mg). The product ratio was determined by ^1H NMR spectroscopic analysis.

Acid-catalyzed etherification between alcohols 5a and 5d (Fig. 4d)

*Reaction using **5d** (1 equiv.)*: Following a standard procedure,[29] a mixture of (pentafluorophenyl)boronic acid (4.2 mg, 0.02 mmol), oxalic acid (3.6 mg, 0.04 mmol), $MeNO_2$ (4.00 mL) was stirred for 5 min at room temperature. After adding alcohols **5a** (101.6 μL, 0.60 mmol) and **5d** (88.1 mg, 0.40 mmol), the reaction mixture was heated to 90 °C for 10 h and then cooled to room temperature. The reaction mixture was diluted with toluene (4 mL) and concentrated under reduced pressure. The ratio of **8a**:**8ad**:**8d** (29:50:21) and the yield of **8ad** (25%) was calculated by ^1H quantitative NMR spectroscopic analysis of the crude mixture using coumarin as the internal standard.

*Reaction using **5a** (1 equiv.)*: Following a standard procedure,[29] a mixture of (pentafluorophenyl)boronic acid (4.2 mg, 0.02 mmol), oxalic acid (3.6 mg, 0.040 mmol), $MeNO_2$ (4.00 mL) was stirred for 5 min at room temperature. After adding alcohols **5a** (67.7 μL, 0.40 mmol) and **5d** (132.2 mg, 0.60 mmol), the reaction mixture was heated to 90 °C for 10 h and then cooled to room temperature. The reaction mixture was diluted with toluene (4 mL) and concentrated under reduced pressure. The ratio of **8a**:**8ad**:**8d** (44:44:12) and the yield of **8ad** (32%) was calculated by ^1H quantitative NMR spectroscopic analysis of the crude mixture using coumarin as the internal standard.

1-(4-*tert*-Butyl-2,6-dimethyl)benzyl-2,2,6,6-tetramethylpiperidine (45)

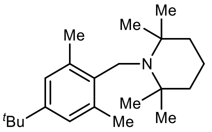

A solution of **3b** in CH_2Cl_2 [prepared from ligand **4b** (271 mg, 1.20 mmol) following GP-2, the preservation time of 1 h] was added to a suspension of amine **44** (51.1 μL, 0.30 mmol), pempidine (249 μL, 1.38 mmol), and molecular sieves 4A (300 mg) in 1,4-dioxane (2.00 mL). The reaction mixture was stirred for 25 h at room temperature and for 7 h at 35 °C. The reaction mixture was treated with saturated aqueous $NaHCO_3$ (162 μL). After 10 min, the mixture was passed through a pad of amine-functionalized silica (CH_2Cl_2 as an eluent). The eluent was concentrated under reduced pressure. The residue was purified by column chromatography [twice, diol-functionalized silica (hexane) for the first chromatography, reverse phase silica (MeOH) for the second chromatography) and recycling preparative HPLC ($CHCl_3$) to afford a pale yellow solid (60.3 mg, 64%). ^1H NMR (600 MHz, $CDCl_3$): δ 6.89 (s, 2H), 3.90 (s, 2H), 2.56 (s, 6H), 1.59–1.51 (m, 2H), 1.51–1.45 (m, 4H), 1.27 (s, 9H), 1.01 (s, 12H); $^{13}C\{^1H\}$ NMR (150 MHz, $CDCl_3$): δ 148.1, 137.5, 136.1, 126.1, 55.8, 44.9, 42.3, 34.1, 31.5, 27.5, 21.9, 18.2; HRMS (DART): calcd for $C_{22}H_{38}N$ $[M + H]^+$: 316.3004; found: 316.2992.

(4-*tert*-Butyl-2,6-dimethyl)benzyl 2,2,2-trichloroacetimidate (46)

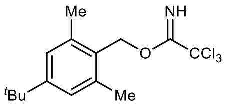

Following a standard procedure[14], DBU (34.3 μL, 0.23 mmol) was added to a solution of benzyl alcohol **5b** (442.5 mg, 2.30 mmol) and 2,2,2-trichloroacetonitrile (264.3 μL, 2.53 mmol) in CH_2Cl_2 (5.75 mL) at 0 °C. After 2 h, the reaction mixture was diluted with CH_2Cl_2 (10 mL) and then washed with saturated aqueous NH_4Cl (10 mL) and brine (25 mL). The organic layer was dried (Na_2SO_4) and filtered. The filtrate was concentrated under reduced pressure. The residue was purified by column chromatography (diol-functionalized silica, hexane) to afford a clear colorless oil (771.6 mg, quant.). ^{1}H NMR (600 MHz, $CDCl_3$): δ 8.36 (s, 1H), 7.08 (s, 2H), 5.33 (s, 2H), 2.42 (s, 6H), 1.31 (s, 9H); ^{13}C{^{1}H} NMR (150 MHz, $CDCl_3$): δ 163.2, 151.8, 138.2, 128.6, 125.3, 91.6, 66.3, 34.4, 31.3, 19.9; HRMS (DART): calcd for $C_{15}H_{21}Cl_3NO$ $[M + H]^+$: 336.0689; found: 336.0689.

Attempted *N*-alkylation of amine 44 using trichloroacetimidate 46.

$MeNO_2$ was used as a co-solvent to dissolve the salt of amine **44** and TfOH in CH_2Cl_2/1,4-dioxane. The CH_2Cl_2/1,4-dioxane/$MeNO_2$ (1:1:1) solvent system is henceforth referred to as Solvent A.

Reaction using TfOH (0.2 equiv.): A solution of TfOH in Solvent A (0.10 M, 0.20 mL, 0.020 mmol) and a solution of trichloroacetimidate **37** (67.3 mg, 0.20 mmol) in Solvent A (0.40 mL) were added to a solution of amine **44** (17.0 μL, 0.10 mmol) in Solvent A (0.60 mL) at room temperature. The reaction mixture was heated at 35 °C for 24 h. The reaction mixture was quenched by the addition of NEt_3 (13.9 μL) and then passed through a pad of amine-functionalized silica (EtOAc as an eluent). The eluent was concentrated under reduced pressure. The alkylated product **45** was not detected by ^{1}H NMR spectroscopic analysis of the crude mixture.

Reaction using TfOH (2.0 equiv.): A solution of amine **44** (17.0 μL, 0.10 mmol), trichloroacetimidate **46** (67.3 mg, 0.20 mmol), and TfOH (17.7 μL, 0.20 mmol) in Solvent A (3.90 mL) was heated at 35 °C for 24 h. The reaction mixture was quenched by the addition of NEt_3 (30.7 μL, 0.22 mmol) and then passed through a pad of amine-functionalized silica (EtOAc as an eluent). The eluent was concentrated under reduced pressure. The alkylated product **45** was not detected by ^{1}H NMR spectroscopic analysis of the crude mixture.

4. Supplementary References

33. Tosato, M. L. & Paoloni, L. The N,N,O-trimethyl ester of cyanuric acid. *Ric. Sci.* **37**, 259 (1967).
34. Li, X.-J., Zhang, J.-L., Geng, Y. & Jin, Z. Nickel-Catalyzed Suzuki–Miyaura Coupling of Heteroaryl Ethers with Arylboronic Acids. *J. Org. Chem.* **78**, 5078–5084 (2013). doi: 10.1021/jo4005537
35. Tashiro, M. & Yamato, T. Studies on Selective Preparation of Aromatic Compounds. Part 16. A Convenient Preparation of 1,2-Di- and 1,2,3-Tri-substituted Benzenes using the t-Butyl Function as a Positional Protective Group. *J. Chem. Soc. Perkin Trans. 1* 176 (1979). doi: 10.1039/p19790000176
36. Tashiro, M. & Yamato, T. Metacyclophanes and Related Compounds. 1. Preparation and Nuclear Magnetic Resonance Spectra of 8,16-Disubstituted [2.2]Metacyclophanes. *J. Org. Chem.* **46**, 4556–4562 (1981). doi: 10.1021/jo00335a047
37. Felstead, R., Gibson, S. E., Rooney, A. & Tse, E. S. Y. A Stereocontrolled Approach to Ethers with Two α Stereocentres. *Eur. J. Org. Chem.* 4963–4971 (2008). doi: 10.1002/ejoc.200800634
38. Reddy, C. R., Rajesh, G., Balaji, S. V. & Chethan, N. Tris(pentafluorophenyl)borane: a mild and efficient catalyst for the chemoselective tritylation of alcohols. *Tetrahedron Lett.* **49**, 970–973 (2008). doi: 10.1016/j.tetlet.2007.12.020
39. Maltese, M., Vergari, M. C. & Donzello, M. P. Zinc chloride homogeneous catalysis in the tritylation of hydroxyl- and amide-bearing molecules. *Tetrahedron Lett.* **52**, 483–487 (2011). doi: 10.1016/j.tetlet.2010.11.095
40. de Nanteuil, F. & Waser, J. Synthesis of Aminocyclobutanes by Iron-Catalyzed [2+2] Cycloaddition. *Angew. Chem. Int. Ed.* **52**, 9009–9013 (2013). doi: 10.1002/anie.201303803
41. Wei, S. & Du, H. A Highly Enantioselective Hydrogenation of Silyl Enol Ethers Catalyzed by Chiral Frustrated Lewis Pairs. *J. Am. Chem. Soc.* **136**, 12261–12264 (2014). doi: 10.1021/ja507536n
42. Huh, D. H., Jeong, J. S., Lee, H. B., Ryu, H. & Kim, Y. G. An efficient method for one-carbon elongation of aryl aldehydes via their dibromoalkene derivatives. *Tetrahedron* **58**, 9925–9932 (2002). doi: 10.1016/S0040-4020(02)01324-8
43. Kunishima, M., Kawachi, C., Hioki, K., Terao, K. & Tani, S. Formation of carboxamides by direct condensation of carboxylic acids and amines in *alcohols* using a new alcohol- and water-soluble condensing agent: DMT-MM. *Tetrahedron*. **57**, 1551–1558 (2001).
44. Ramón, R. S. *et al.* Gold-Catalyzed Meyer−Schuster Rearrangement: Application to the Synthesis of Prostaglandins. *Organometallics* **29**, 3665–3668 (2010). doi: 10.1016/j.tetlet.2010.11.095
45. Weidmann, N., Harenberg, J. H. & Knochel, P. Continuous Flow Preparation of (Hetero)benzylic Lithiums via Iodine–Lithium Exchange Reaction under Barbier Conditions. *Org. Lett.* **22**, 5895–5899 (2020). doi: 10.1021/acs.orglett.0c01991
46. Ikeuchi, K., Murasawa, K. & Yamada, H. A Simple Method for the Preparation of Stainless and Highly Pure Trichloroacetimidates. *Synlett* **30**, 1308–1312 (2019). doi: 10.1055/s-0037-1611551
