# Supplementary material for: Carbocationoids, a concept for controlling highly reactive cationic species
Source: Commun Chem. 2024 Mar 13;7:55. doi: 10.1038/s42004-024-01139-w (PMC10937719; doi:10.1038/s42004-024-01139-w)
Supplement: Supplementary file 2 — Description of Additional Supplementary Files [file 42004_2024_1139_MOESM2_ESM.pdf]

Description of Additional Supplementary Files

File name: Supplementary Data 1

Description: NMR spectral data
