# Supplementary material for: Carbocationoids, a concept for controlling highly reactive cationic species
Source: Commun Chem. 2024 Mar 13;7:55. doi: 10.1038/s42004-024-01139-w (PMC10937719; doi:10.1038/s42004-024-01139-w)
Supplement: Supplementary file 3 — Supplementary Data [file 42004_2024_1139_MOESM3_ESM.pdf]

Supplementary Data 1

<u>^{1}H and ^{13}C{^{1}H} NMR spectra</u>

2,4-Dimethoxy-6-(neopentyloxy)-1,3,5-triazine (S1)

^{1}H NMR ($CDCl_3$, 600 MHz)

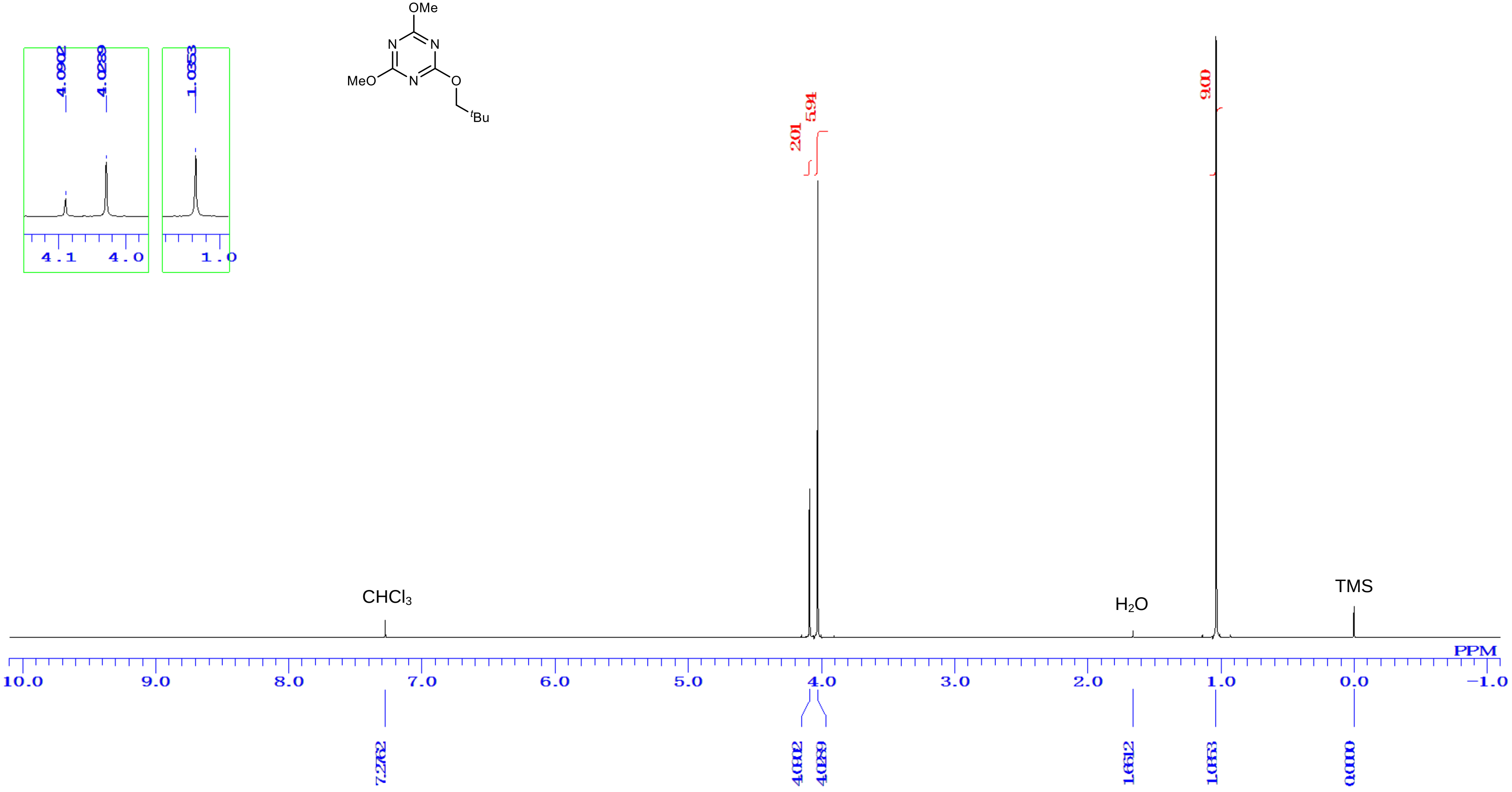

2,4-Dimethoxy-6-(neopentyloxy)-1,3,5-triazine (S1)

$^{13}C\{^{1}H\}$ NMR ($CDCl_3$, 150 MHz)

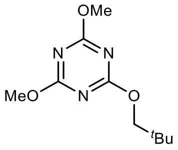

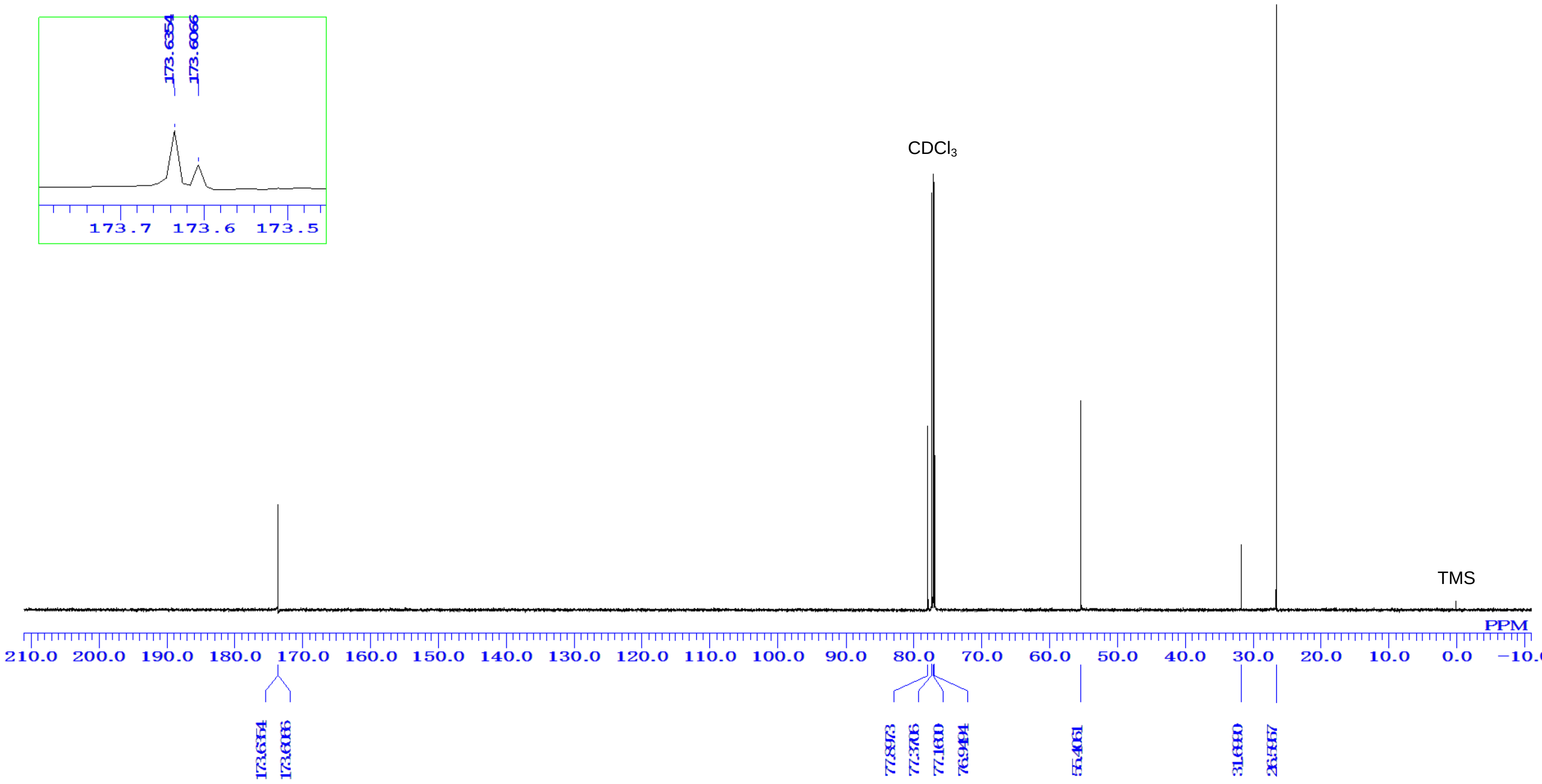

1,3-Dimethyl-6-(neopentyloxy)-1,3,5-triazine-2,4(1*H*,3*H*)-dione (4a)

^{1}H NMR ($CDCl_3$, 600 MHz)

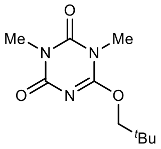

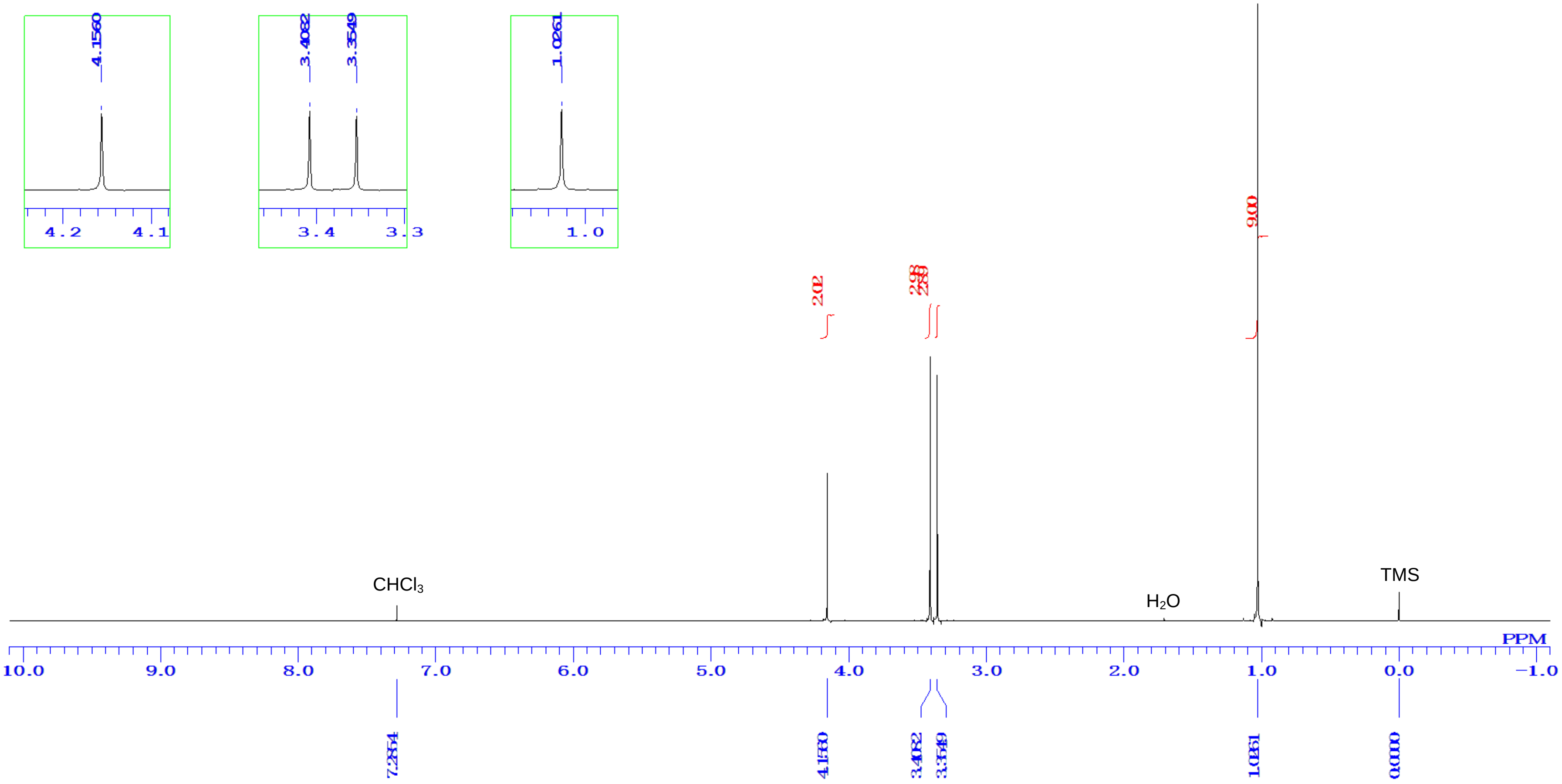

1,3-Dimethyl-6-(neopentyloxy)-1,3,5-triazine-2,4(1*H*,3*H*)-dione (4a)
$^{13}C\{^1H\}$ NMR ($CDCl_3$, 150 MHz)

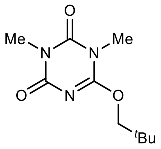

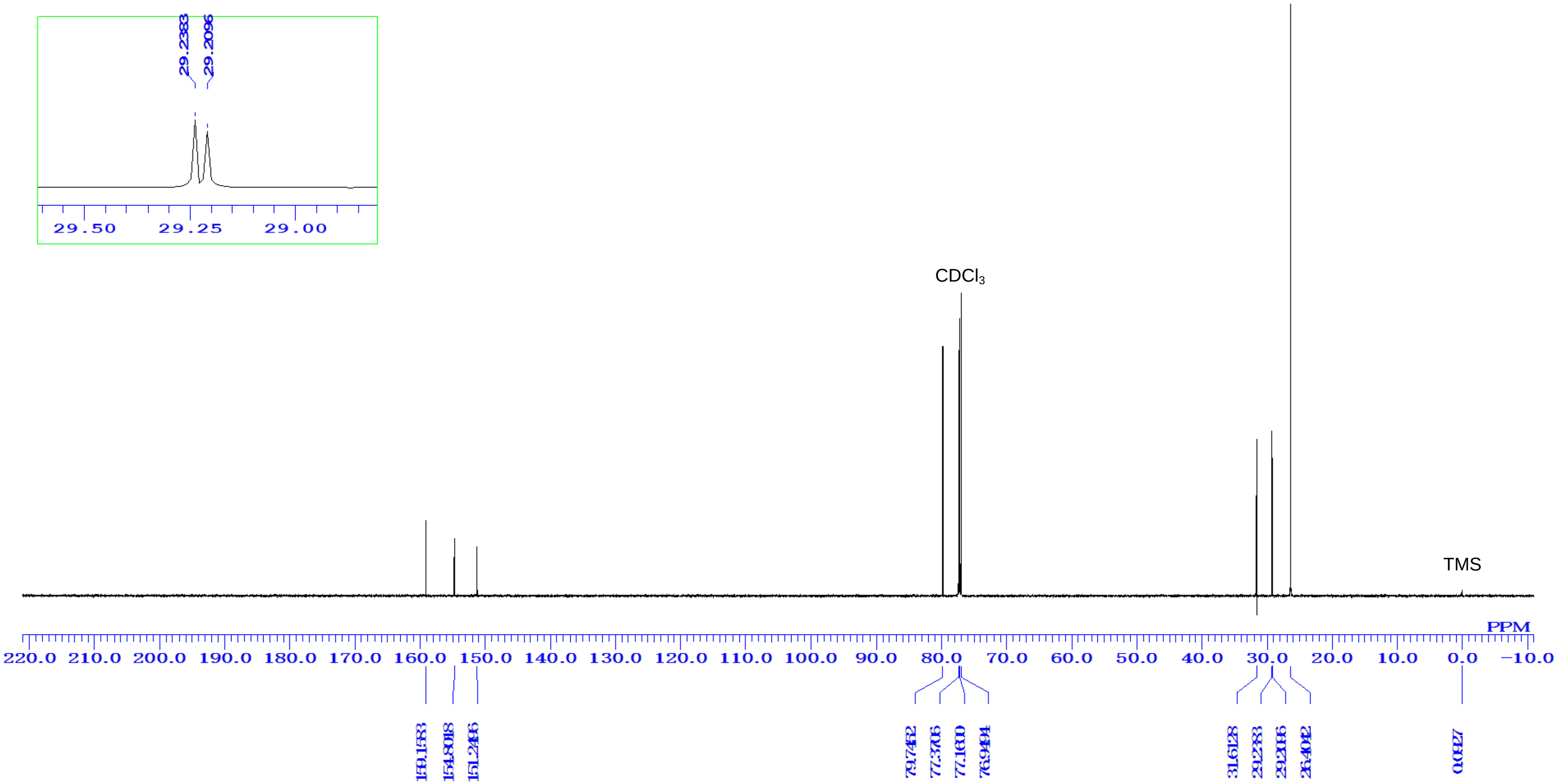

2,4-Dimethoxy-6-phenoxy-1,3,5-triazine (S2)

^{1}H NMR ($CDCl_3$, 400 MHz)

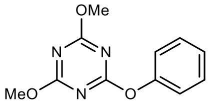

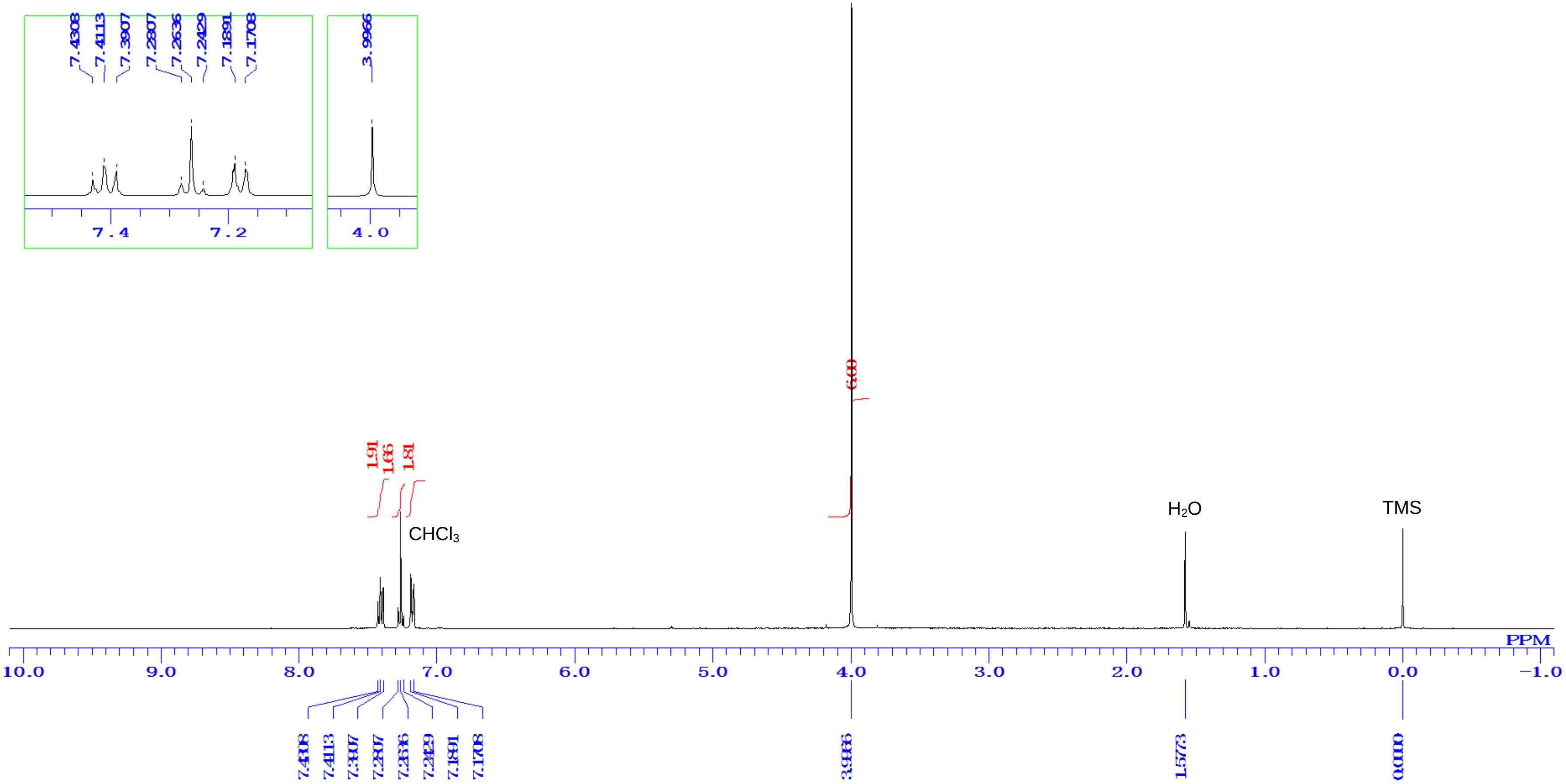

2,4-Dimethoxy-6-phenoxy-1,3,5-triazine (S2)

$^{13}C\{^{1}H\}$ NMR ($CDCl_3$, 150 MHz)

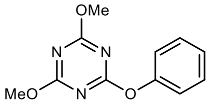

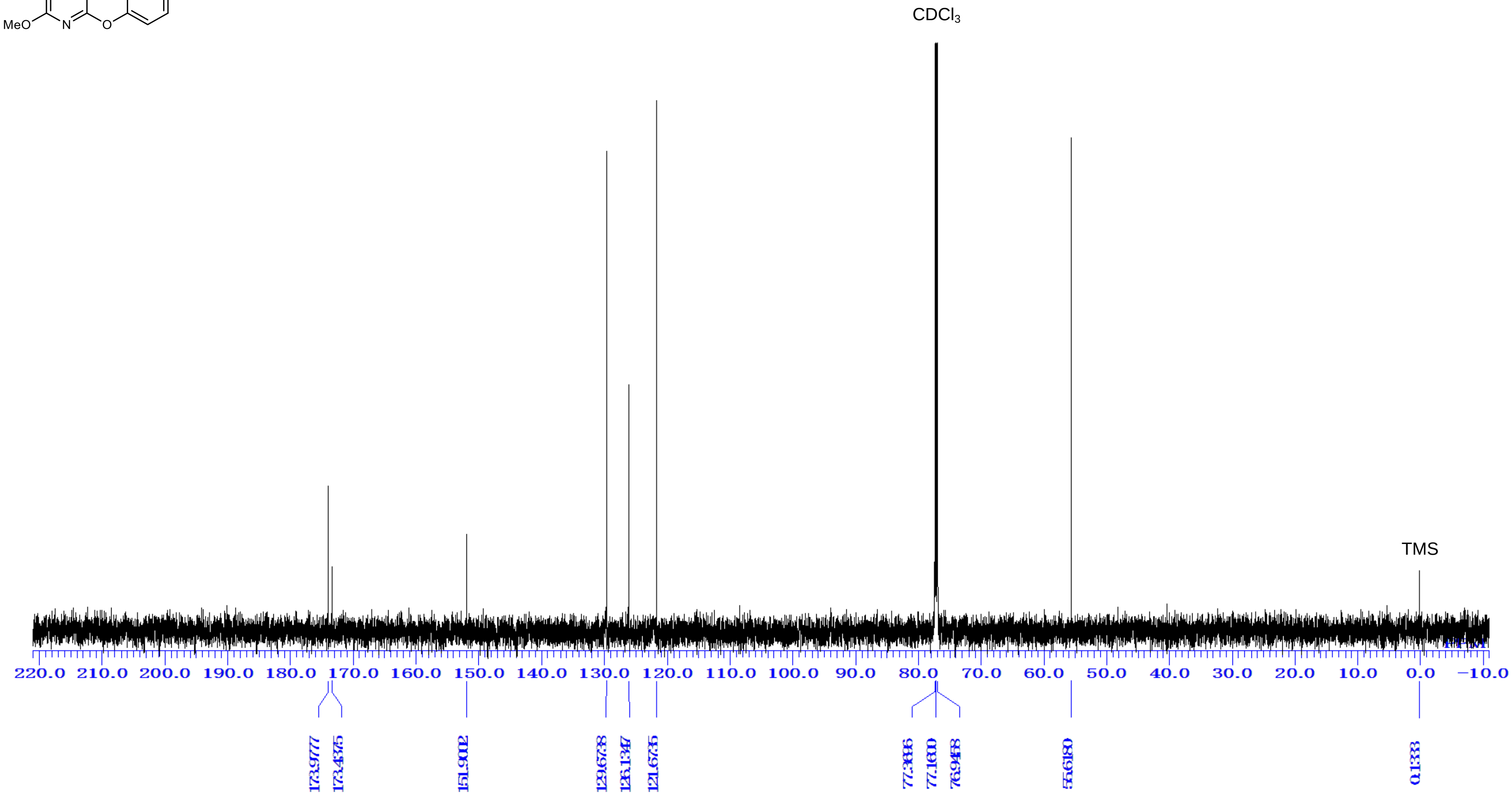

1,3-Dimethyl-6-phenoxy-1,3,5-triazine-2,4(1*H*,3*H*)-dione (S3)

^{1}H NMR ($CDCl_3$, 600 MHz)

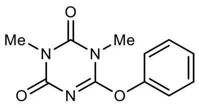

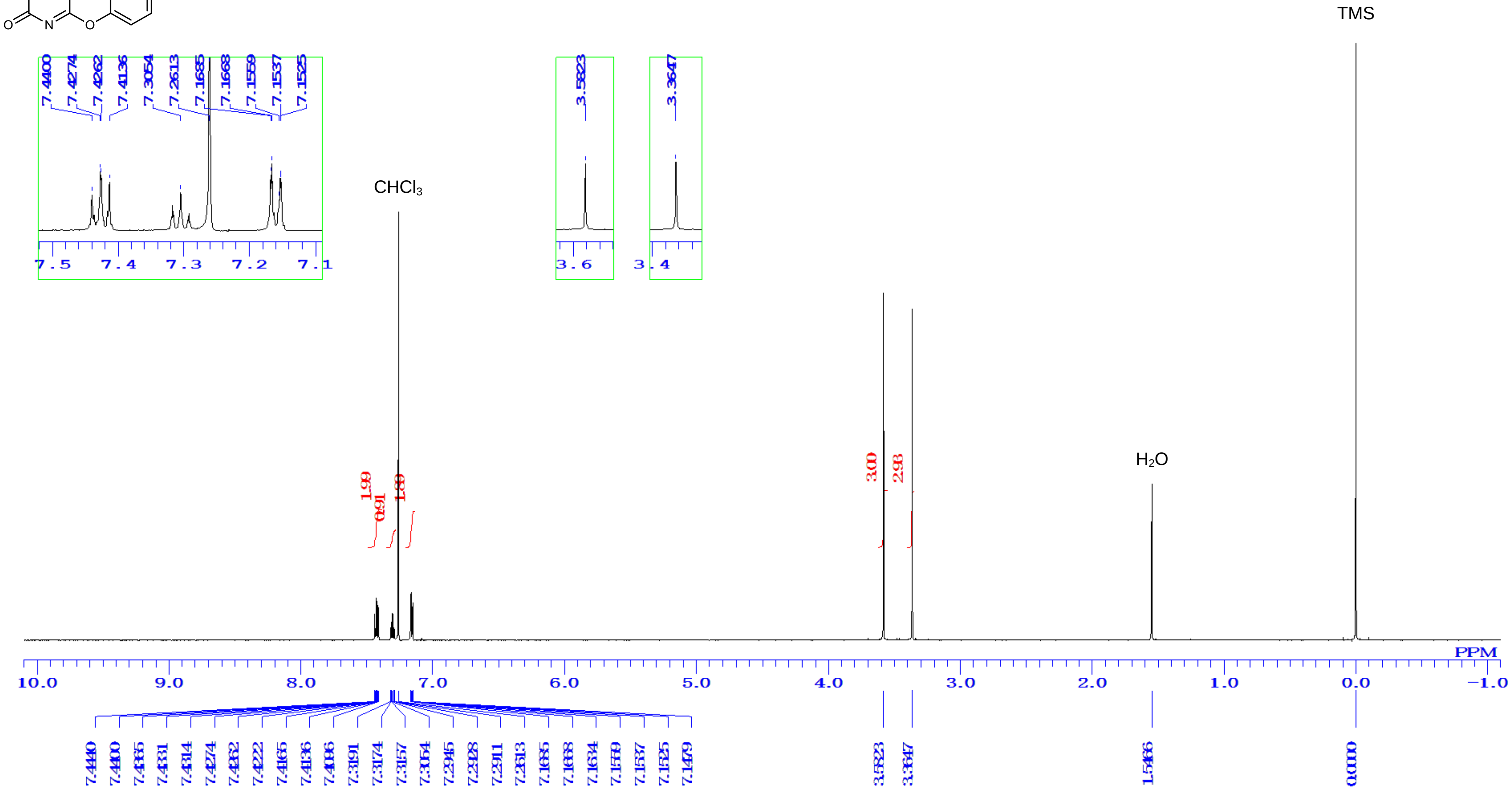

1,3-Dimethyl-6-phenoxy-1,3,5-triazine-2,4(1*H*,3*H*)-dione (S3)

$^{13}C\{^1H\}$ NMR ($CDCl_3$, 150 MHz)

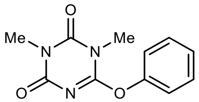

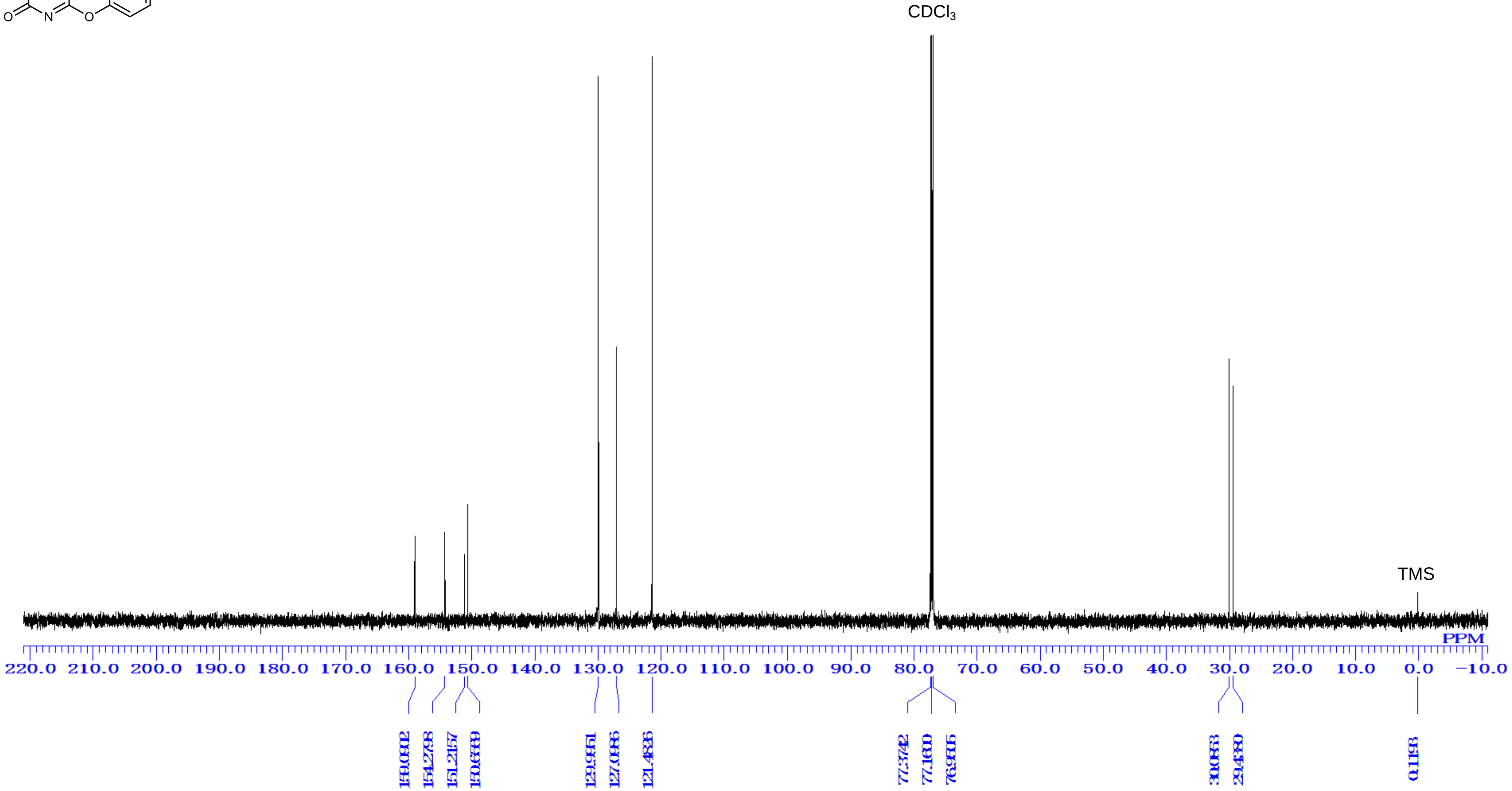

1,3-Dimethyl-6-morpholino-1,3,5-triazine-2,4(1*H*,3*H*)-dione (4b)
^{1}H NMR ($CDCl_3$, 600 MHz)

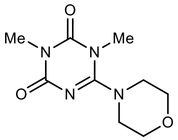

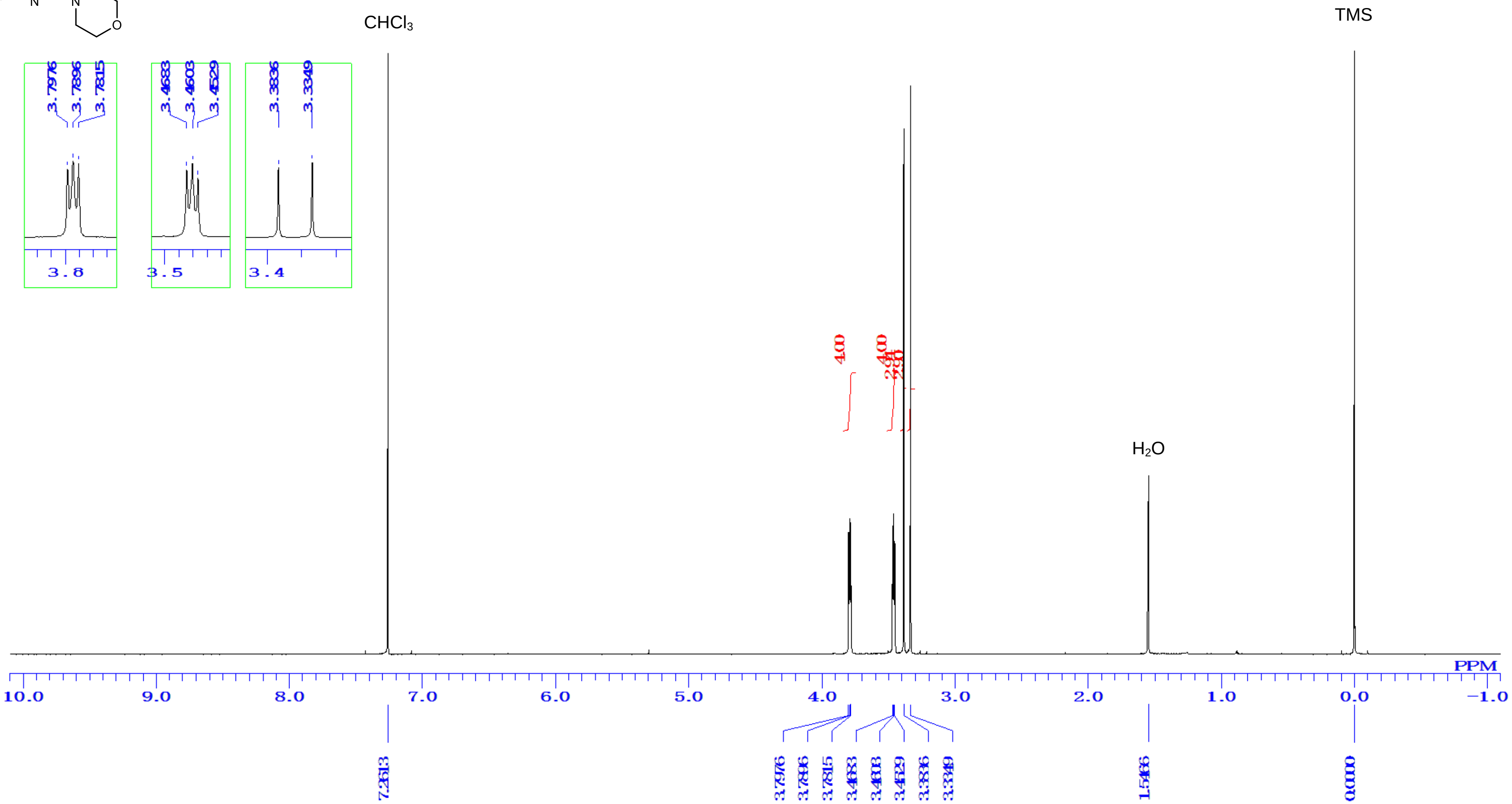

1,3-Dimethyl-6-morpholino-1,3,5-triazine-2,4(1*H*,3*H*)-dione (4b)

$^{13}C\{^{1}H\}$ NMR ($CDCl_3$, 150 MHz)

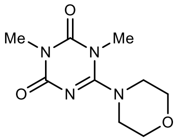

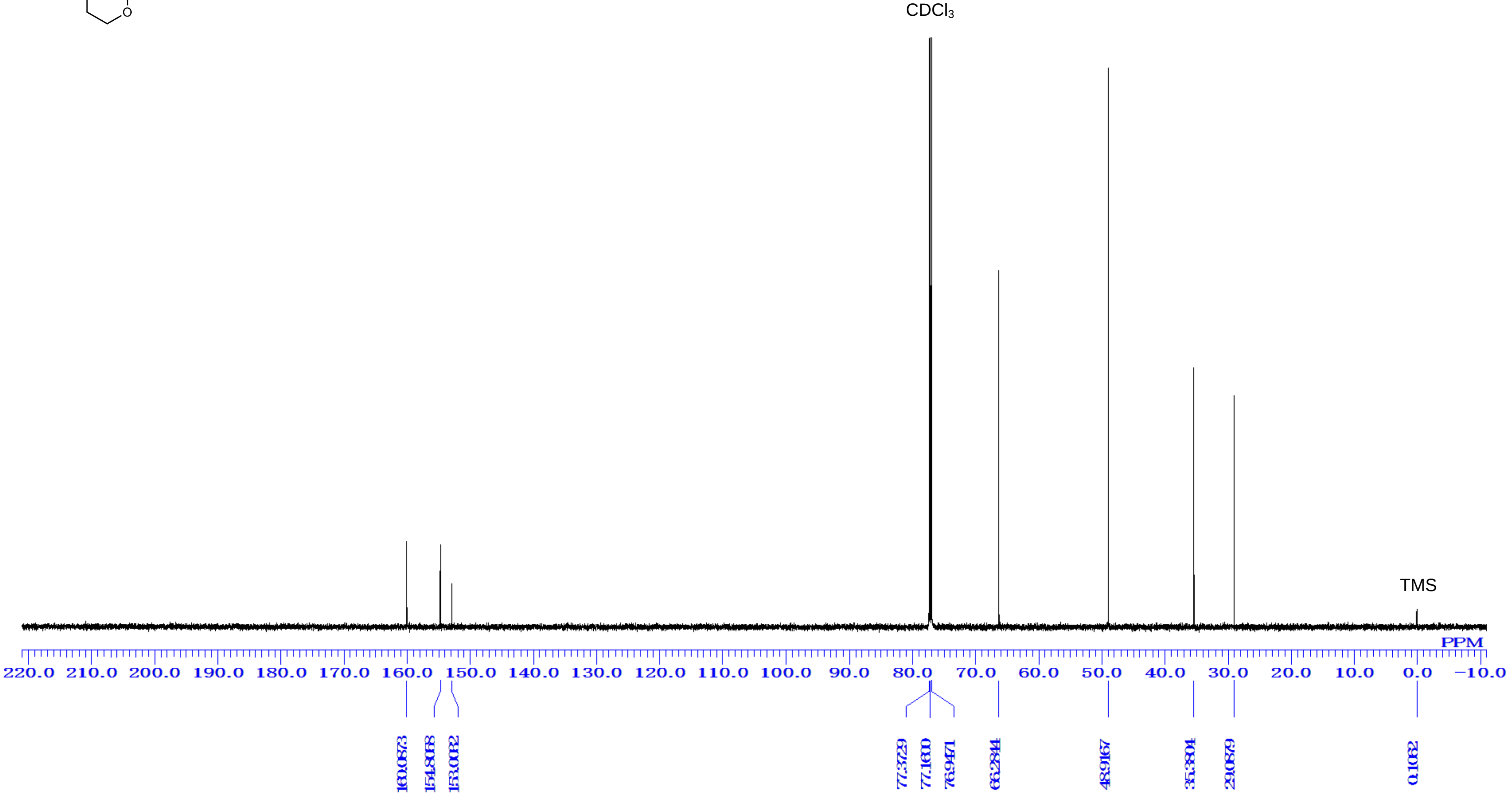

(4-*tert*-Butyl-2,6-dimethyl)benzyl chloride (S4)

^{1}H NMR ($CDCl_3$, 400 MHz)

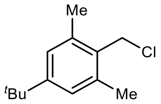

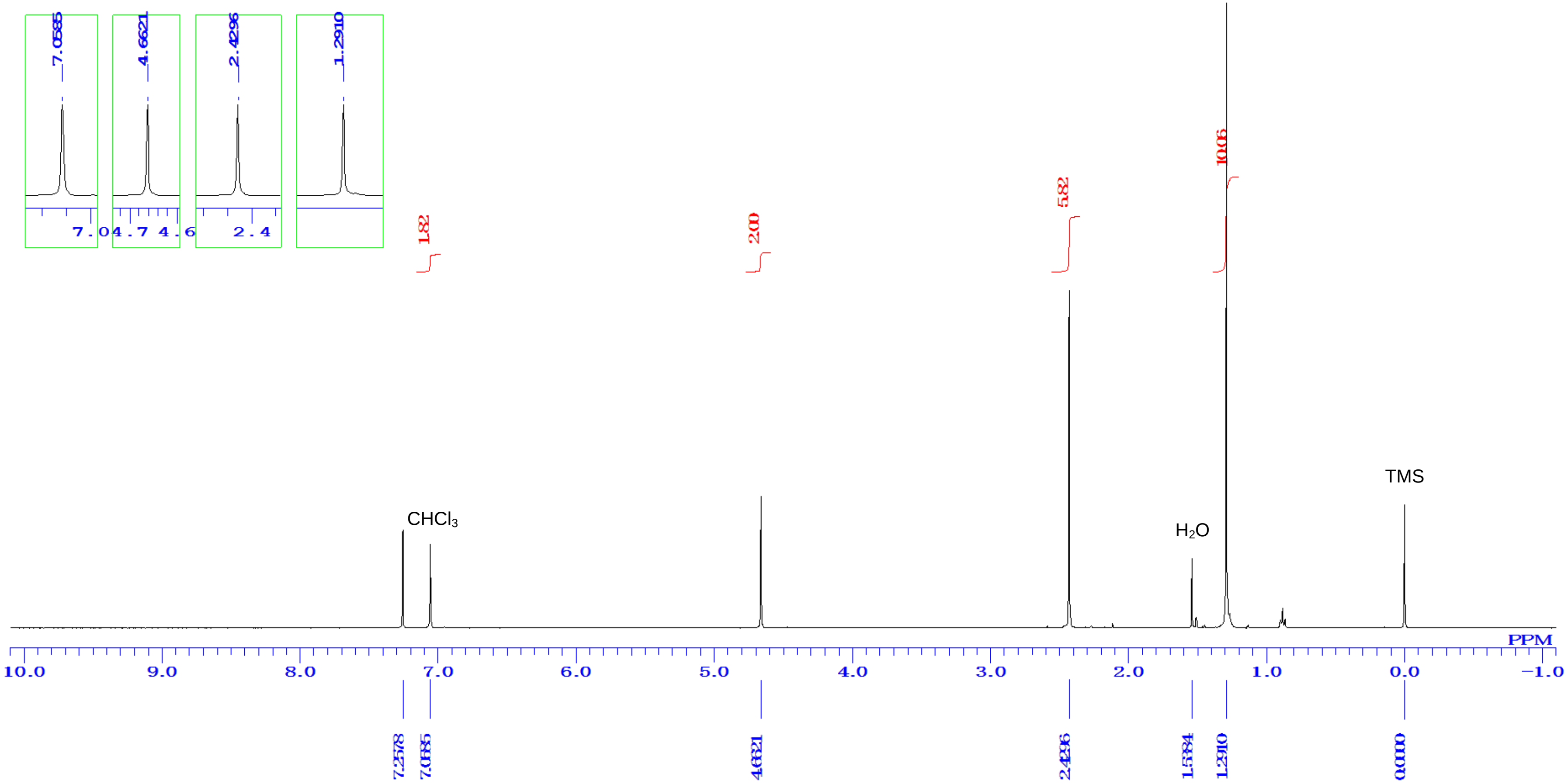

(4-*tert*-Butyl-2,6-dimethyl)benzyl chloride (S4)

$^{13}C\{^{1}H\}$ NMR ($CDCl_3$, 150 MHz)

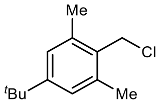

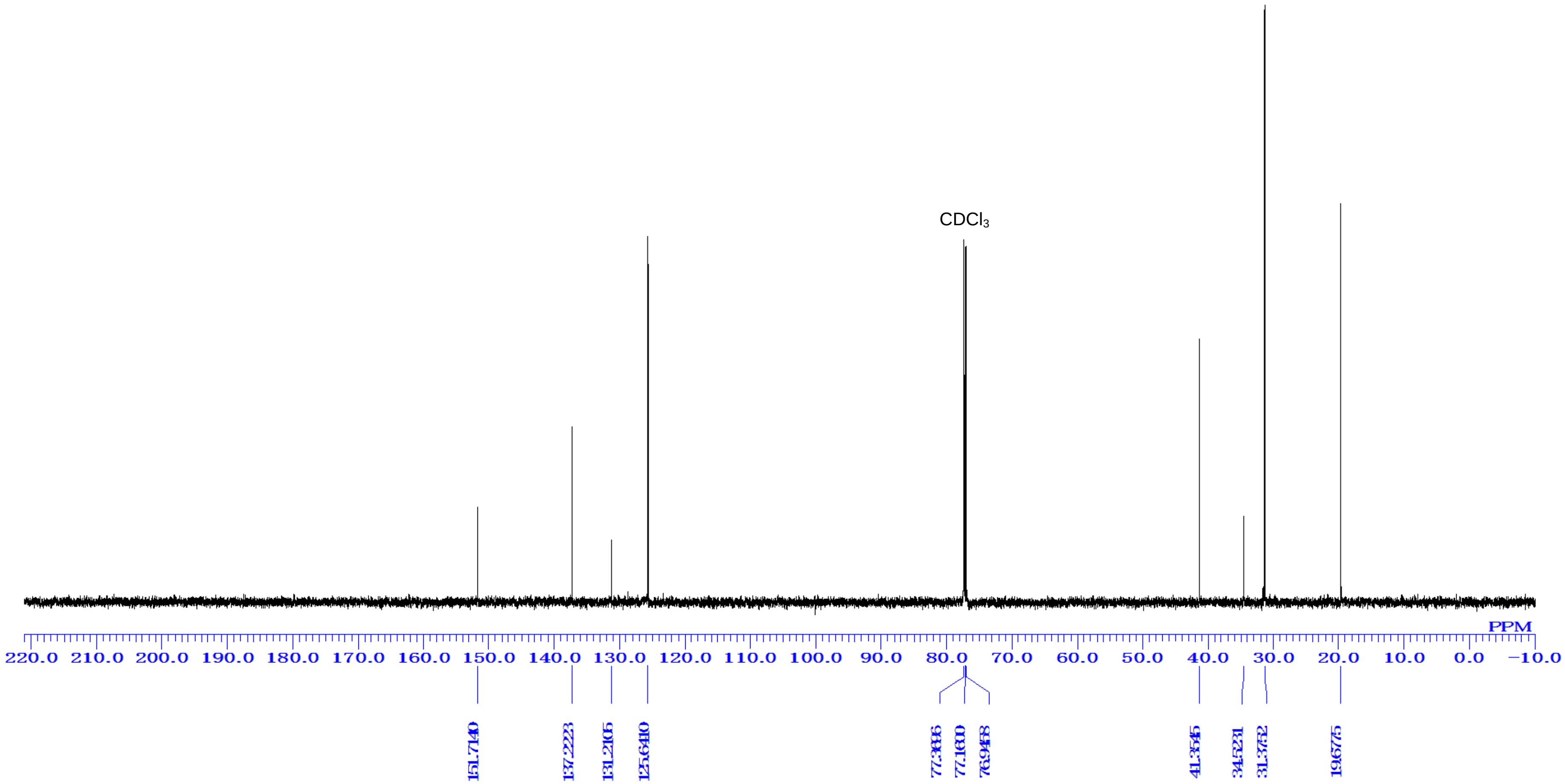

(4-*tert*-Butyl-2,6-dimethyl)benzyl alcohol (5b)

^{1}H NMR ($CDCl_3$, 600 MHz)

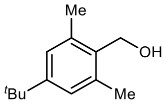

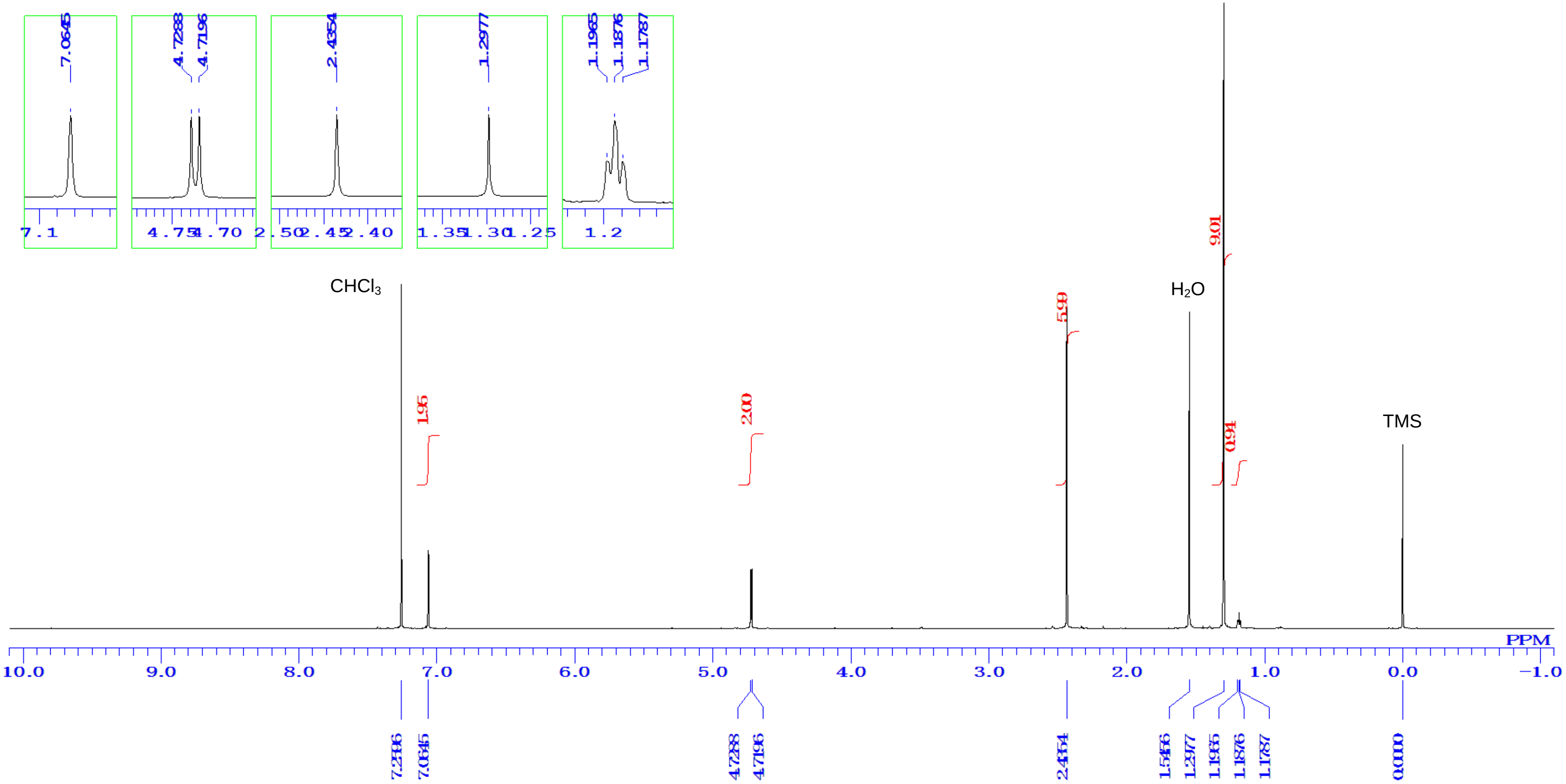

(4-*tert*-Butyl-2,6-dimethyl)benzyl alcohol (5b)
$^{13}C\{^{1}H\}$ NMR ($CDCl_3$, 150 MHz)

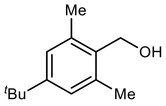

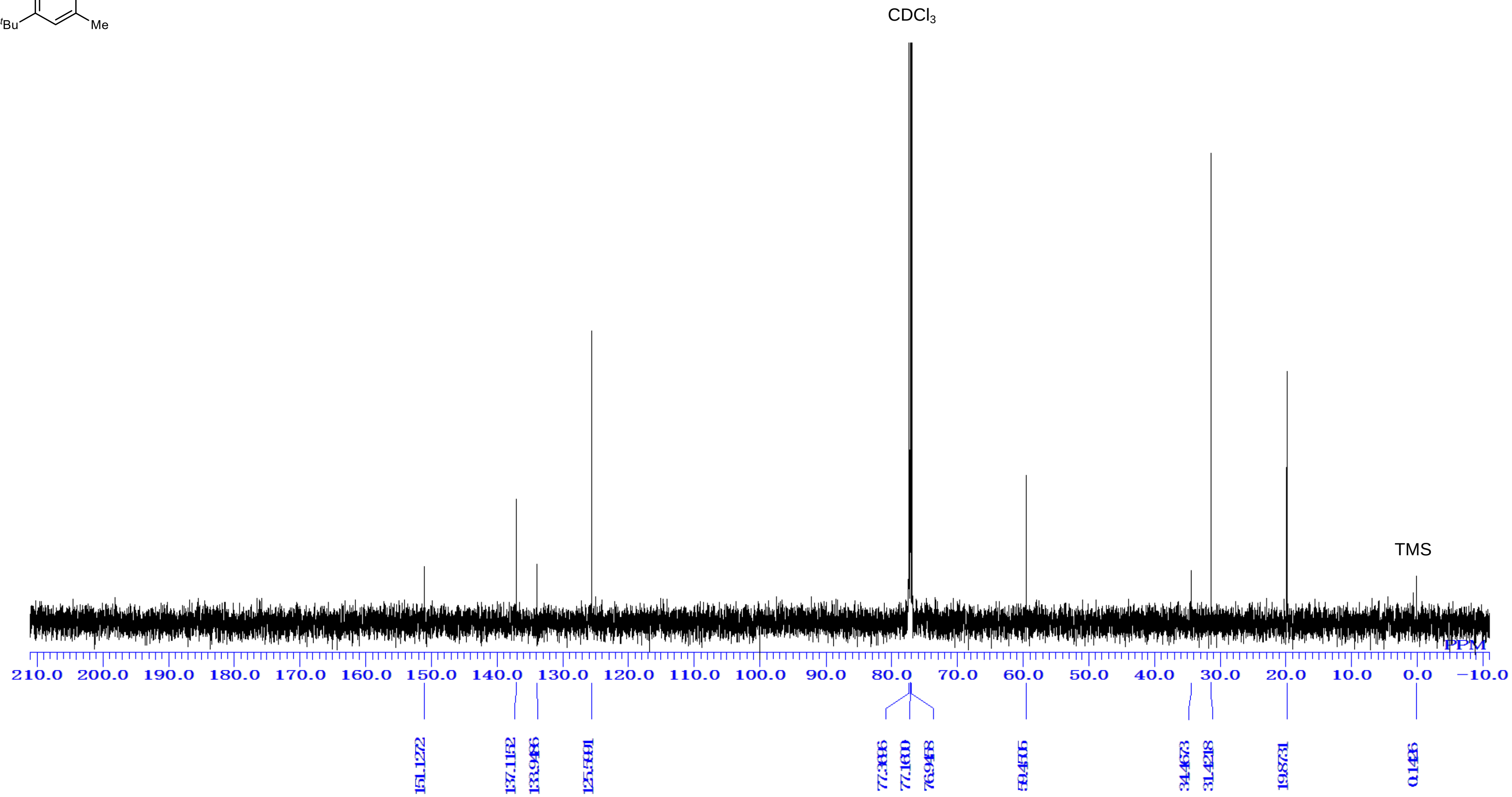

(10-Acetoxy)decyl 4-(*tert*-butyl)benzyl ether (7a)

^{1}H NMR ($CDCl_3$, 600 MHz)

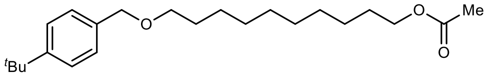

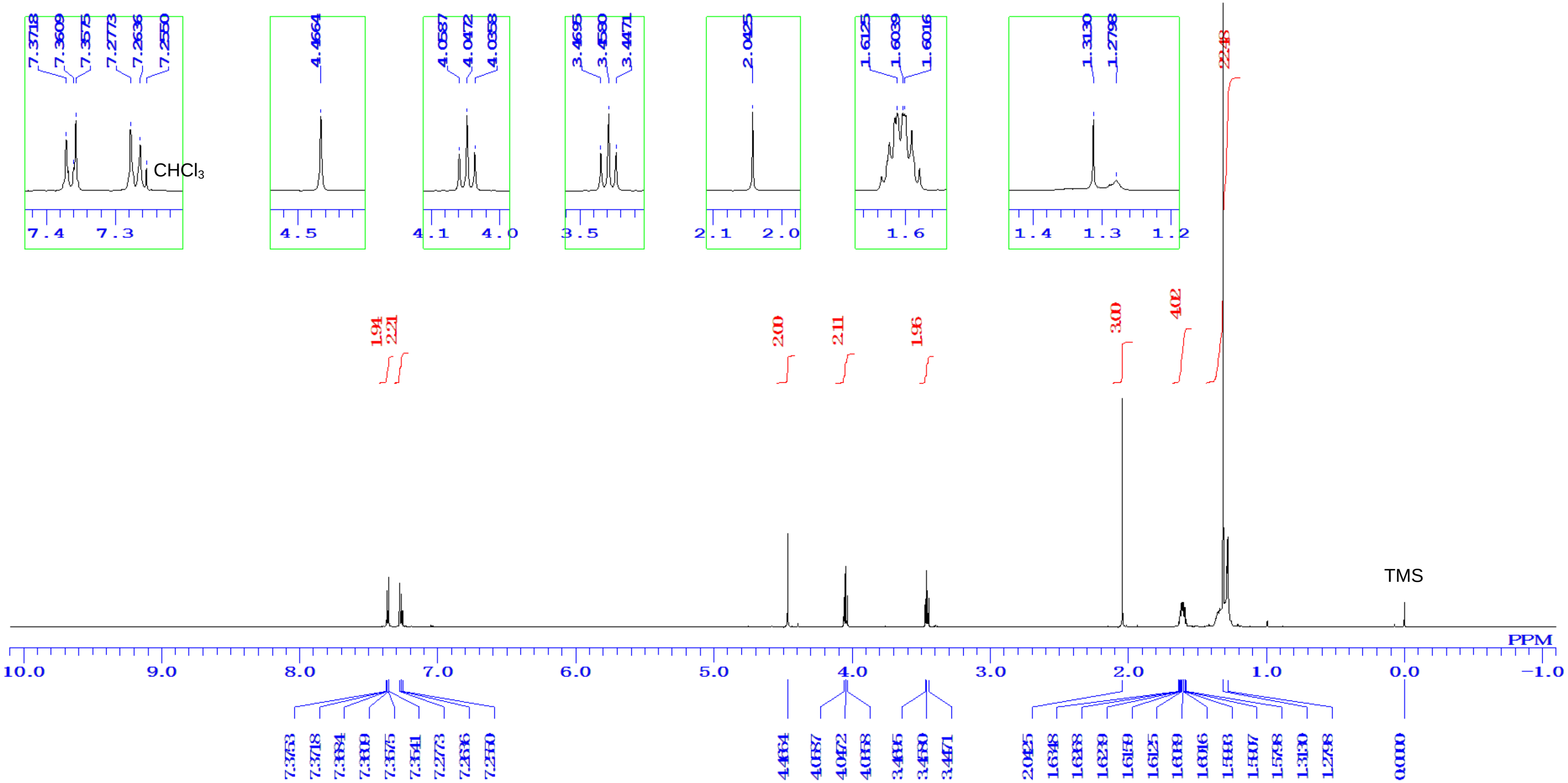

(10-Acetoxy)decyl 4-(*tert*-butyl)benzyl ether (7a)
$^{13}C\{^{1}H\}$ NMR ($CDCl_3$, 150 MHz)

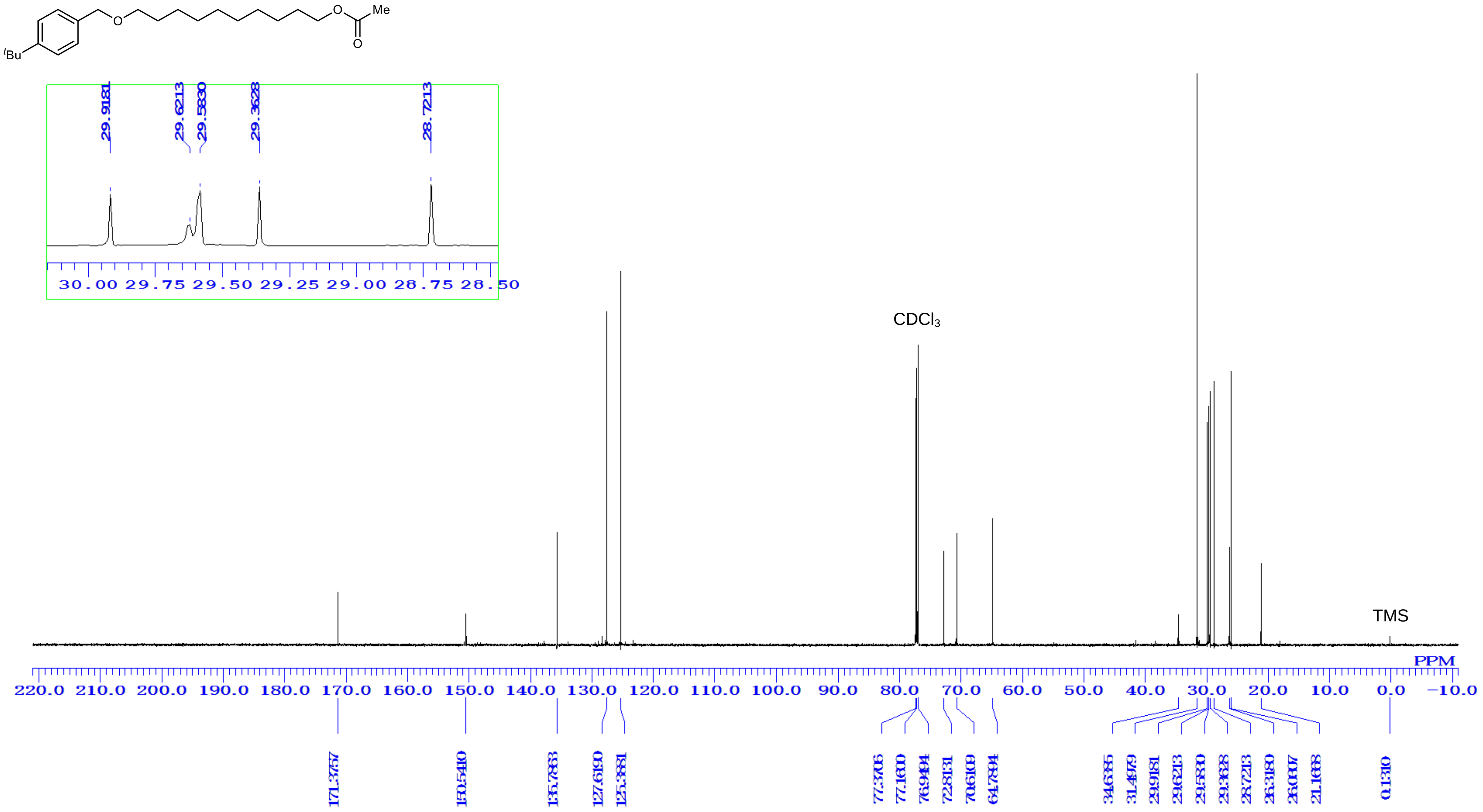

(10-Acetoxy)decyl (4-*tert*-butyl-2,6-dimethyl)benzyl ether (7b)

^{1}H NMR ($CDCl_3$, 600 MHz)

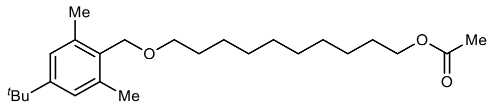

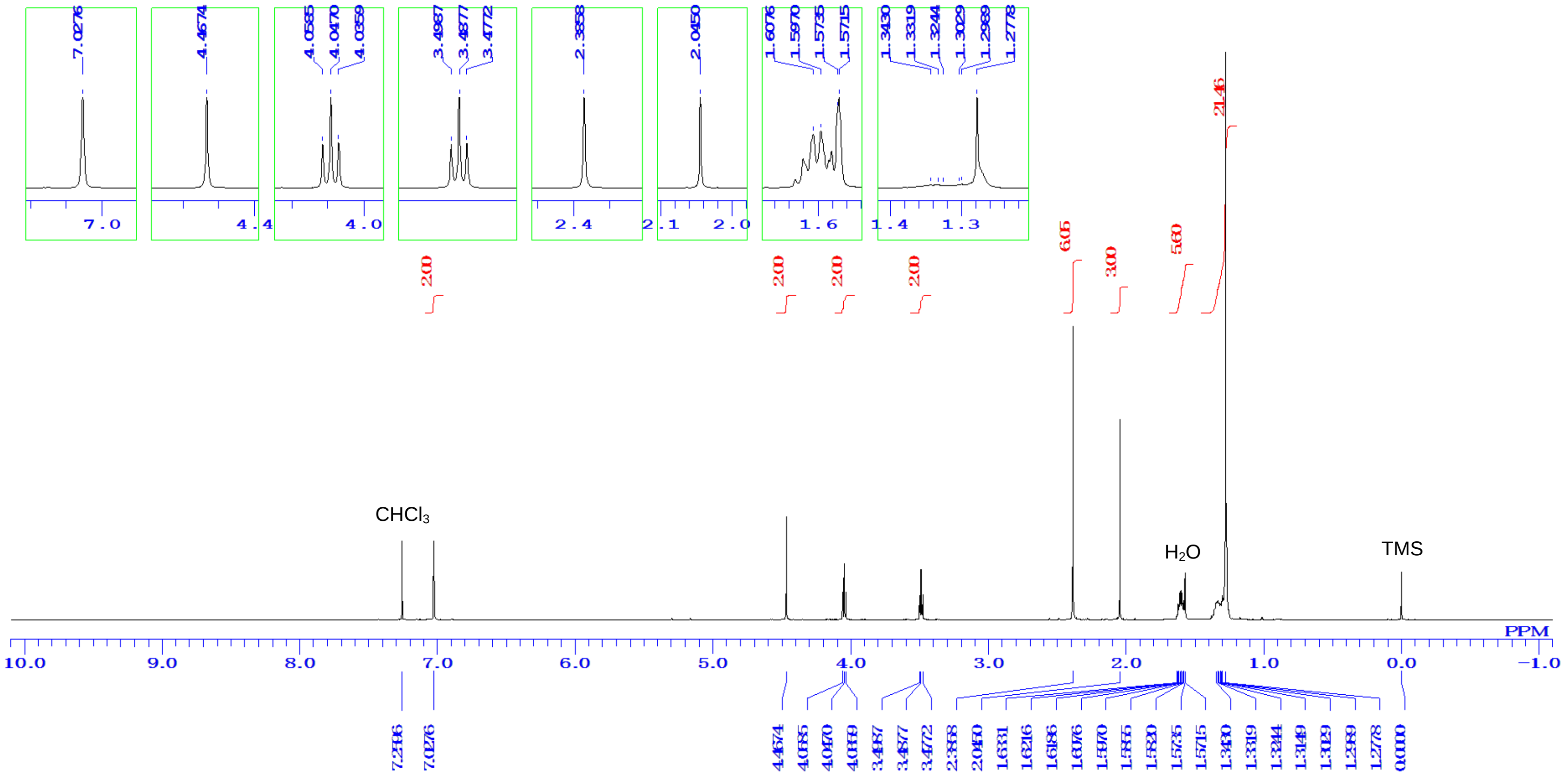

(10-Acetoxy)decyl (4-*tert*-butyl-2,6-dimethyl)benzyl ether (7b)

$^{13}C\{^{1}H\}$ NMR ($CDCl_3$, 150 MHz)

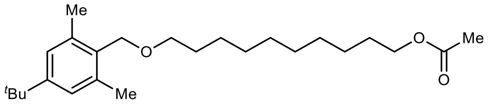

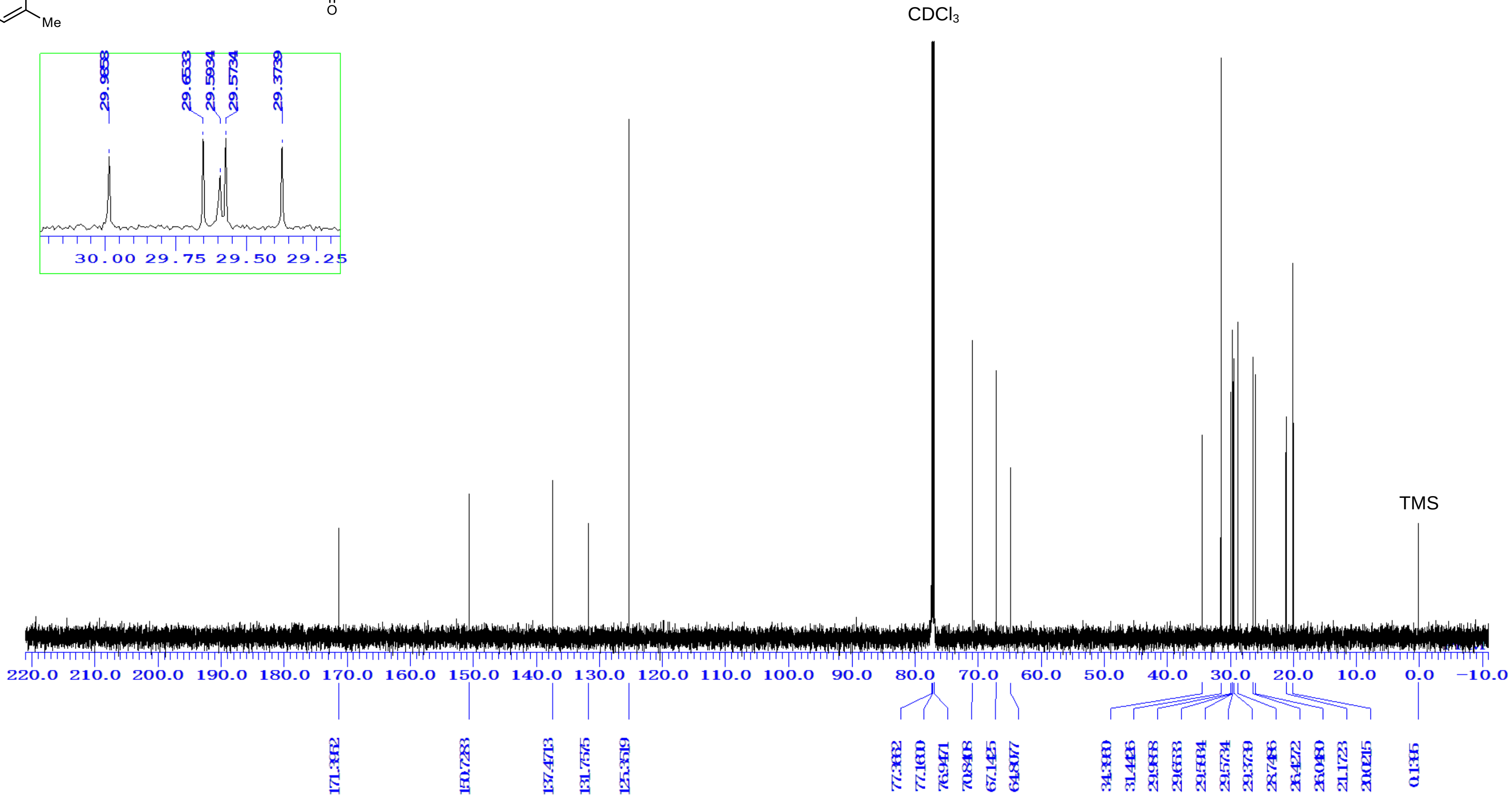

Bis(4-*tert*-butyl-2,6-dimethyl)benzyl ether (8b)

^1H NMR ($CDCl_3$, 400 MHz)

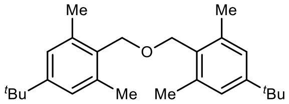

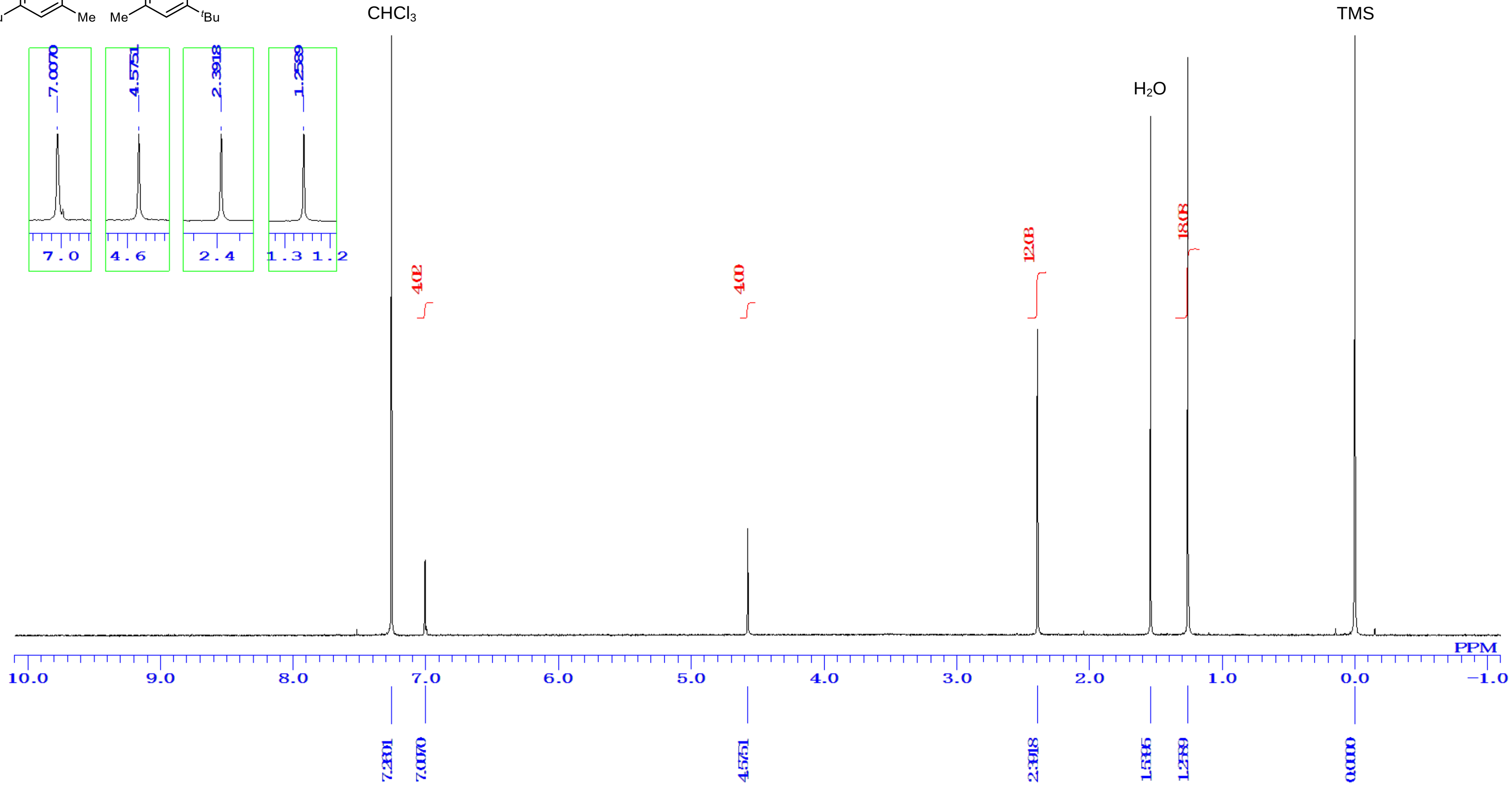

Bis(4-*tert*-butyl-2,6-dimethyl)benzyl ether (8b)

$^{13}C\{^{1}H\}$ NMR ($CDCl_3$, 150 MHz)

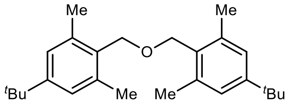

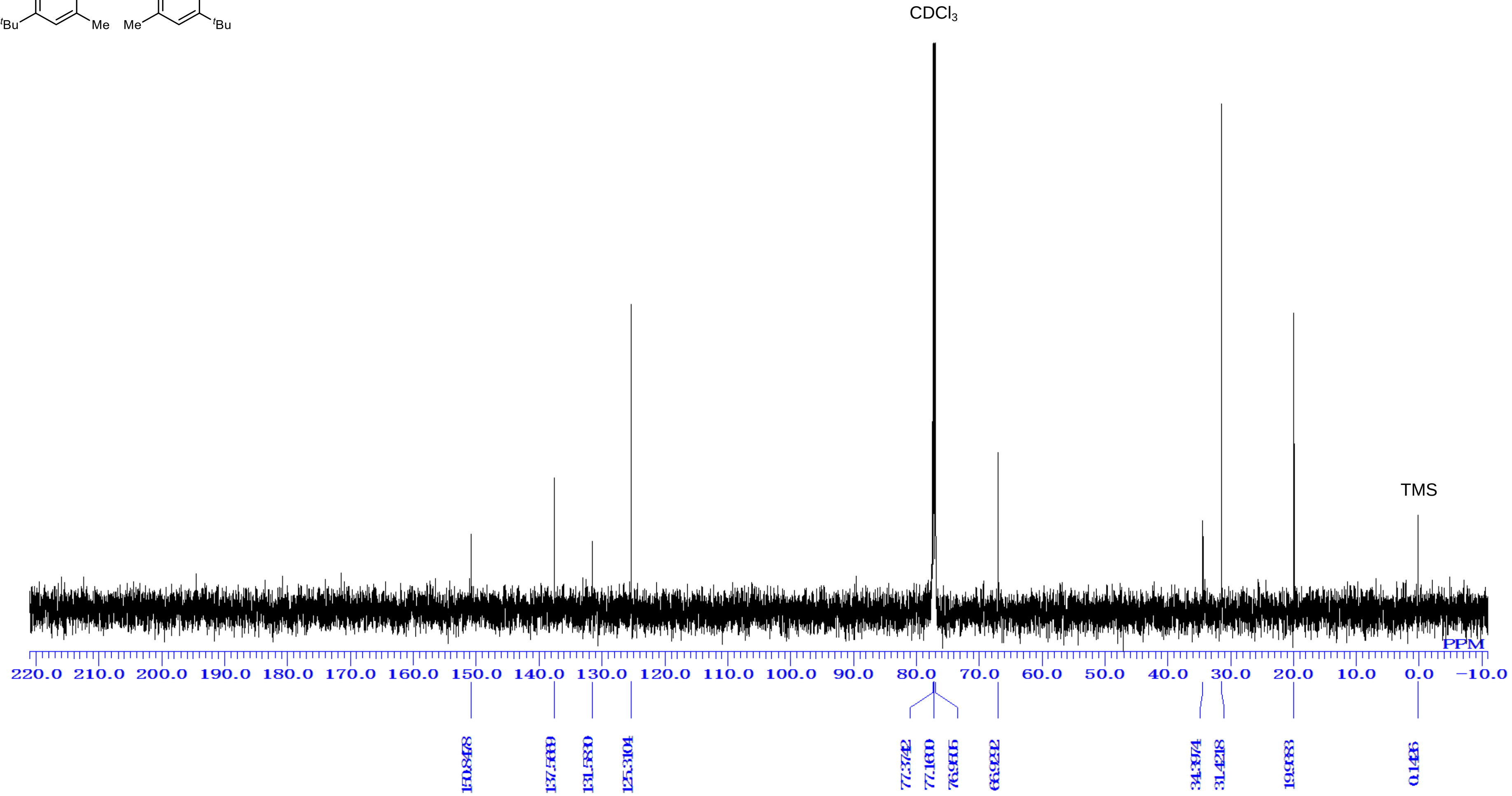

1,2,2,6,6-Pentamethylpiperidin-1-ium trifluoromethanesulfonate (S5)

^{1}H NMR ($CDCl_3$, 600 MHz)

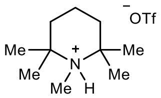

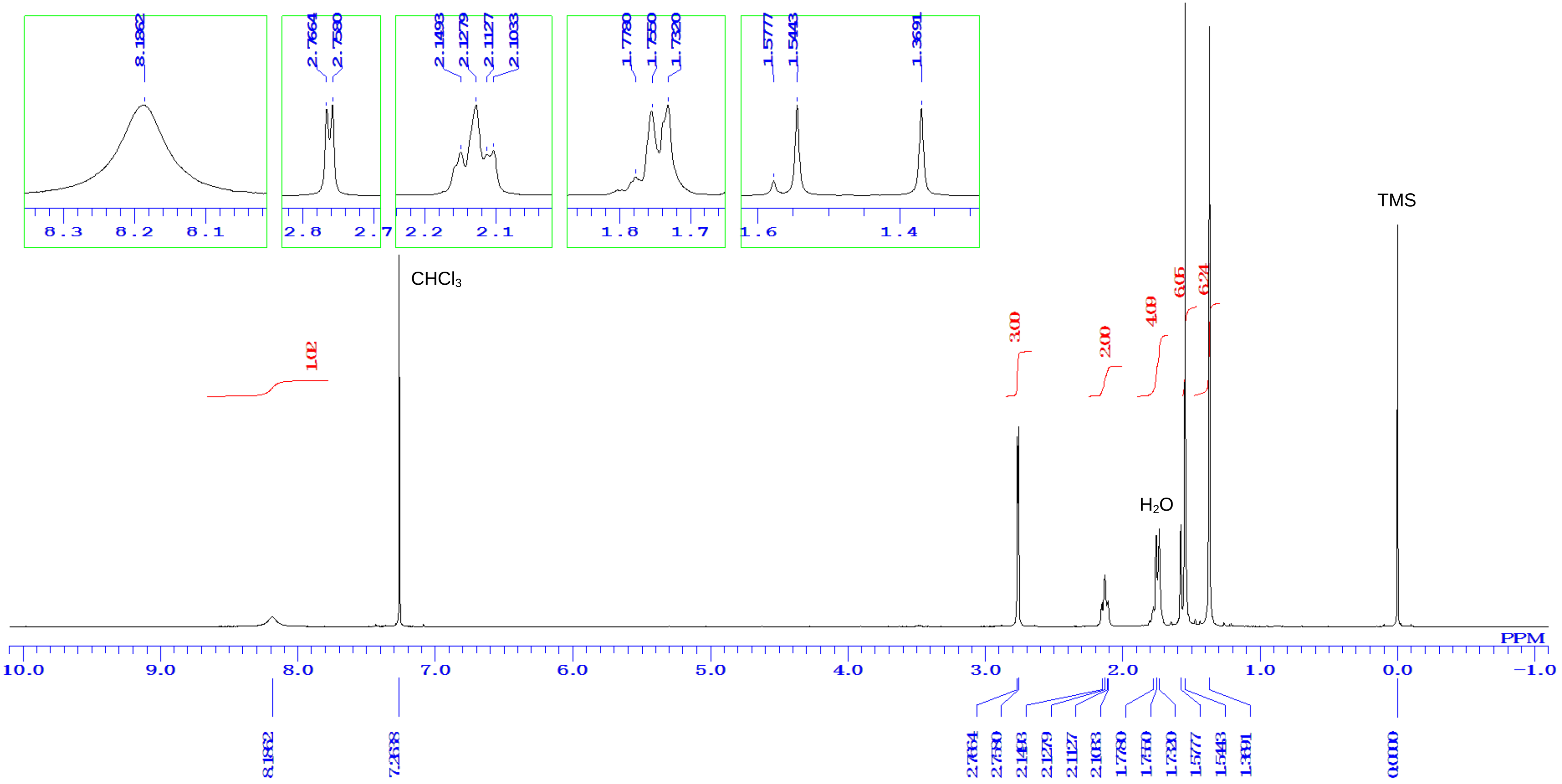

1,2,2,6,6-Pentamethylpiperidin-1-ium trifluoromethanesulfonate (S5)

$^{13}C\{^1H\}$ NMR ($CDCl_3$, 150 MHz)

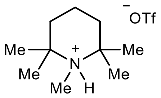

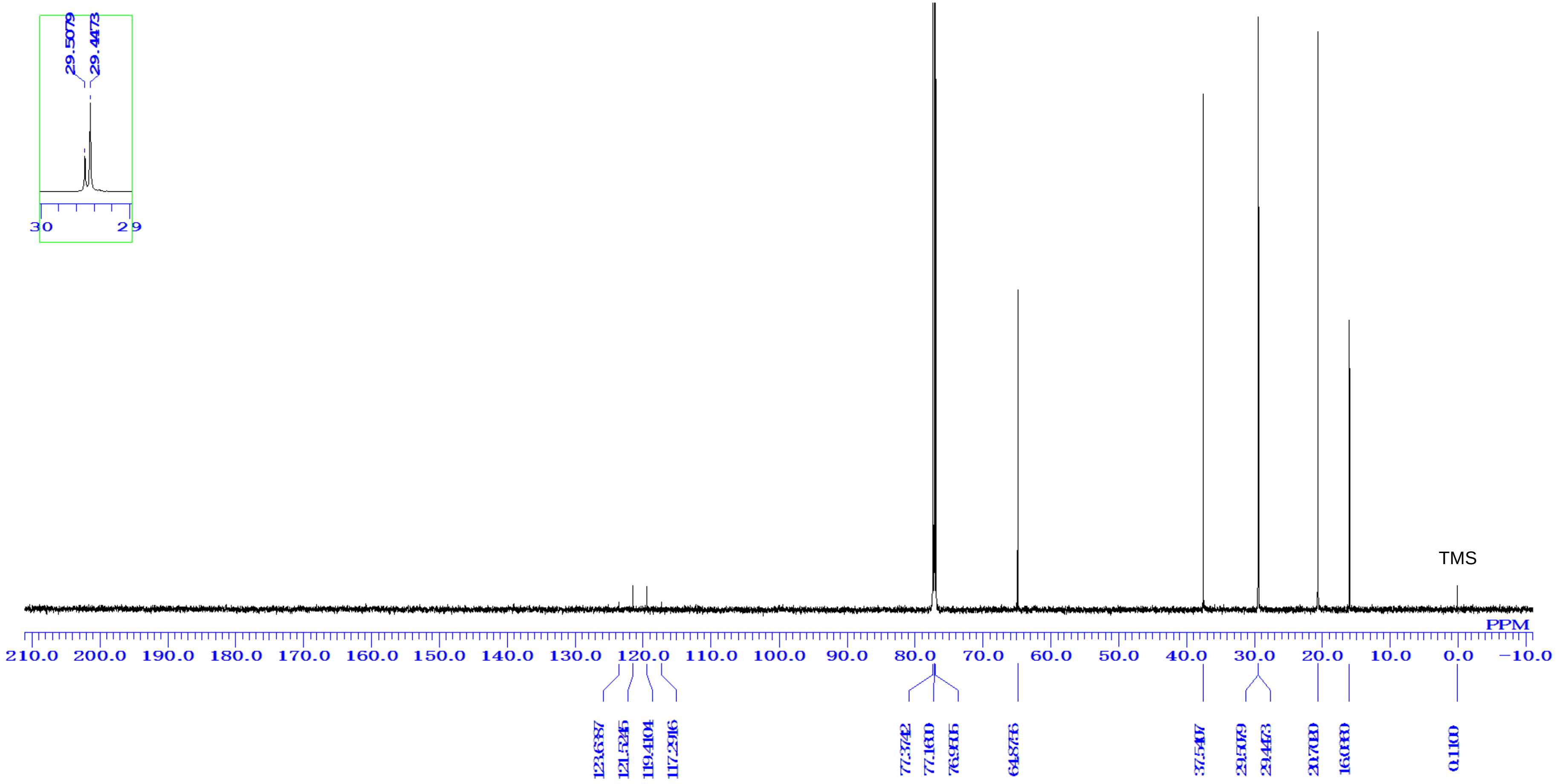

Bis(4-*tert*-butylbenzyl) ether (8a)

^{1}H NMR ($CDCl_3$, 600 MHz)

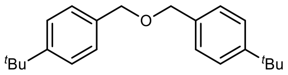

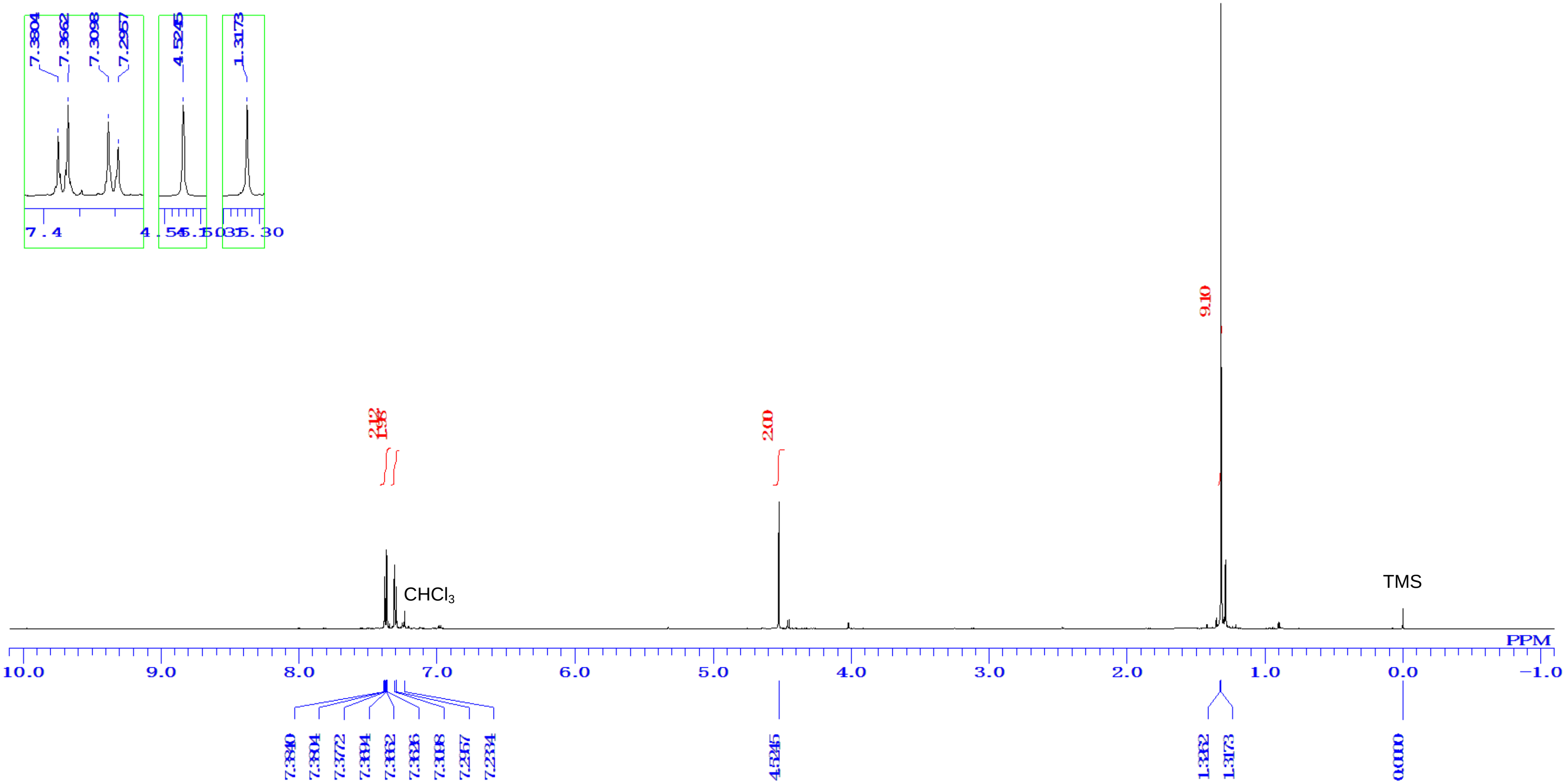

Bis(4-*tert*-butylbenzyl) ether (8a)

$^{13}C\{^{1}H\}$ NMR ($CDCl_3$, 150 MHz)

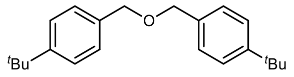

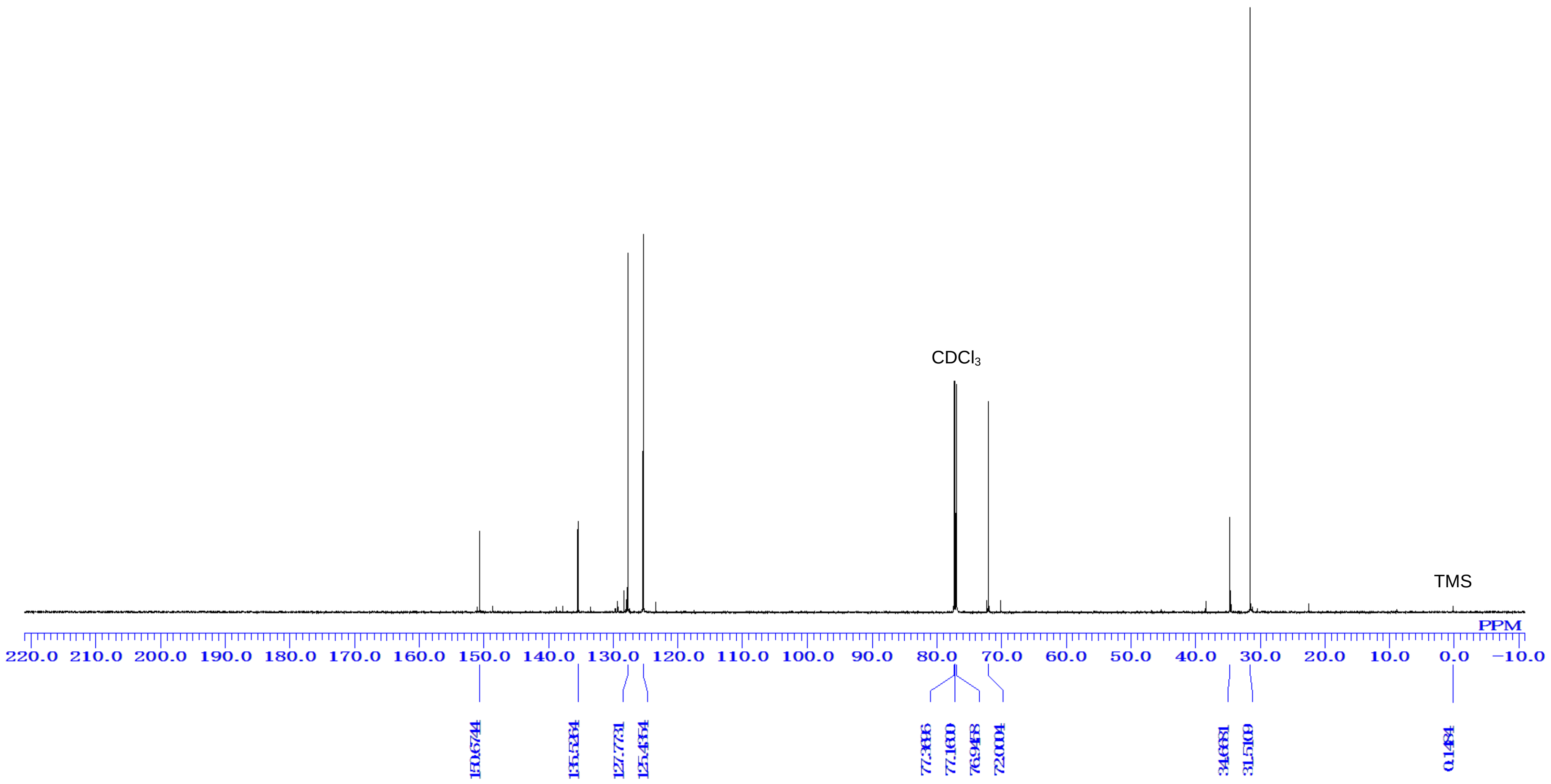

Carbocationoid 3a

^{1}H NMR ($CDCl_3$, 600 MHz)

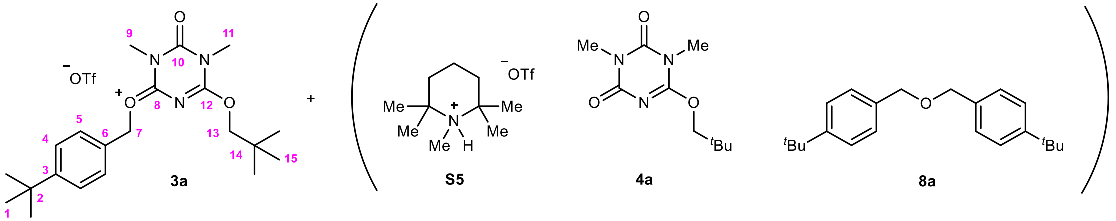

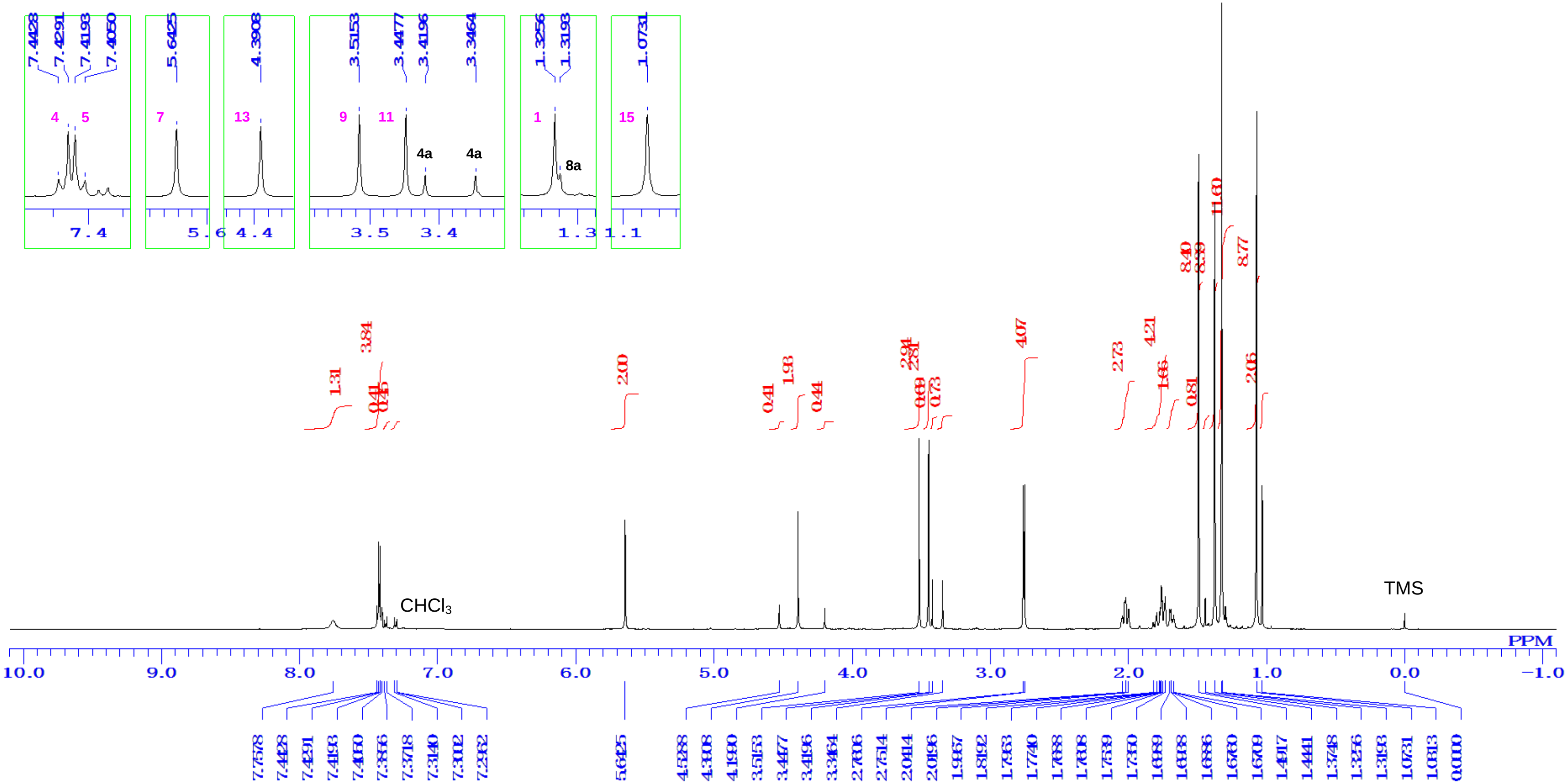

Carbocationoid 3a

$^{13}C\{^{1}H\}$ NMR ($CDCl_3$, 150 MHz)

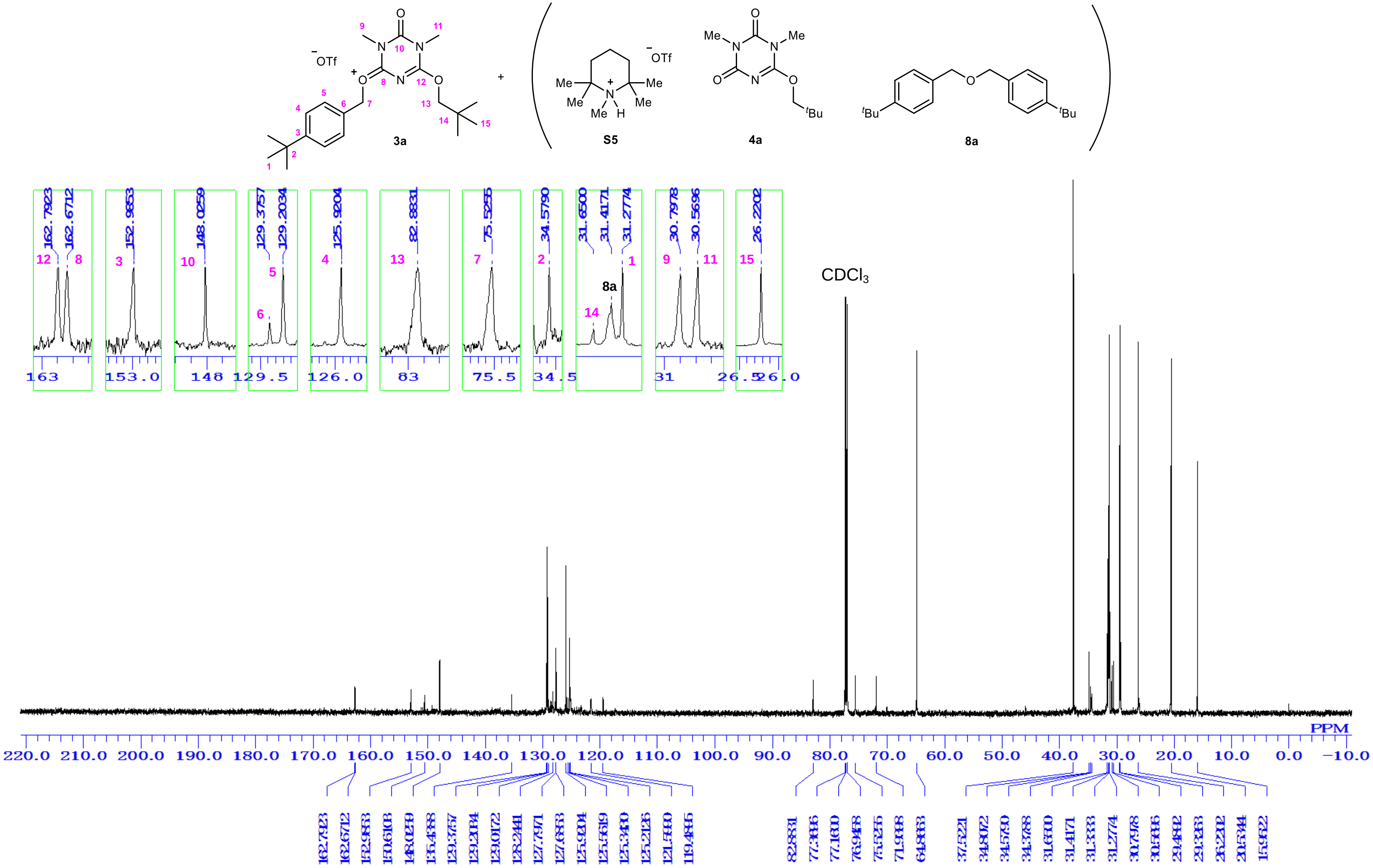

Carbocationoid 3b

^{1}H NMR ($CDCl_3$, 600 MHz)

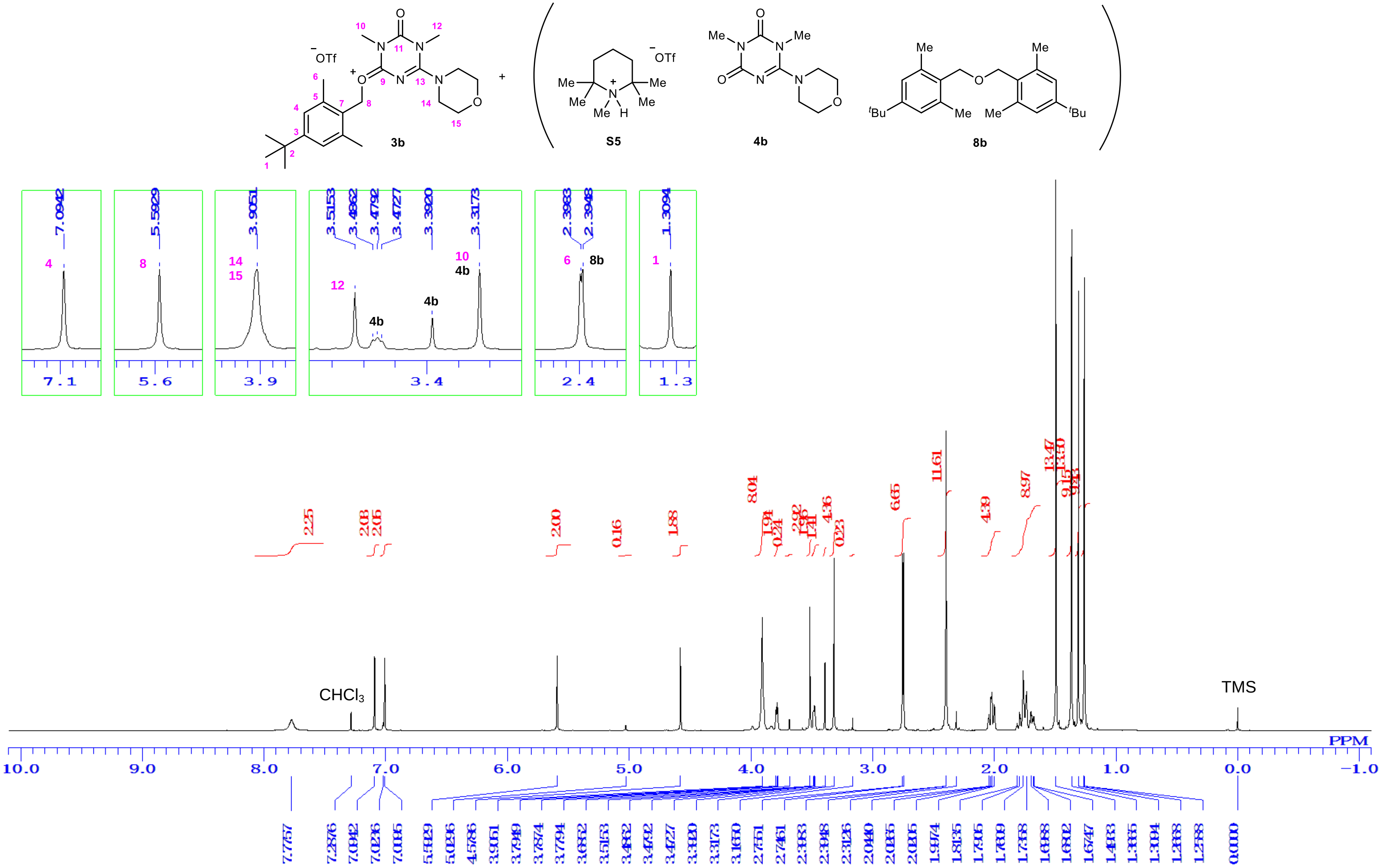

Carbocationoid 3b

$^{13}C\{^{1}H\}$ NMR ($CDCl_3$, 150 MHz)

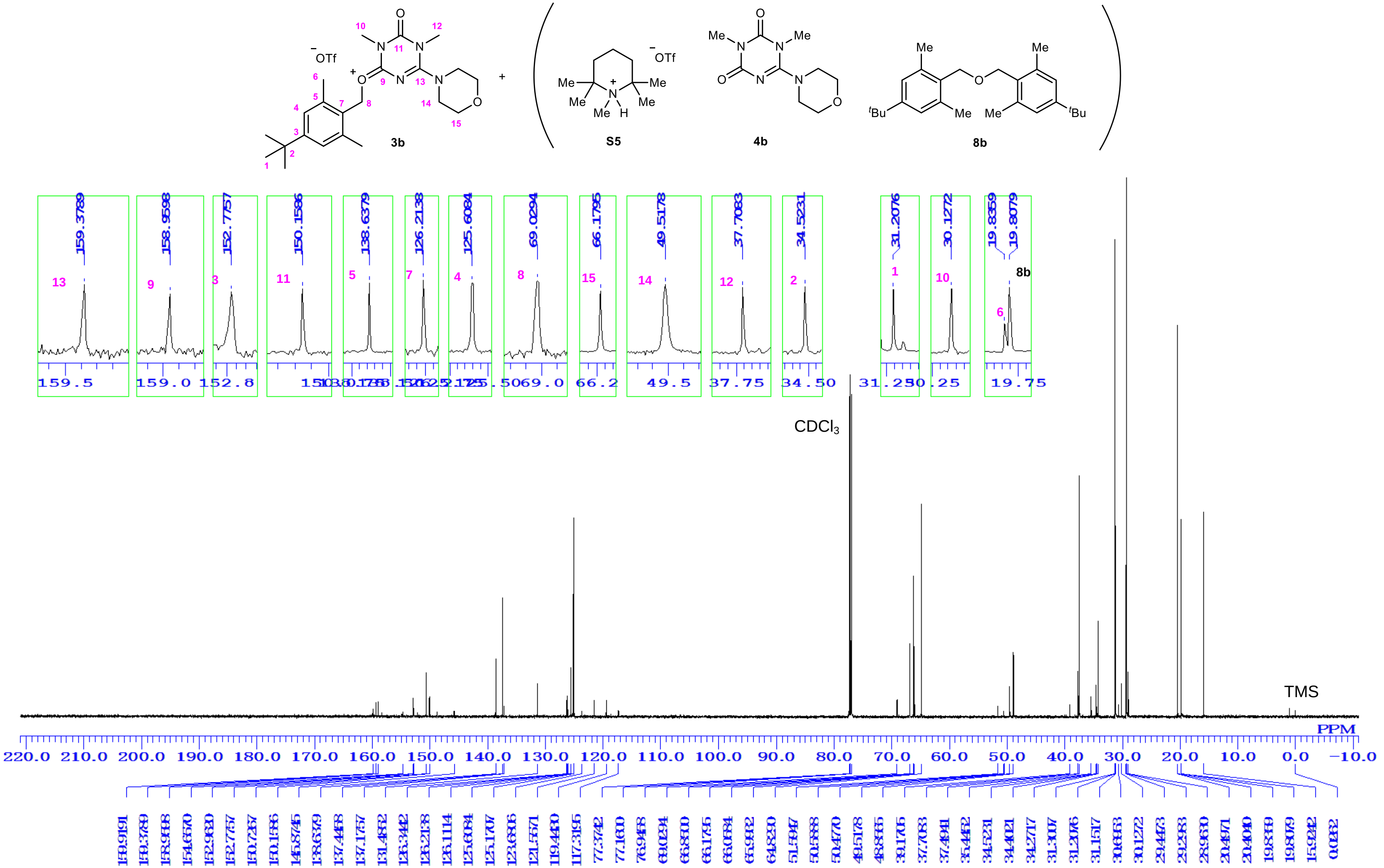

Benzhydryl 4-*tert*-butylbenzyl ether (24)

^{1}H NMR ($CDCl_3$, 600 MHz)

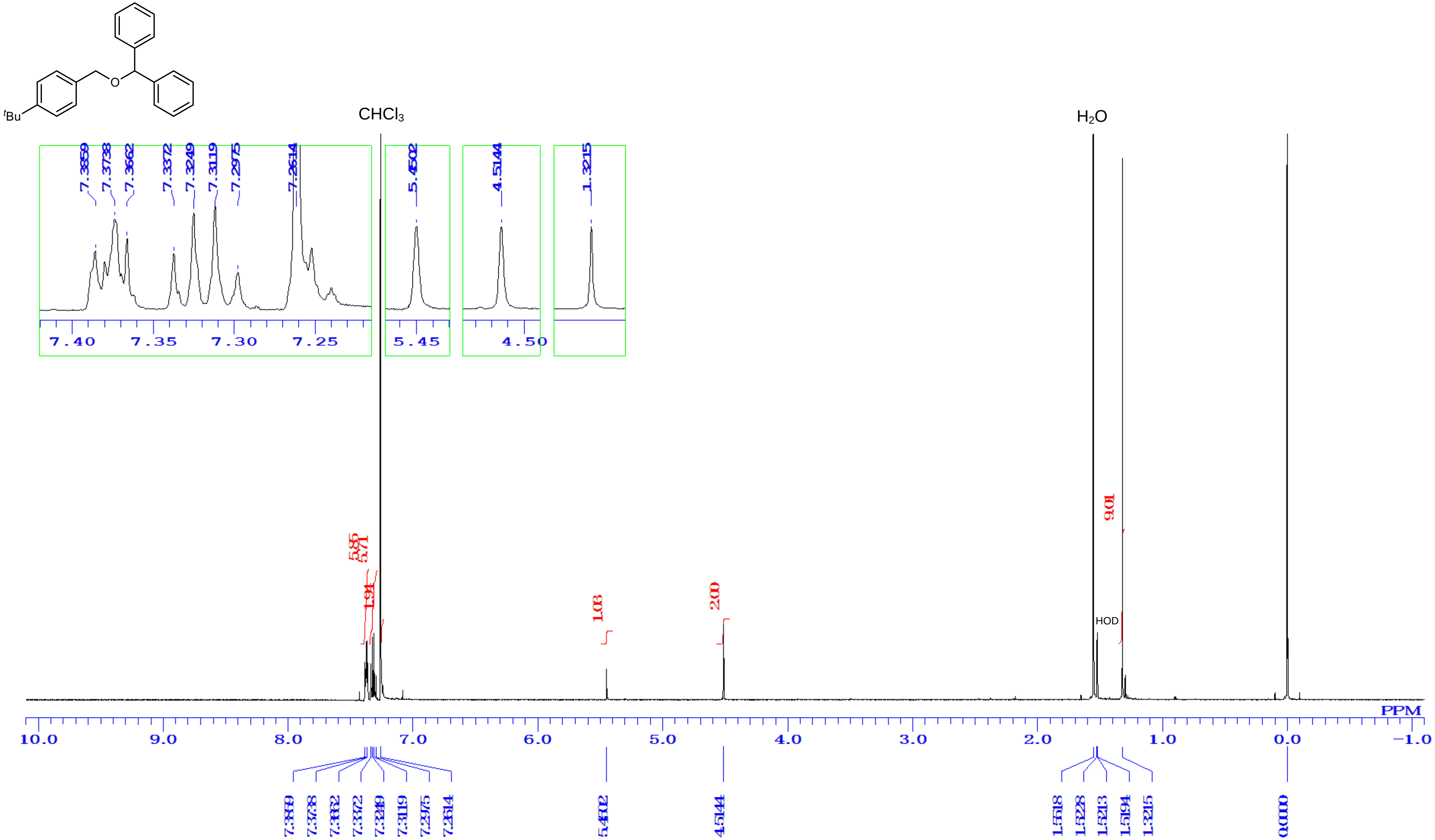

Benzhydryl 4-*tert*-butylbenzyl ether (24)

$^{13}C\{^1H\}$ NMR (150 MHz, $CDCl_3$)

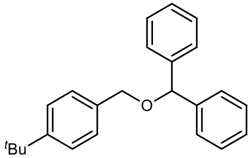

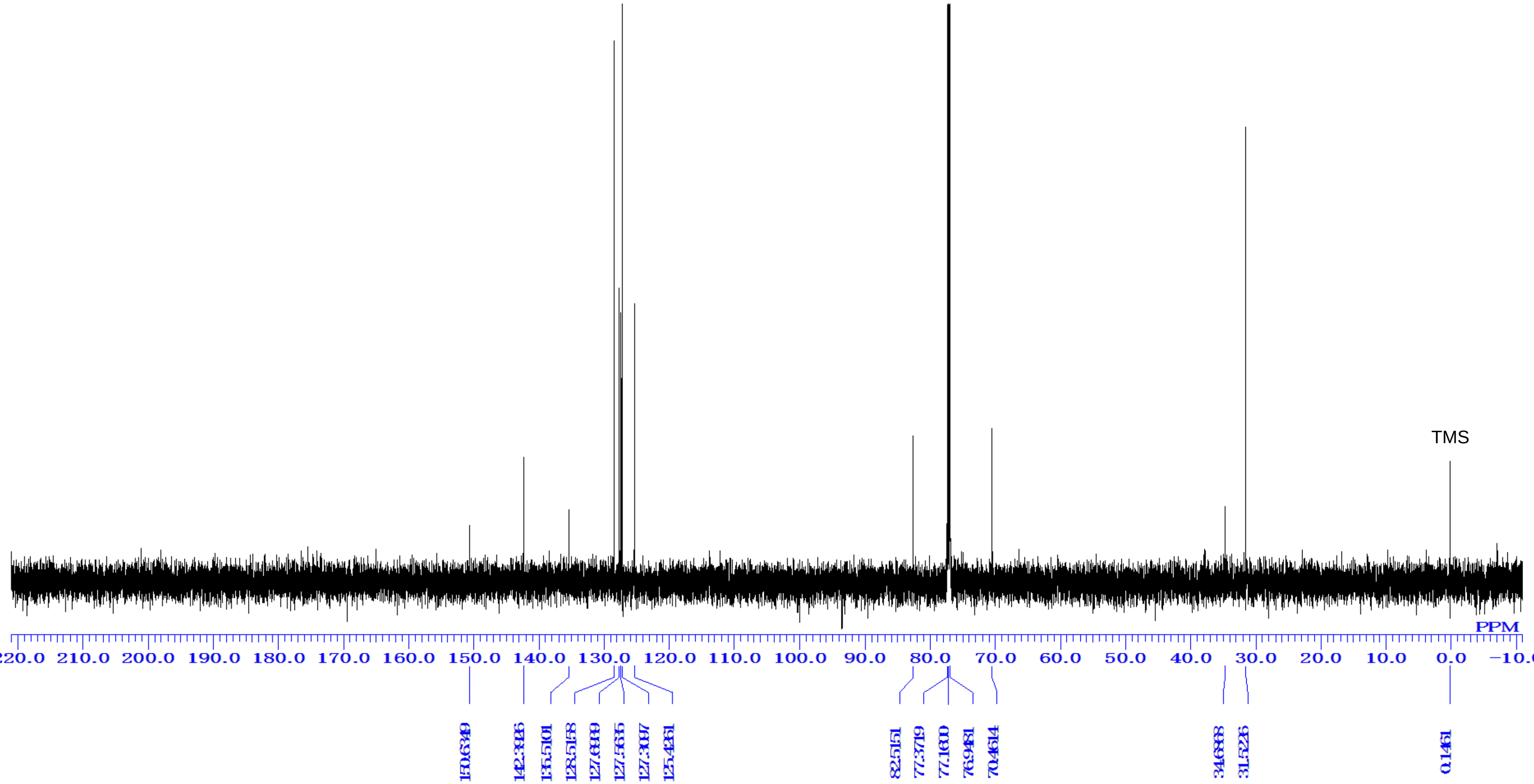

4-*tert*-Butylbenzyl 2-phenylpropan-2-yl ether (25)

^{1}H NMR ($CDCl_3$, 600 MHz)

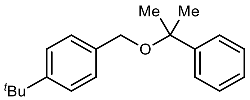

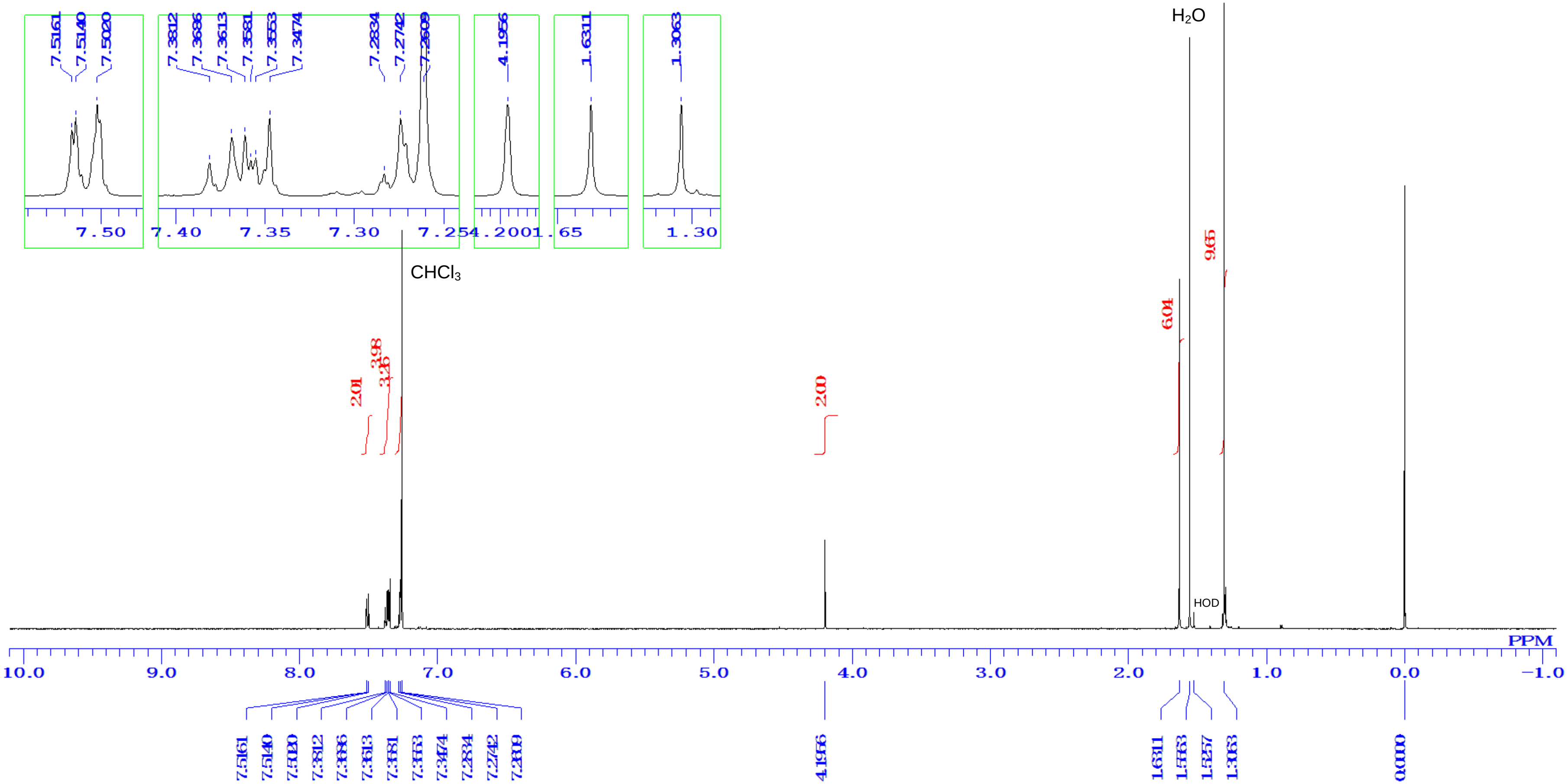

4-*tert*-Butylbenzyl 2-phenylpropan-2-yl ether (25)

$^{13}C\{^1H\}$ NMR (150 MHz, $CDCl_3$)

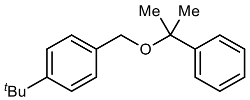

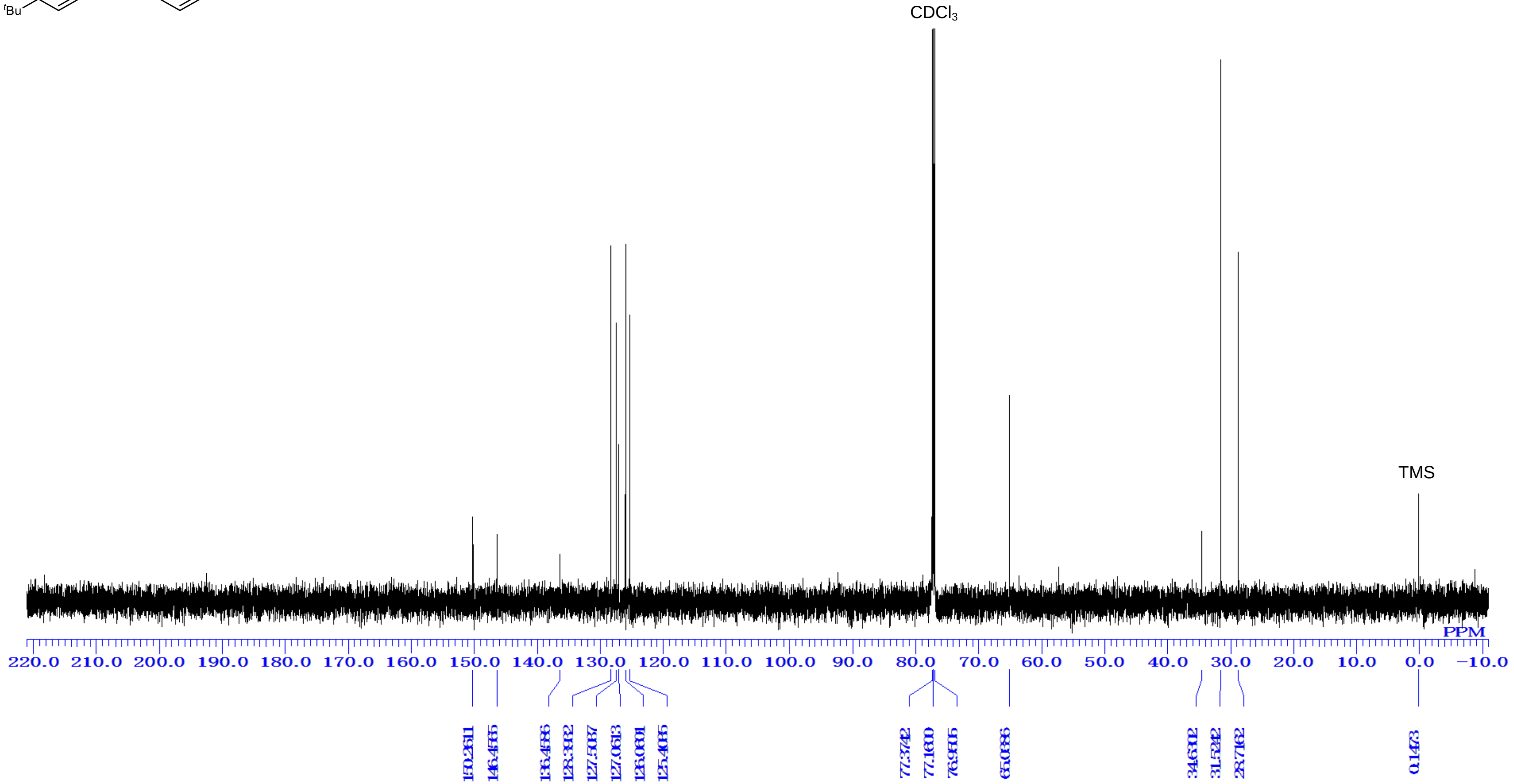

1-Adamantyl 4-*tert*-butylbenzyl ether (26)
^{1}H NMR ($CDCl_3$, 600 MHz)

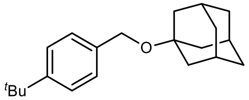

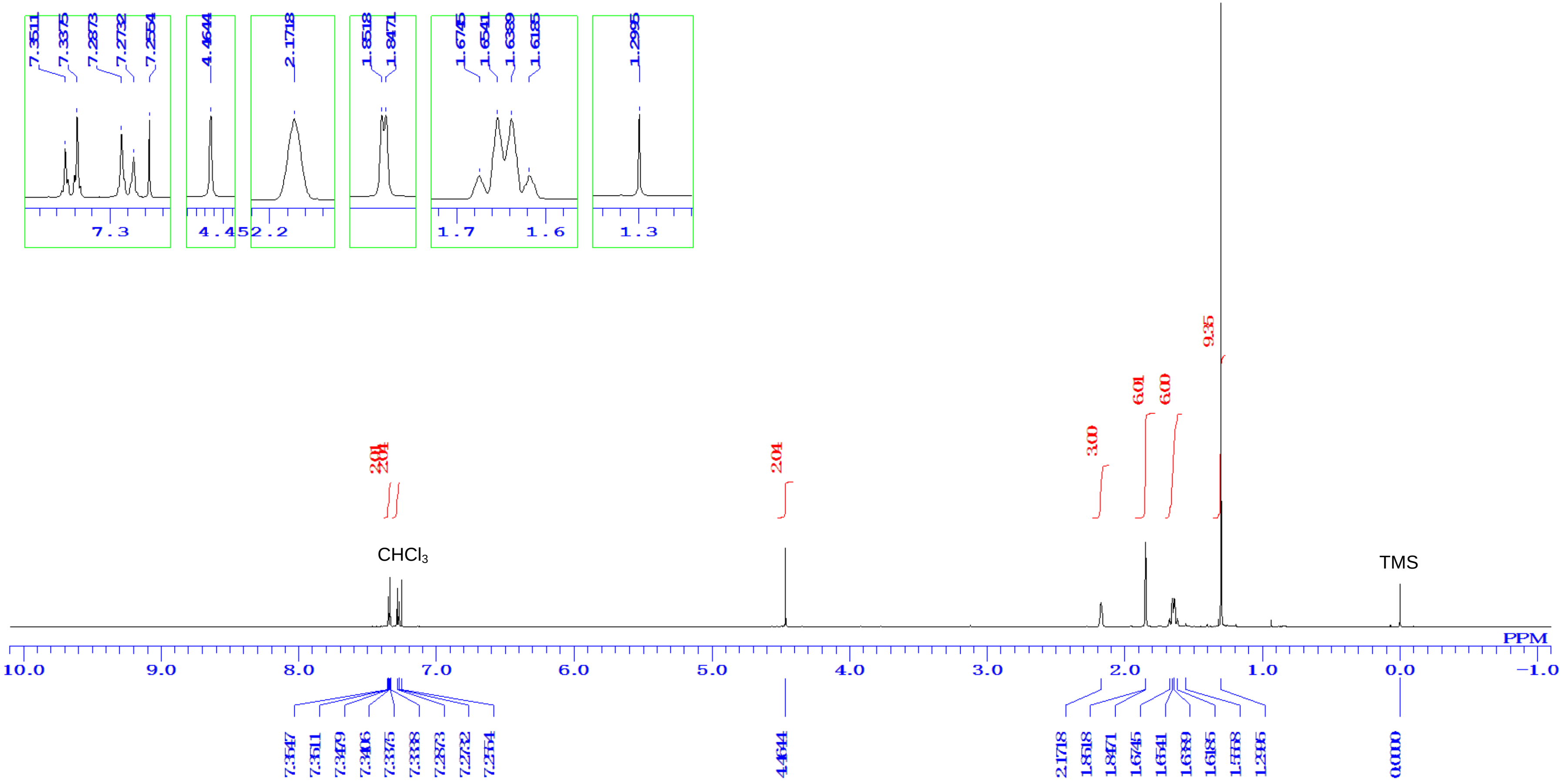

1-Adamantyl 4-*tert*-butylbenzyl ether (26)

$^{13}C\{^{1}H\}$ NMR ($CDCl_3$, 150 MHz)

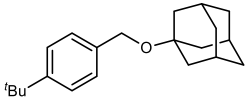

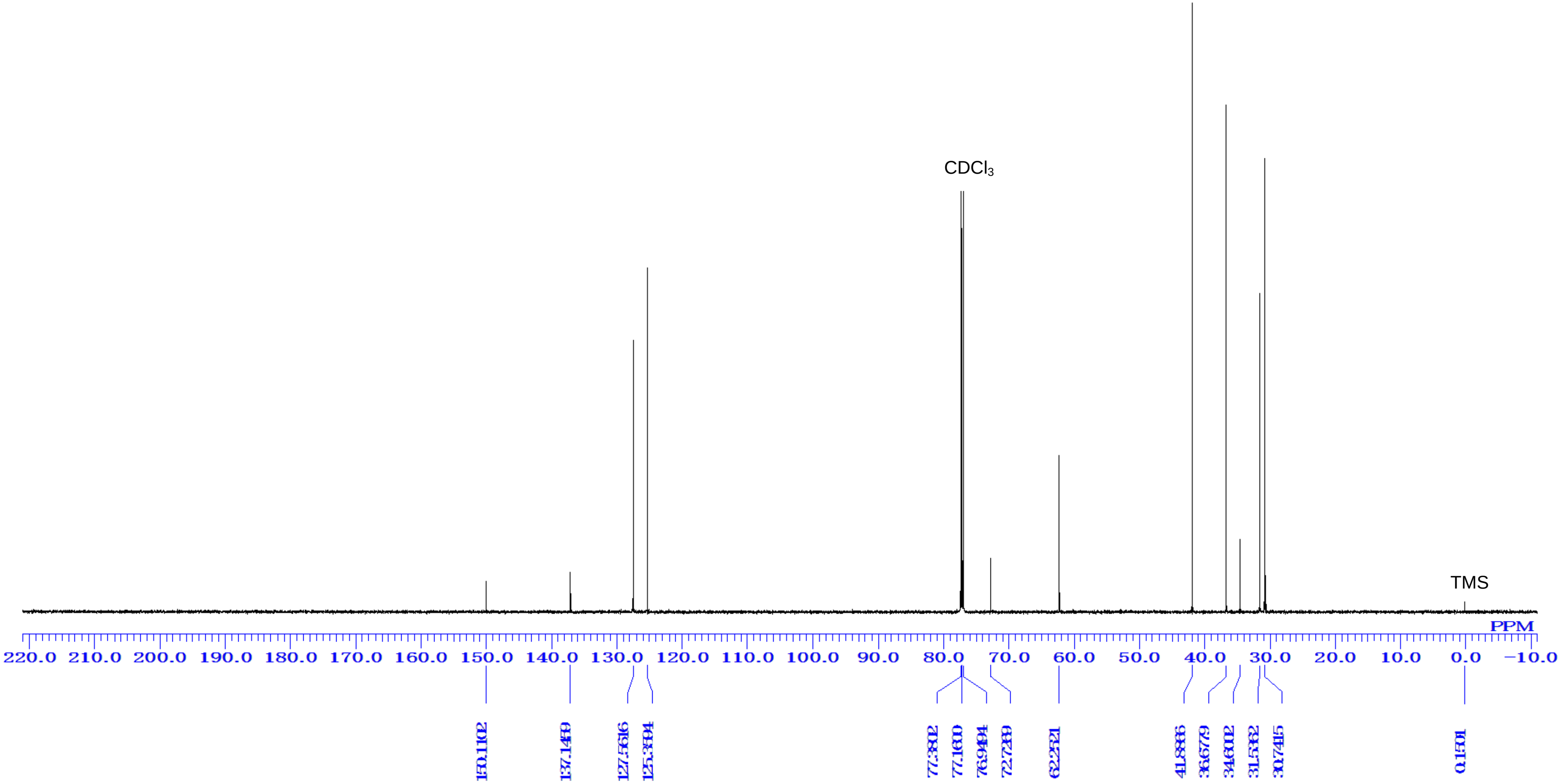

4-*tert*-Butylbenzyl 3-(trityloxy)propyl ether (27)

^{1}H NMR ($CDCl_3$, 600 MHz)

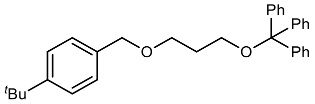

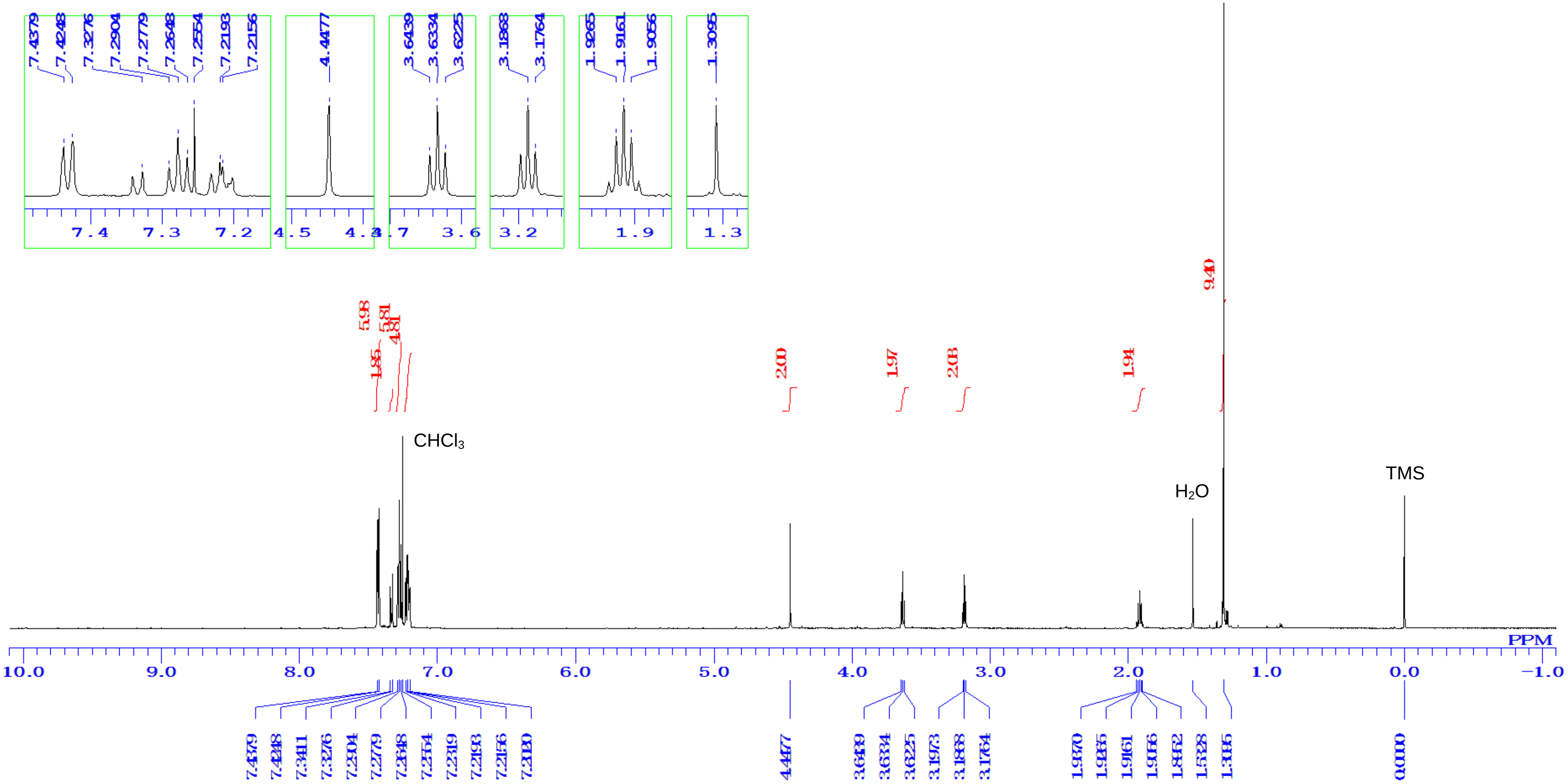

4-*tert*-Butylbenzyl 3-(trityloxy)propyl ether (27)

$^{13}C\{^{1}H\}$ NMR ($CDCl_3$, 150 MHz)

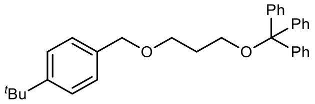

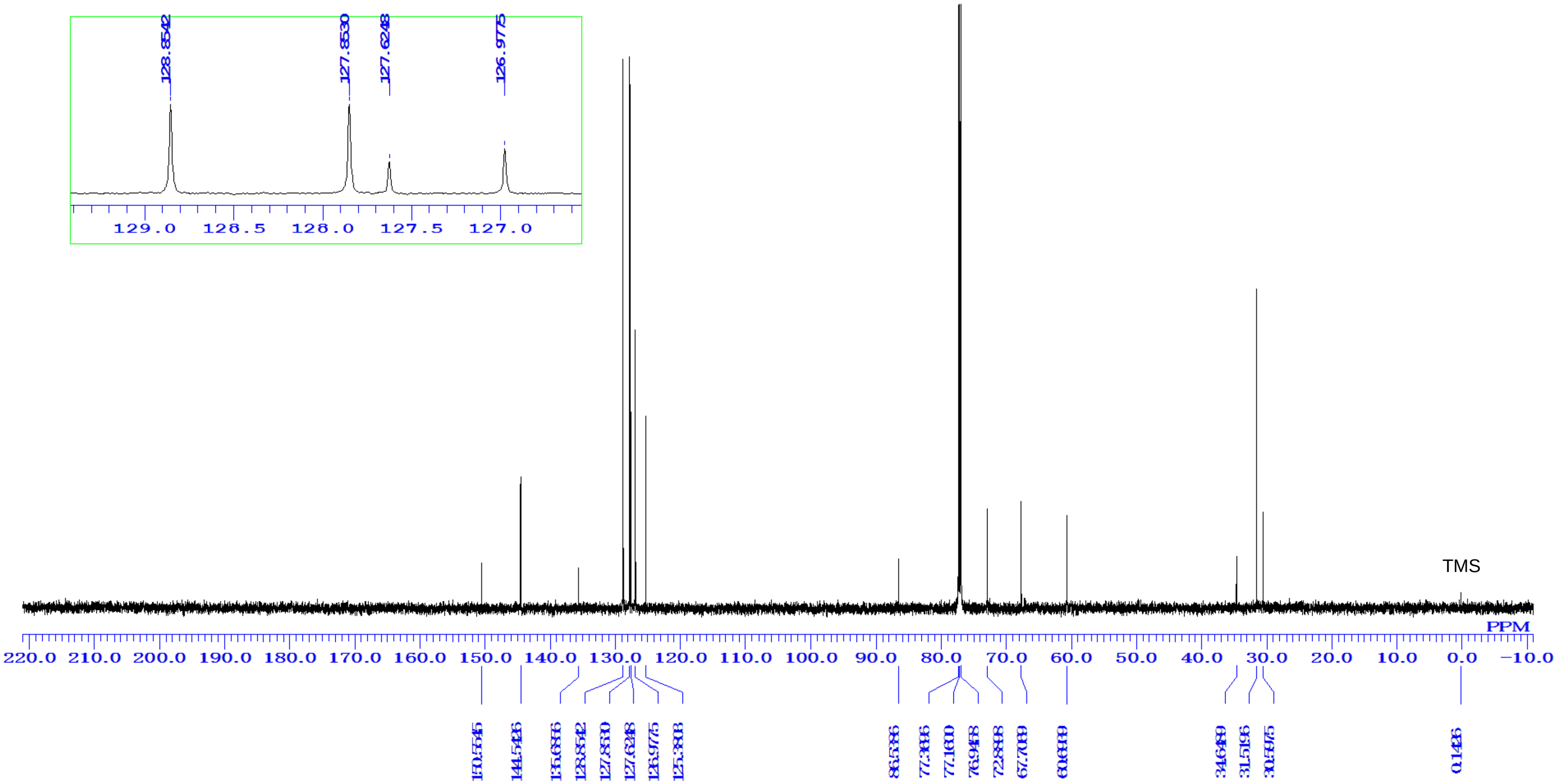

1,3-Bis[(4-*tert*-butylbenzyl)oxy]propane (28)

^1H NMR ($CDCl_3$, 600 MHz)

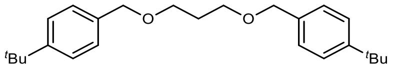

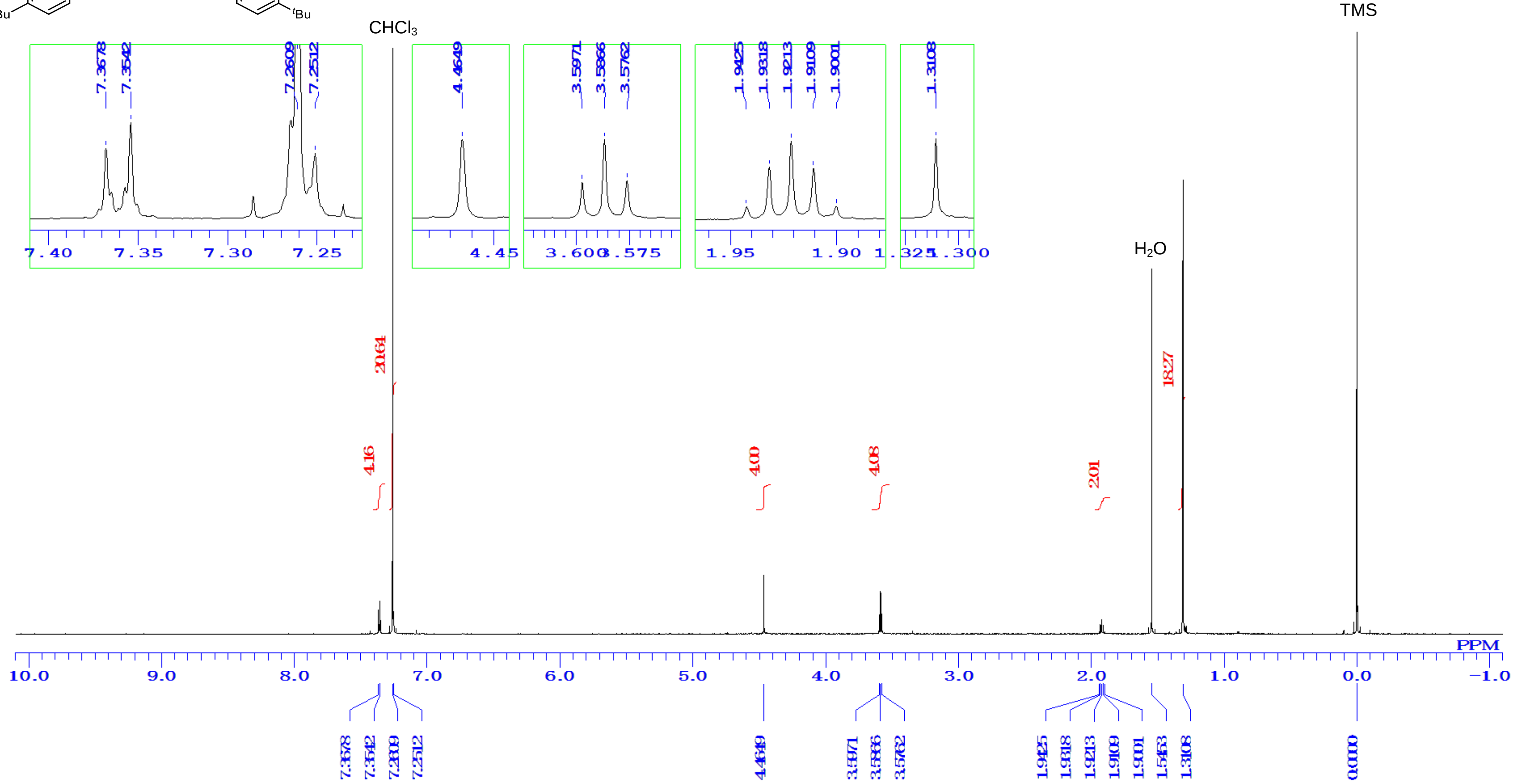

1,3-Bis[(4-*tert*-butylbenzyl)oxy]propane (28)

$^{13}C\{^{1}H\}$ NMR ($CDCl_3$, 150 MHz)

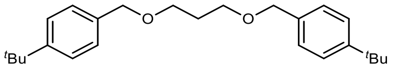

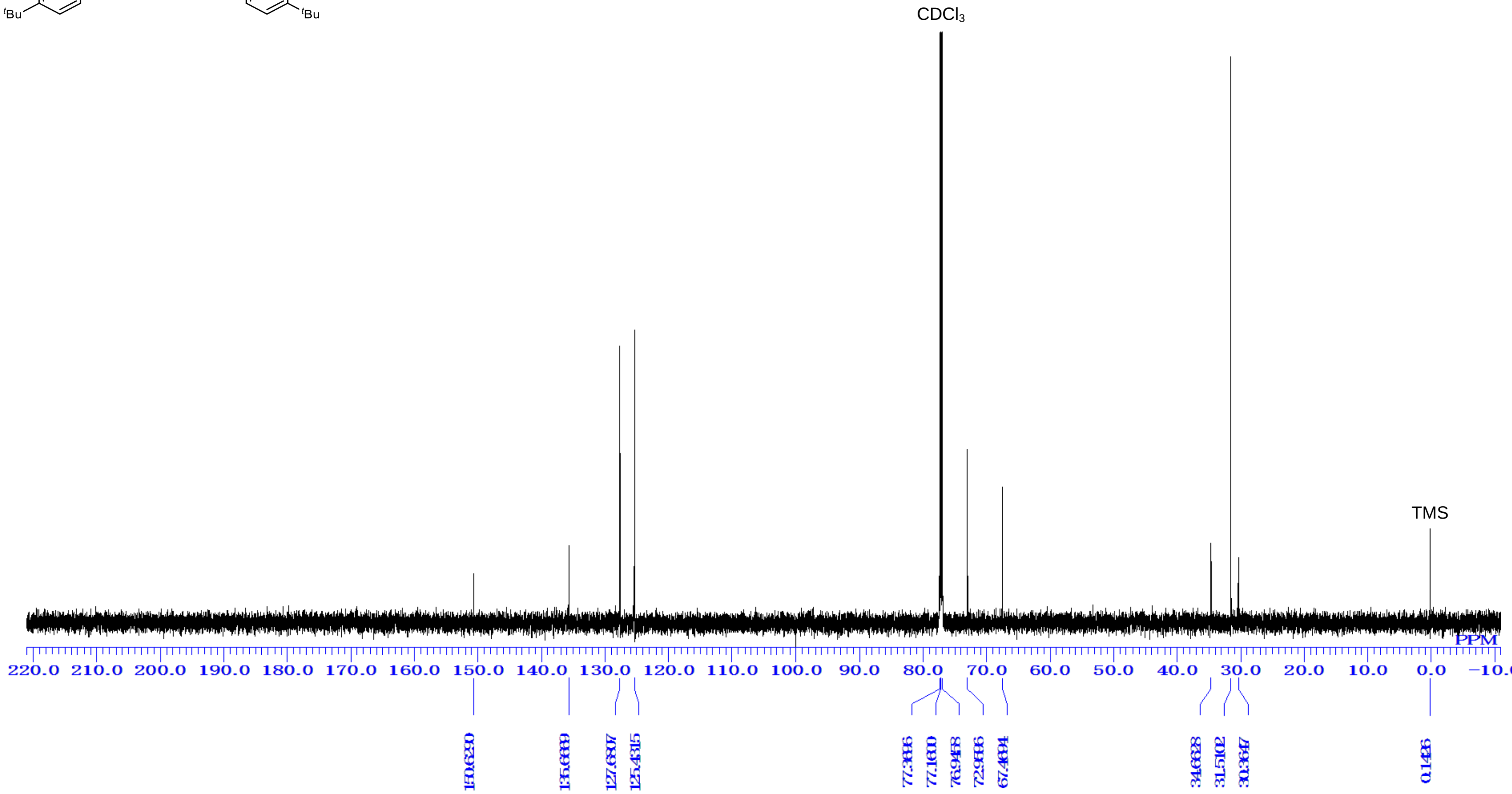

Methyl 3-(4-*tert*-butylphenyl)-2,2-dimethylpropanoate (29)
^{1}H NMR ($CDCl_3$, 600 MHz)

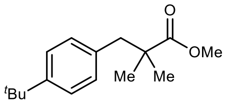

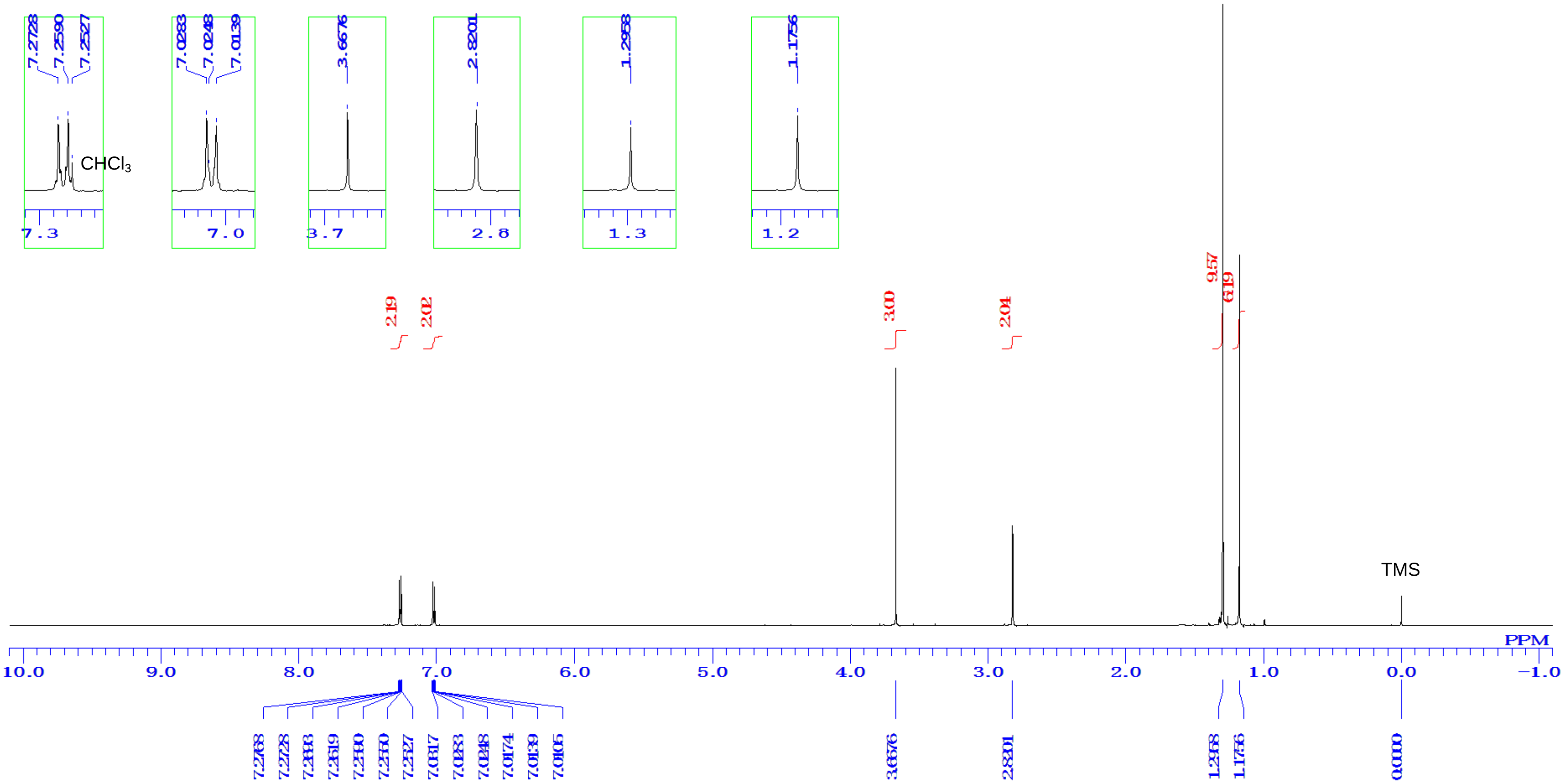

Methyl 3-(4-*tert*-butylphenyl)-2,2-dimethylpropanoate (29)

$^{13}C\{^{1}H\}$ NMR ($CDCl_3$, 150 MHz)

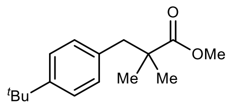

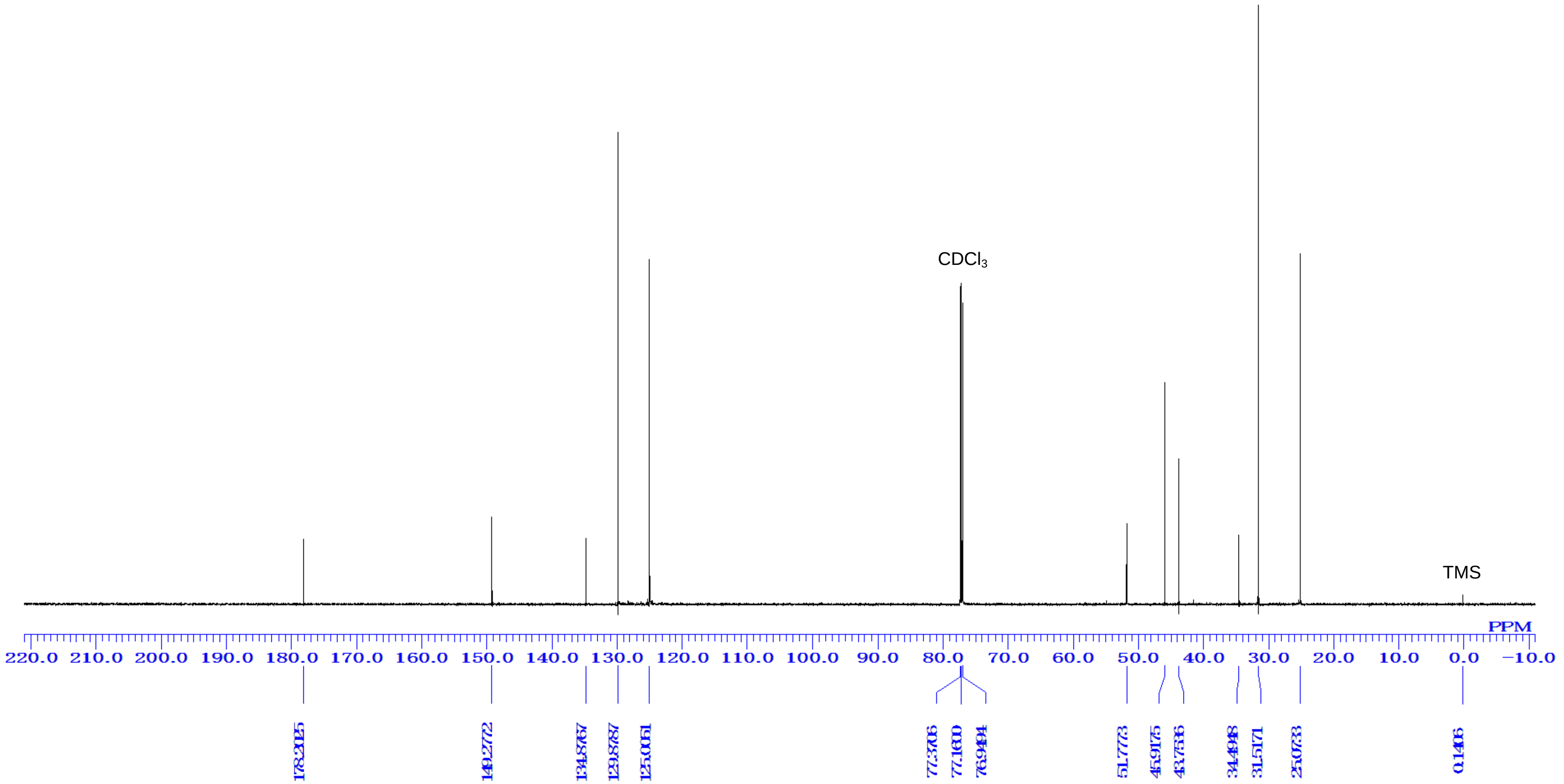

3-(4-*tert*-Butylphenyl)-1-phenylpropan-1-one (30)

^{1}H NMR ($CDCl_3$, 600 MHz)

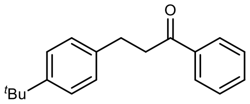

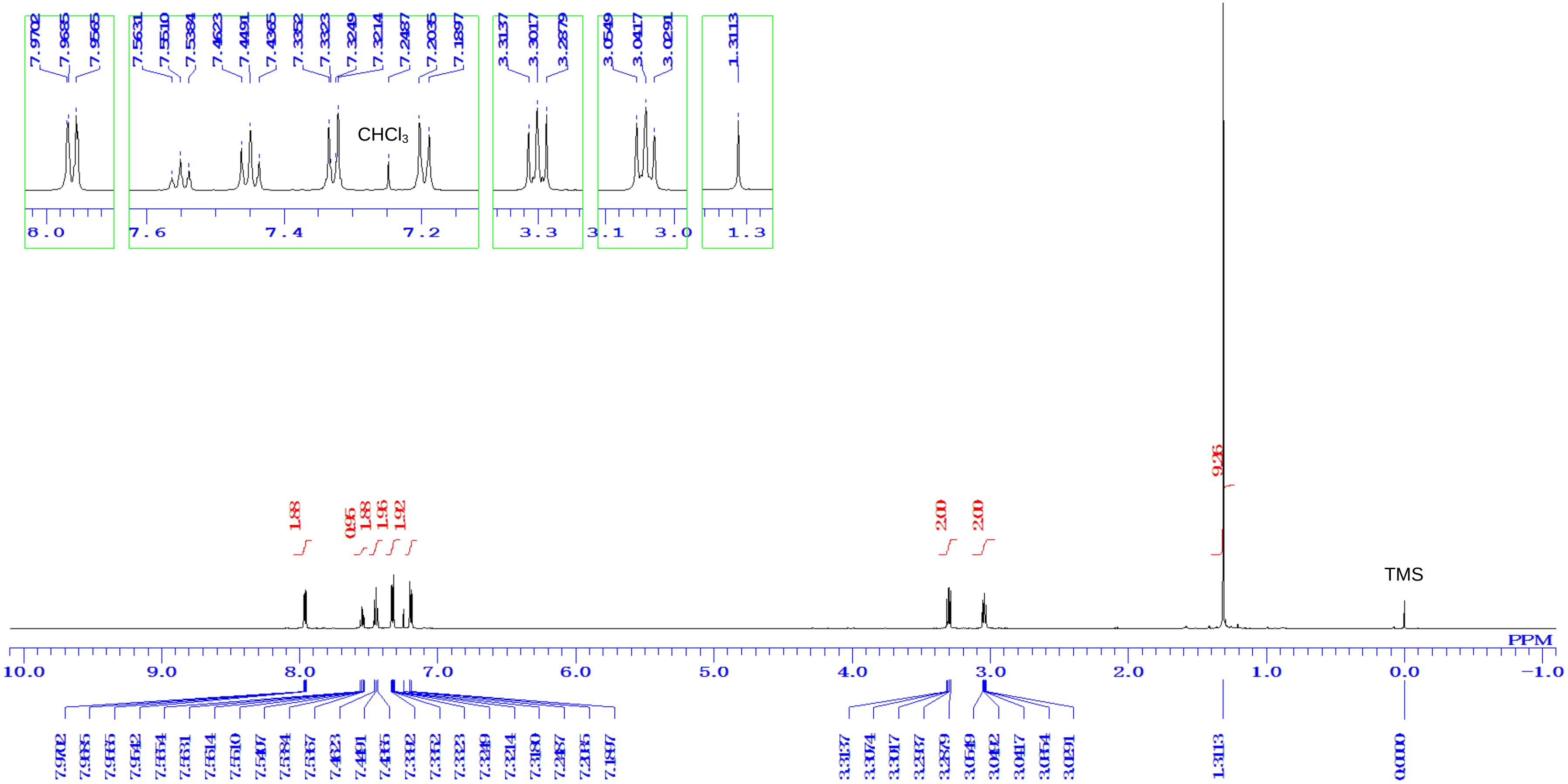

3-(4-*tert*-Butylphenyl)-1-phenylpropan-1-one (30)
$^{13}C\{^{1}H\}$ NMR ($CDCl_3$, 150 MHz)

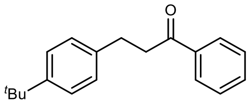

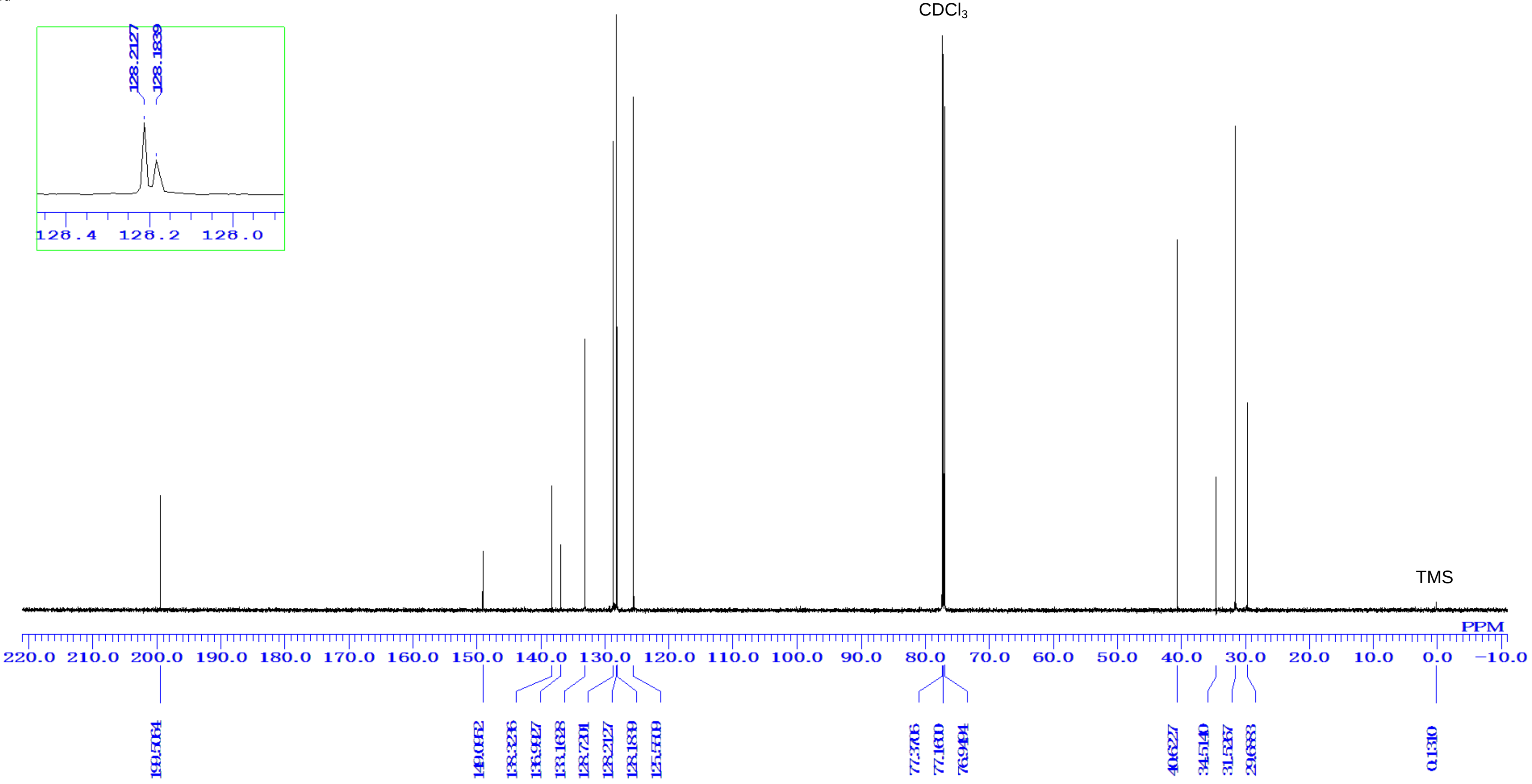

3-(4-*tert*-Butylphenyl)-1-(4-chlorophenyl)propan-1-one (31)

^{1}H NMR ($CDCl_3$, 600 MHz)

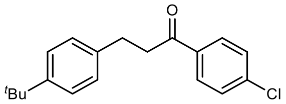

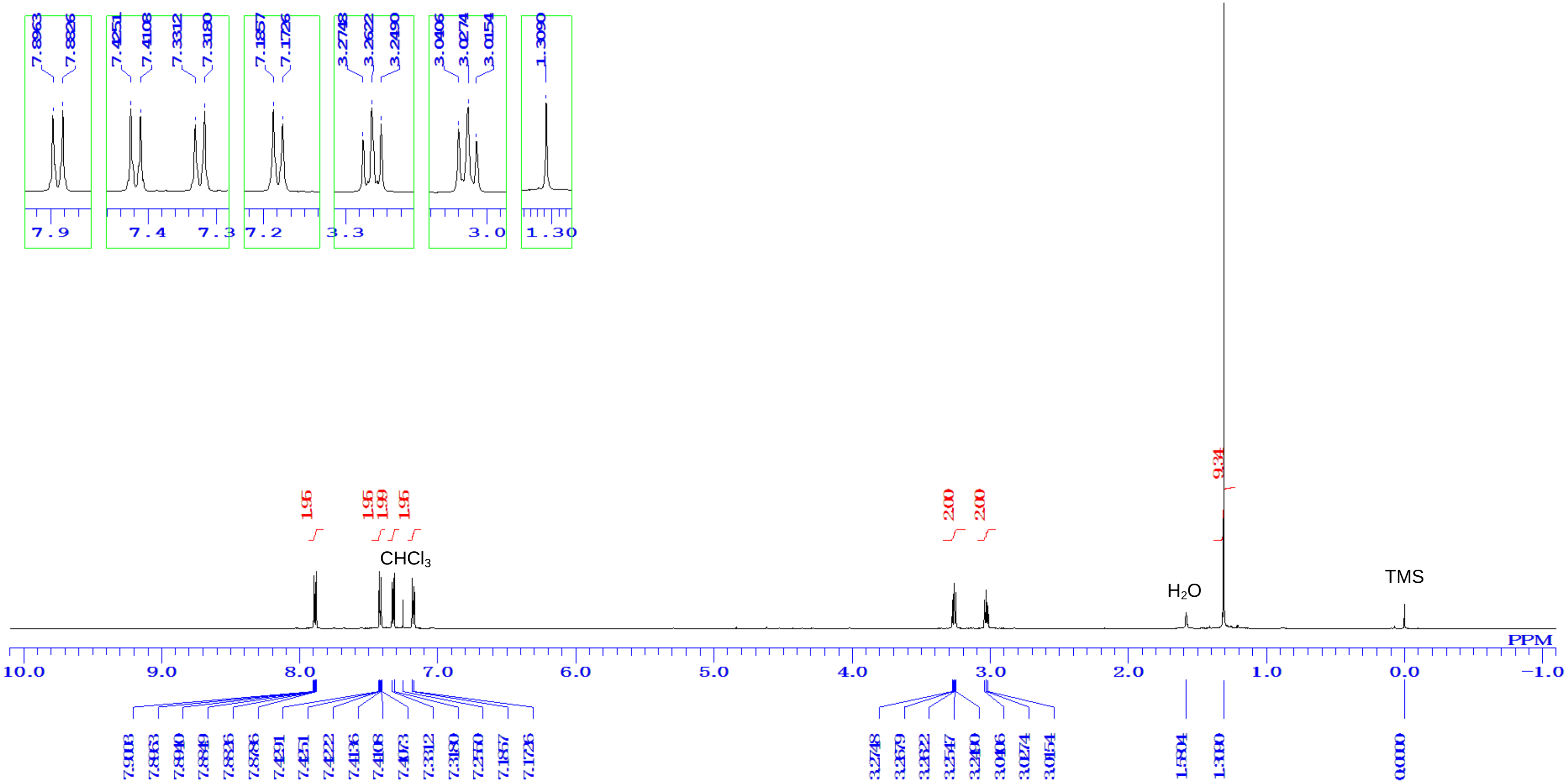

3-(4-*tert*-Butylphenyl)-1-(4-chlorophenyl)propan-1-one (31)

$^{13}C\{^1H\}$ NMR ($CDCl_3$, 150 MHz)

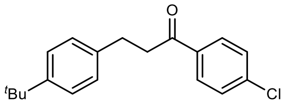

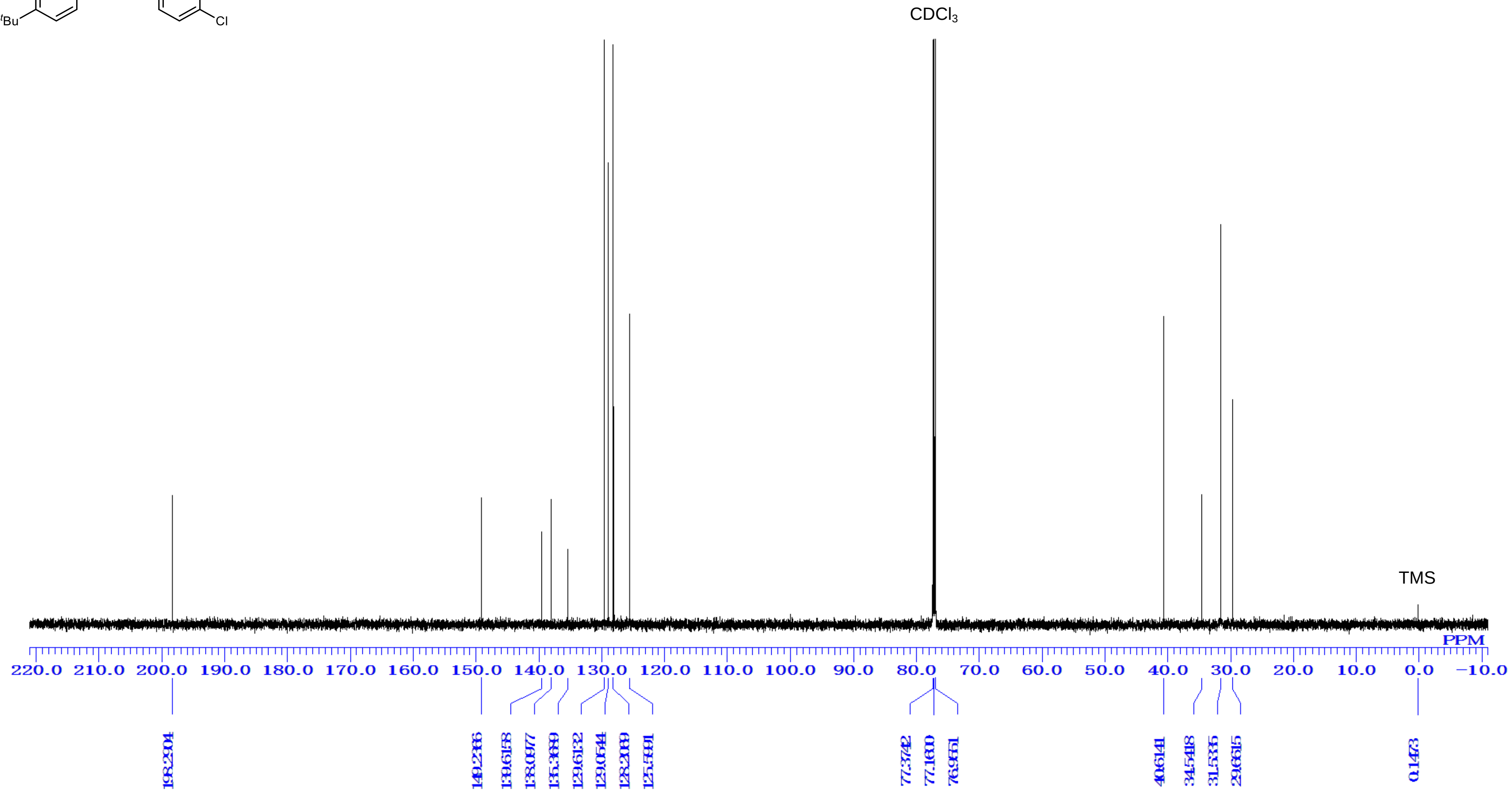

2-(4-Chlorophenyl)-1-(pyrrolidin-1-yl)ethan-1-one (18)

^{1}H NMR ($CDCl_3$, 600 MHz)

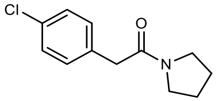

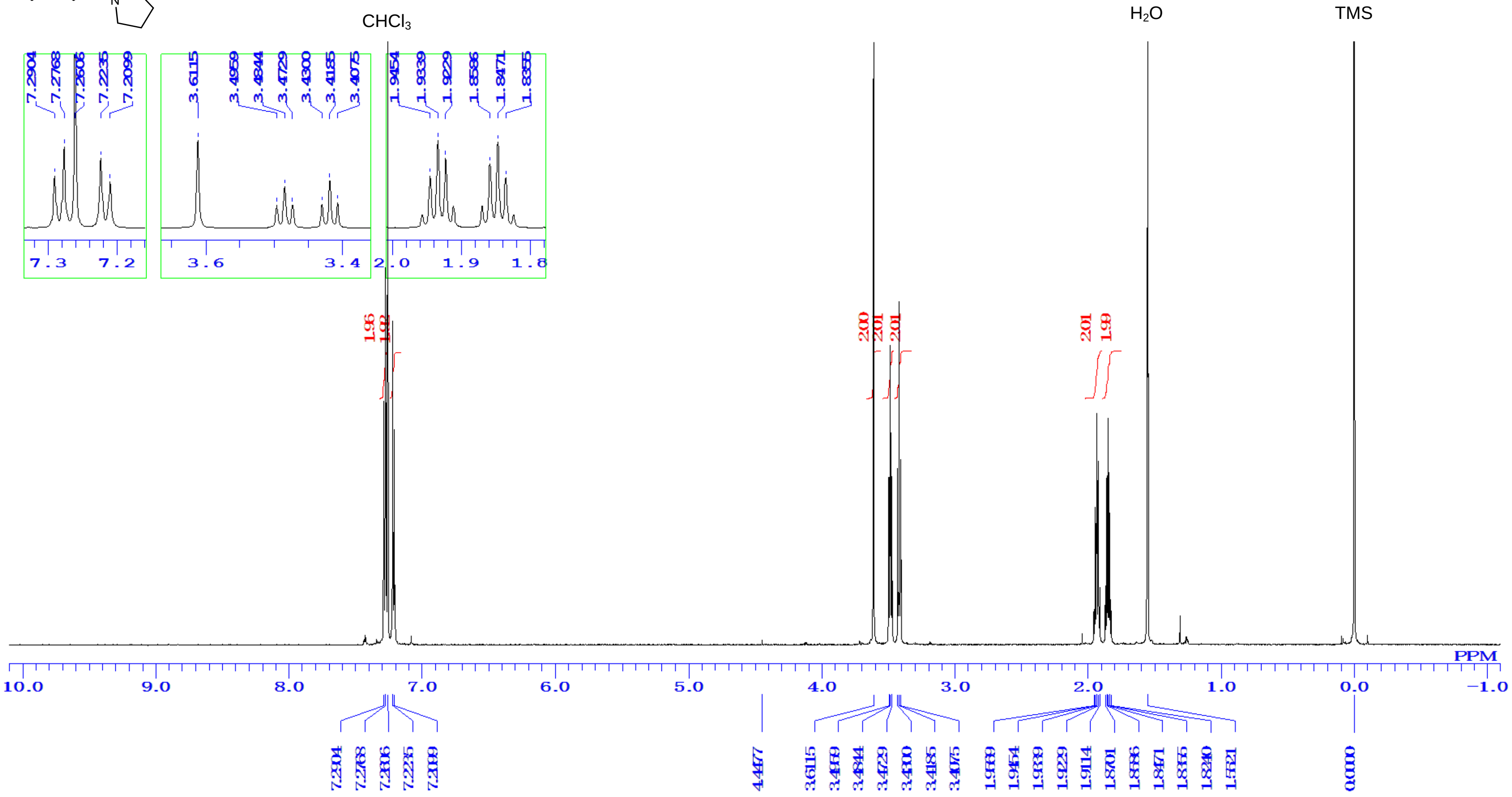

2-(4-Chlorophenyl)-1-(pyrrolidin-1-yl)ethan-1-one (18)

$^{13}C\{^{1}H\}$ NMR ($CDCl_3$, 150 MHz)

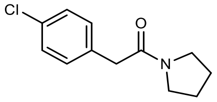

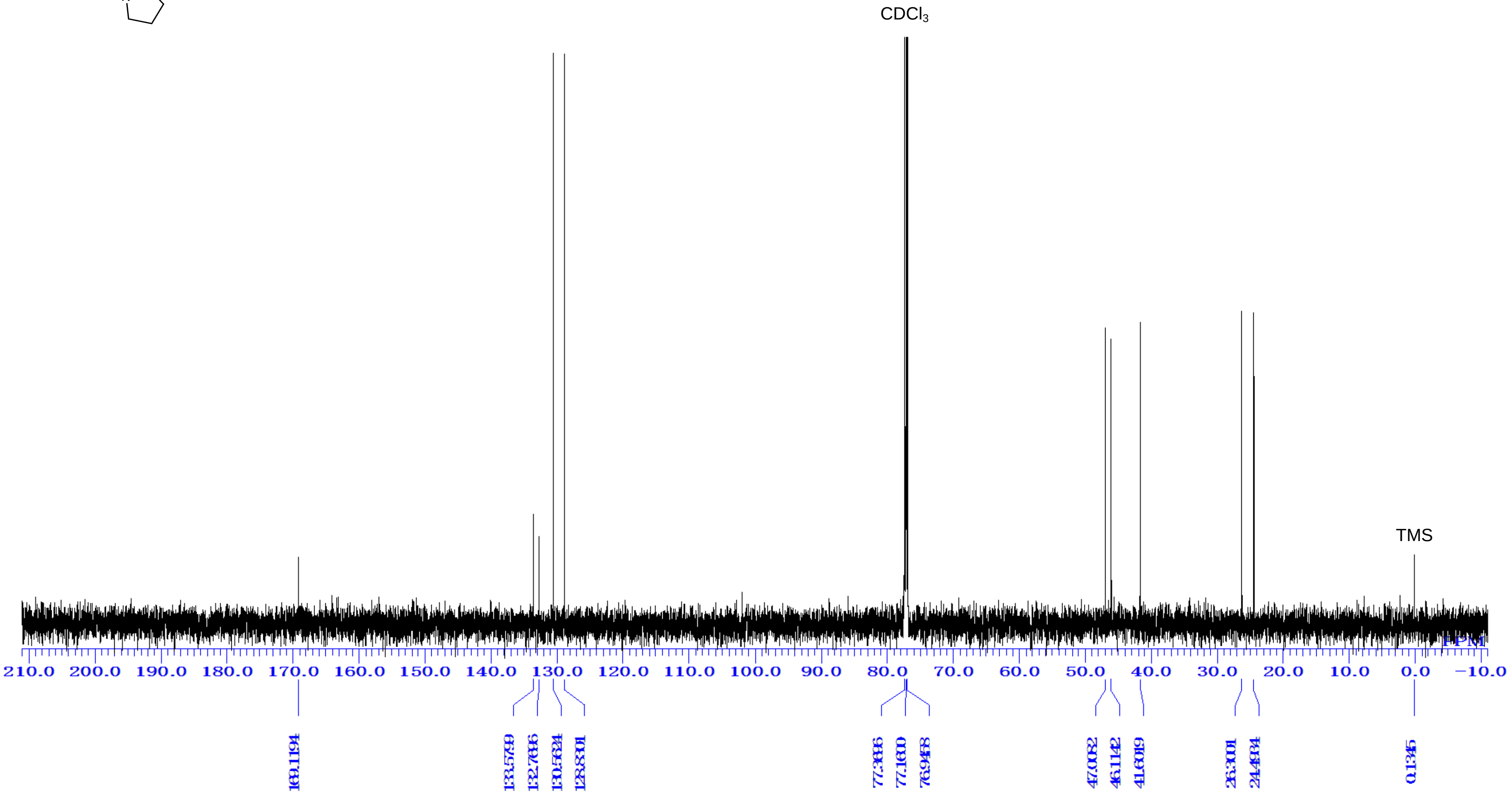

3-(4-*tert*-Butylphenyl)-2-(4-chlorophenyl)-1-(pyrrolidin-1-yl)propan-1-one (32)

^{1}H NMR ($CDCl_3$, 600 MHz)

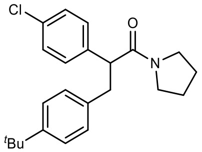

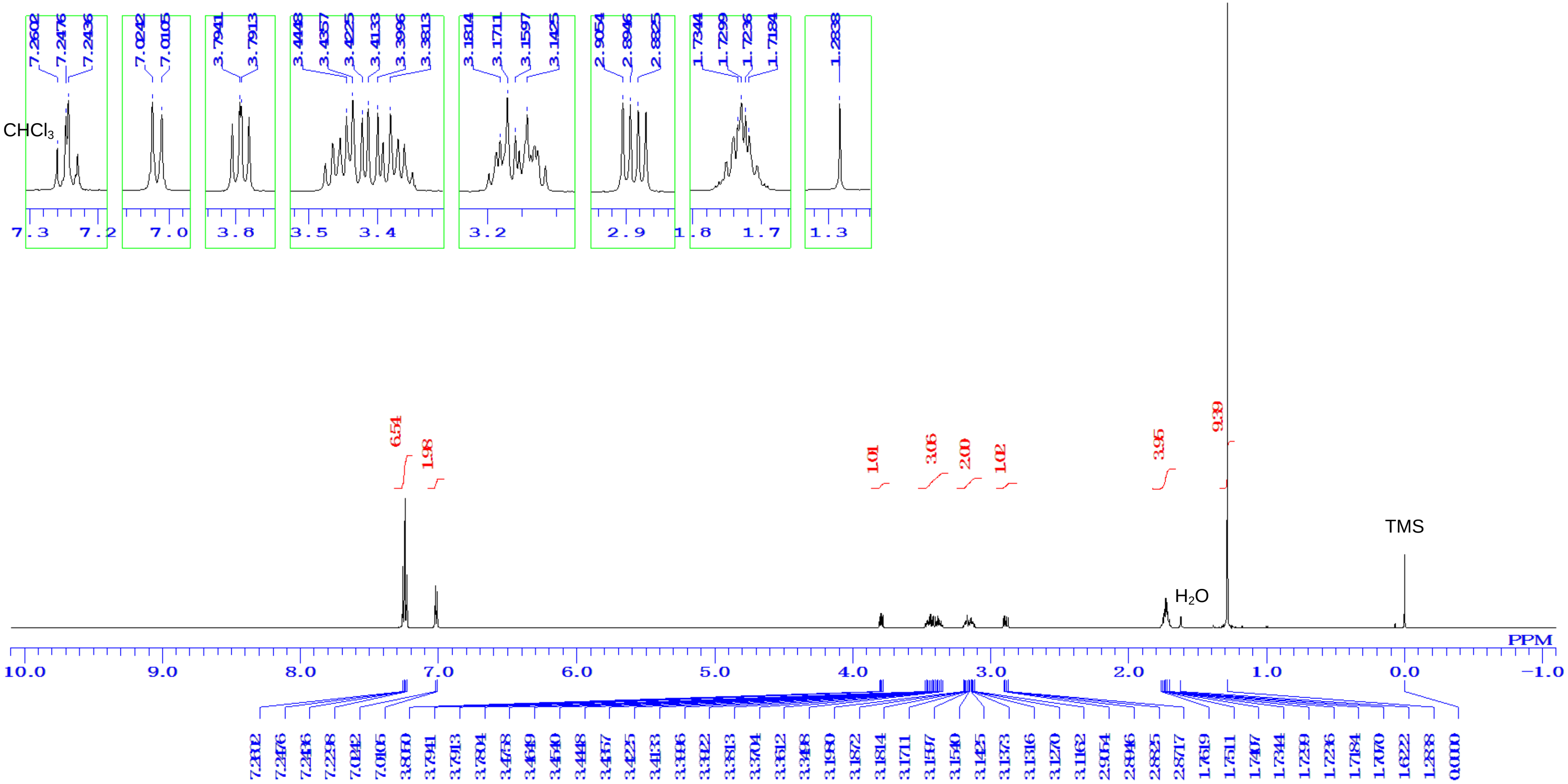

3-(4-*tert*-Butylphenyl)-2-(4-chlorophenyl)-1-(pyrrolidin-1-yl)propan-1-one (32)

$^{13}C\{^{1}H\}$ NMR ($CDCl_3$, 150 MHz)

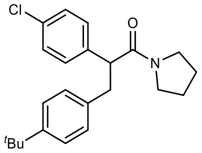

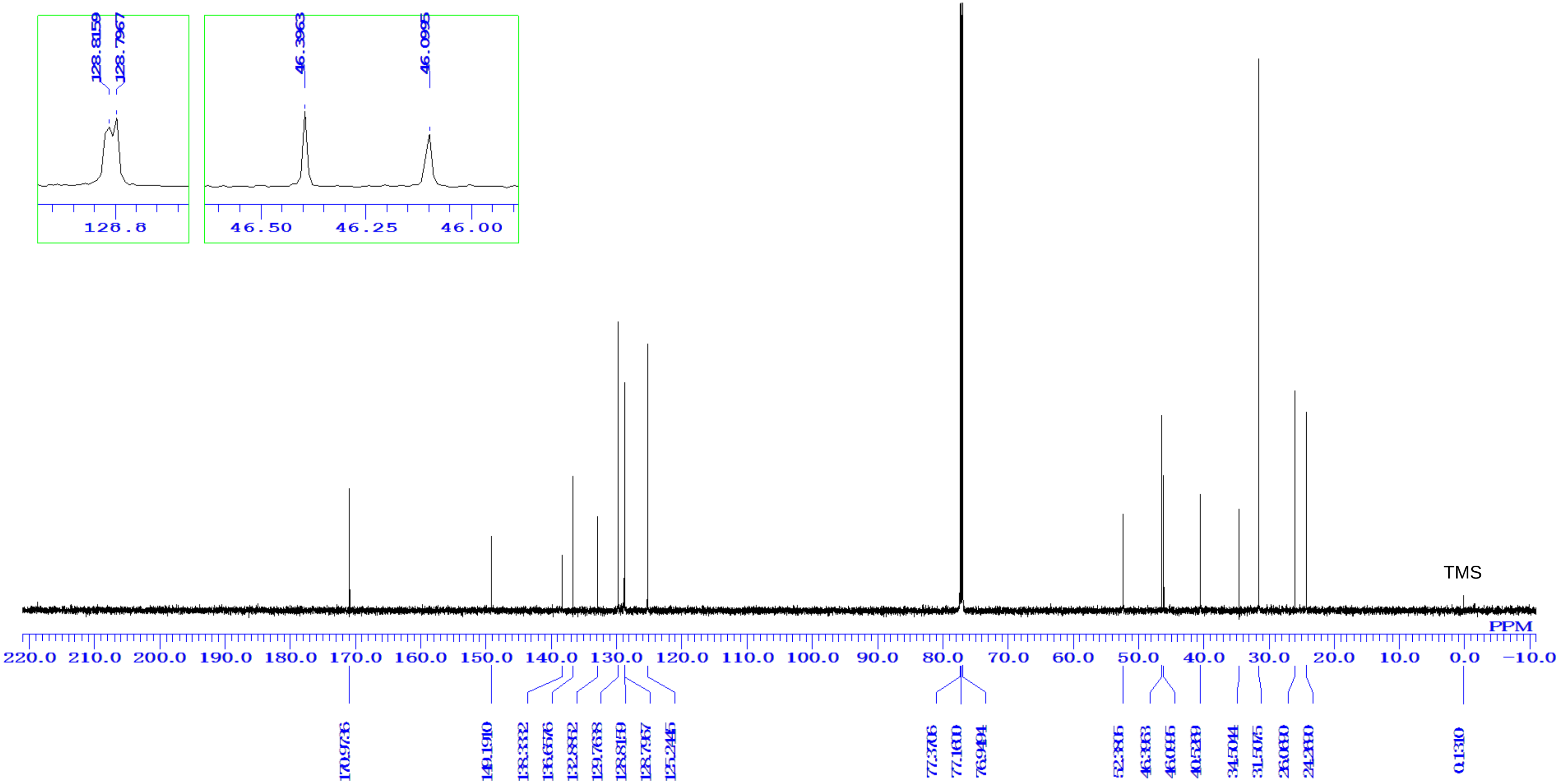

Benzhydryl 4-(*tert*-butyl-2,6-dimethyl)benzyl ether (33)

^1H NMR ($CDCl_3$, 600 MHz)

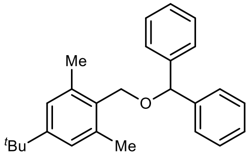

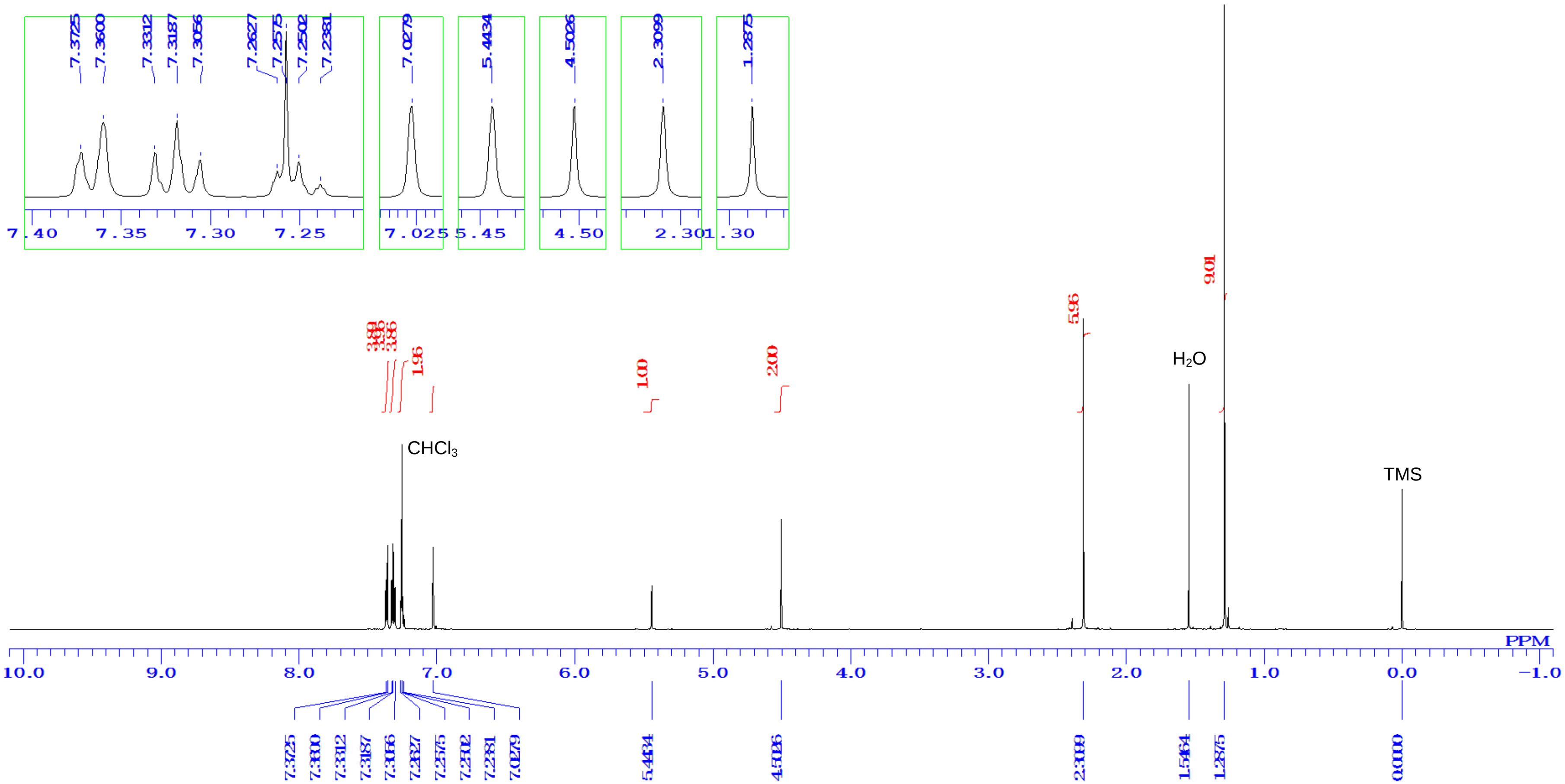

Benzhydryl 4-(*tert*-butyl-2,6-dimethyl)benzyl ether (33)

$^{13}C\{^1H\}$ NMR ($CDCl_3$, 150 MHz)

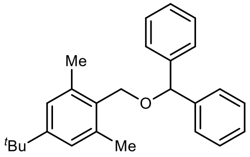

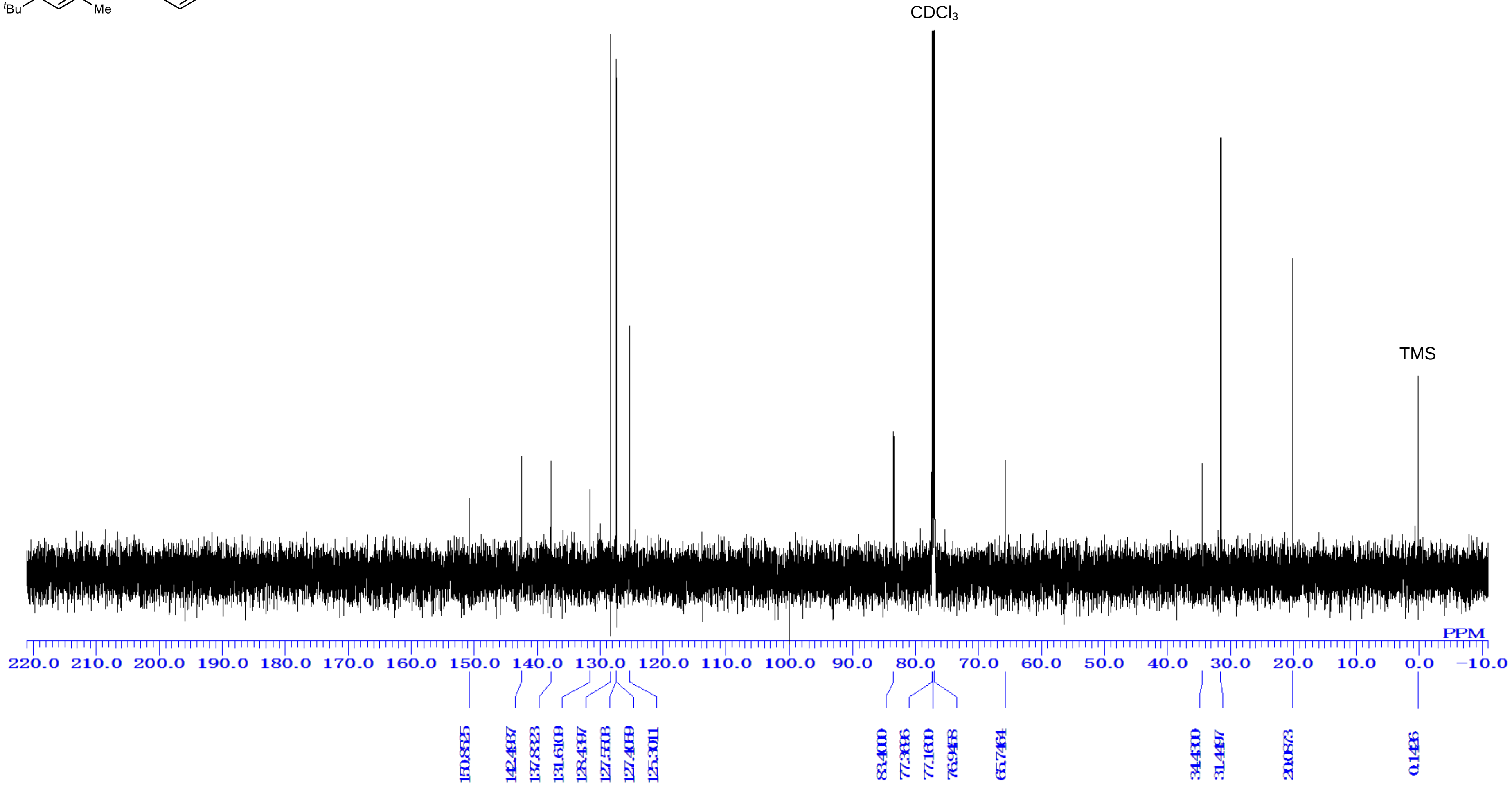

4-(*tert*-Butyl-2,6-dimethyl)benzyl 2-phenylpropan-2-yl ether (34)

^{1}H NMR ($CDCl_3$, 600 MHz)

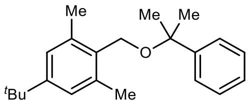

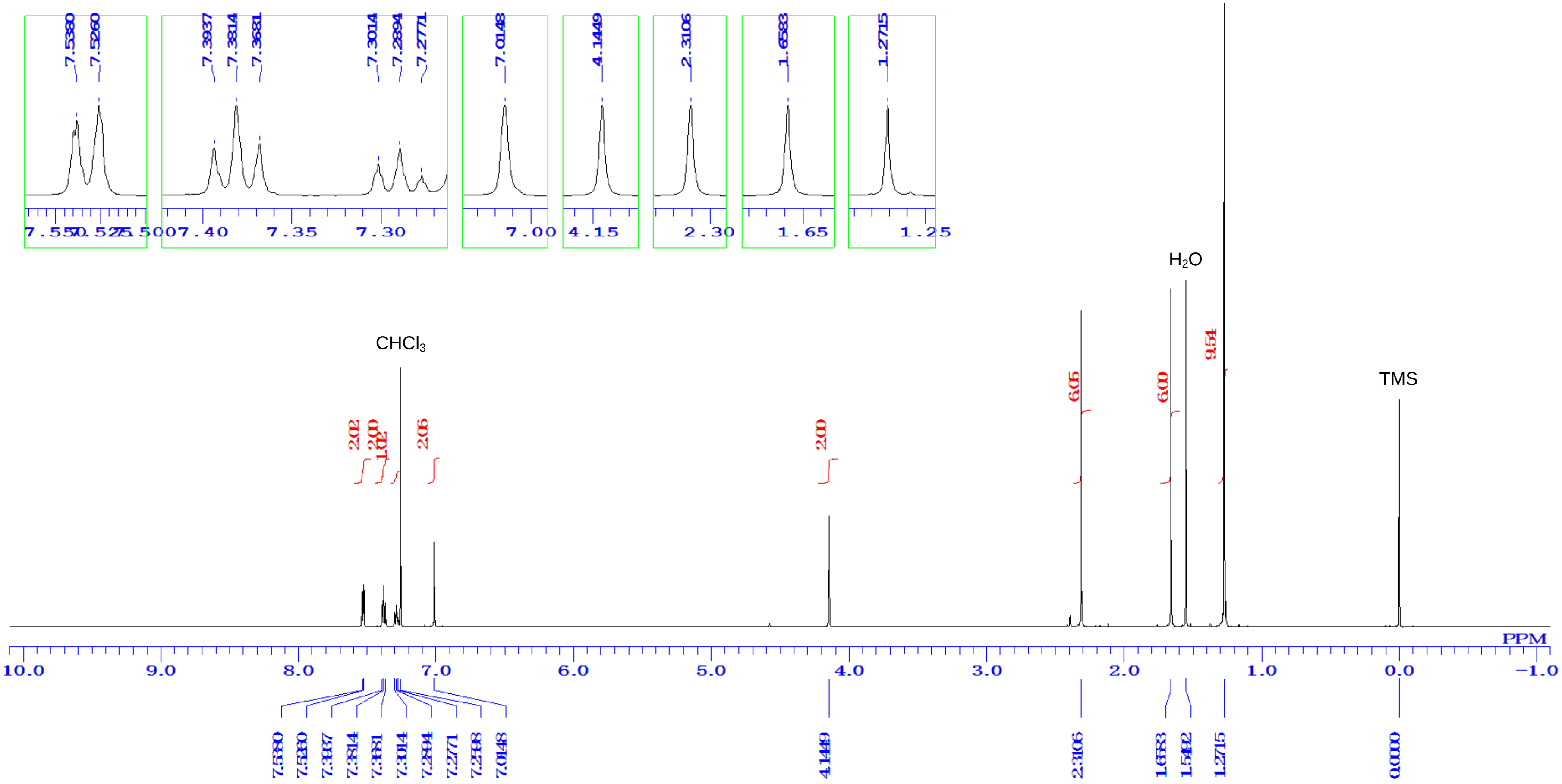

4-(*tert*-Butyl-2,6-dimethyl)benzyl 2-phenylpropan-2-yl ether (34)

$^{13}C\{^{1}H\}$ NMR ($CDCl_3$, 150 MHz)

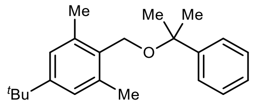

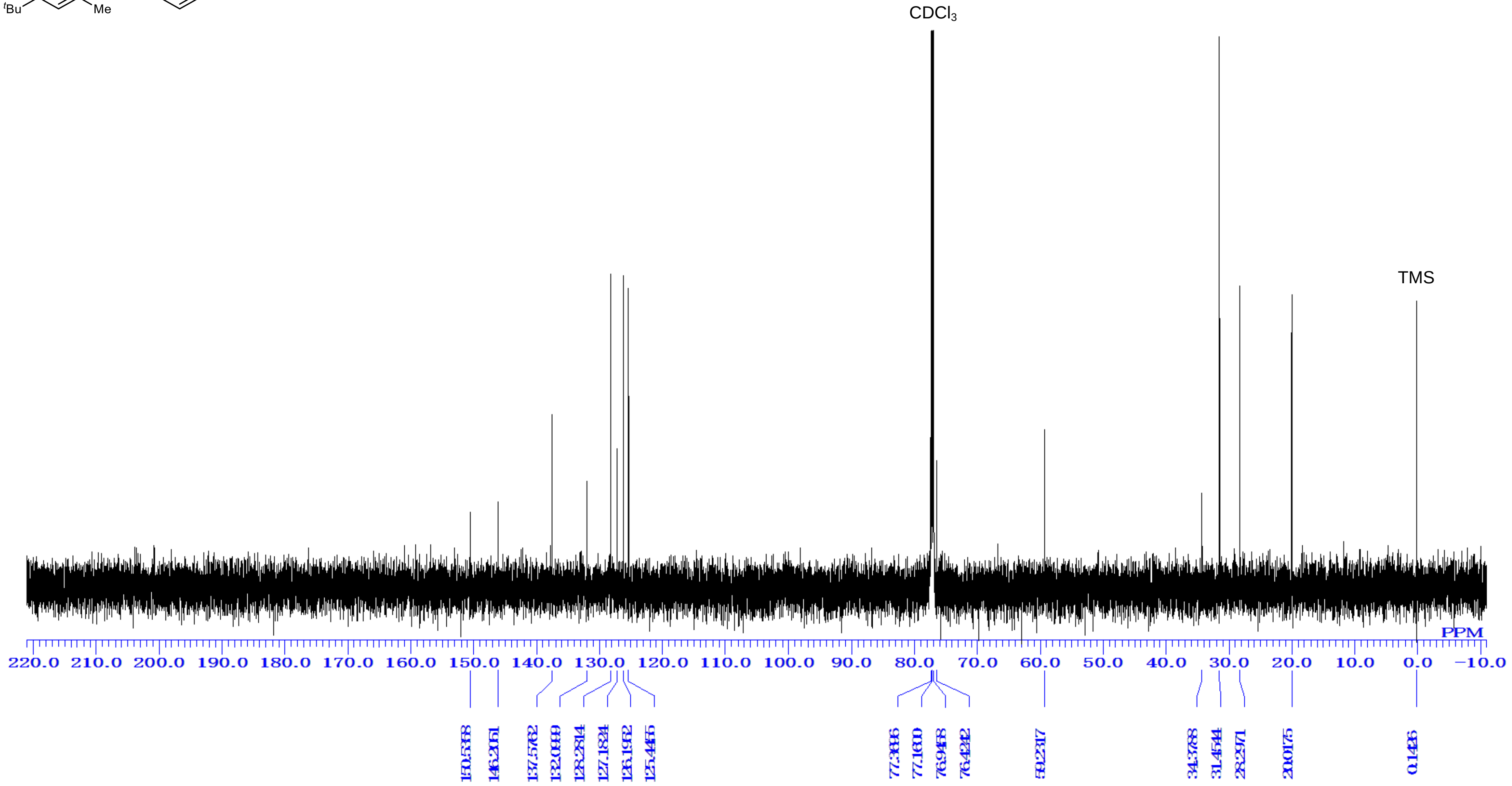

1-Adamantyl 4-(*tert*-butyl-2,6-dimethyl)benzyl ether (35)

^{1}H NMR ($CDCl_3$, 600 MHz)

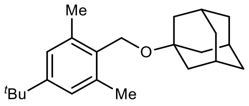

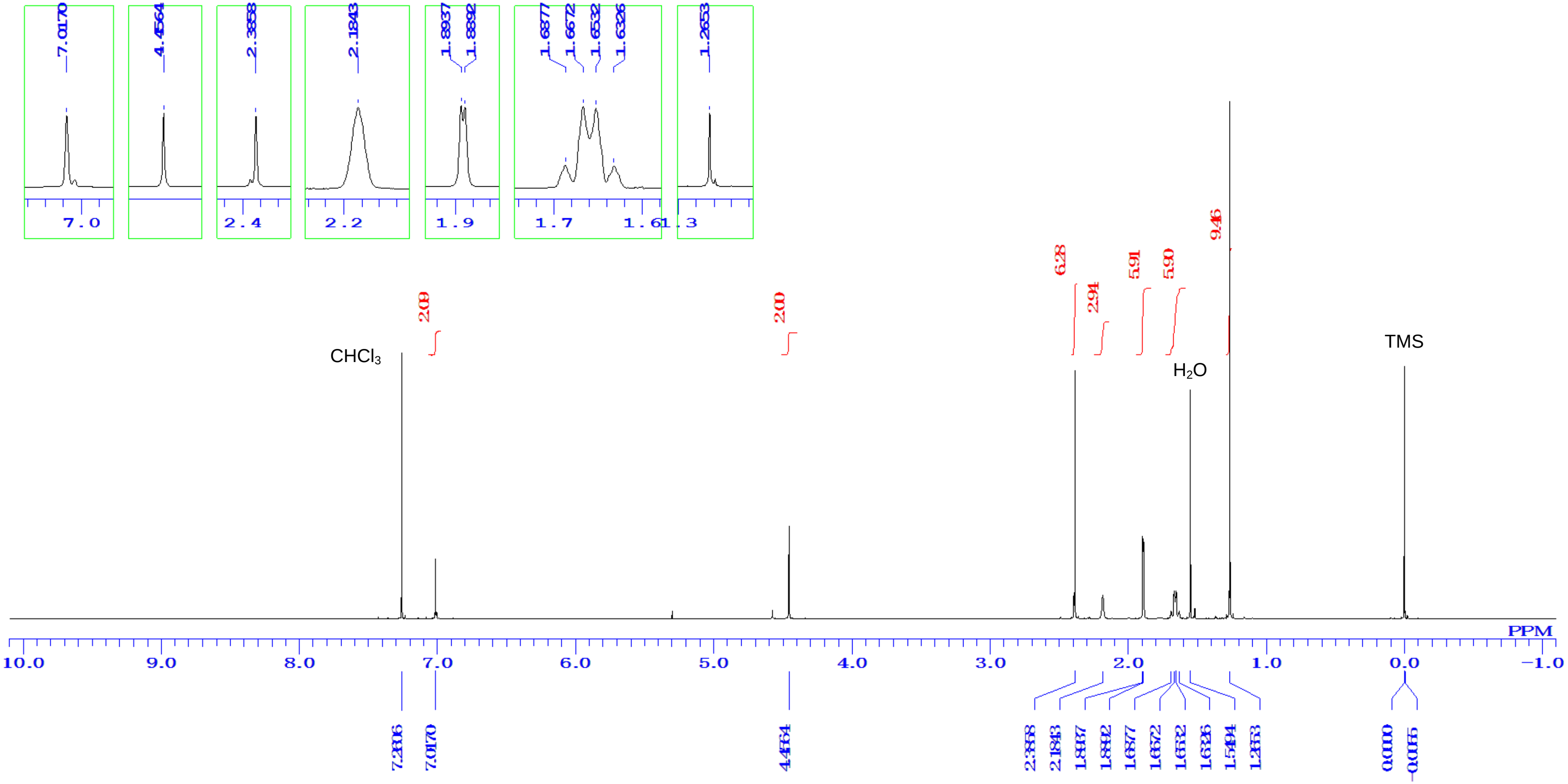

1-Adamantyl 4-(*tert*-butyl-2,6-dimethyl)benzyl ether (35)

$^{13}C\{^{1}H\}$ NMR ($CDCl_3$, 150 MHz)

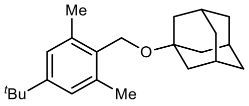

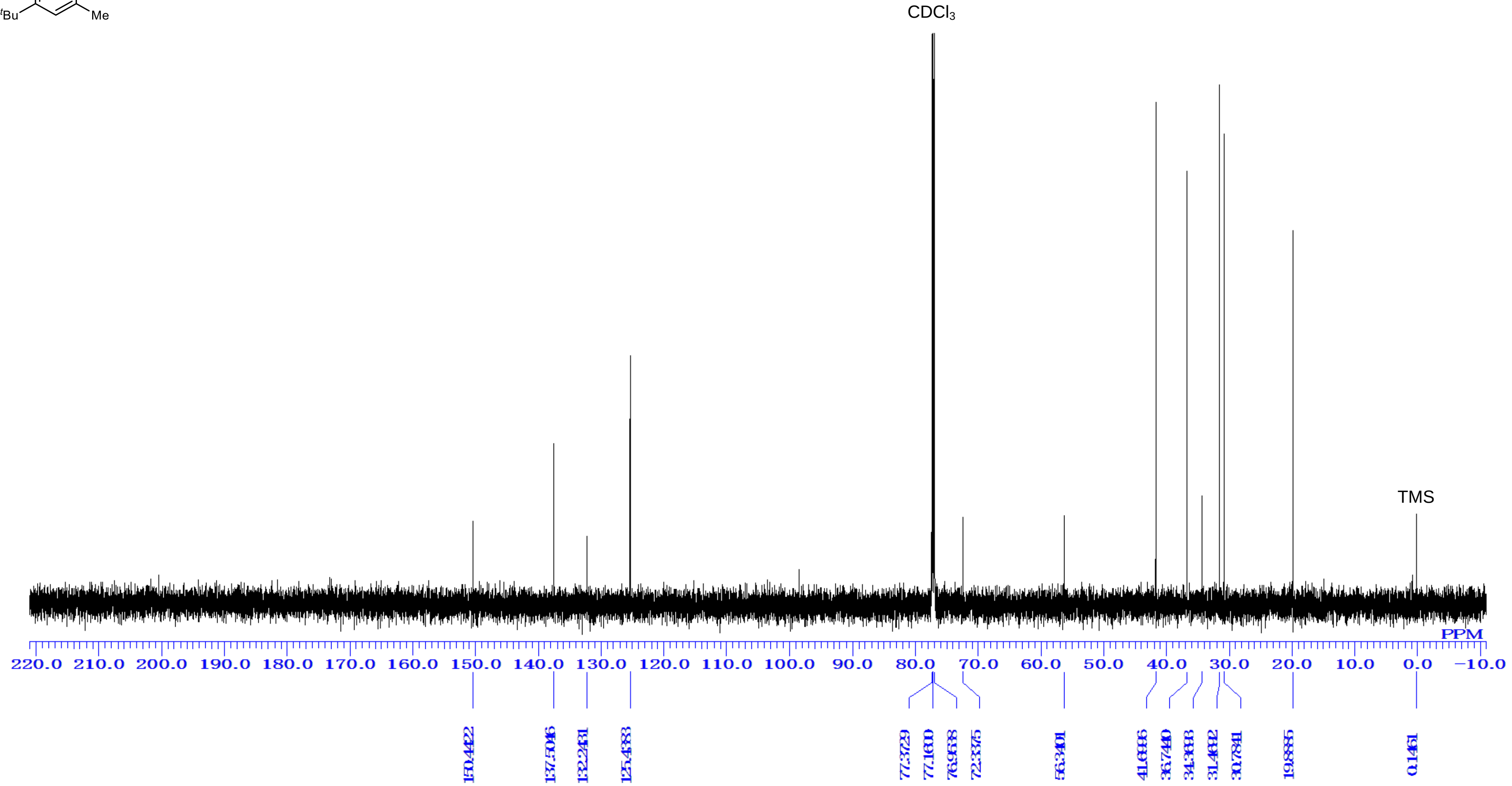

Methyl 3-(4-*tert*-butyl-2,6-dimethylphenyl)-2,2-dimethylpropanoate (36)

^{1}H NMR ($CDCl_3$, 600 MHz)

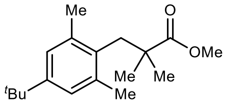

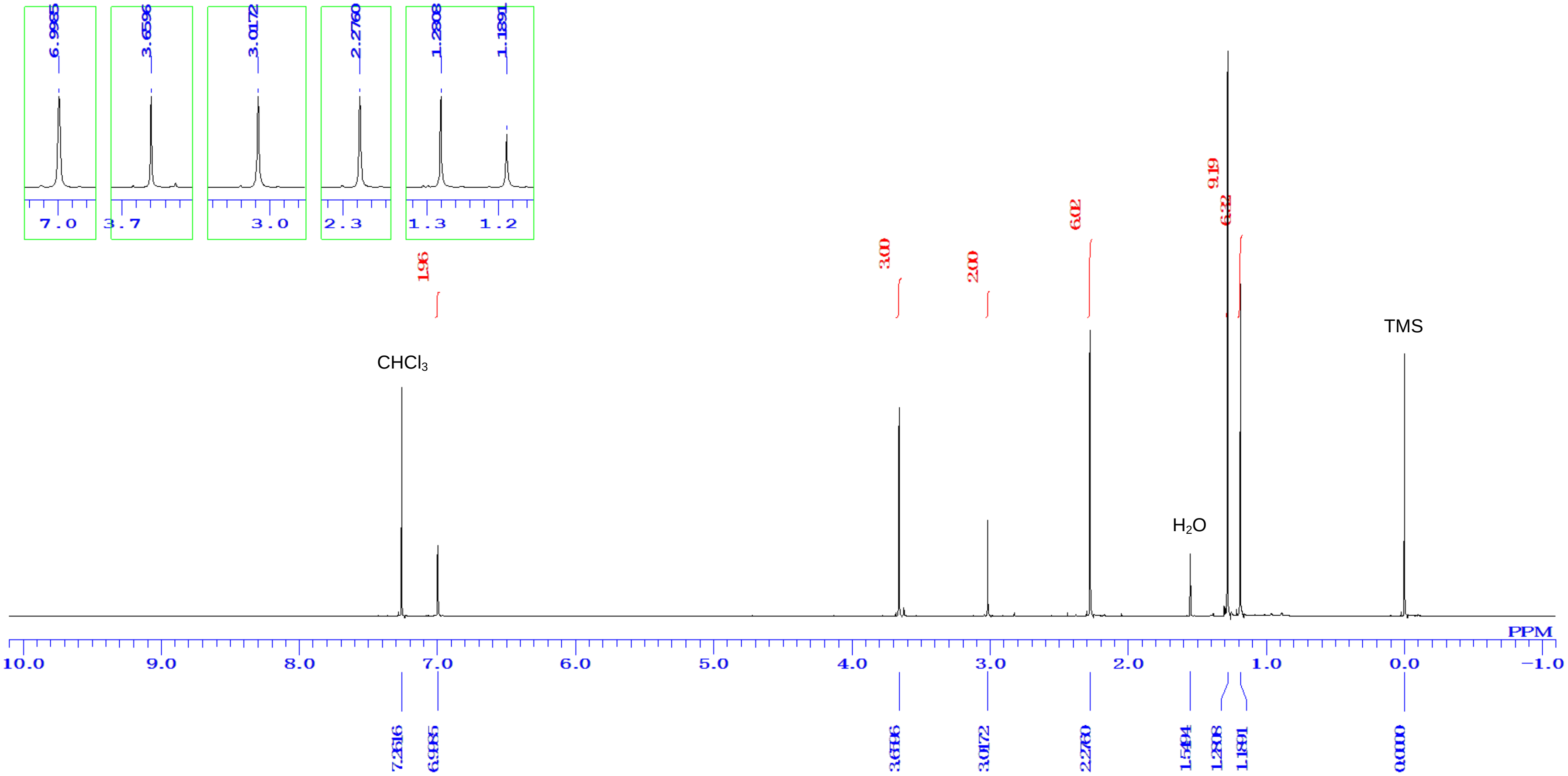

Methyl 3-(4-*tert*-butyl-2,6-dimethylphenyl)-2,2-dimethylpropanoate (36)
$^{13}C\{^{1}H\}$ NMR ($CDCl_3$, 150 MHz)

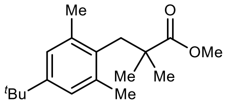

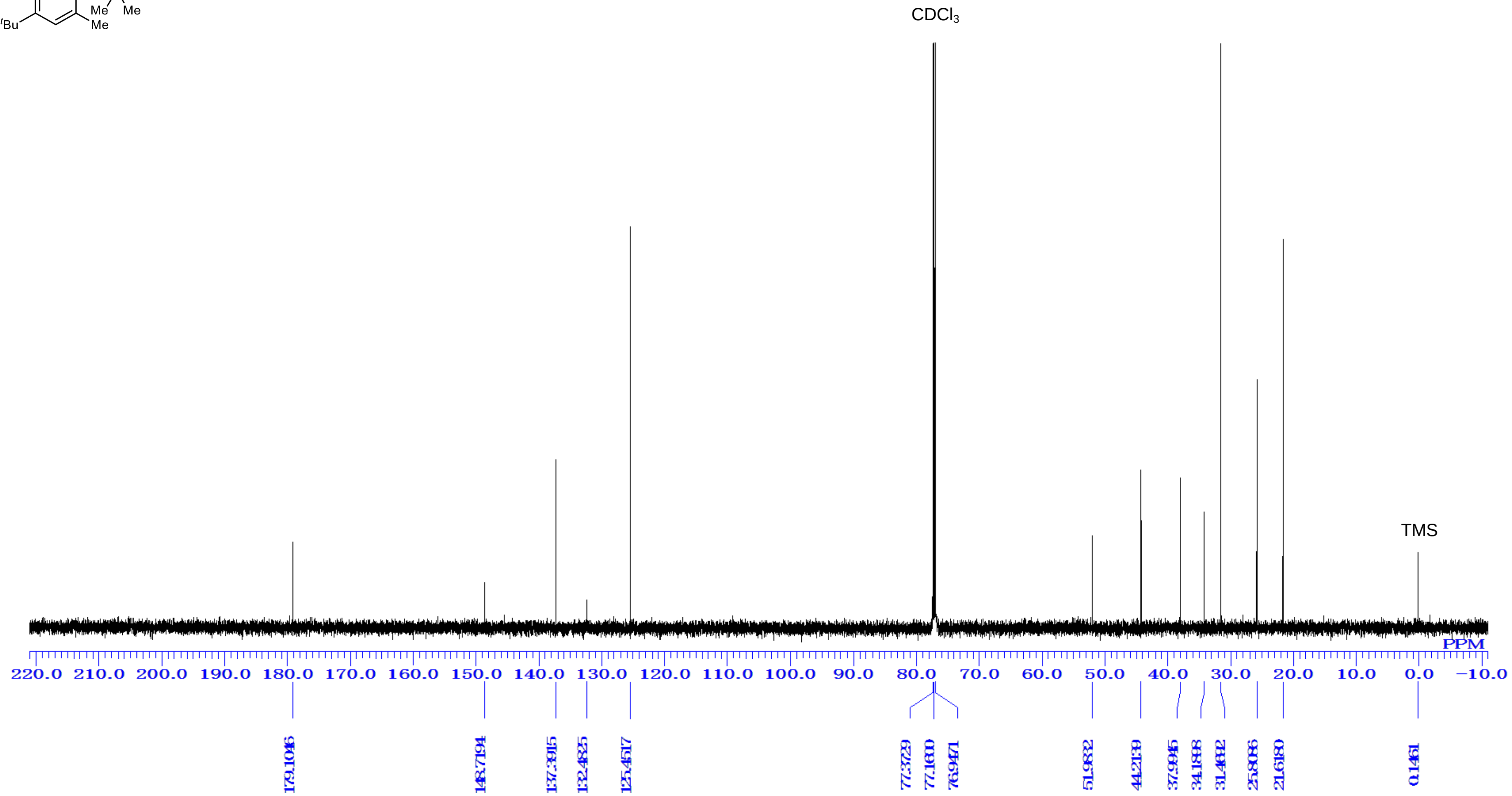

3-(4-*tert*-Butyl-2,6-dimethylphenyl)-1-phenylpropan-1-one (37)

^{1}H NMR ($CDCl_3$, 600 MHz)

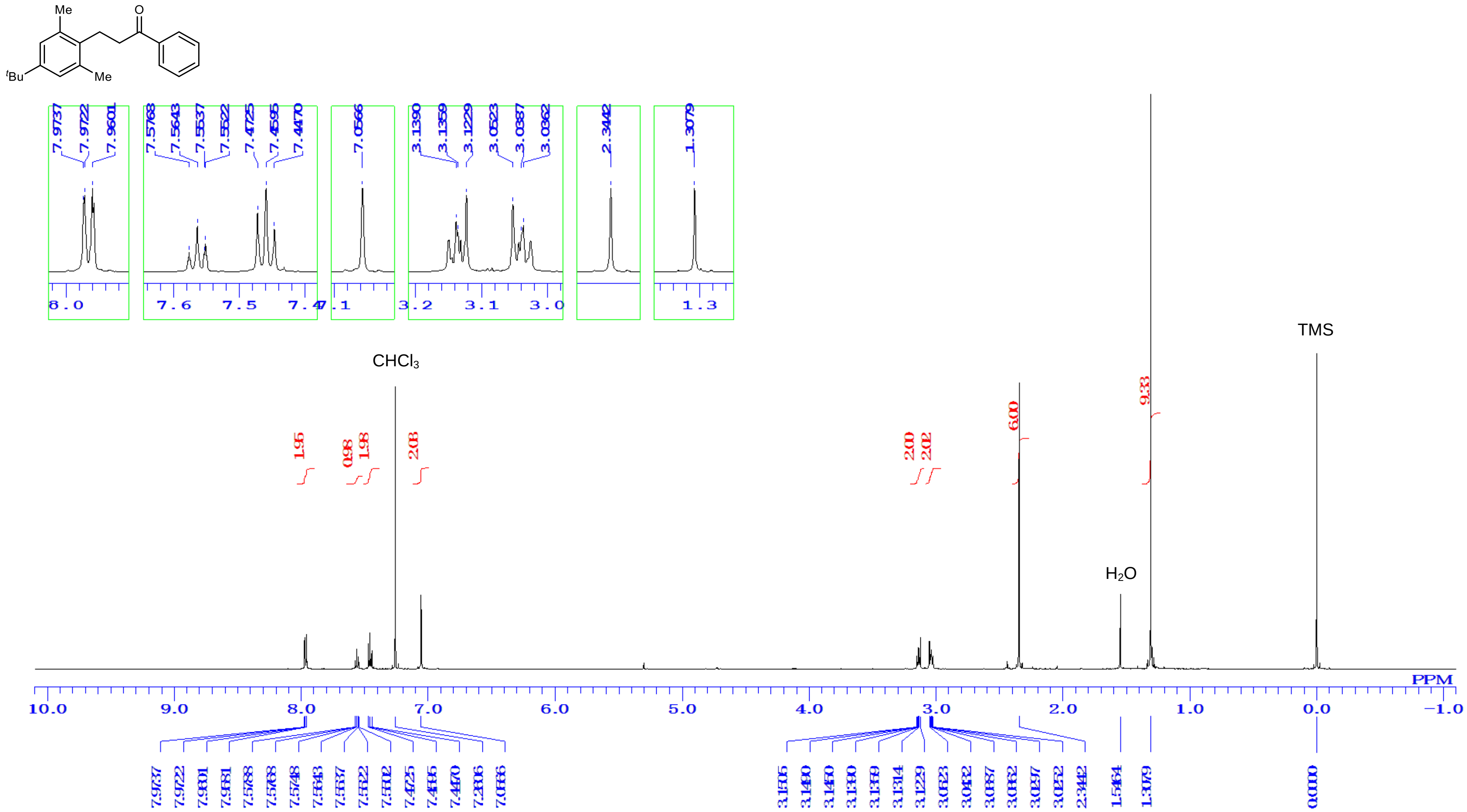

3-(4-*tert*-Butyl-2,6-dimethylphenyl)-1-phenylpropan-1-one (37)

$^{13}C\{^{1}H\}$ NMR ($CDCl_3$, 150 MHz)

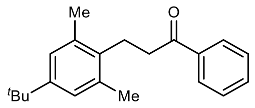

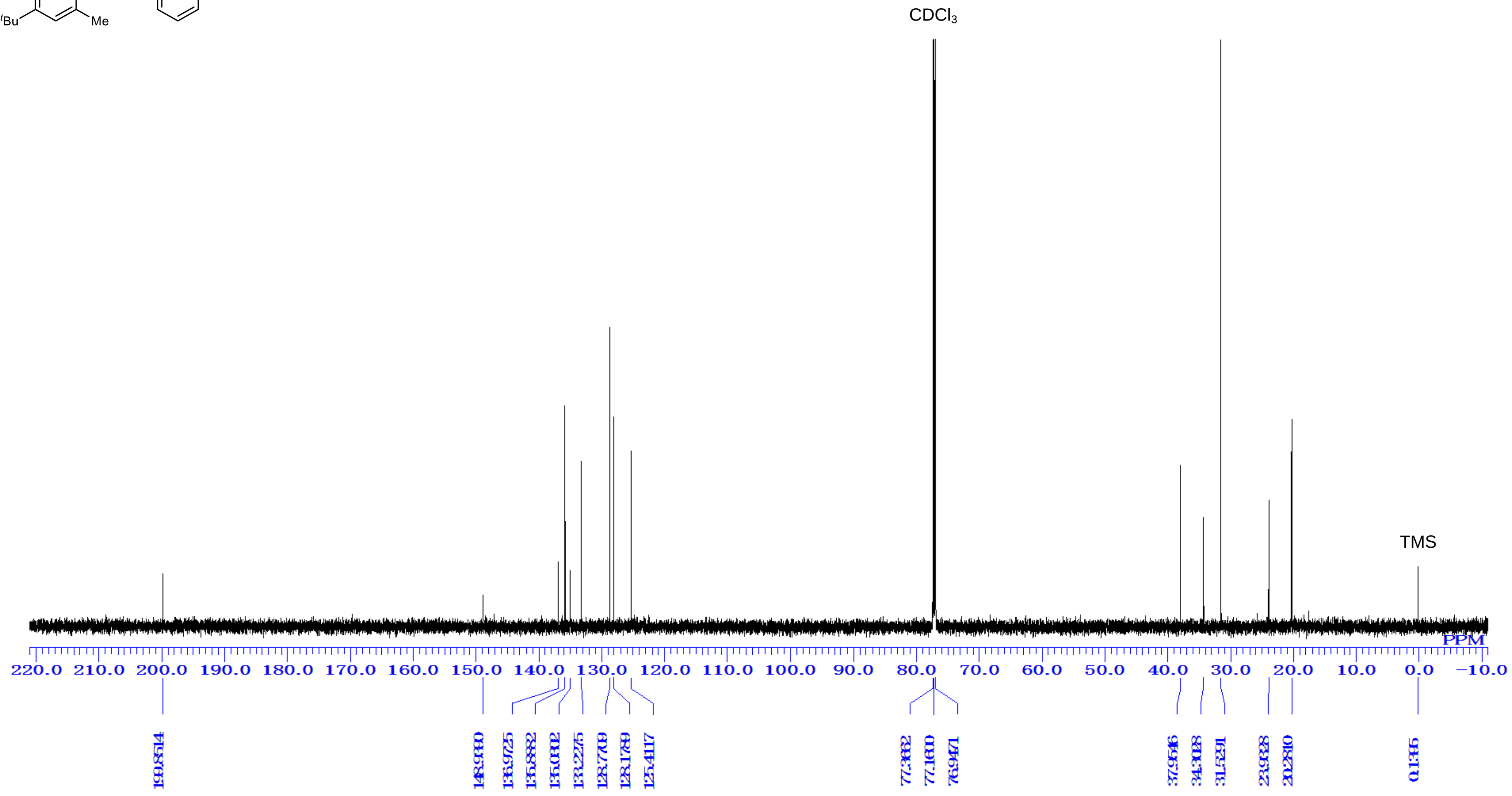

2-(4-*tert*-Butyl-2,6-dimethylbenzyl)cyclohexan-1-one (38)

^{1}H NMR ($CDCl_3$, 600 MHz)

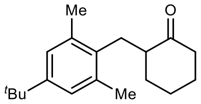

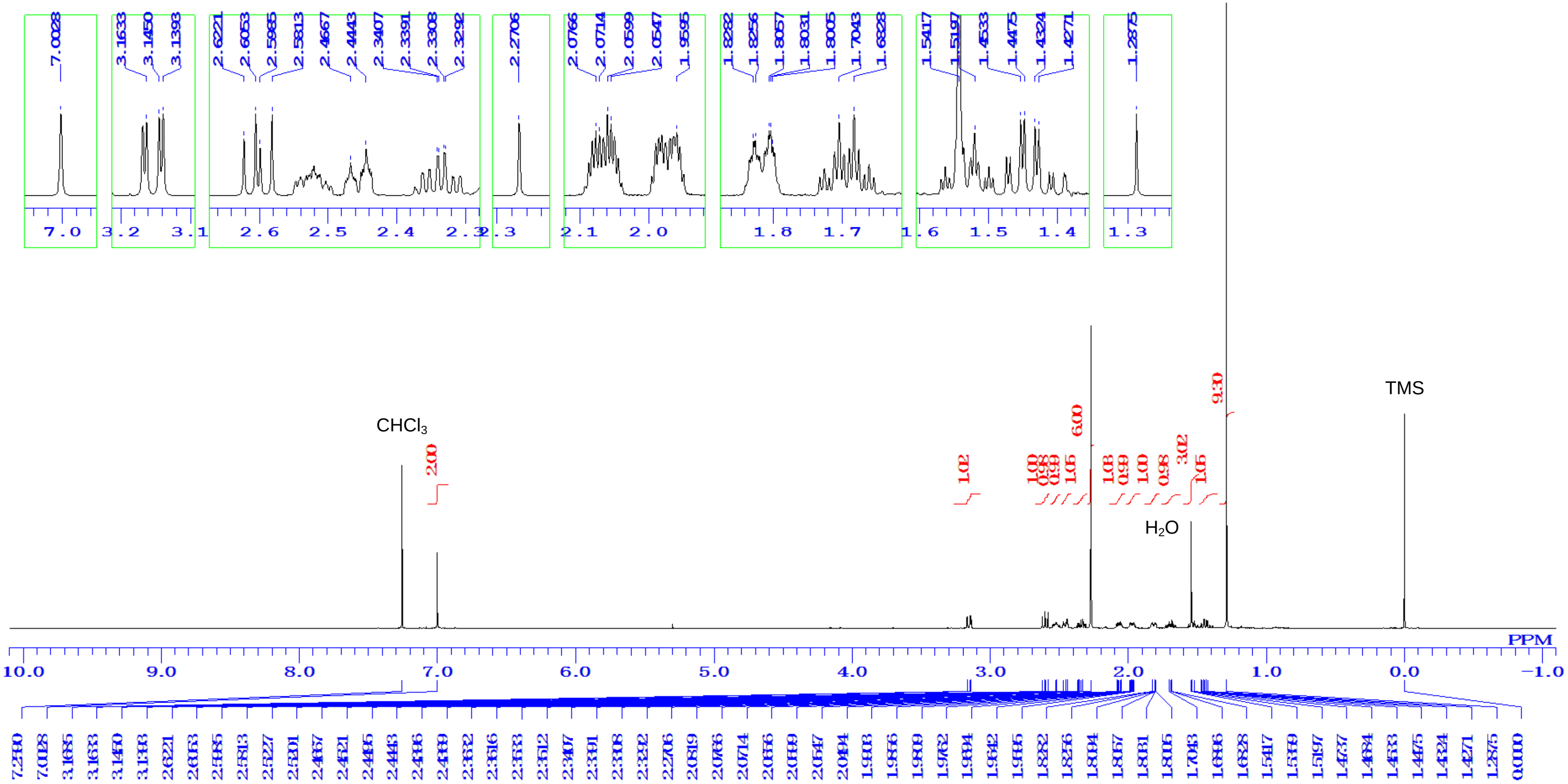

2-(4-*tert*-Butyl-2,6-dimethylbenzyl)cyclohexan-1-one (38)

$^{13}C\{^1H\}$ NMR ($CDCl_3$, 150 MHz)

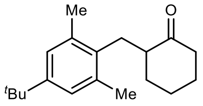

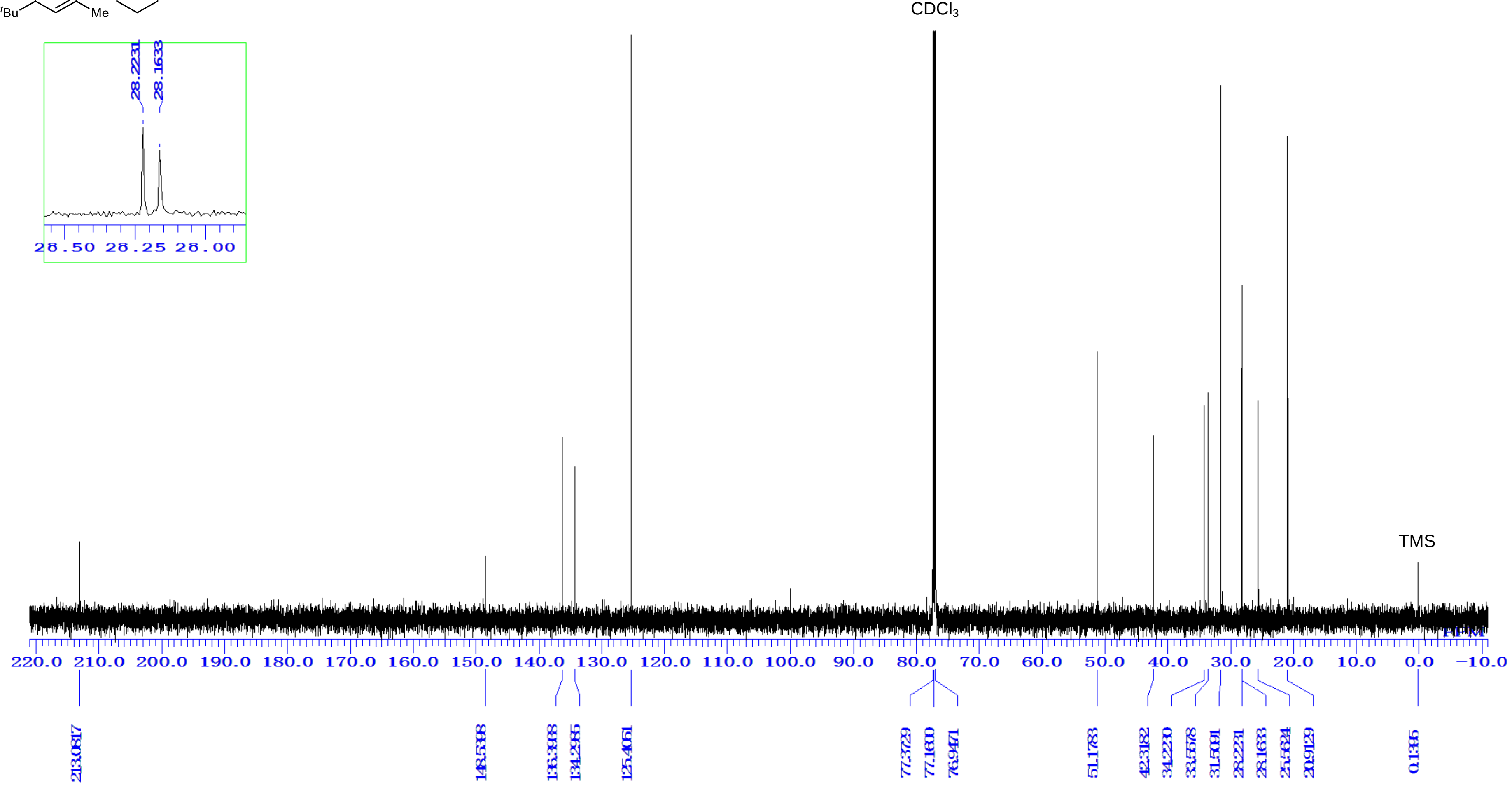

2-(But-3-en-1-yl)-5-*tert*-butyl-1,3-dimethylbenzene (39)
^{1}H NMR ($CDCl_3$, 600 MHz)

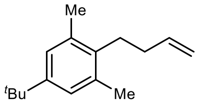

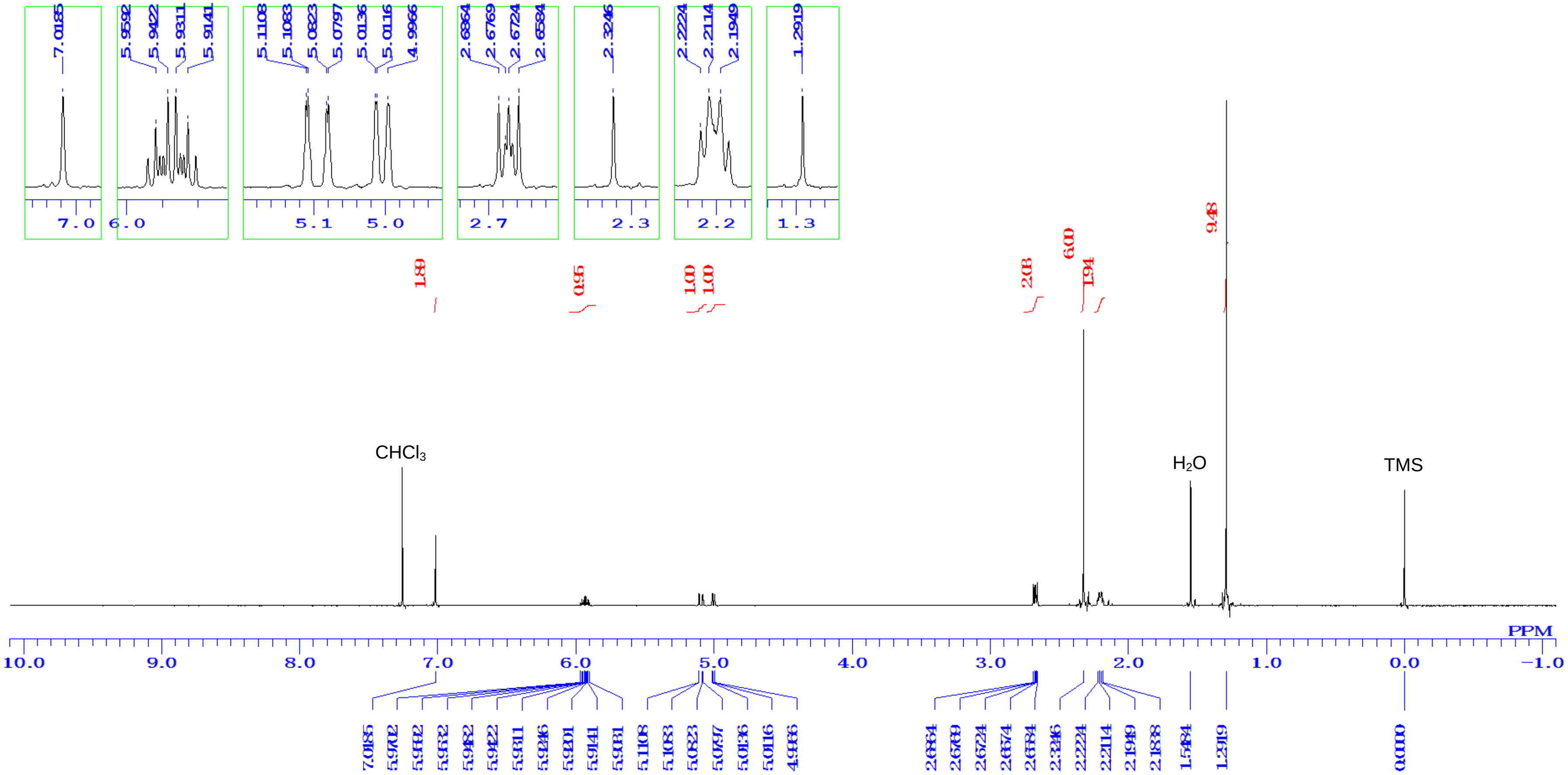

2-(But-3-en-1-yl)-5-*tert*-butyl-1,3-dimethylbenzene (39)

$^{13}C\{^{1}H\}$ NMR ($CDCl_3$, 150 MHz)

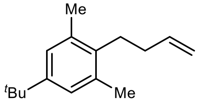

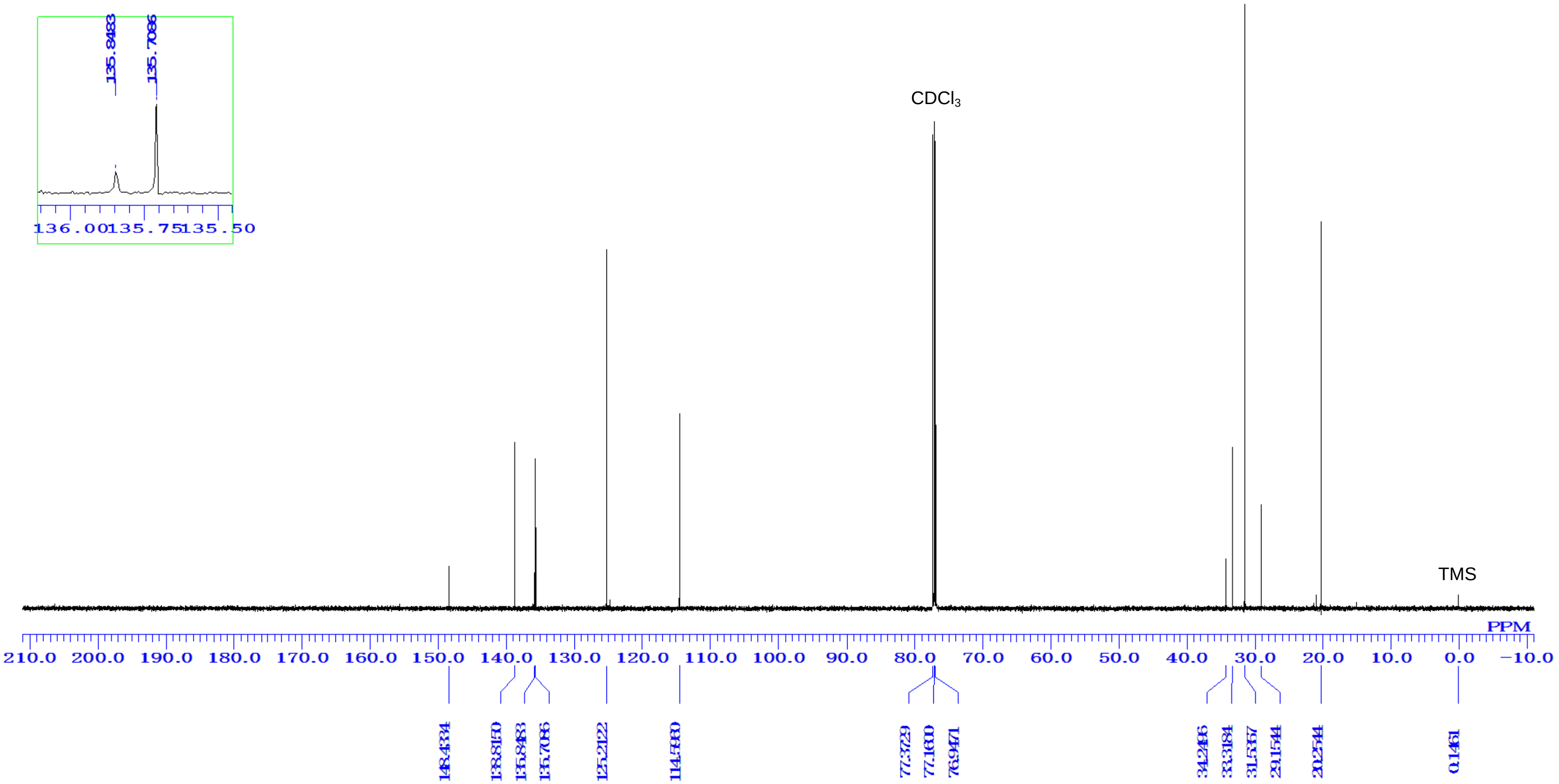

5-*tert*-Butyl-1,3-dimethyl-2-(3-methylbut-3-en-1-yl)benzene (40)

^{1}H NMR ($CDCl_3$, 600 MHz)

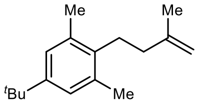

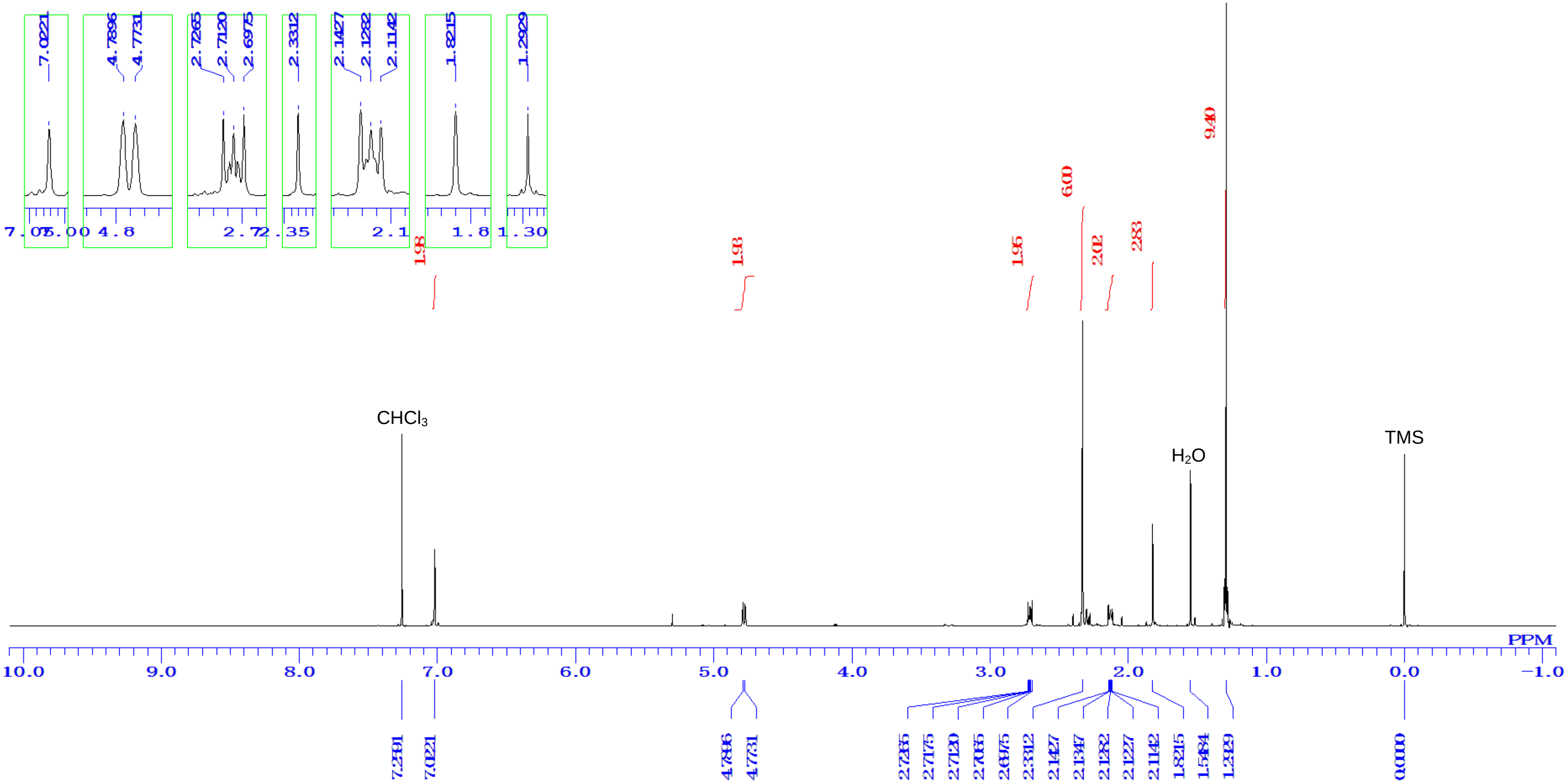

5-*tert*-Butyl-1,3-dimethyl-2-(3-methylbut-3-en-1-yl)benzene (40)

$^{13}C\{^{1}H\}$ NMR ($CDCl_3$, 150 MHz)

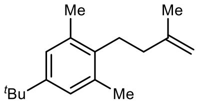

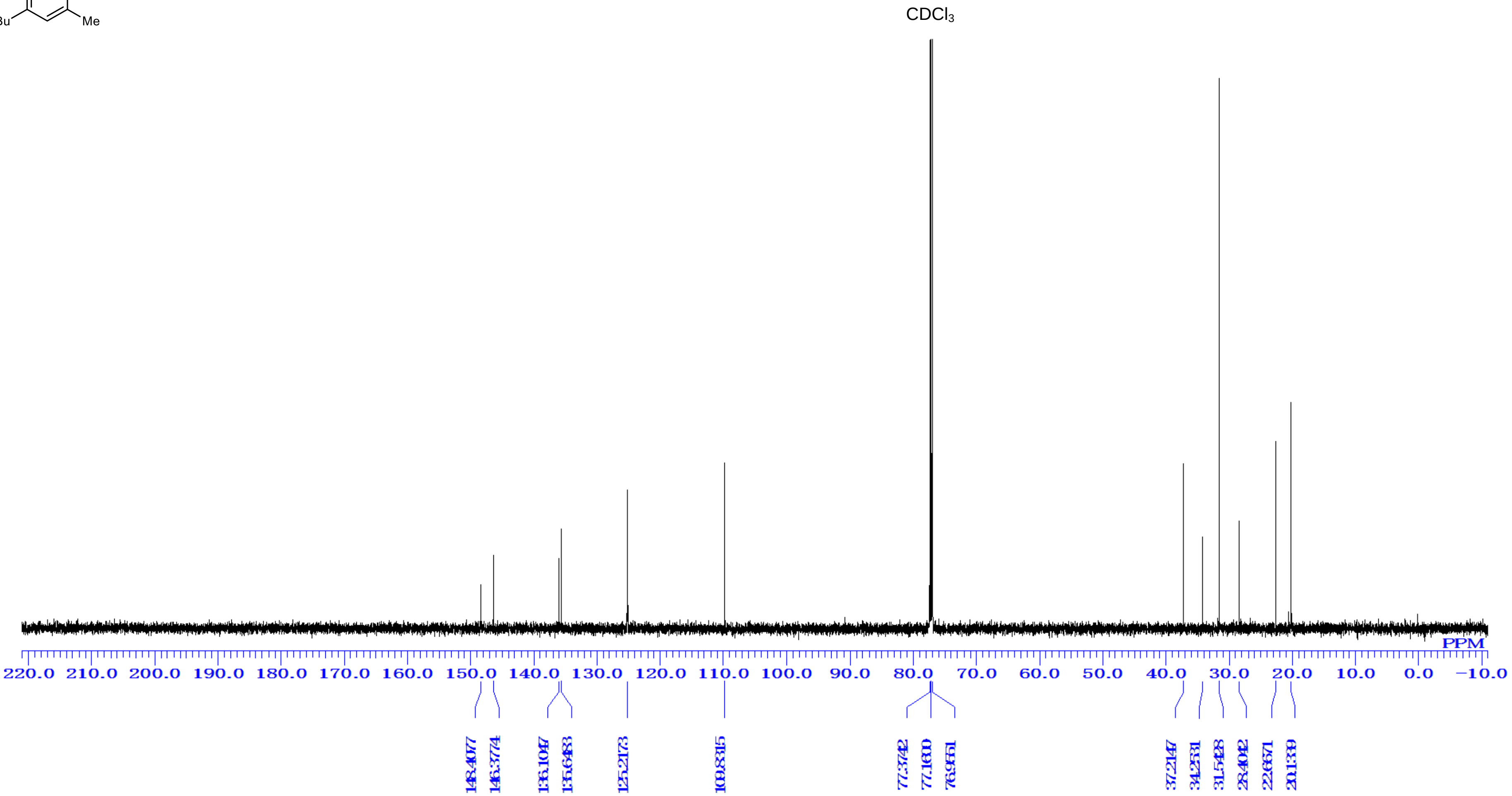

5-*tert*-Butyl-2-(2,2-dimethylbut-3-en-1-yl)-1,3-dimethylbenzene (41)

^1H NMR ($CDCl_3$, 600 MHz)

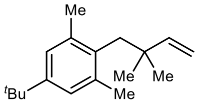

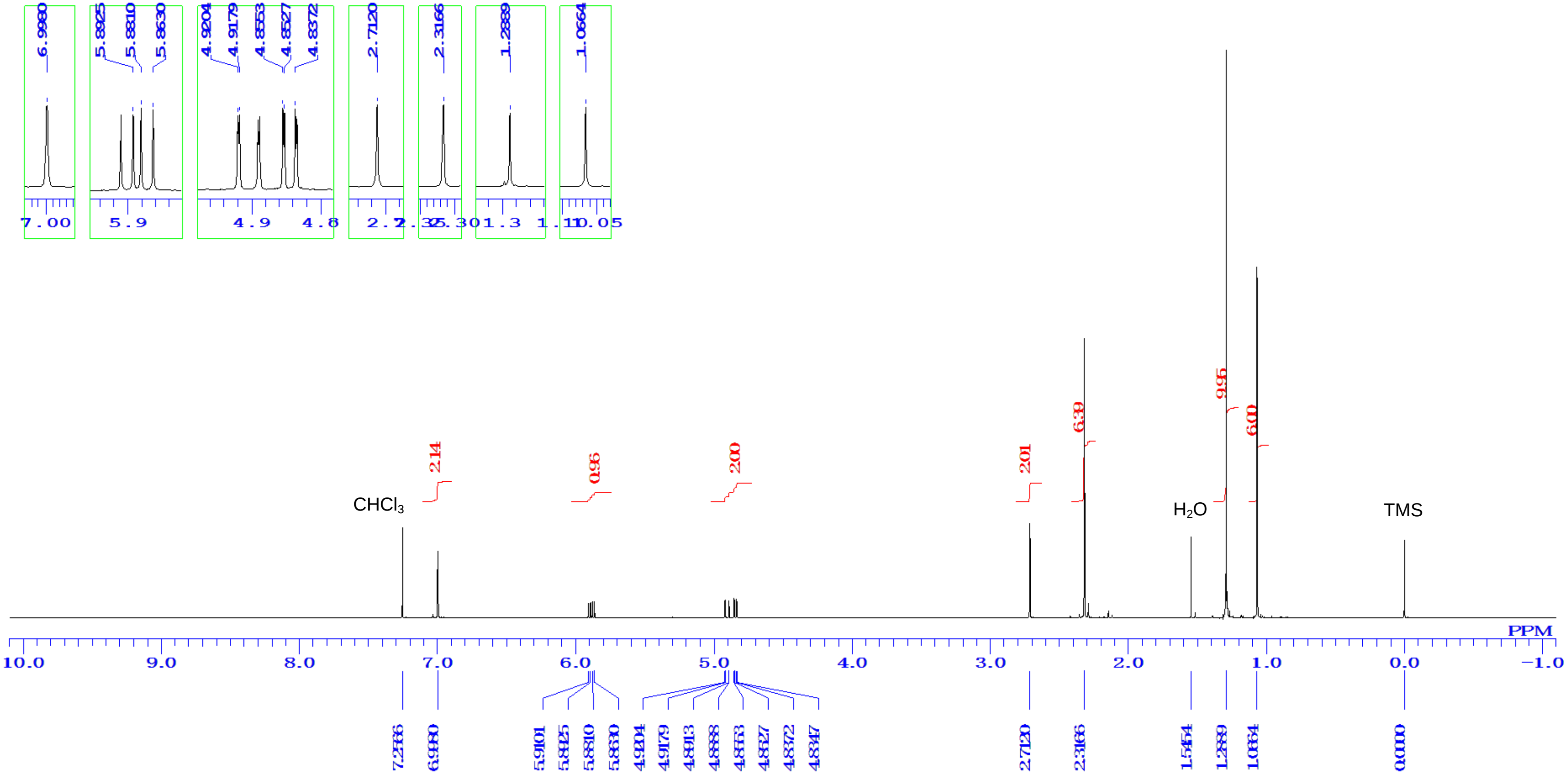

5-*tert*-Butyl-2-(2,2-dimethylbut-3-en-1-yl)-1,3-dimethylbenzene (41)

$^{13}C\{^{1}H\}$ NMR ($CDCl_3$, 150 MHz)

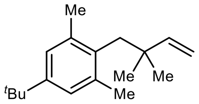

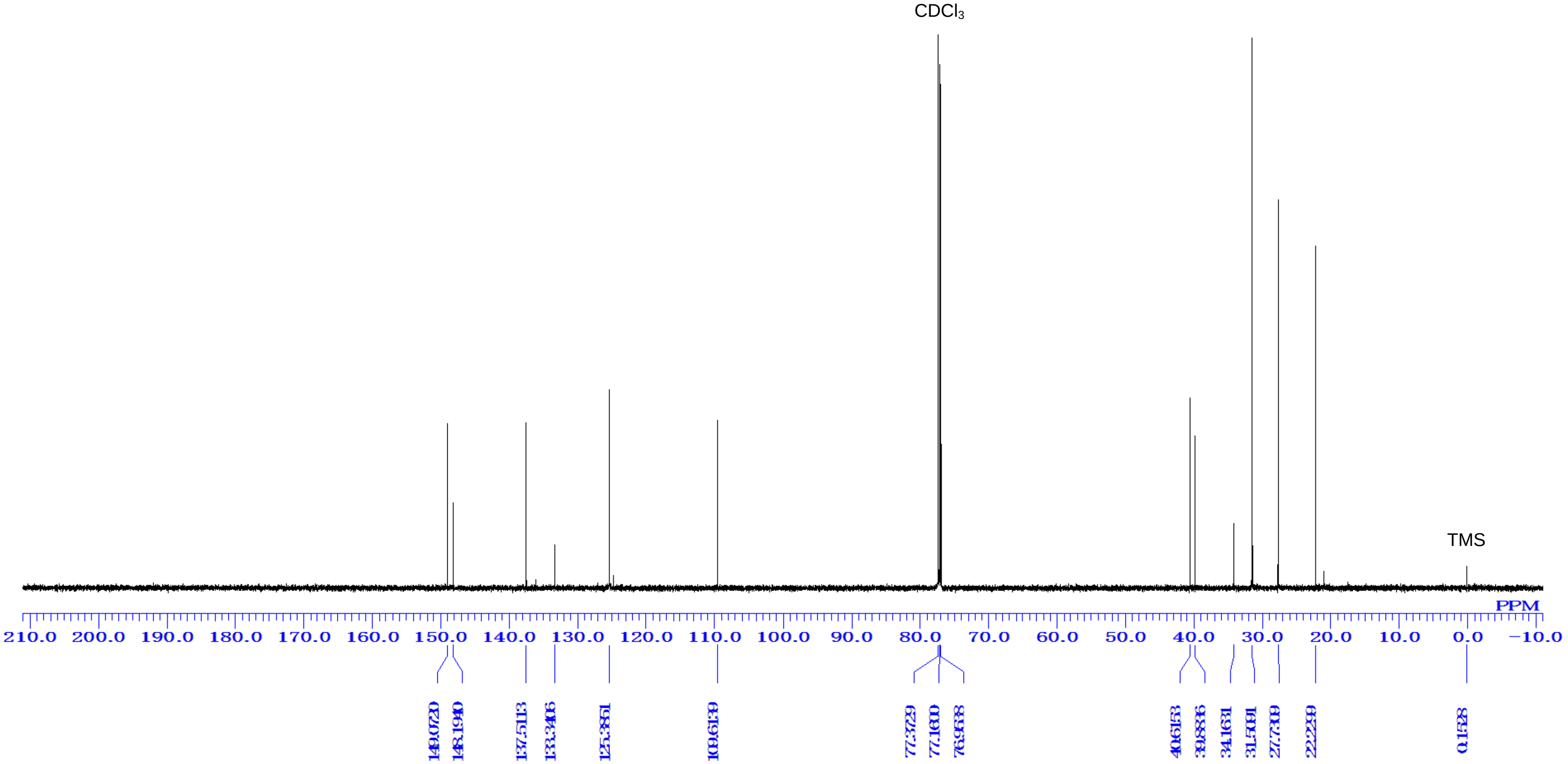

(10-Acetoxy)decyl (4,4-dimethyl-1-phenylpent-2-yn-1-yl) ether (42)

^{1}H NMR ($CDCl_3$, 600 MHz)

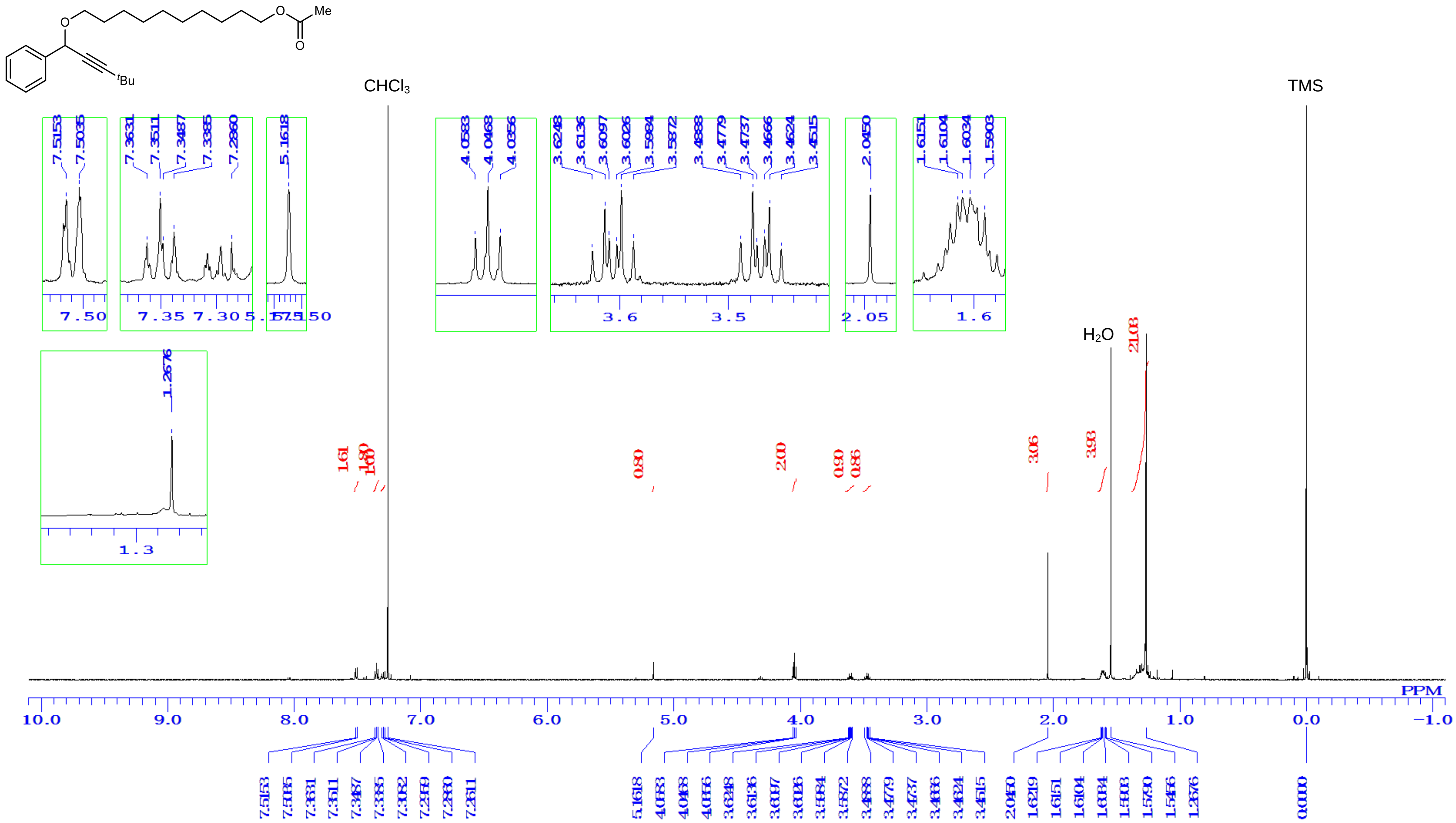

(10-Acetoxy)decyl (4,4-dimethyl-1-phenylpent-2-yn-1-yl) ether (42)

$^{13}C\{^{1}H\}$ NMR ($CDCl_3$, 100 MHz)

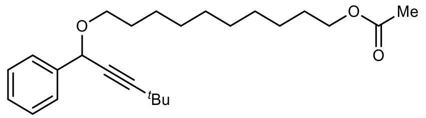

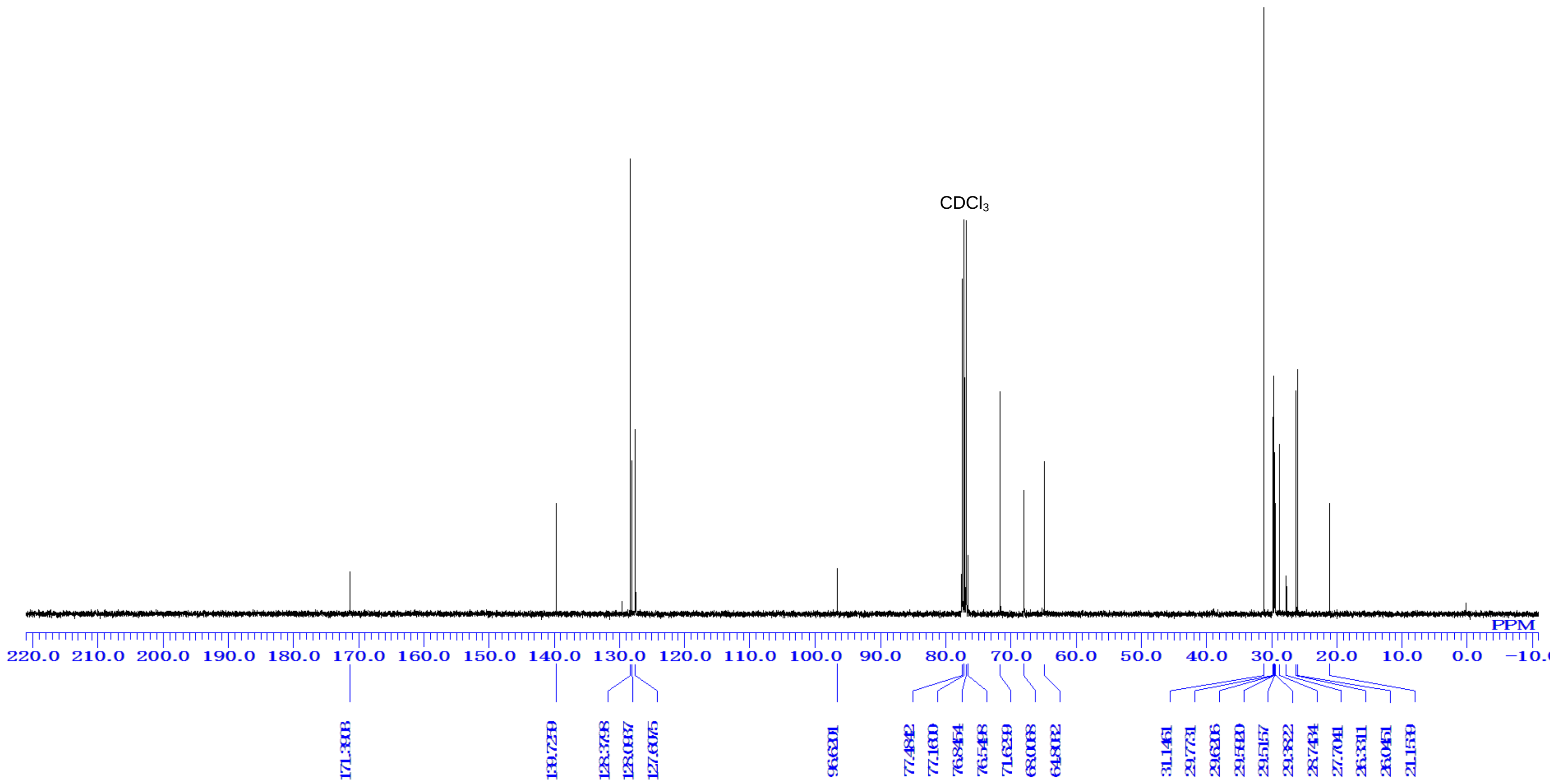

Bis(3,5-di-*tert*-butylbenzyl) ether (8d)
^{1}H NMR ($CDCl_3$, 600 MHz)

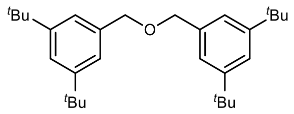

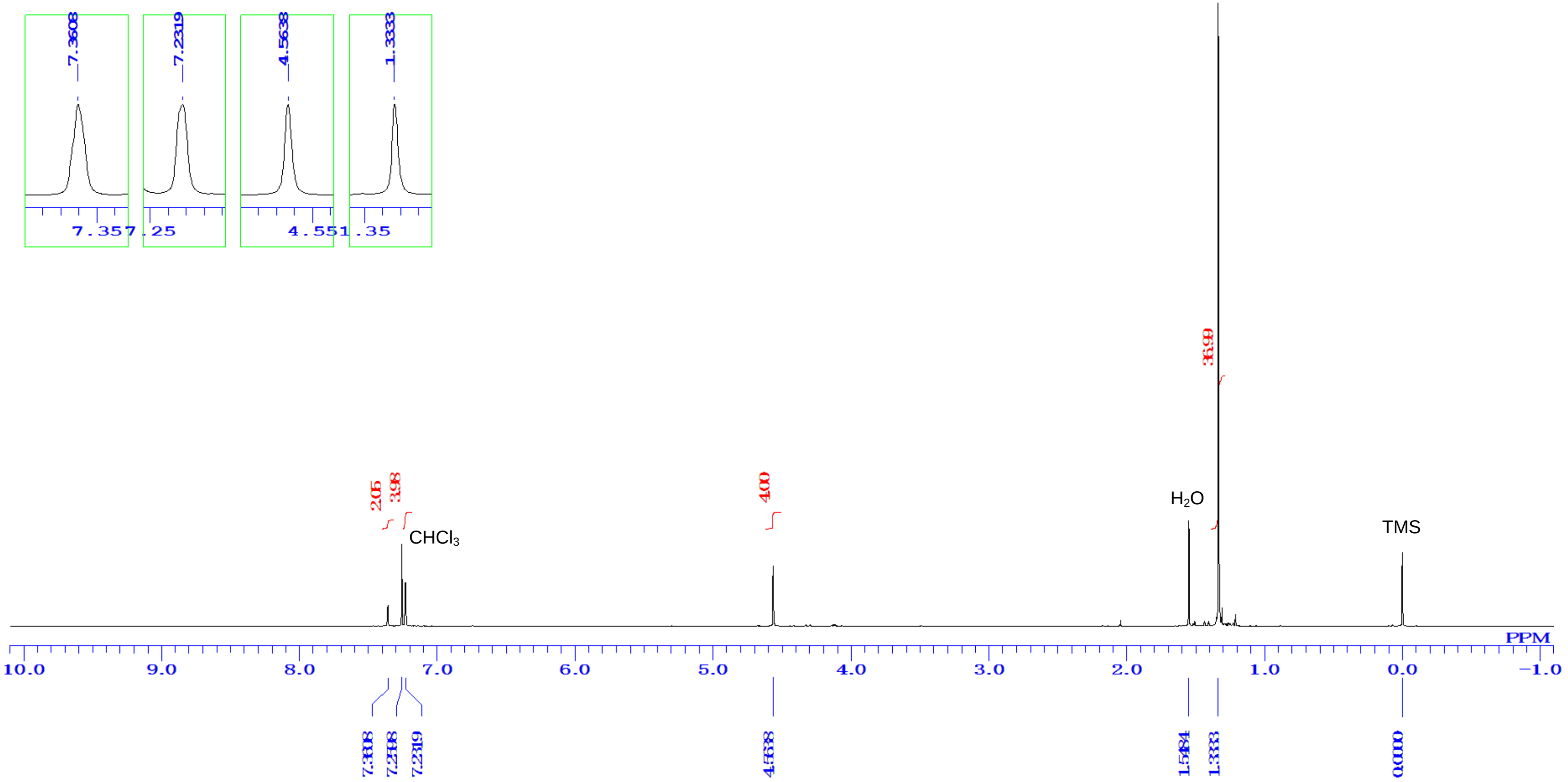

Bis(3,5-di-*tert*-butylbenzyl) ether (8d)

$^{13}C\{^{1}H\}$ NMR ($CDCl_3$, 150 MHz)

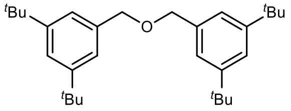

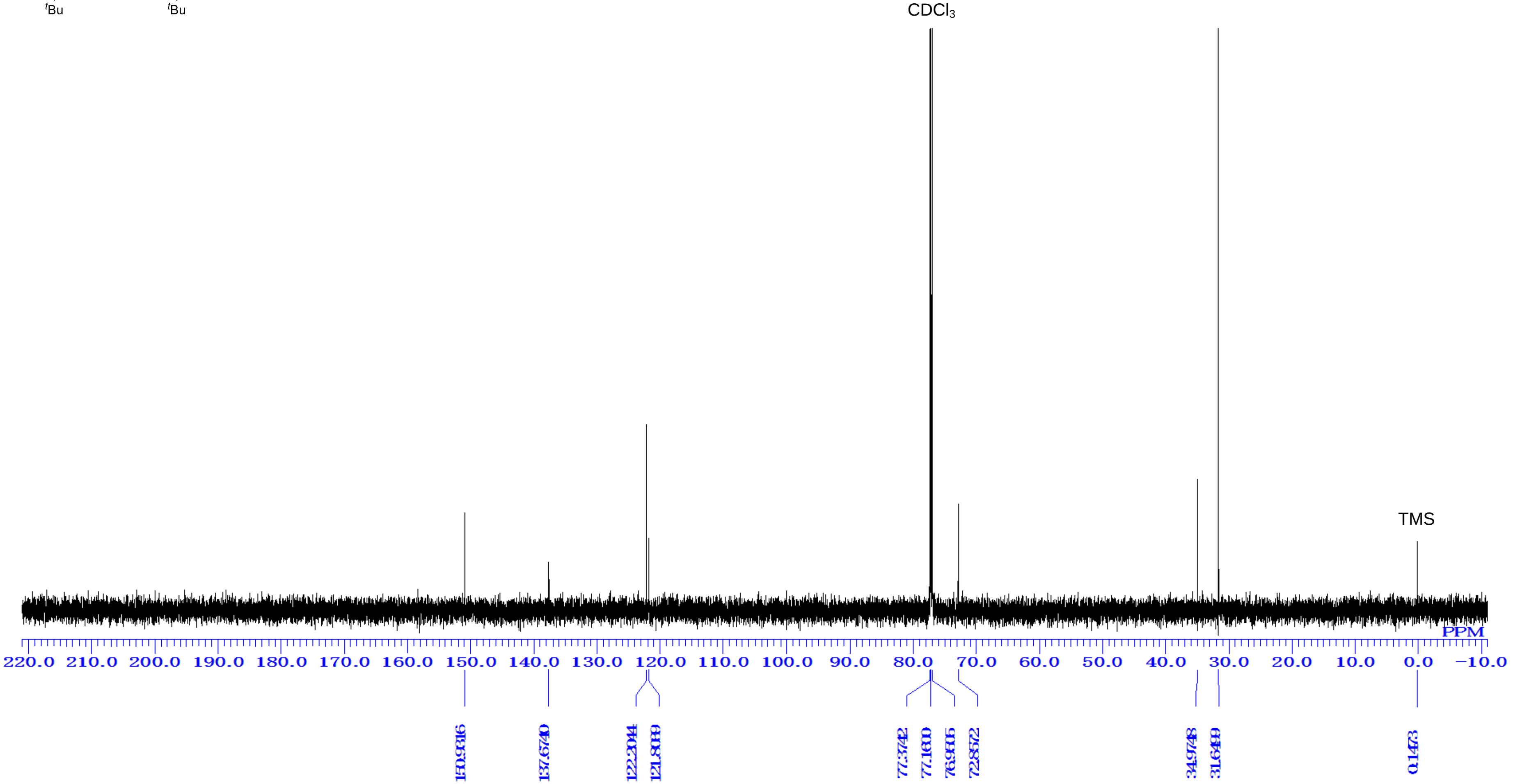

4-*tert*-Butylbenzyl 3,5-di-*tert*-butylbenzyl ether (8ad)

^{1}H NMR ($CDCl_3$, 600 MHz)

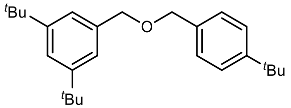

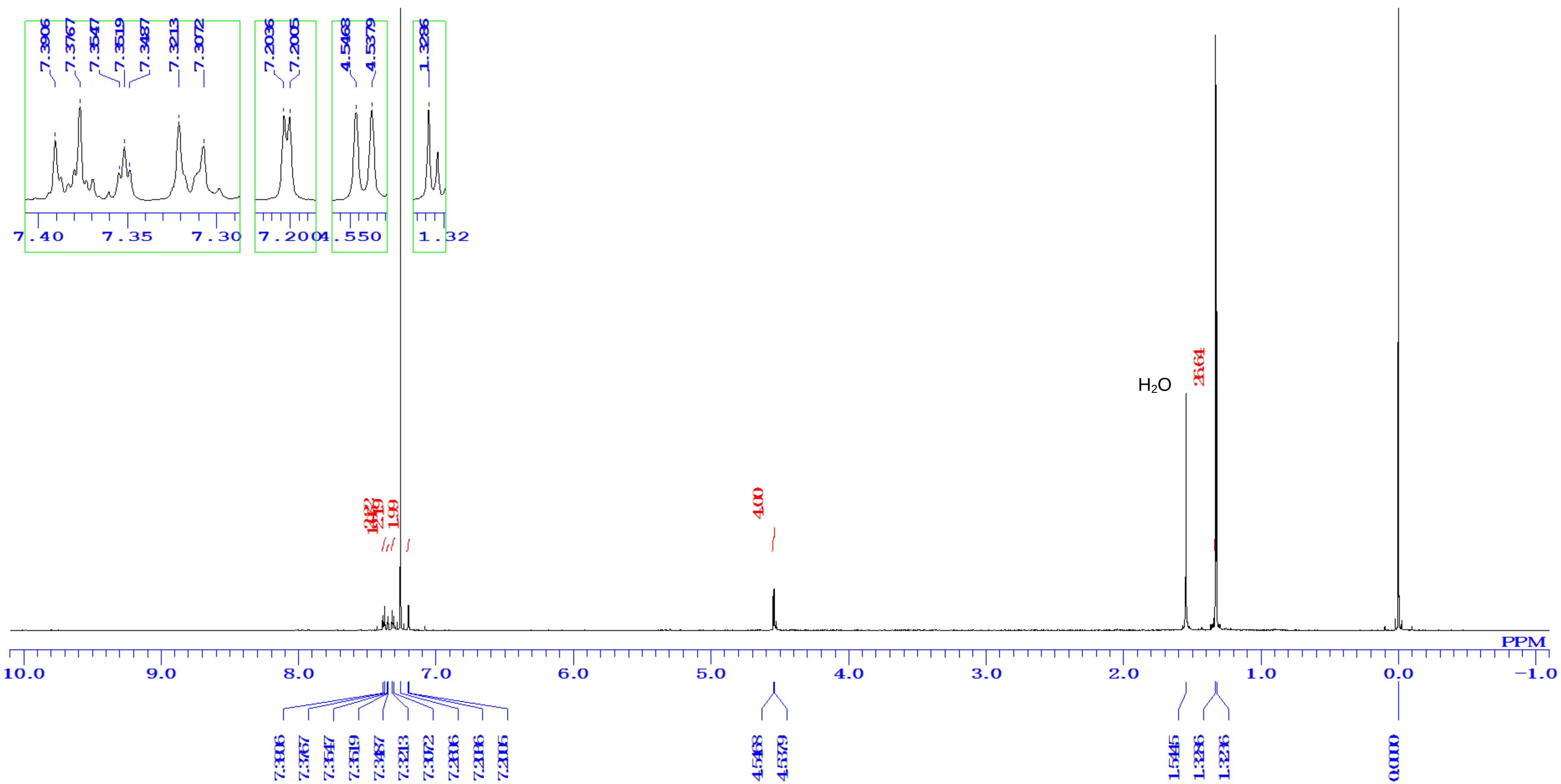

4-*tert*-Butylbenzyl 3,5-di-*tert*-butylbenzyl ether (8ad)

$^{13}C\{^{1}H\}$ NMR ($CDCl_3$, 150 MHz)

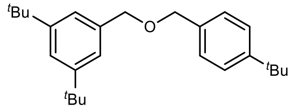

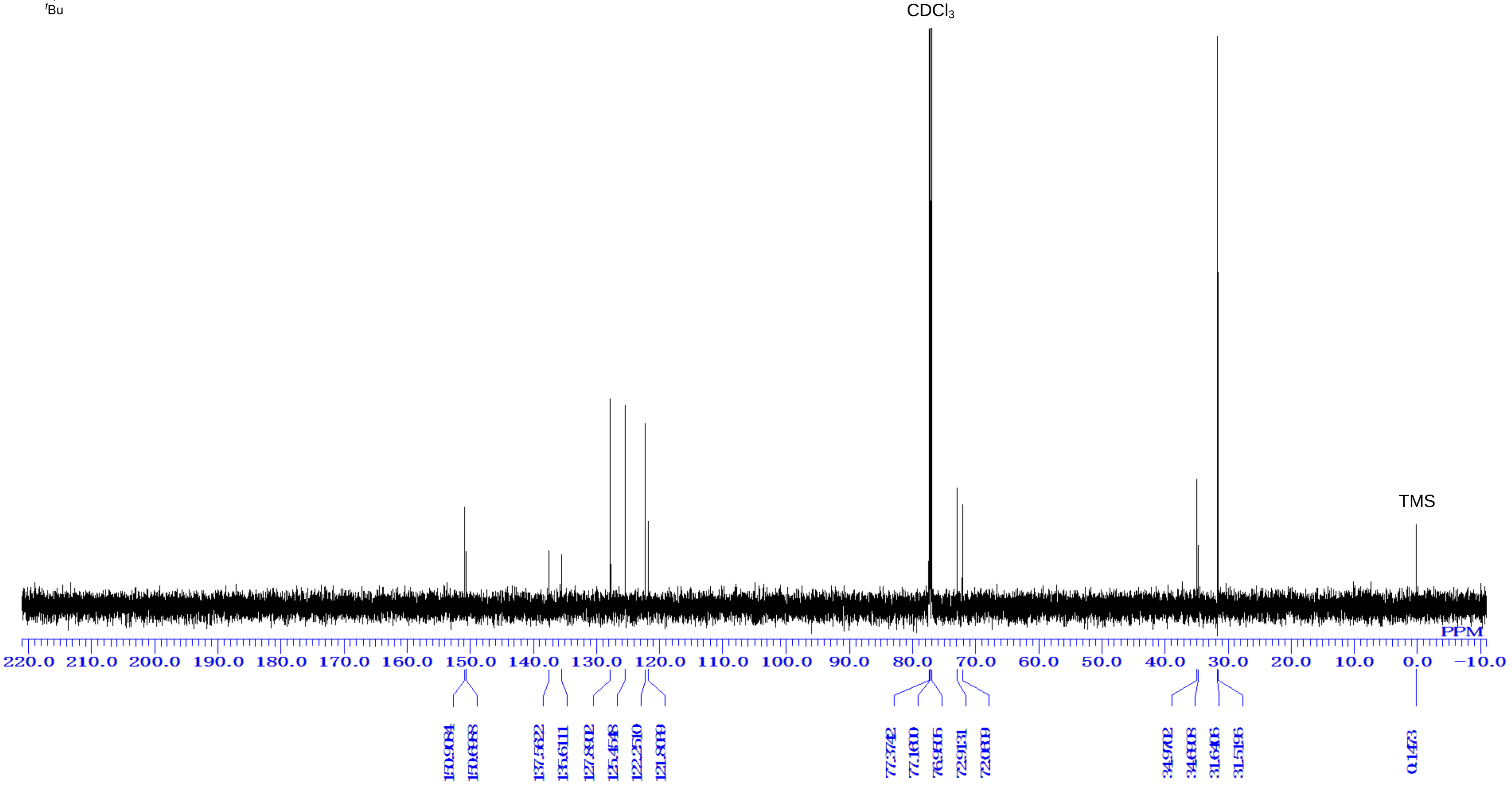

1-(4-*tert*-Butyl-2,6-dimethyl)benzyl-2,2,6,6-tetramethylpiperidine (45)

^{1}H NMR ($CDCl_3$, 600 MHz)

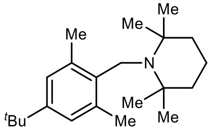

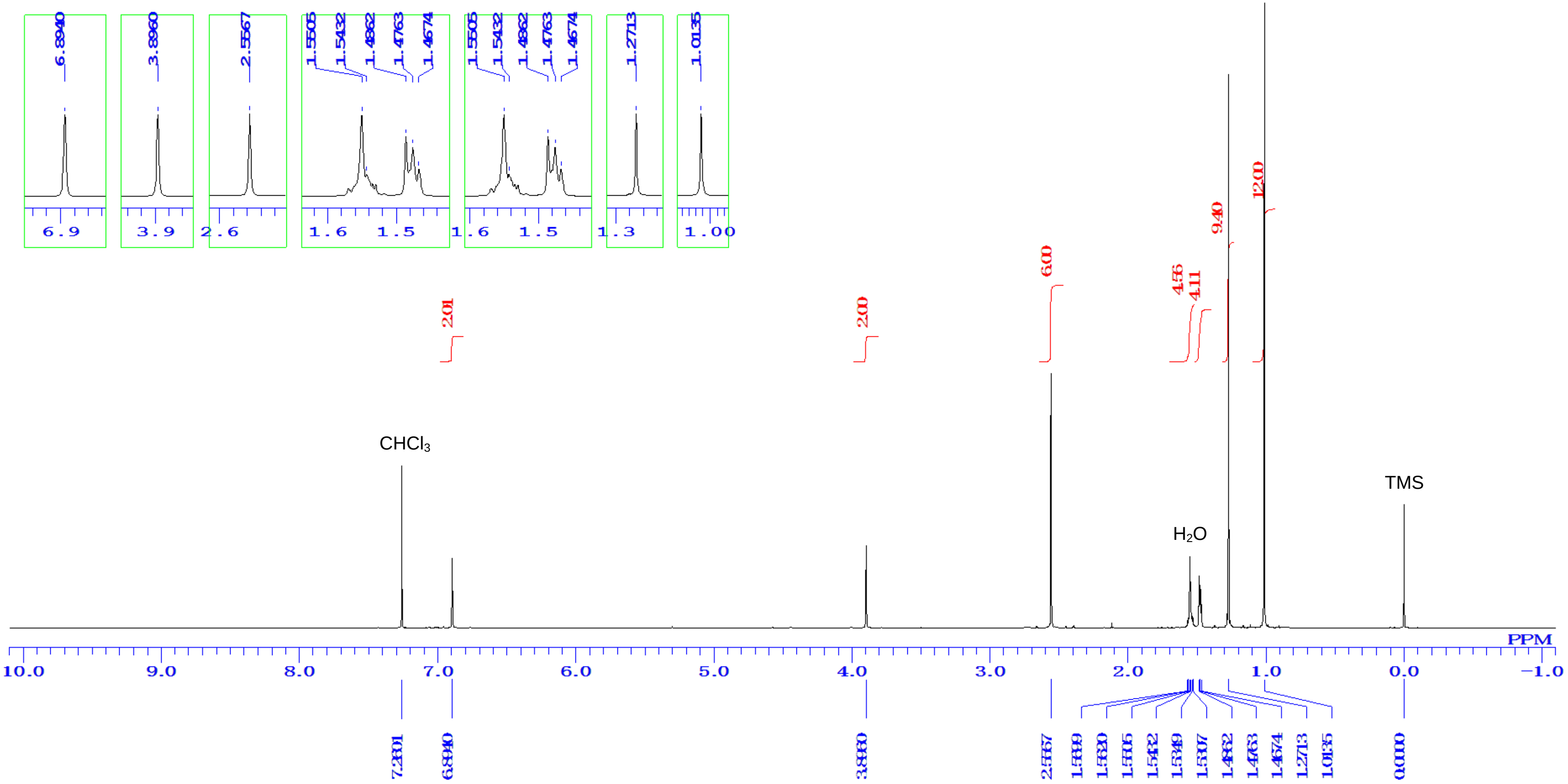

1-(4-*tert*-Butyl-2,6-dimethyl)benzyl-2,2,6,6-tetramethylpiperidine (45)

$^{13}C\{^{1}H\}$ NMR ($CDCl_3$, 150 MHz)

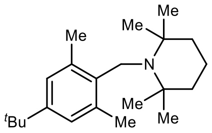

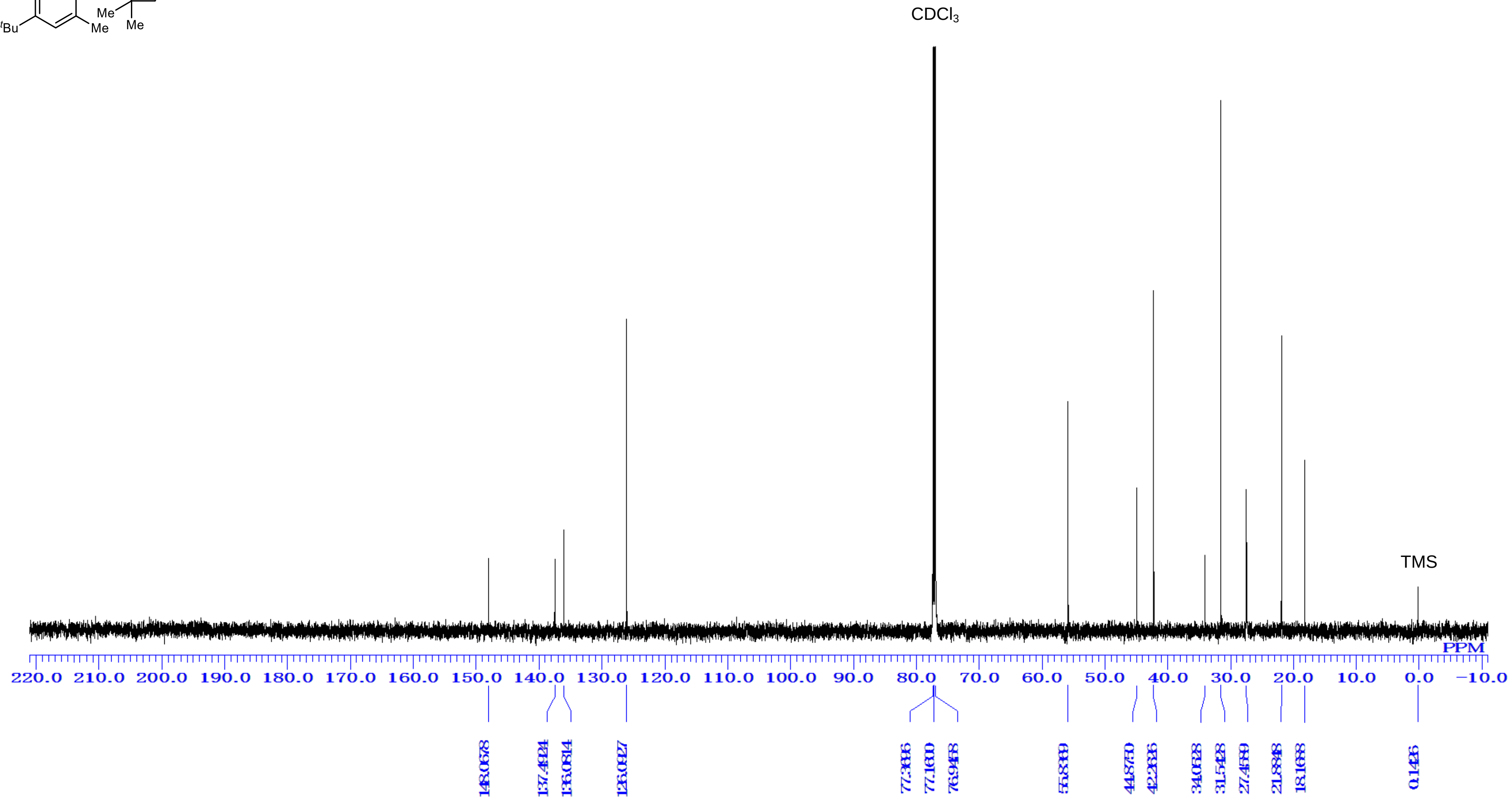

(4-*tert*-Butyl-2,6-dimethyl)benzyl 2,2,2-trichloroacetimidate (46)

^{1}H NMR ($CDCl_3$, 600 MHz)

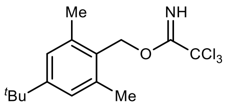

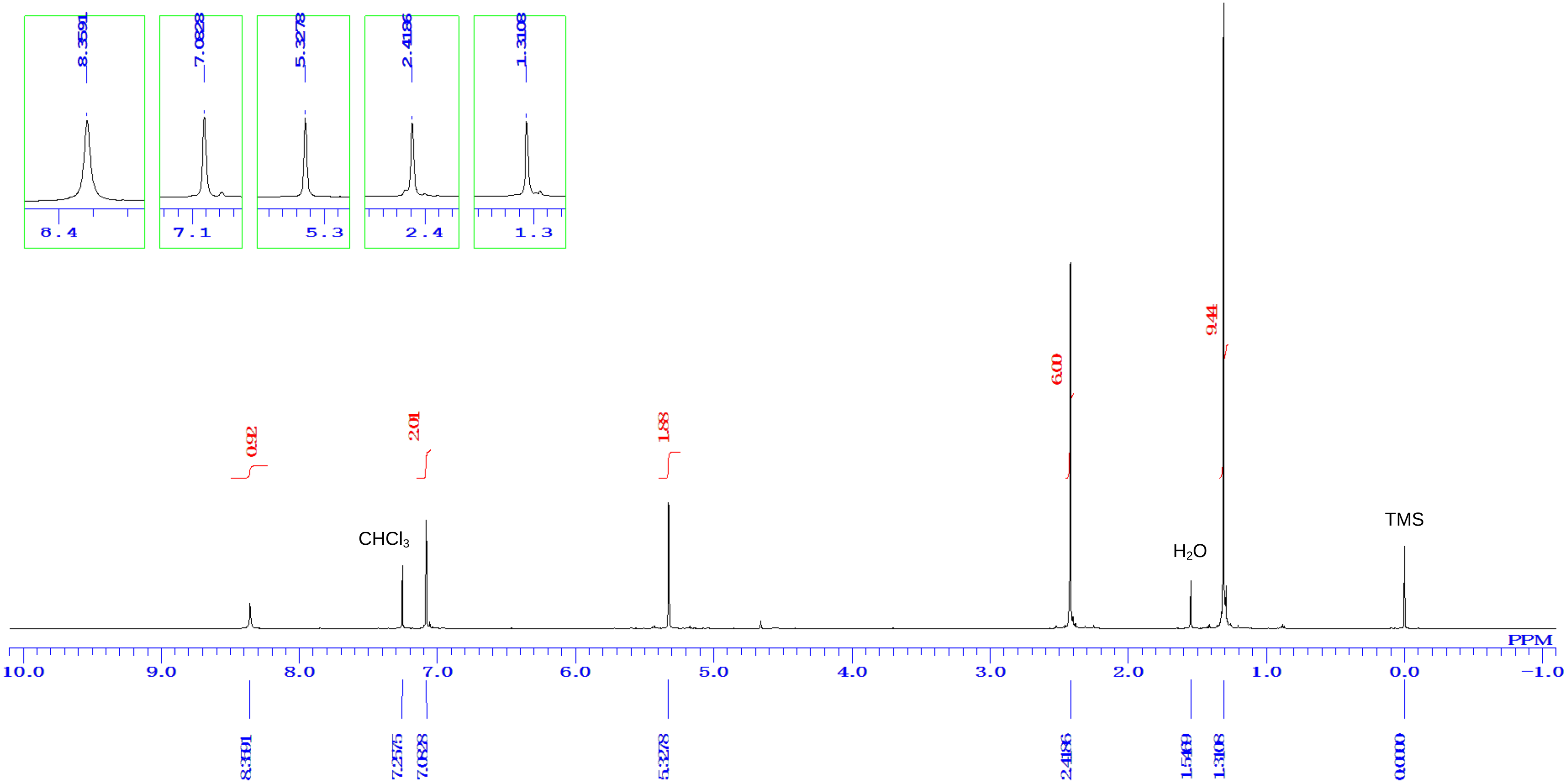

(4-*tert*-Butyl-2,6-dimethyl)benzyl 2,2,2-trichloroacetimidate (46)

$^{13}C\{^{1}H\}$ NMR ($CDCl_3$, 150 MHz)

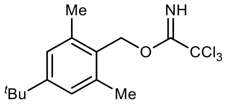

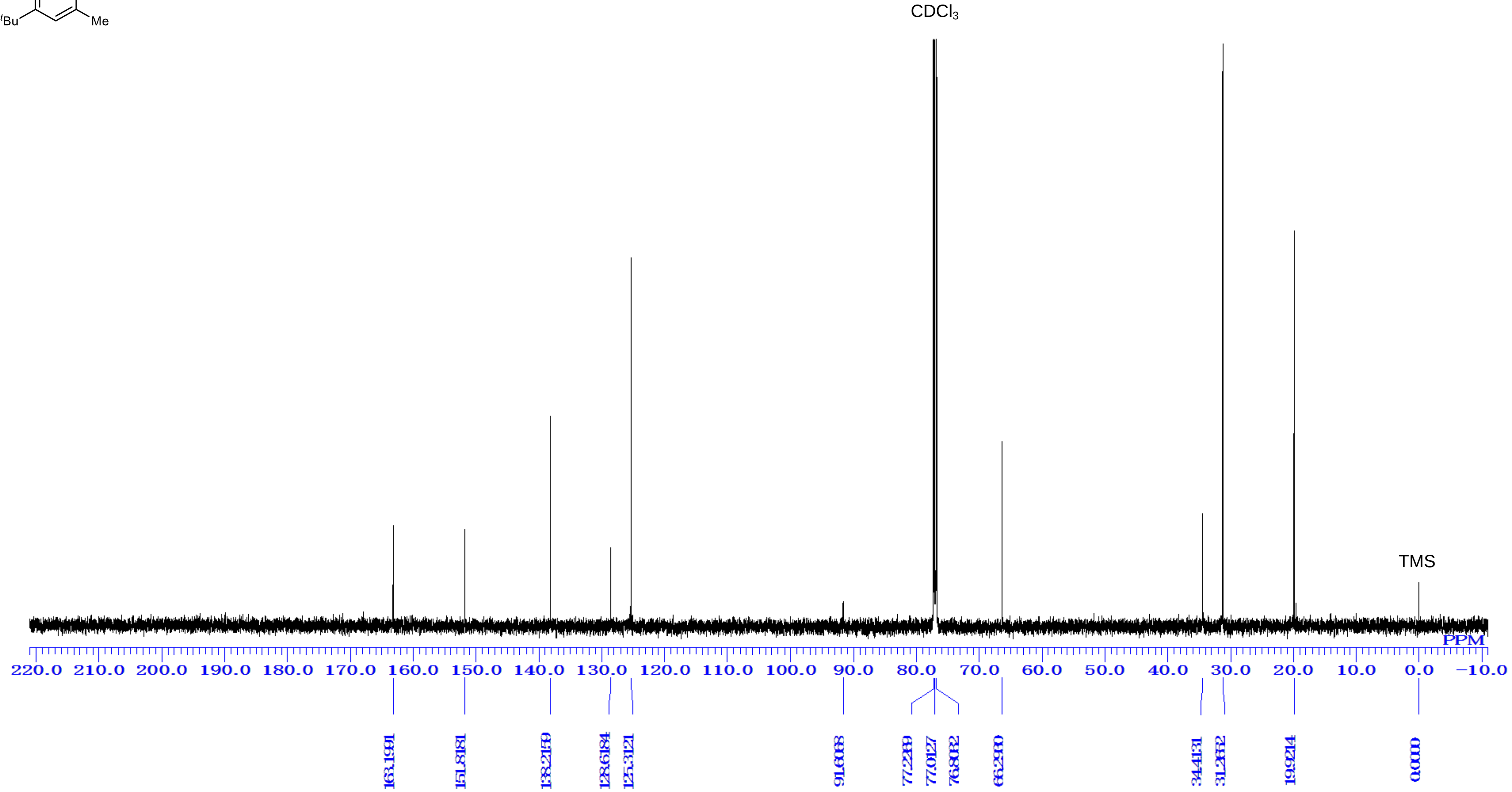

HMQC and HMBC spectra

HMQC spectrum of a $CDCl_3$ solution containing carbocationoid **3a**

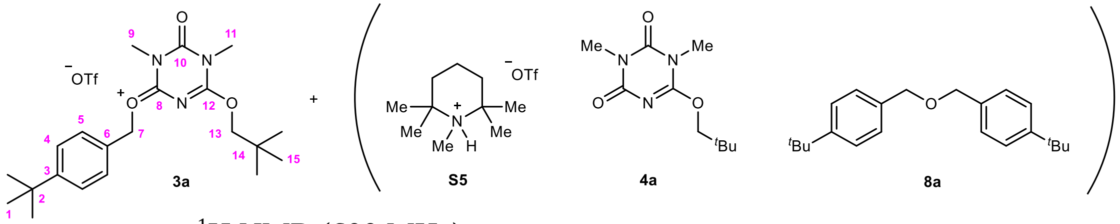

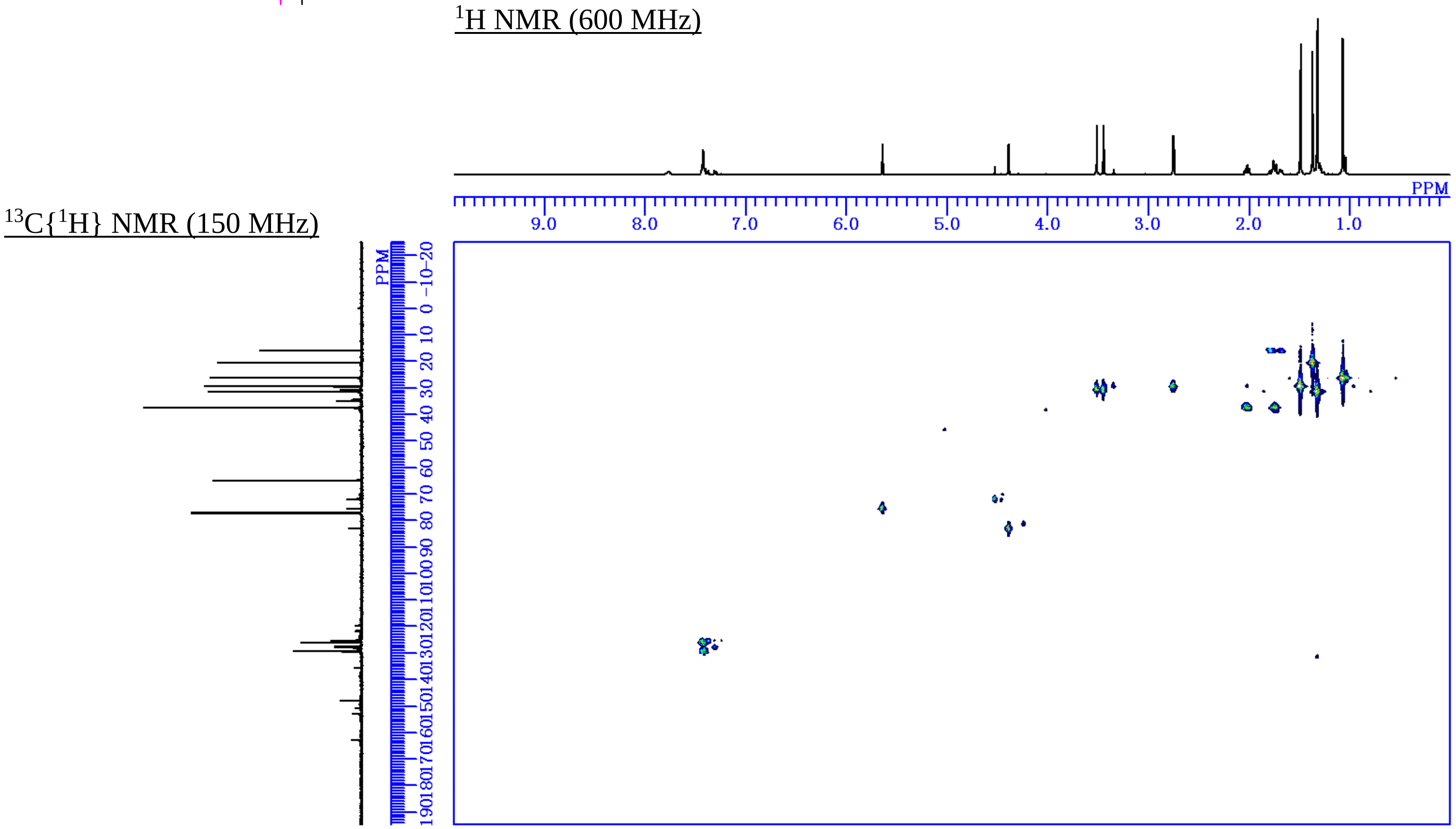

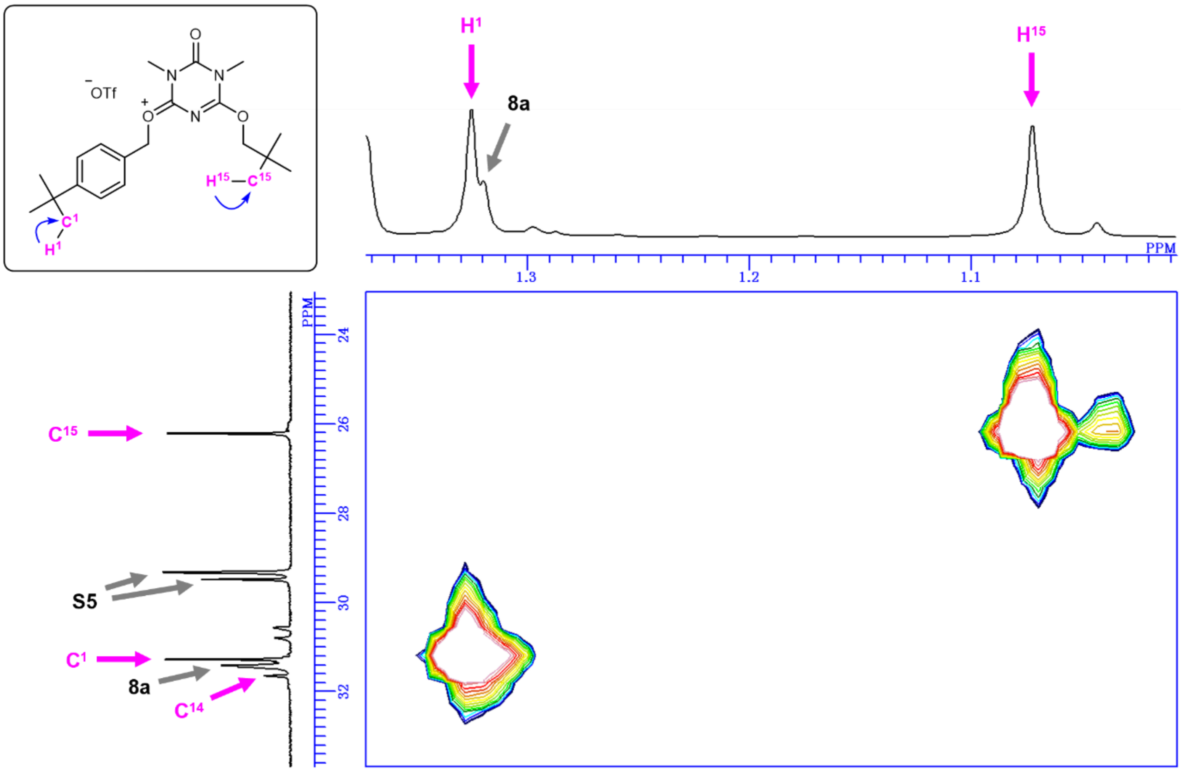

-OTf
H1
H15
8a
S5
C15
C1
C14
PPM

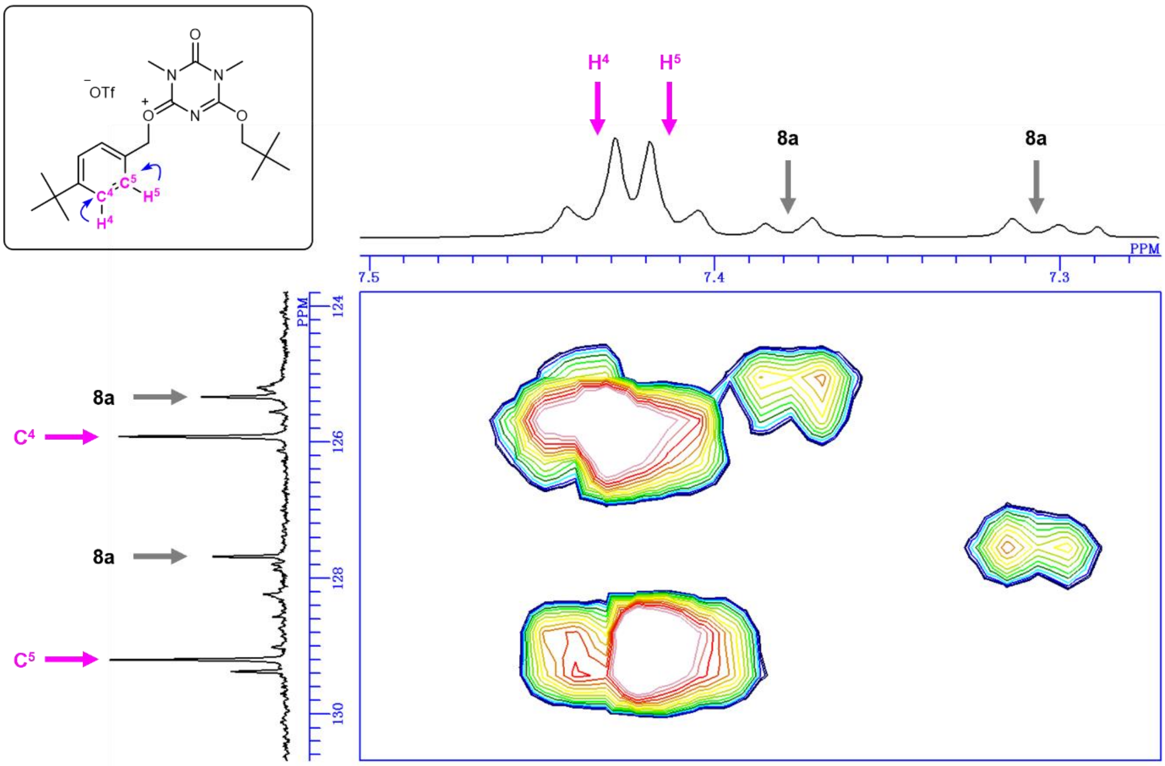

-OTf
H4
H5
8a
C4
C5
PPM

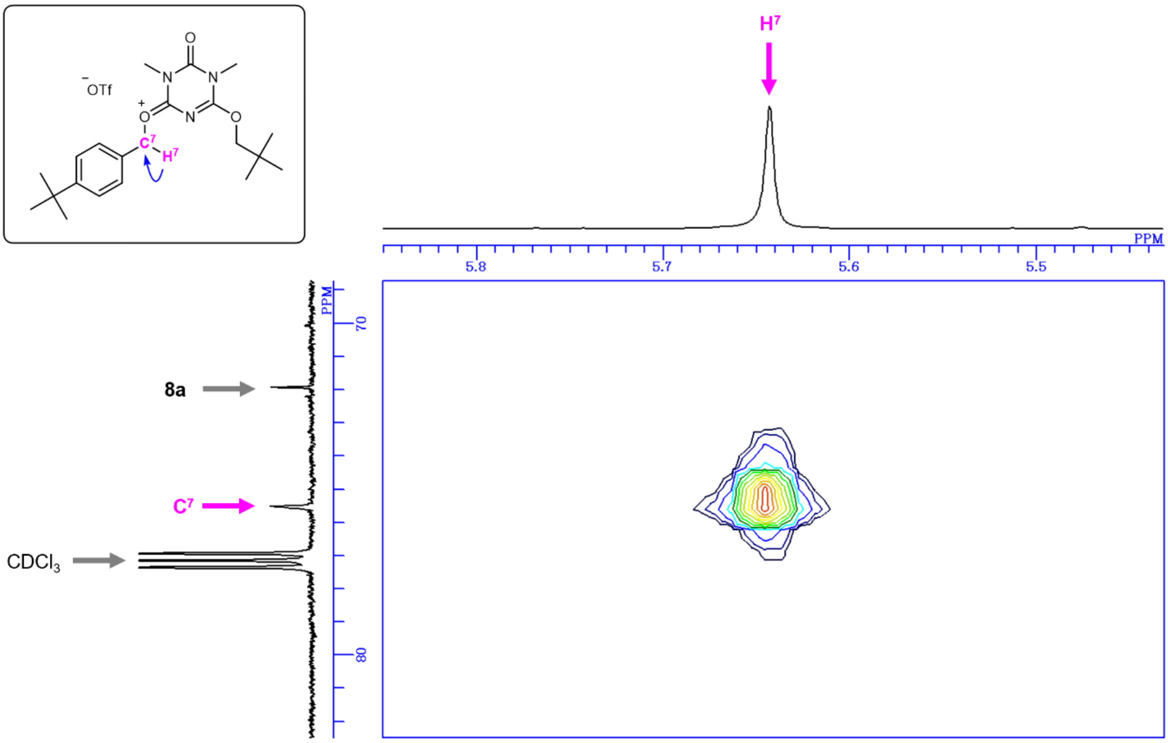

OTf
H7
C7
PPM
5.8
5.7
5.6
5.5
70
80
8a
CDCl3

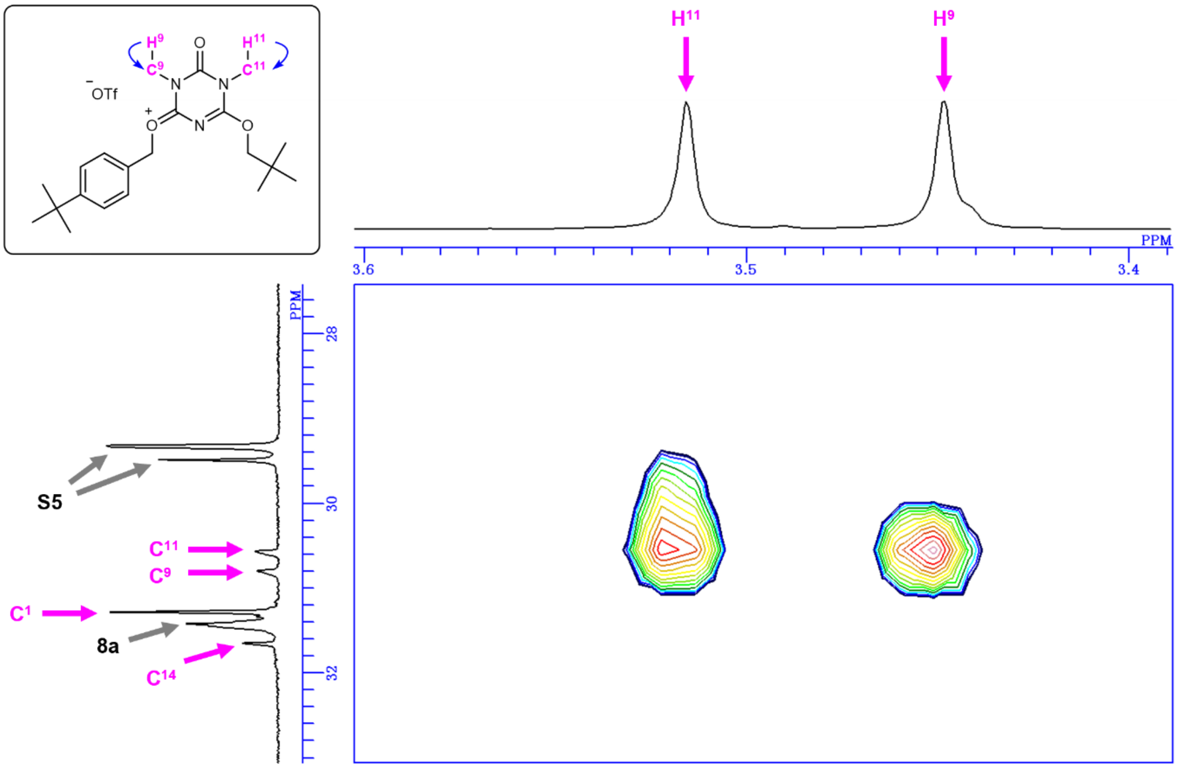

OTf
H9
C9
H11
C11
PPM
3.6
3.5
3.4
28
30
32
S5
C11
C9
C1
8a
C14

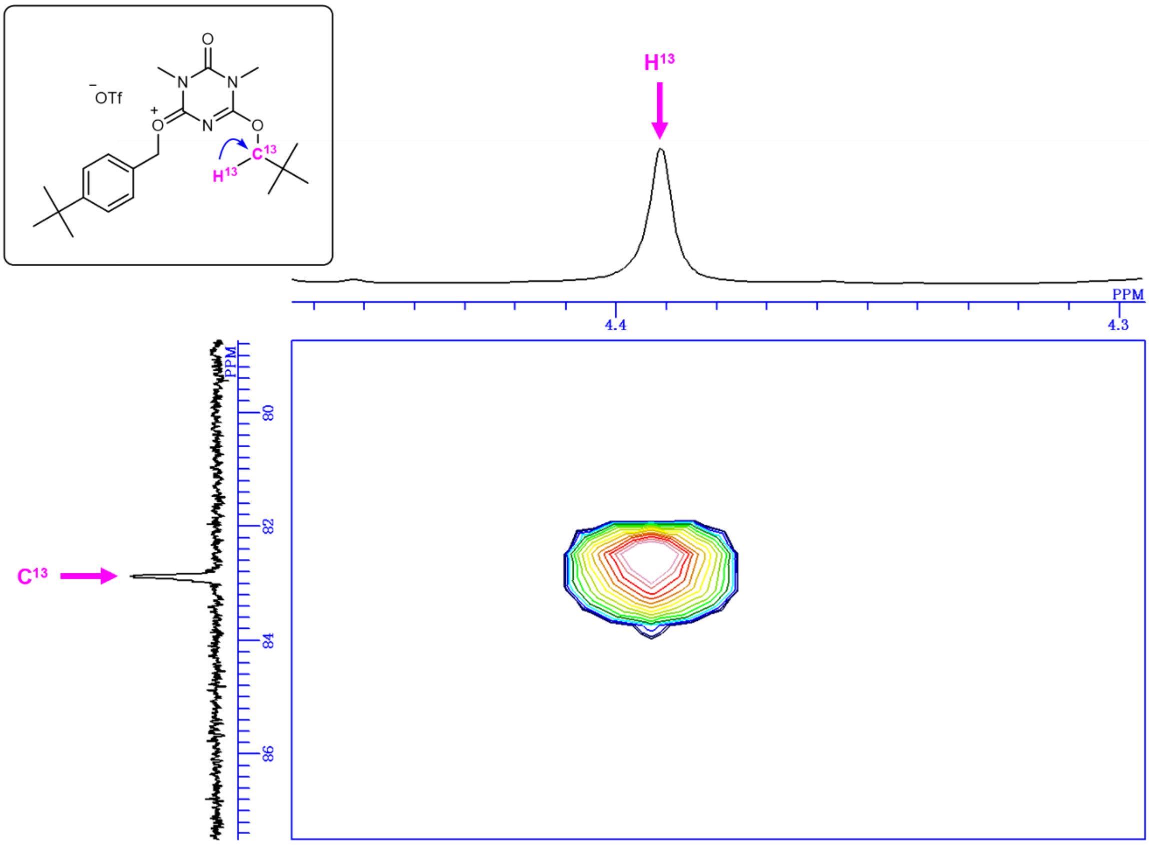

OTf
O
N
H13
C13
PPM
4.4
4.3
80
82
84
86

HMBC spectrum of a CDCl3 solution containing carbocationoid **3a**

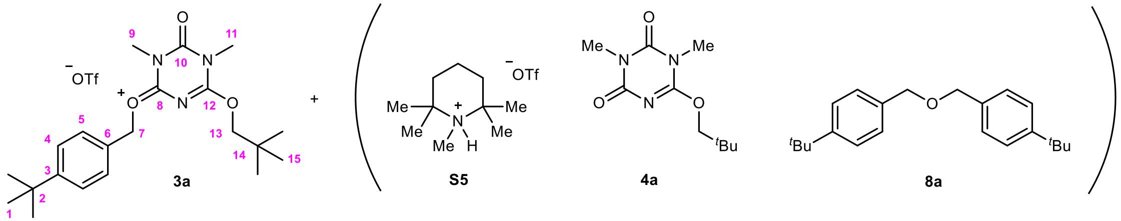

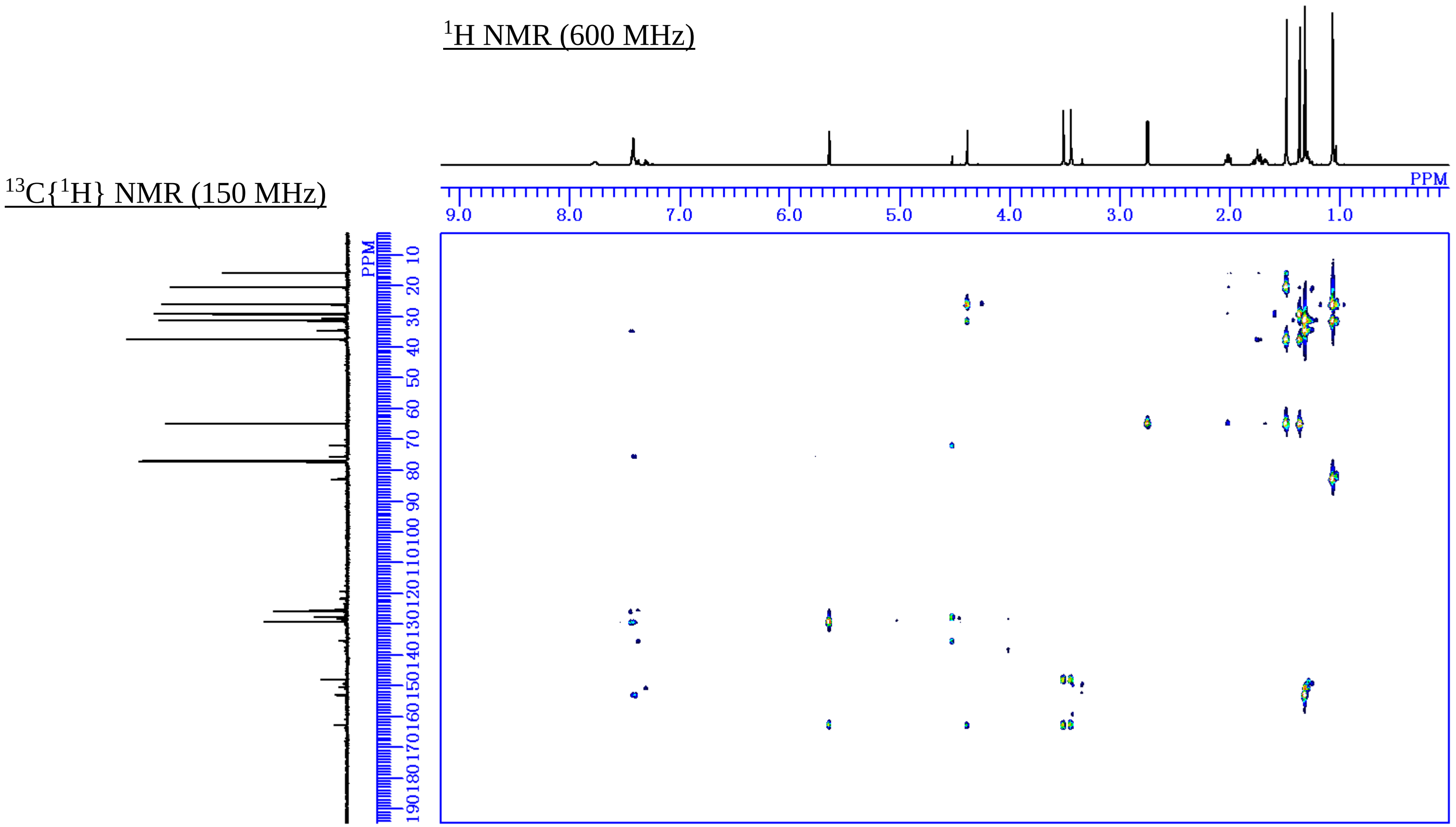

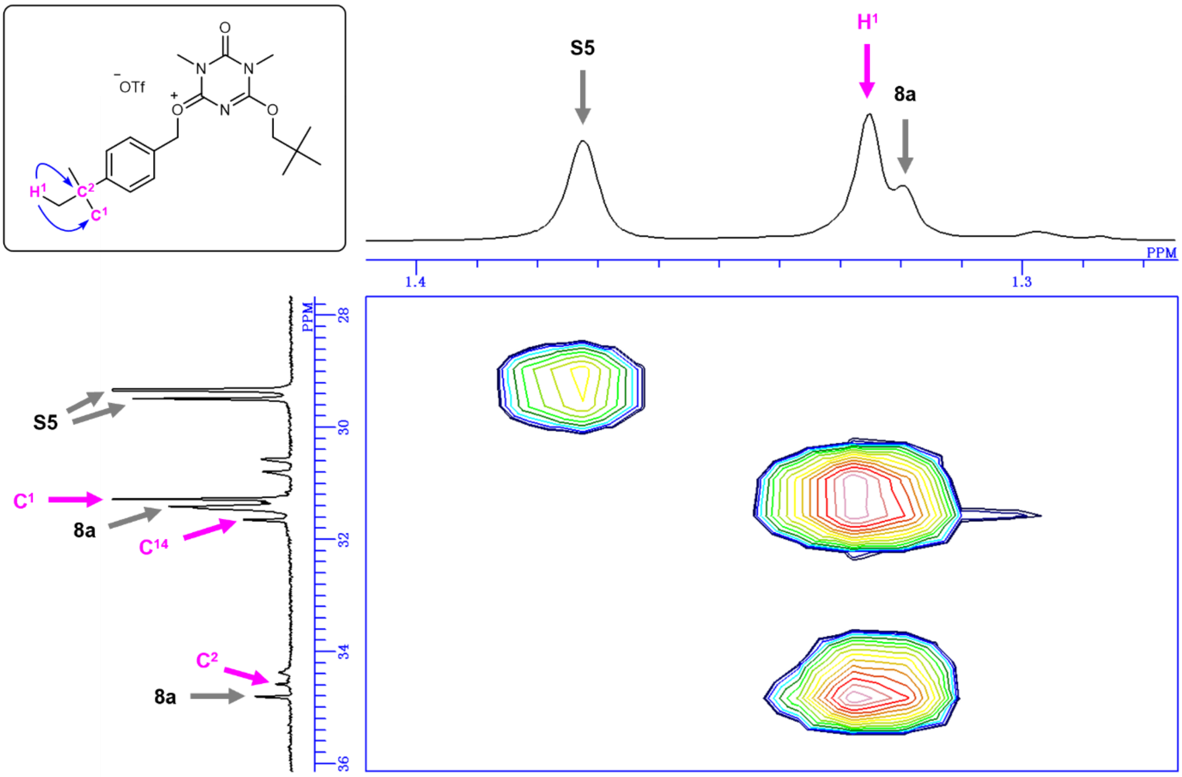

OTf
O
N
N
N
O
O
H1
C2
C1
S5
H1
8a
PPM
1.4
1.3
28
30
32
34
36
S5
C1
8a
C14
C2
8a

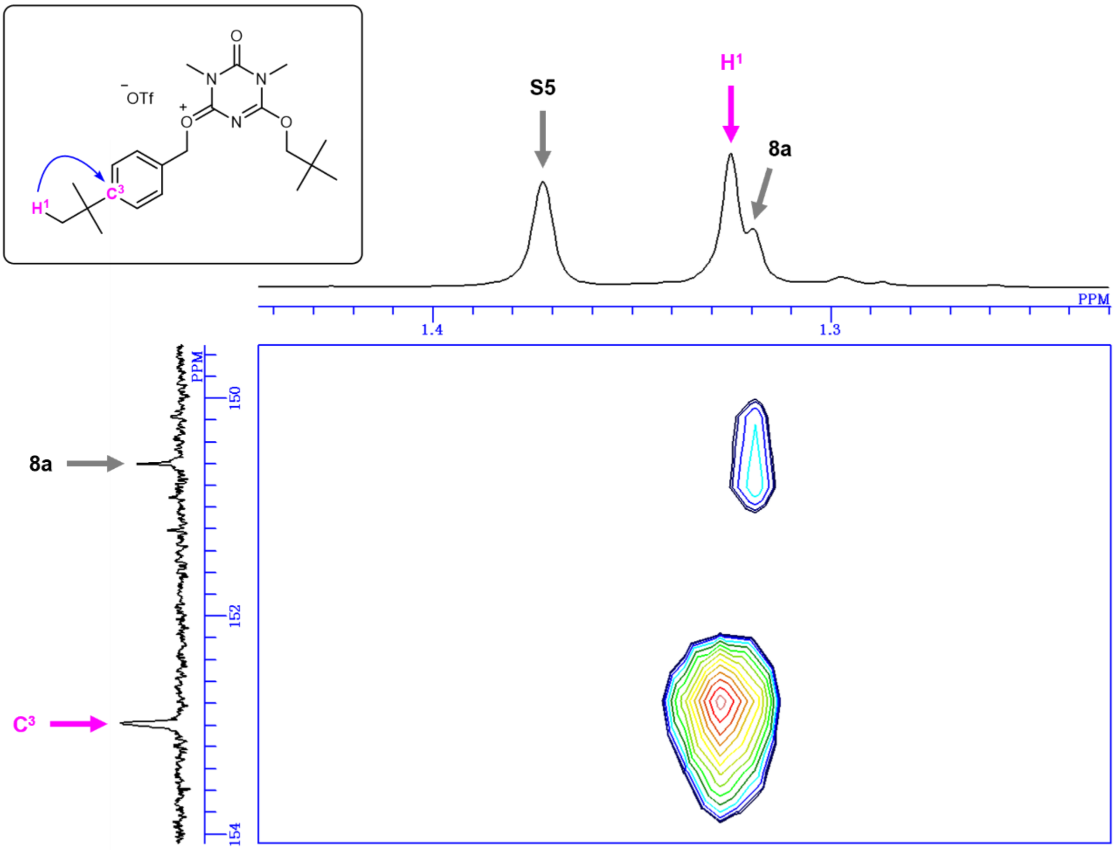

OTf
O
N
N
N
O
O
H1
C3
S5
H1
8a
PPM
1.4
1.3
150
152
154
8a
C3

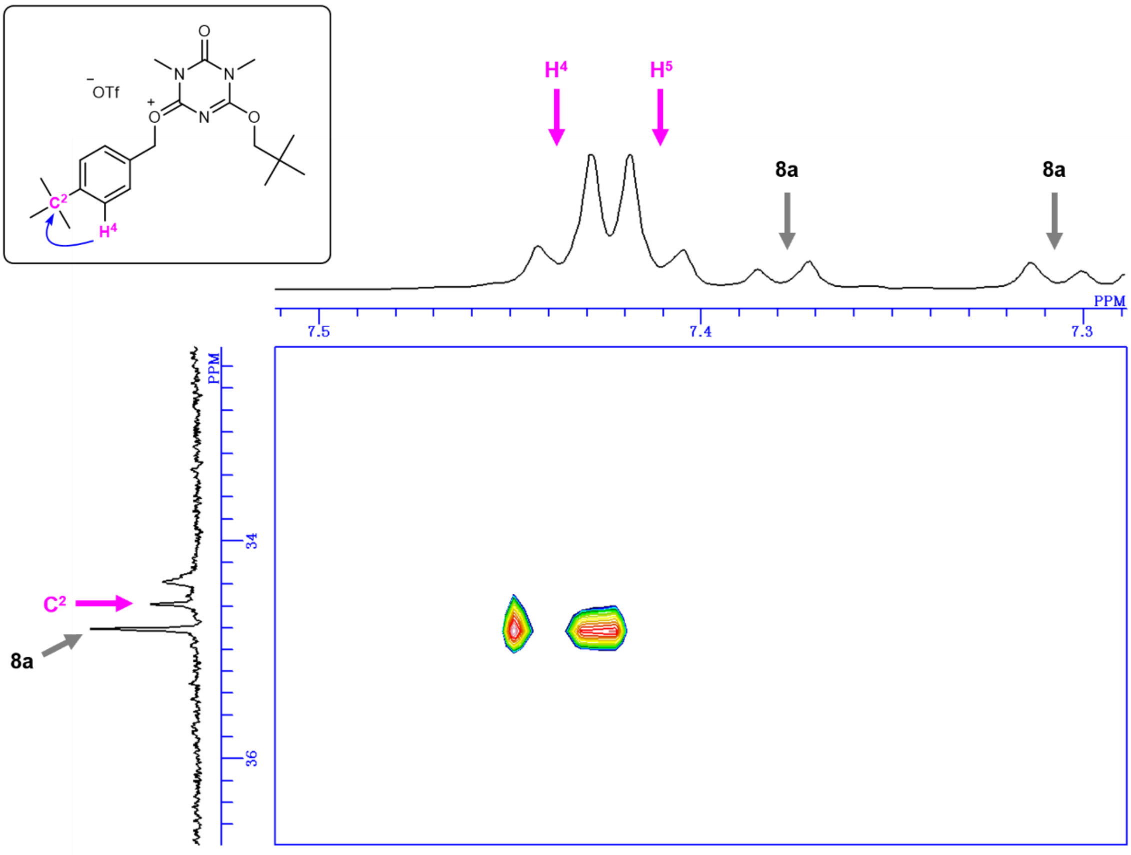

OTf
H4
H5
8a
8a
PPM
7.5
7.4
7.3
34
36
C2
8a

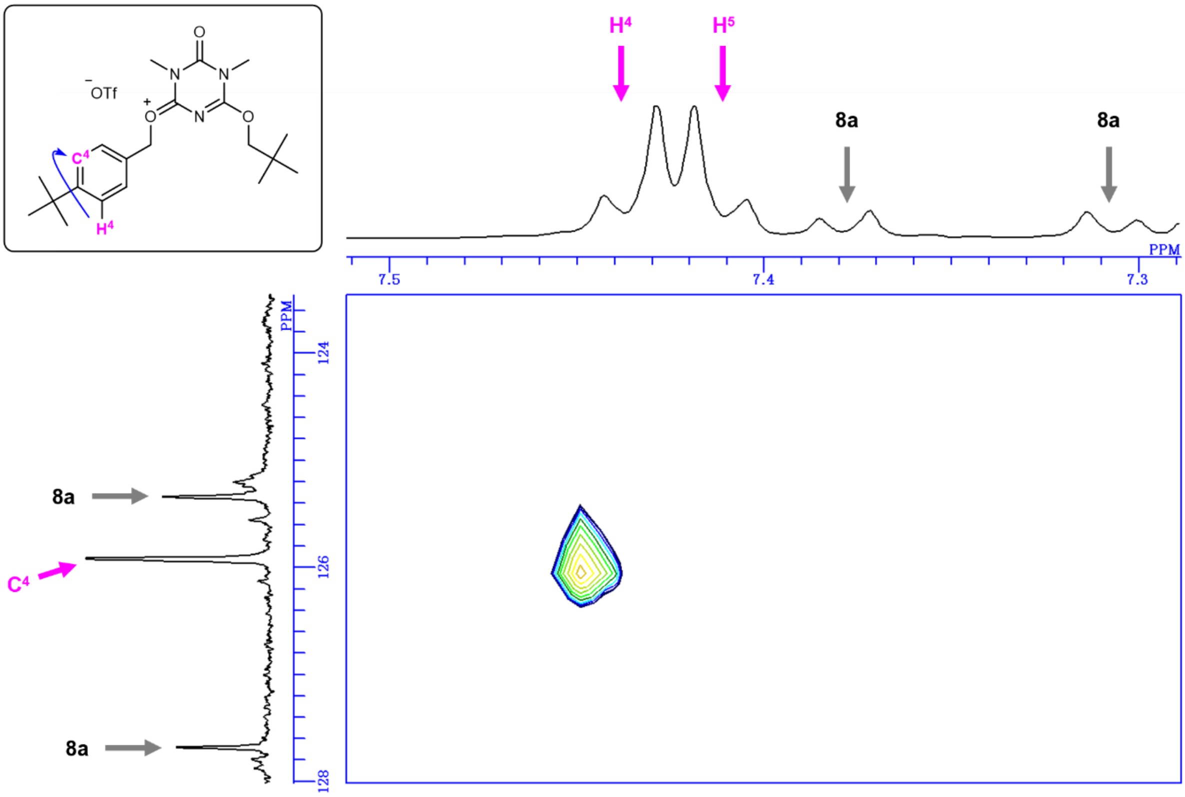

OTf
C4
H4
H5
8a
8a
PPM
7.5
7.4
7.3
124
126
128
8a
C4
8a

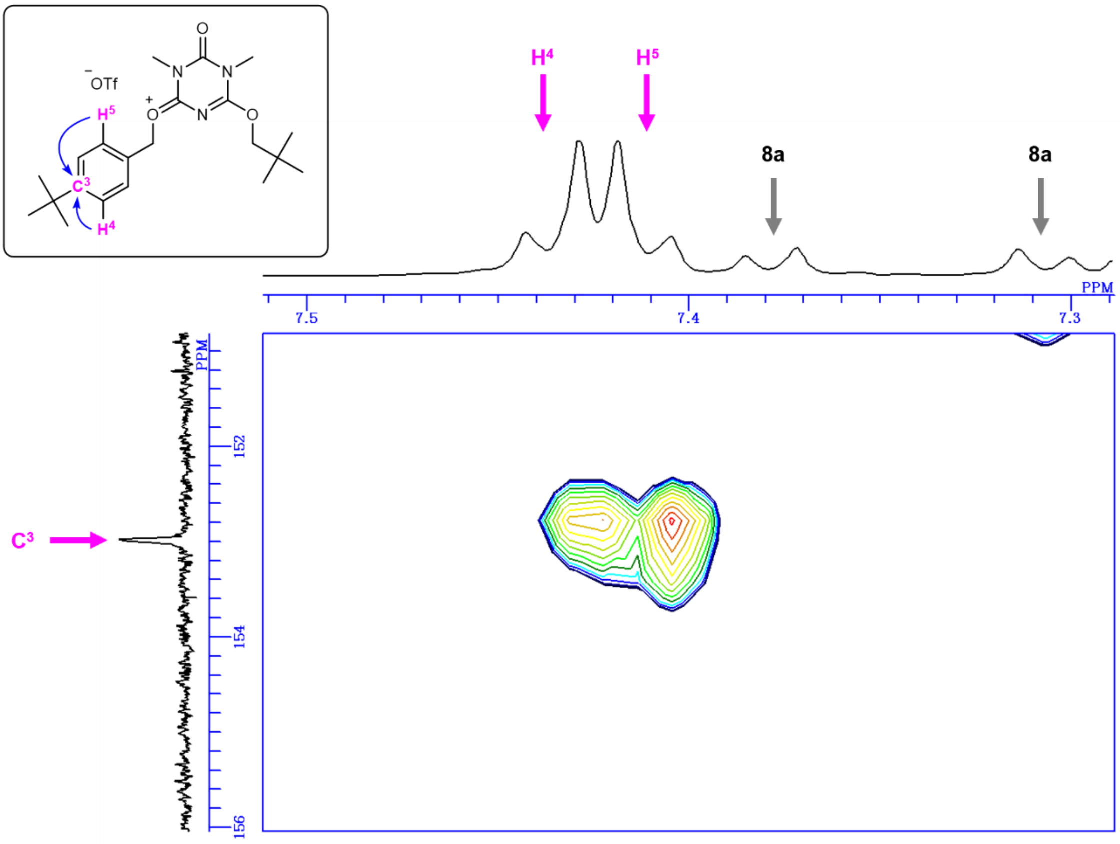
OTf
H5
C3
H4
H4
H5
8a
8a
PPM
7.5
7.4
7.3
152
154
156
C3

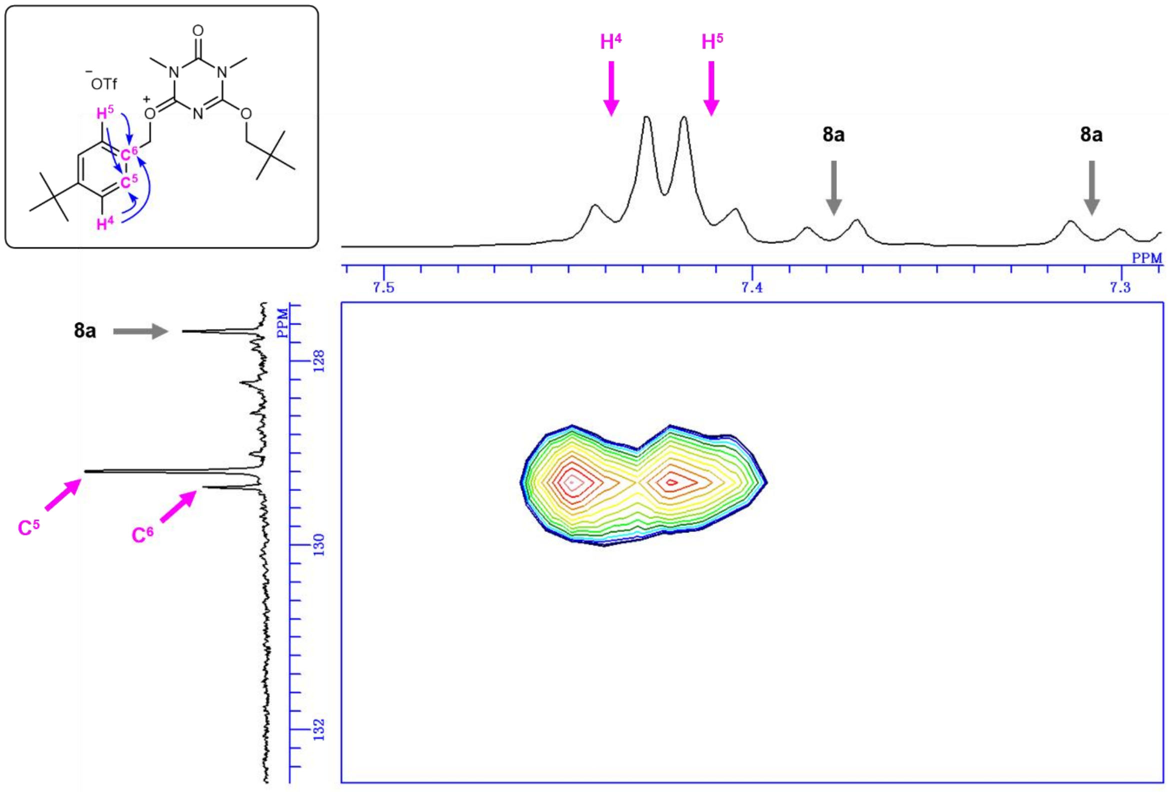
OTf
H5
C6
C5
H4
H4
H5
8a
8a
PPM
7.5
7.4
7.3
8a
128
130
132
C5
C6

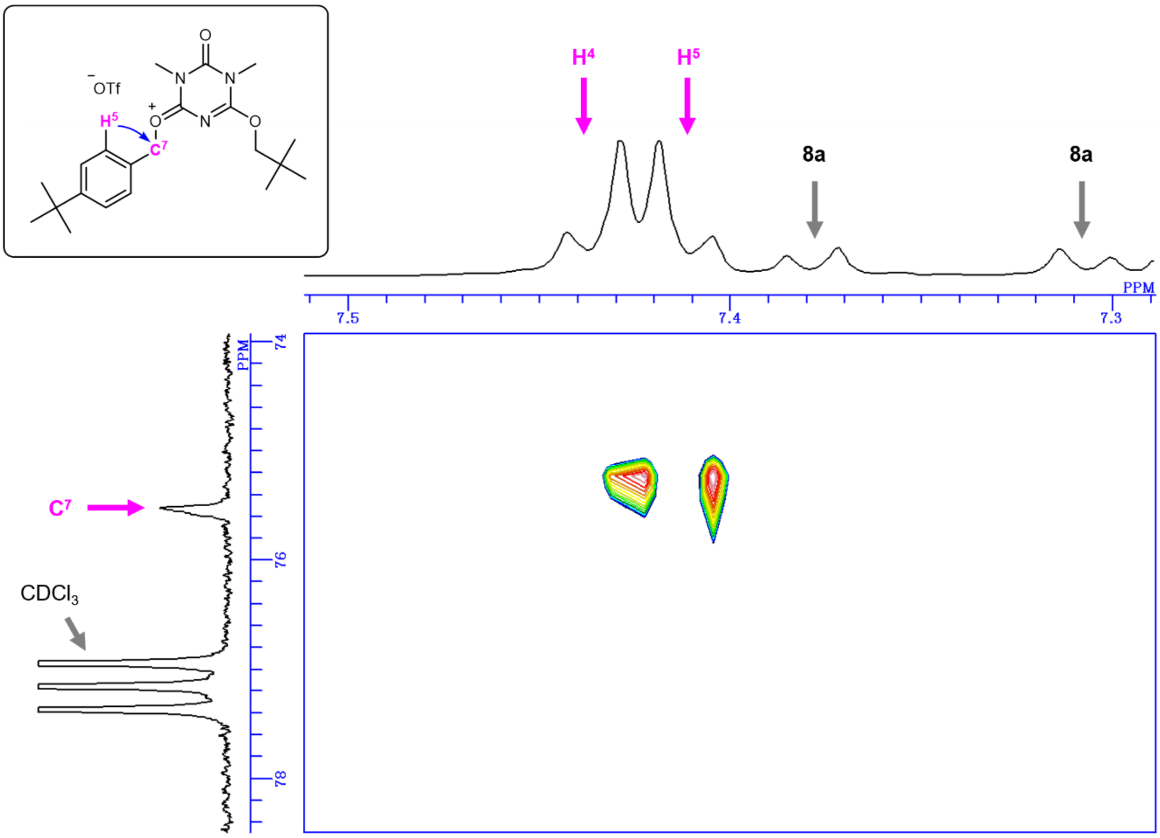

OTf
O
N
N
O
N
O
H5
C7
H4
H5
8a
8a
PPM
7.5
7.4
7.3
C7
CDCl3
PPM
74
76
78

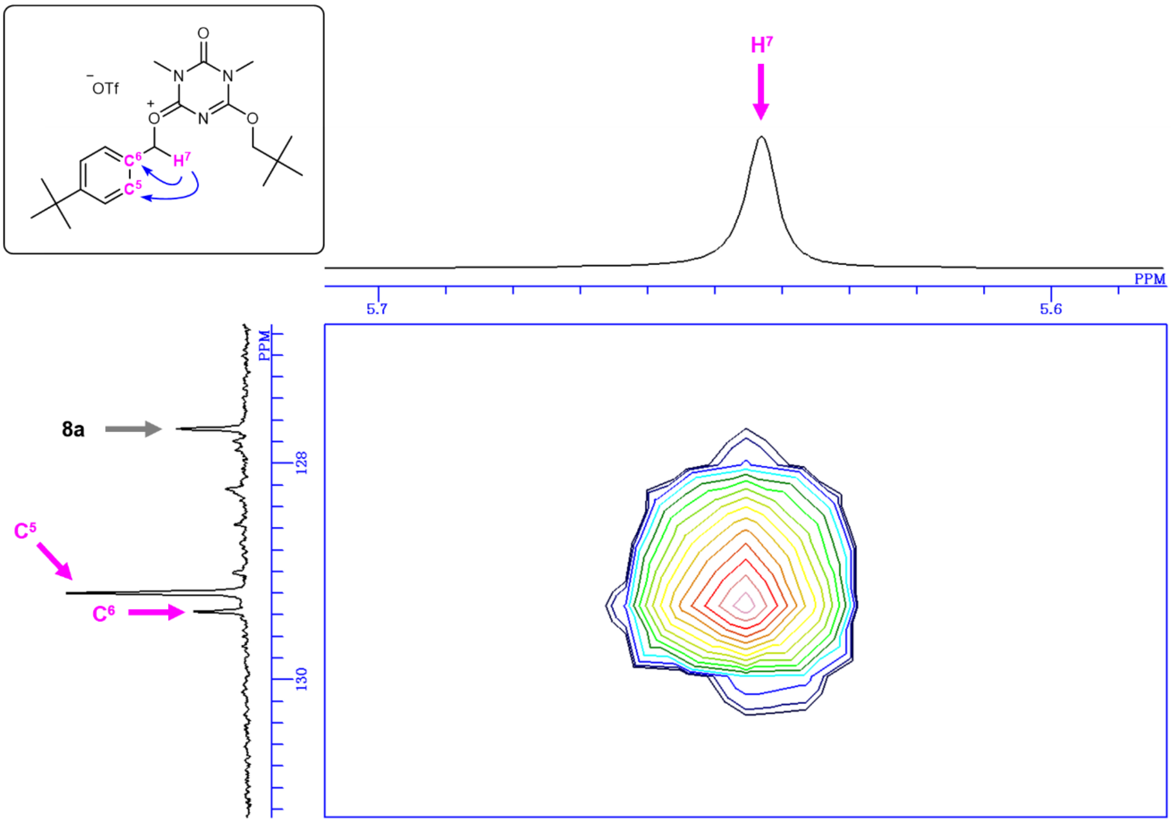

OTf
O
N
N
O
N
O
C6
C5
H7
H7
PPM
5.7
5.6
8a
C5
C6
PPM
128
130

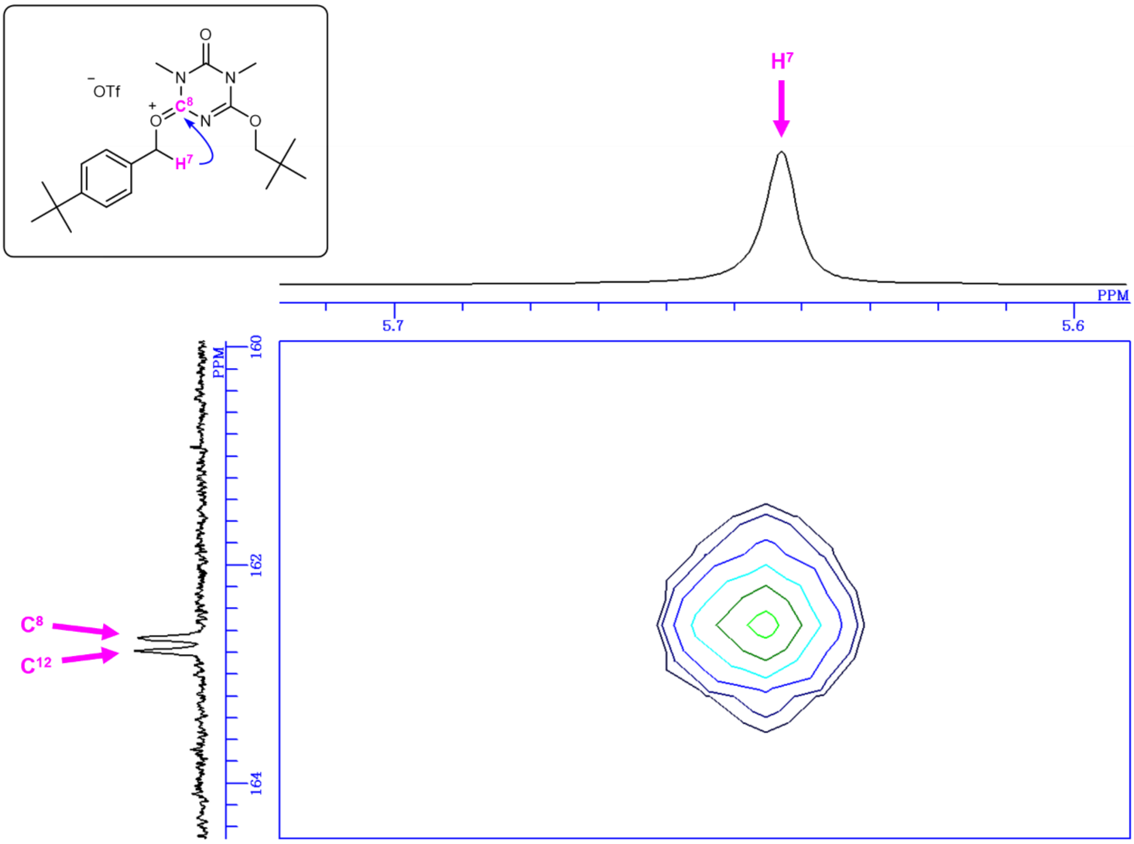

OTf
H7
C8
C12
PPM
5.7
5.6
160
162
164

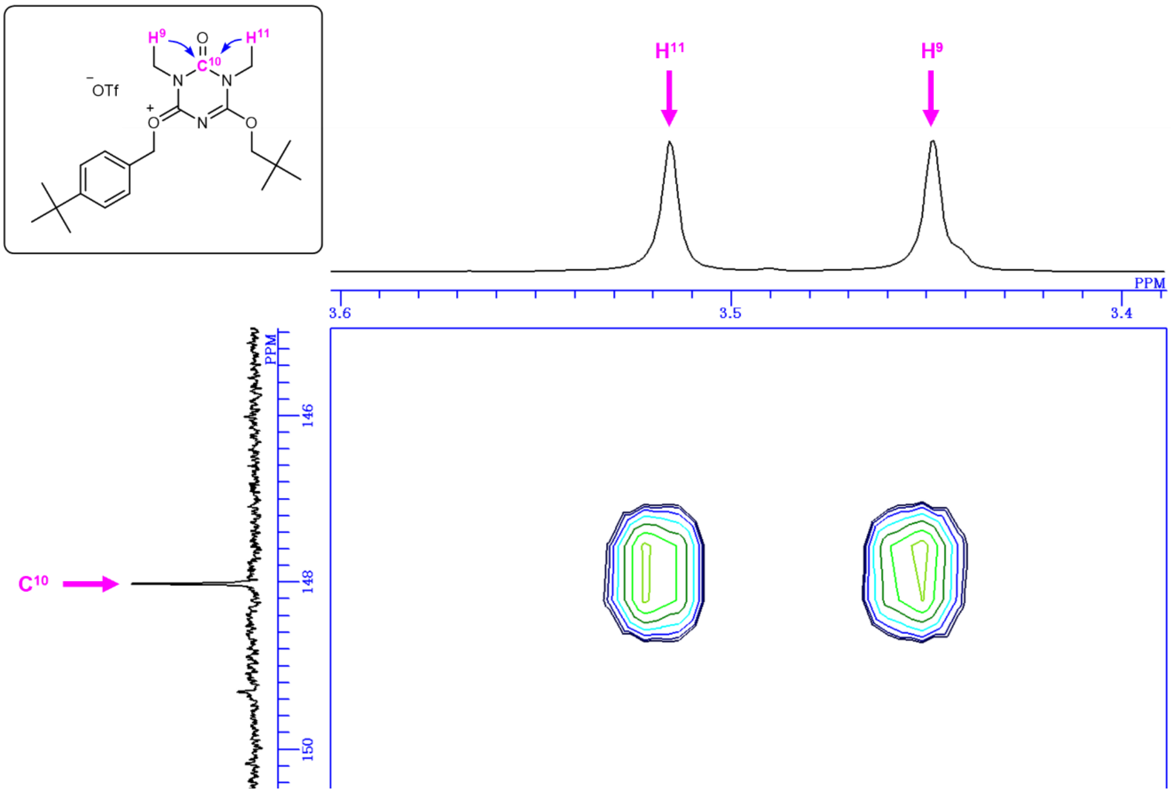

OTf
H9
H11
C10
PPM
3.6
3.5
3.4
146
148
150

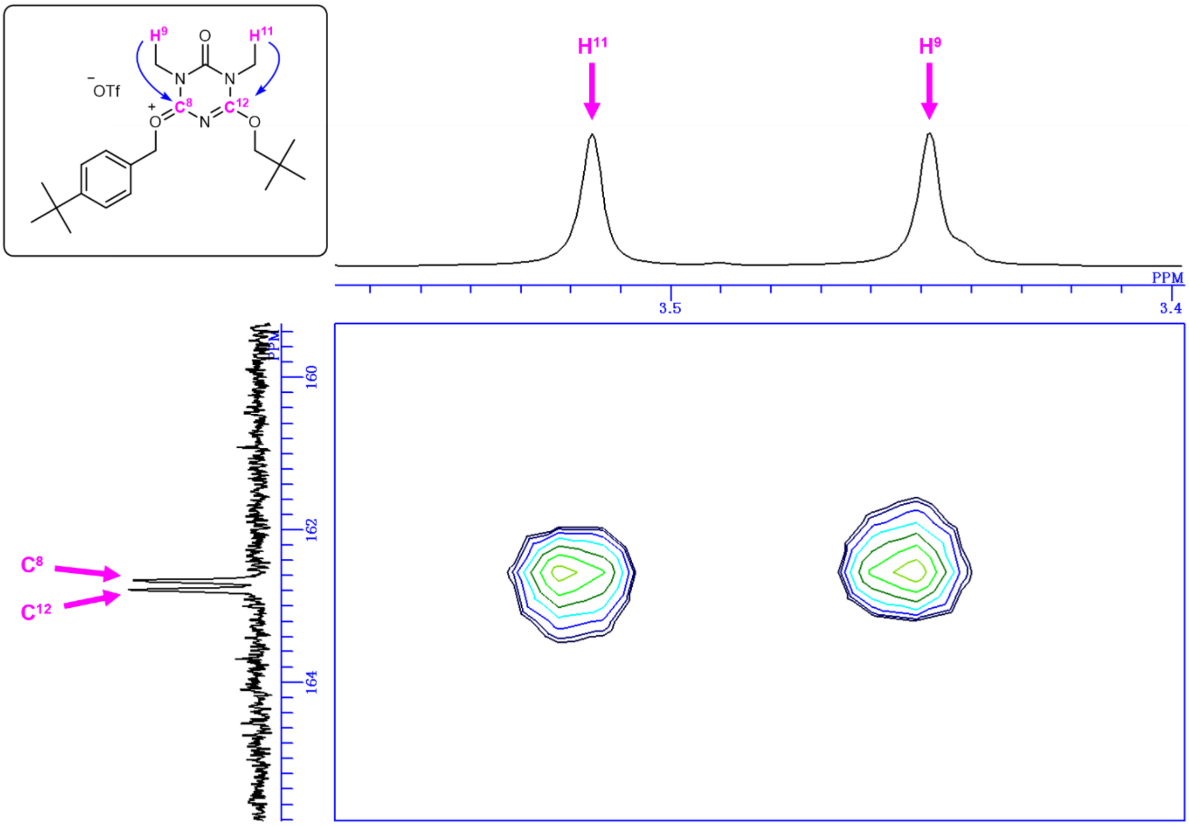

H9
H11
OTf
C8
C12
H11
H9
PPM
3.5
3.4
PPM
160
162
164
C8
C12

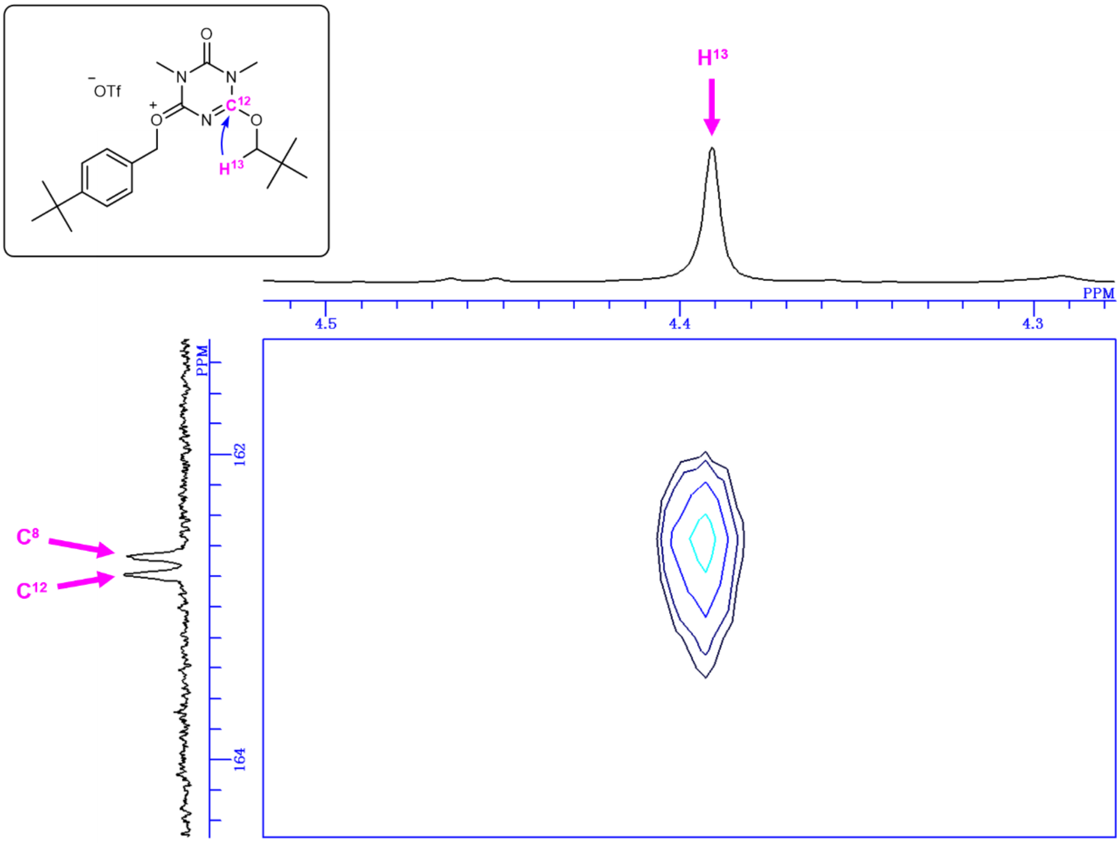

OTf
C12
H13
H13
PPM
4.5
4.4
4.3
PPM
162
164
C8
C12

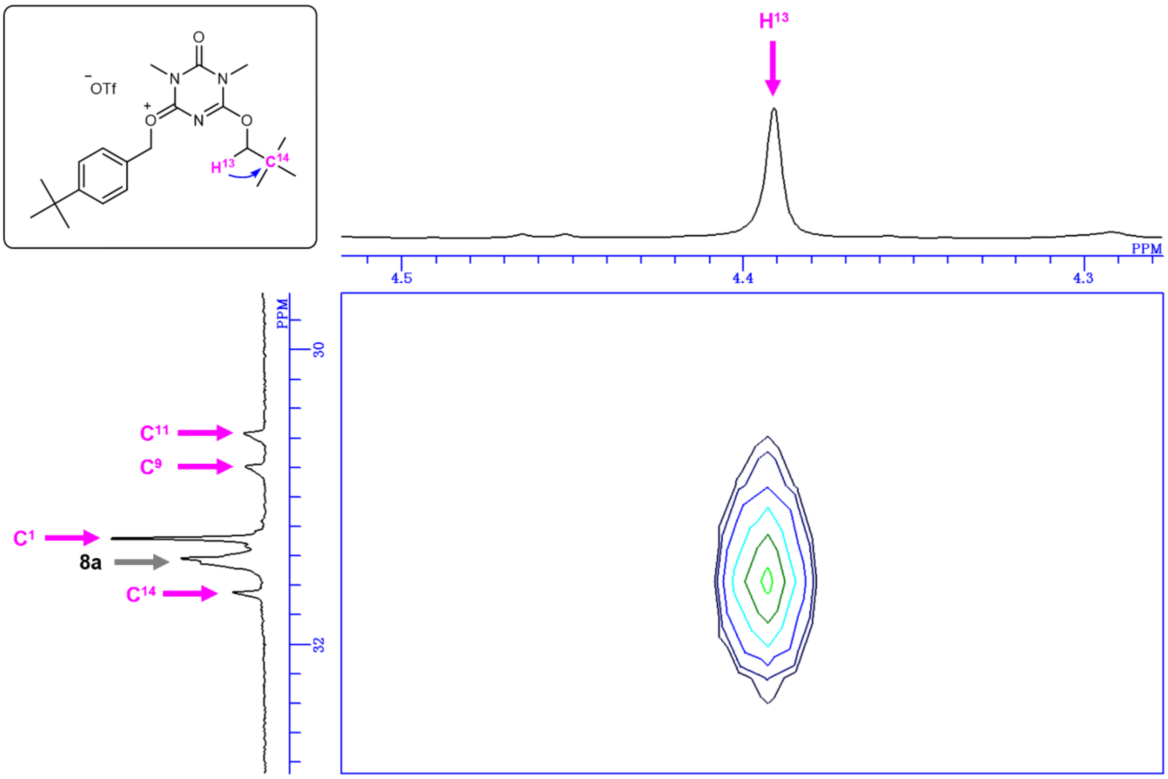

OTf
H13
C14
PPM
4.5
4.4
4.3
30
32
C11
C9
C1
8a

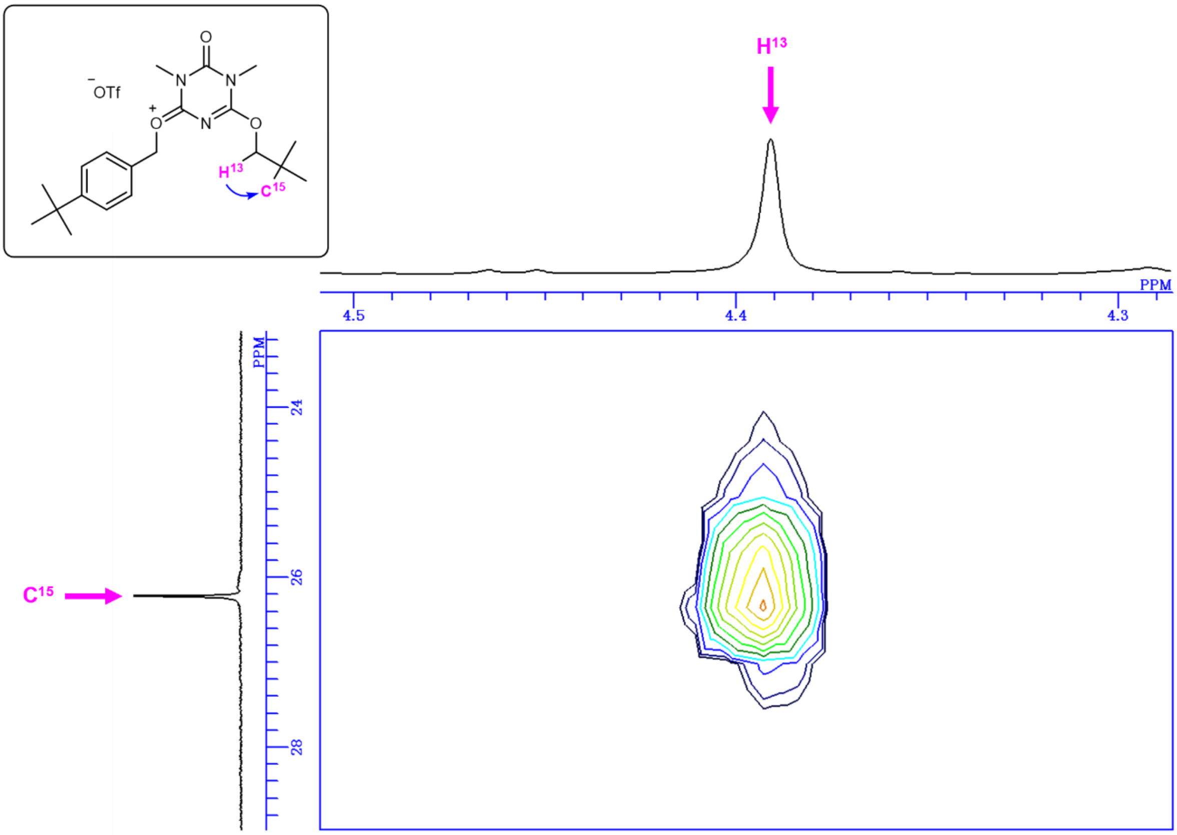

OTf
H13
C15
PPM
4.5
4.4
4.3
24
26
28

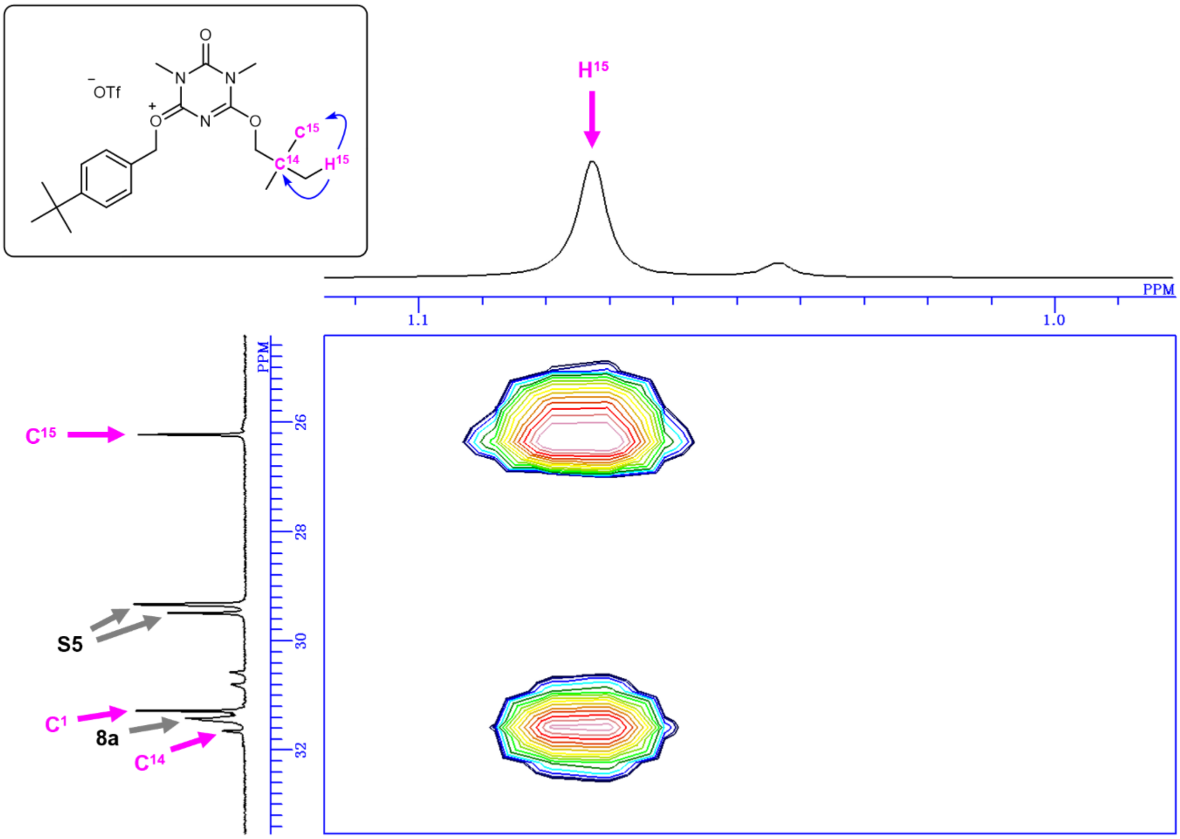

OTf
H15
C15
C14
PPM
1.1
1.0
26
28
30
32
S5
C1
8a
C14

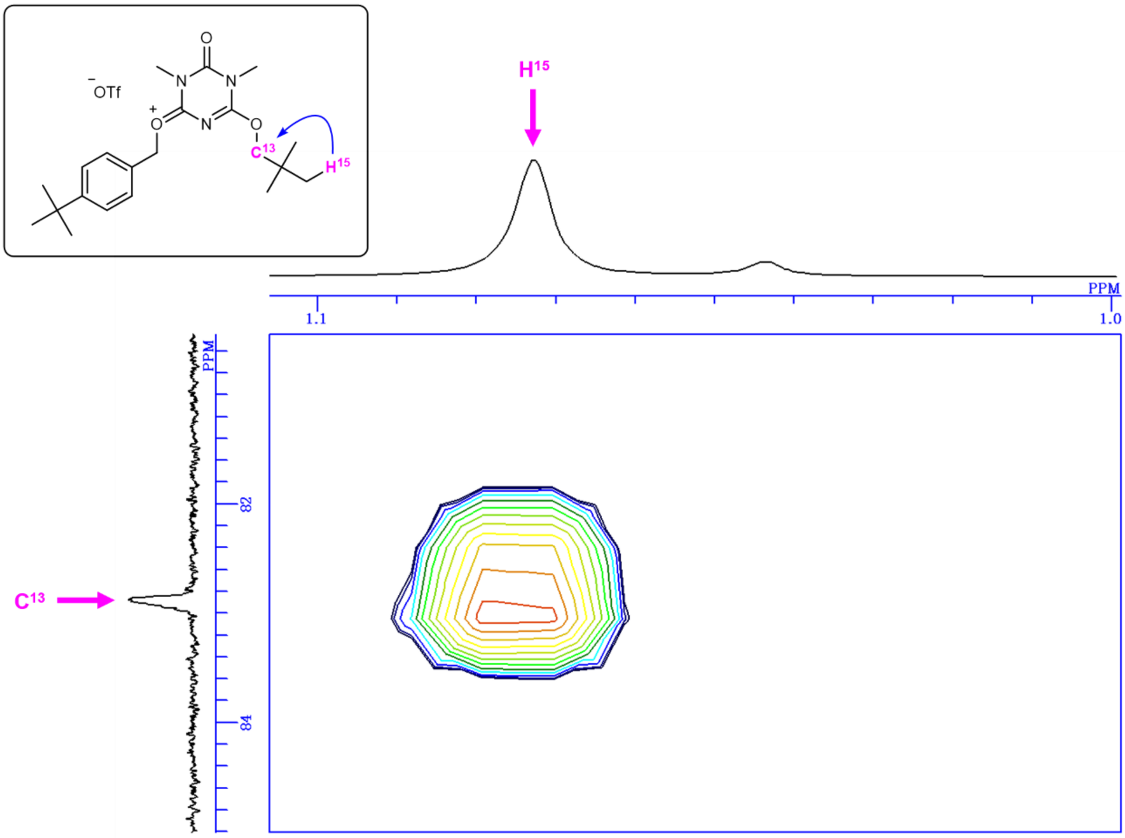

OTf
H15
C13
PPM
1.1
1.0
82
84

HMQC spectrum of a $CDCl_3$ solution containing carbocationoid **3b**

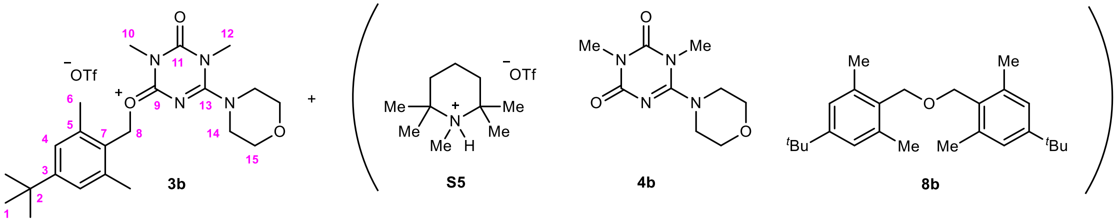

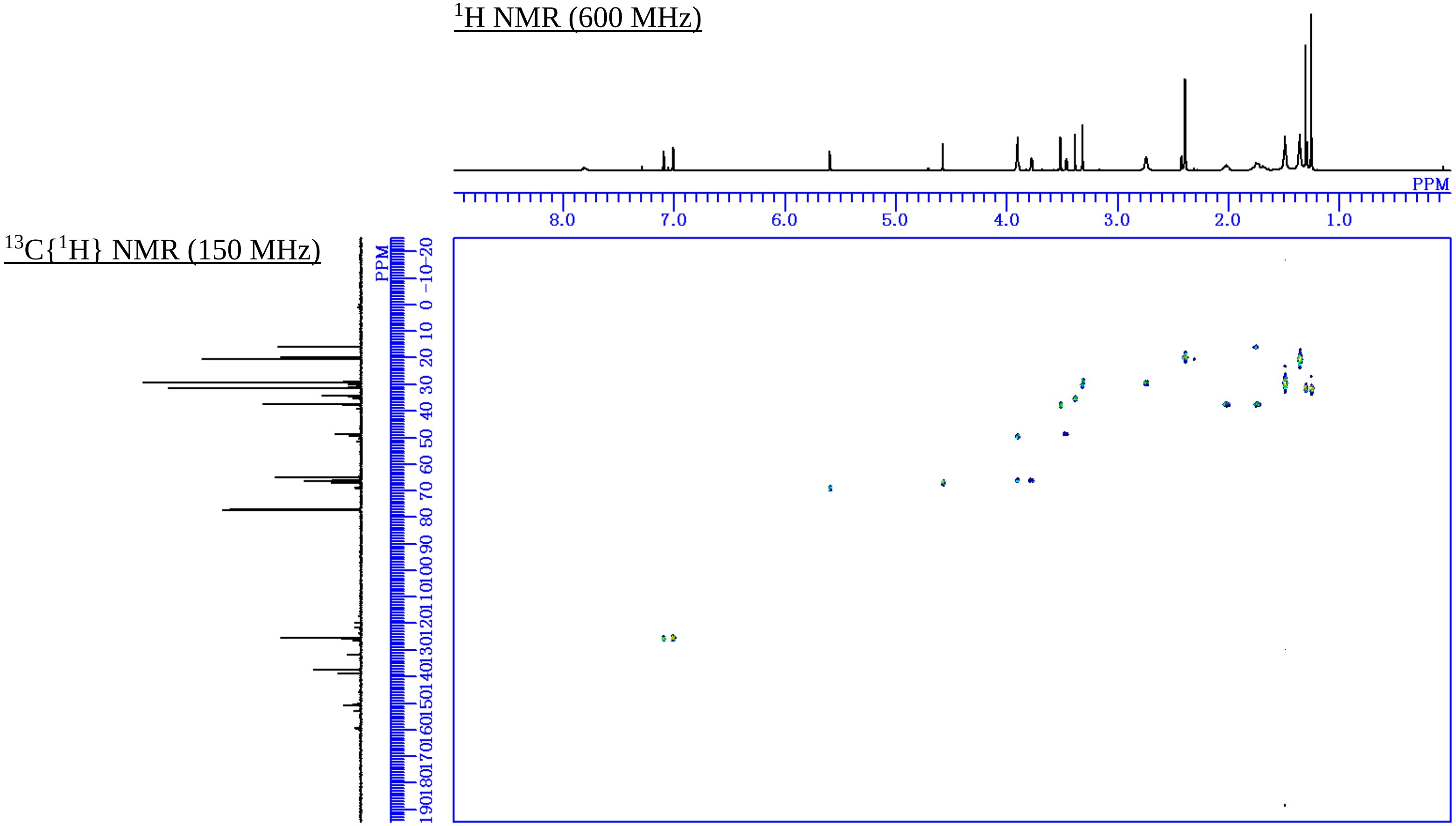

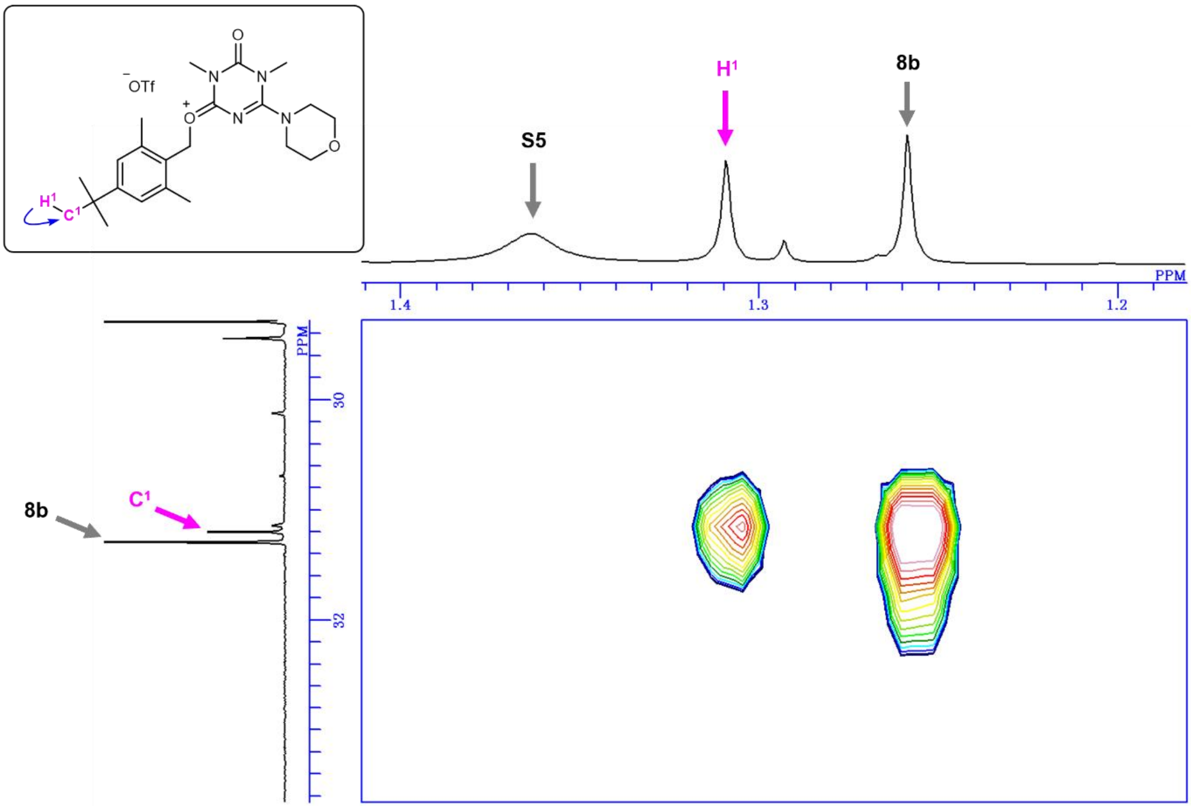

OTf
H1
C1
S5
H1
8b
PPM
1.4
1.3
1.2
30
32
8b
C1

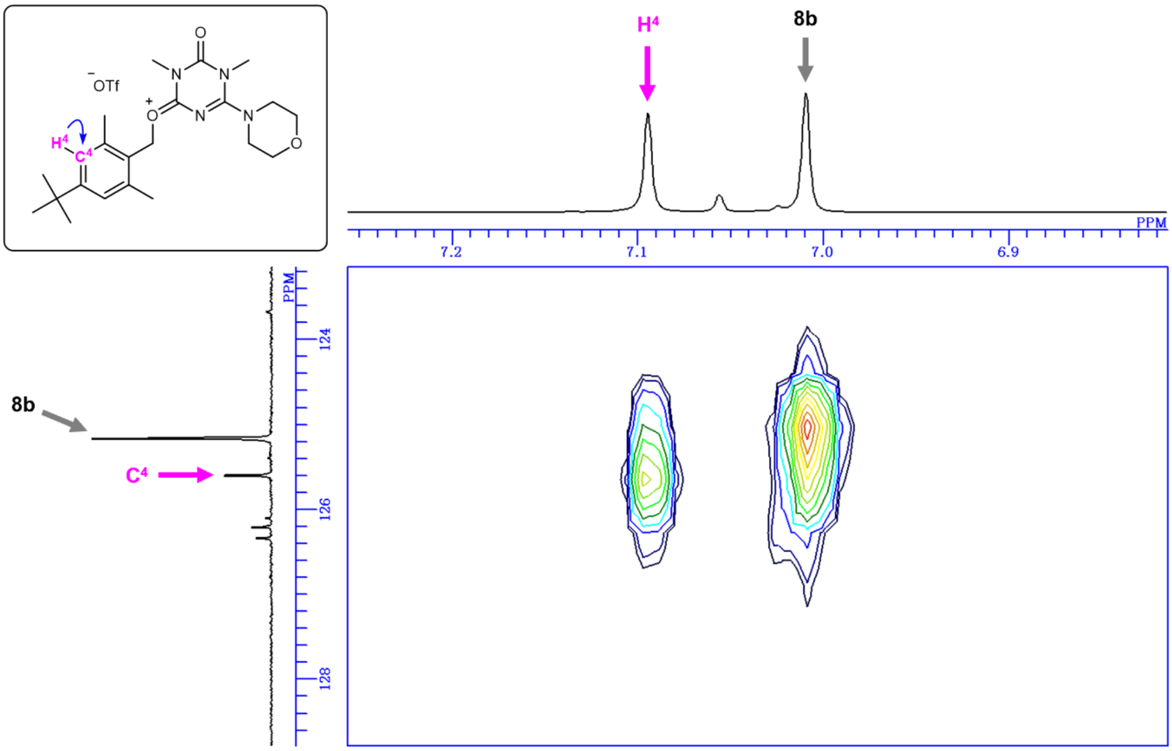

OTf
H4
C4
H4
8b
PPM
7.2
7.1
7.0
6.9
124
126
128
8b
C4

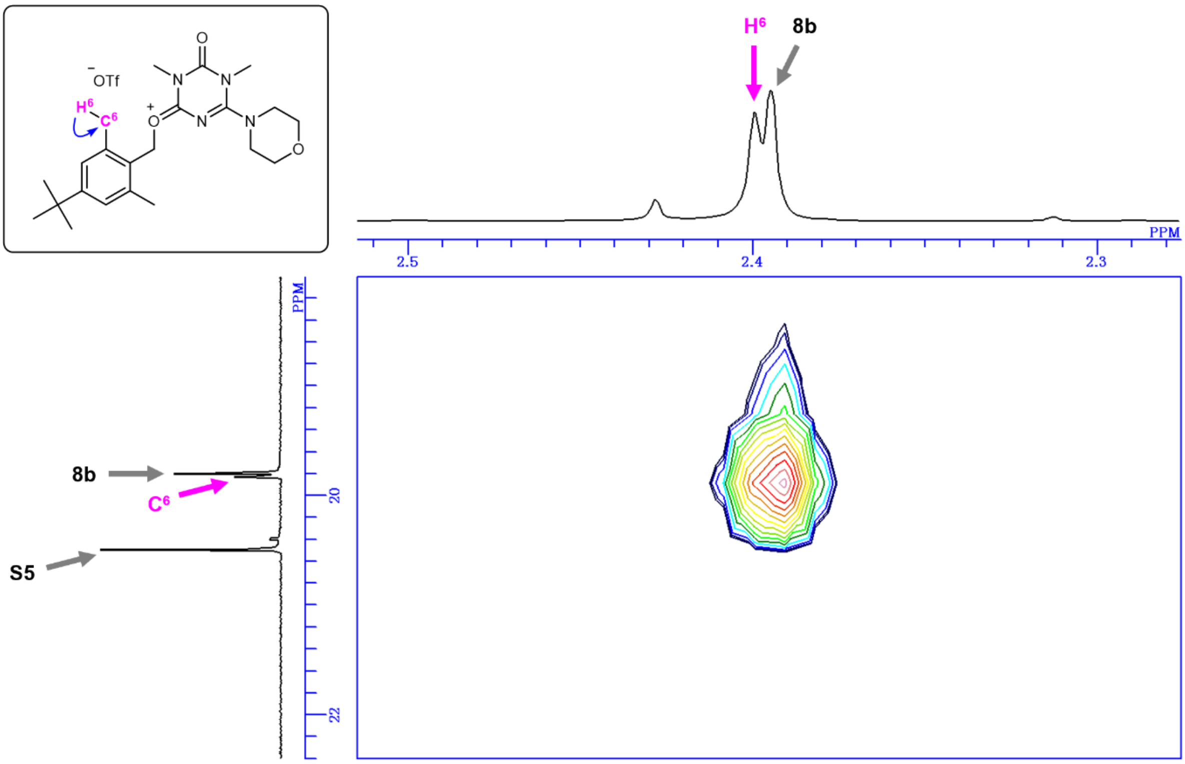

OTf
H6
C6
8b
PPM
2.5
2.4
2.3
20
22
C6
S5

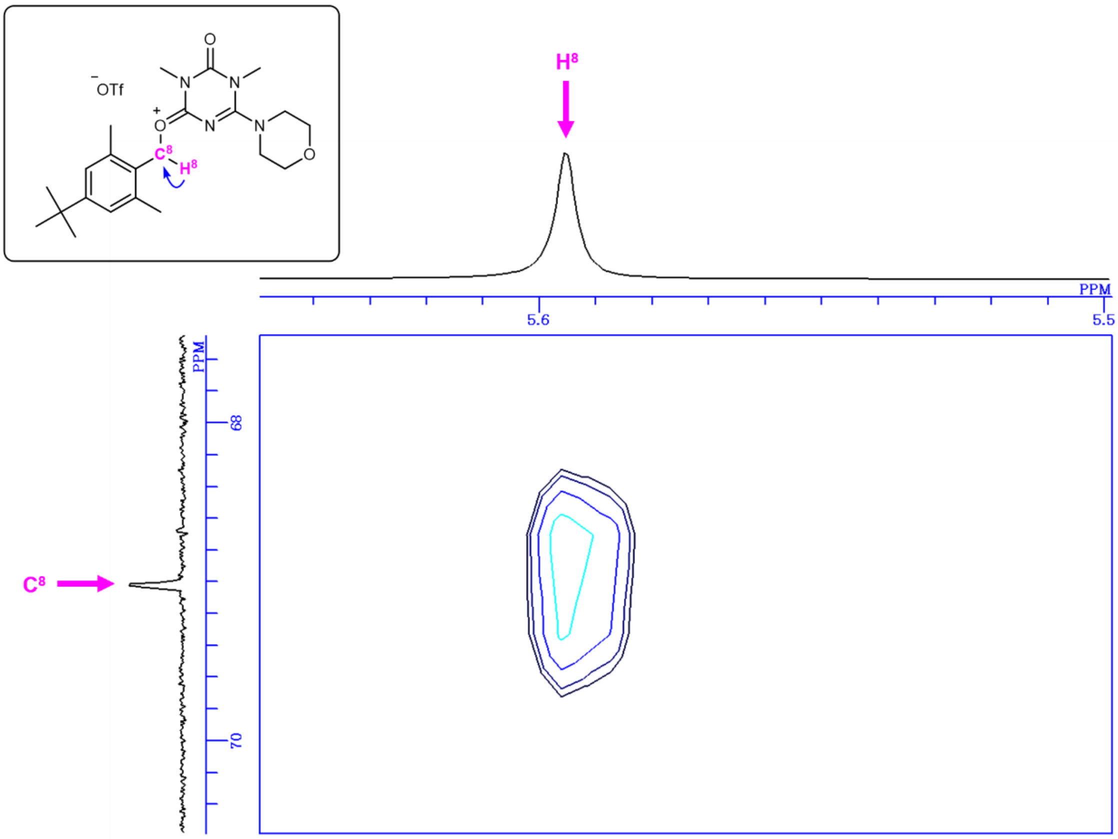

OTf
C8
H8
PPM
5.6
5.5
68
70
C8

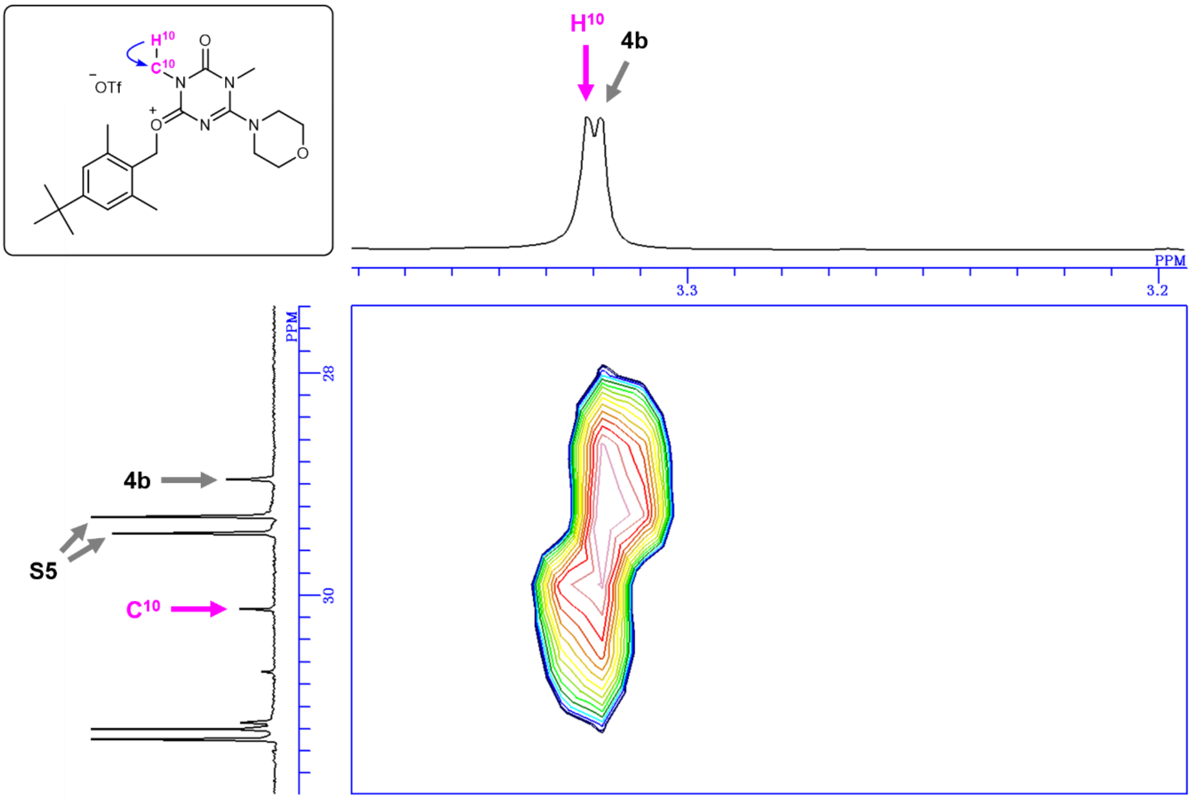

H10
C10
OTf
H10
4b
PPM
3.3
3.2
28
30
S5
C10

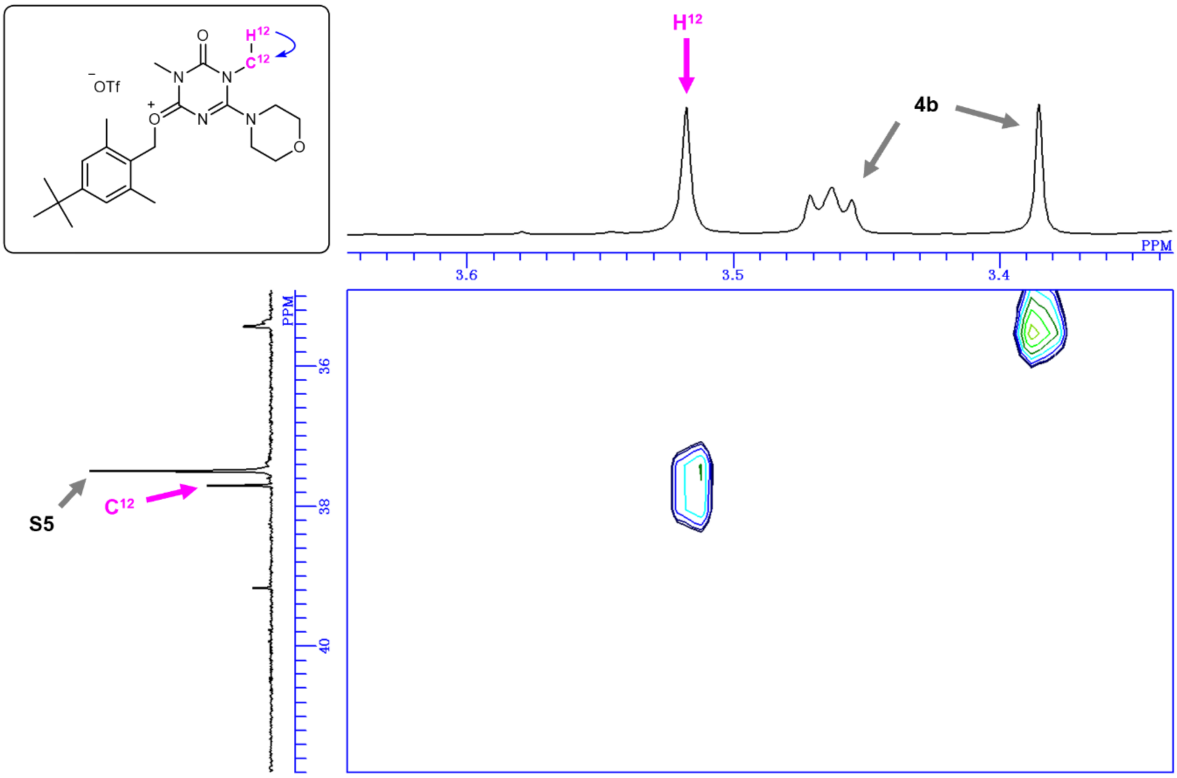

H12
C12
OTf
H12
4b
PPM
3.6
3.5
3.4
36
38
40
S5
C12

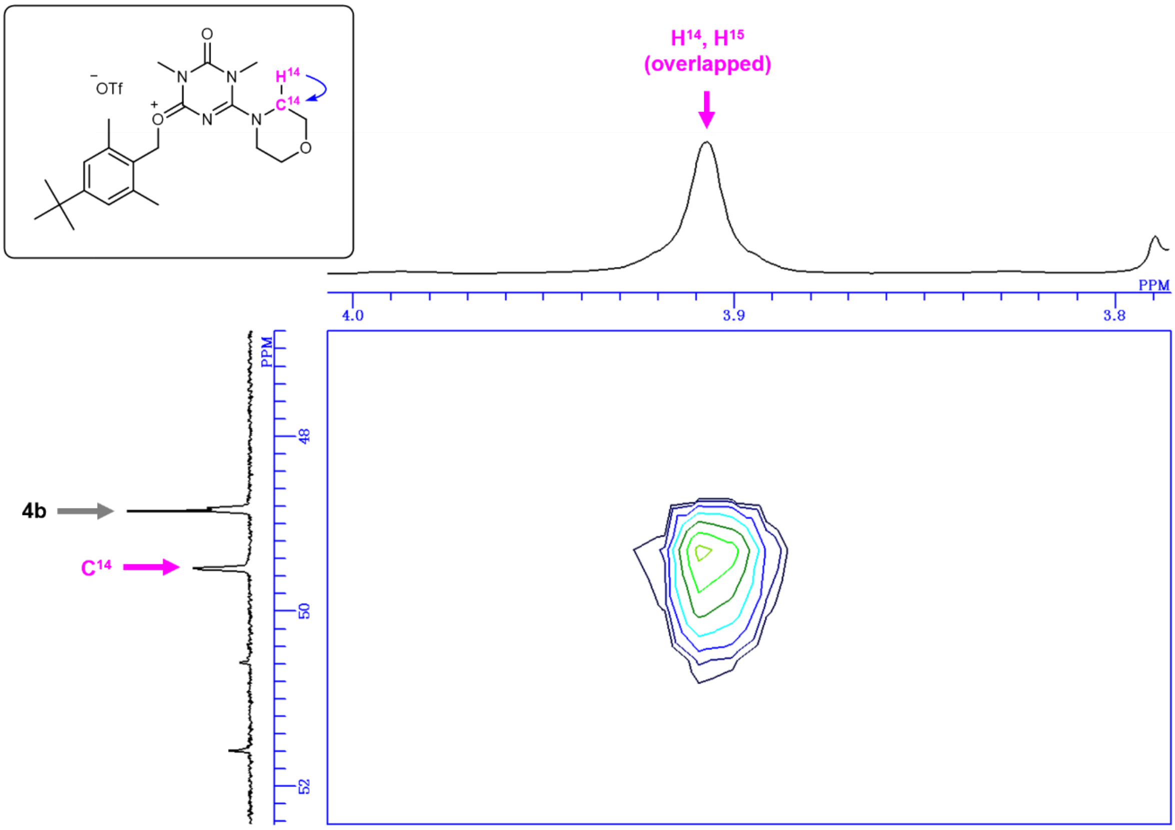

OTf
O
N
N
N
N
O
H14
C14
O
H14, H15
(overlapped)
PPM
4.0
3.9
3.8
PPM
48
50
52
4b
C14

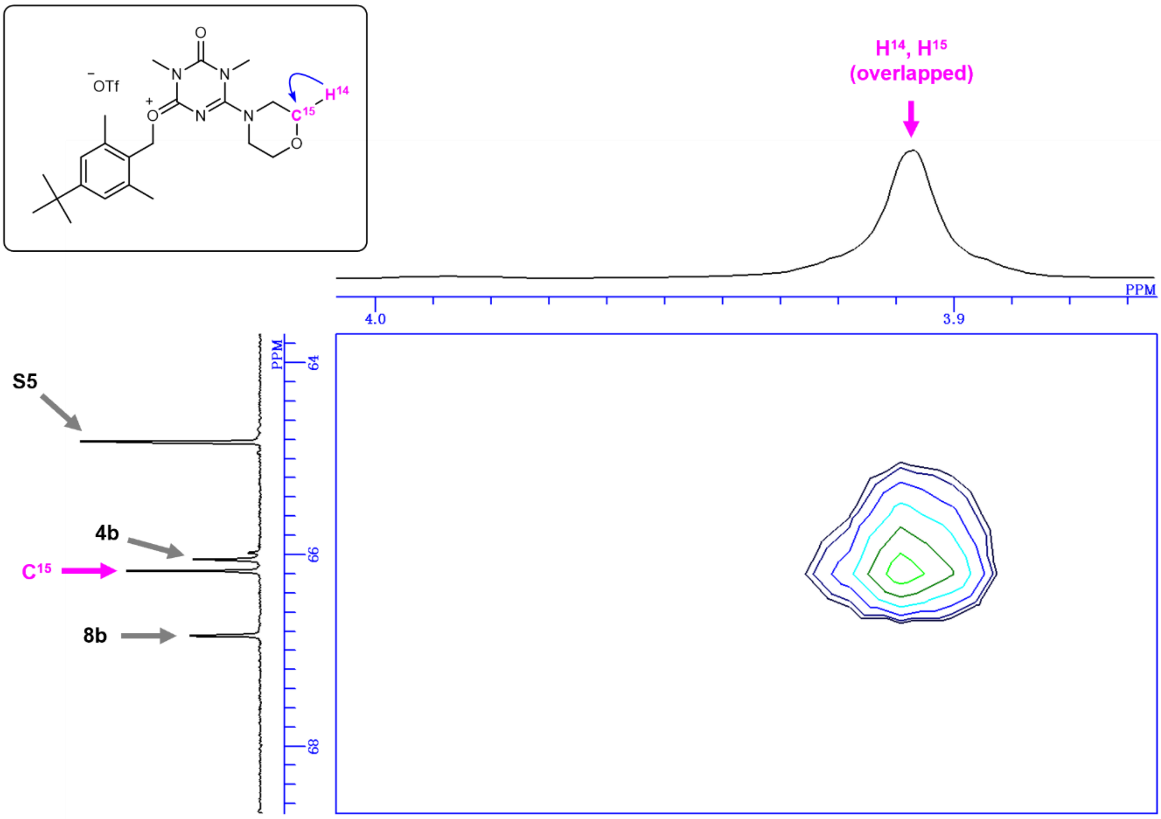

OTf
O
N
N
N
N
O
H14
C15
O
H14, H15
(overlapped)
PPM
4.0
3.9
PPM
64
66
68
S5
4b
C15
8b

HMBC spectrum of a $CDCl_3$ solution containing carbocationoid **3b**

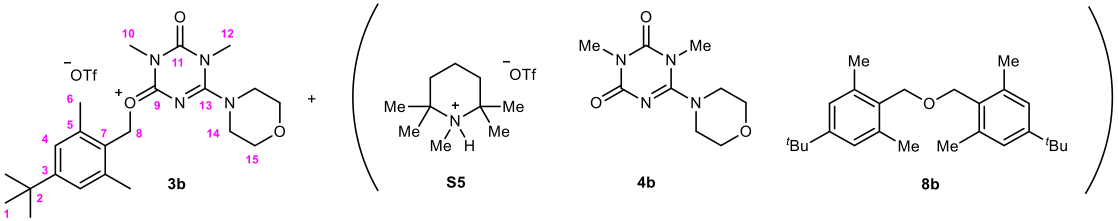

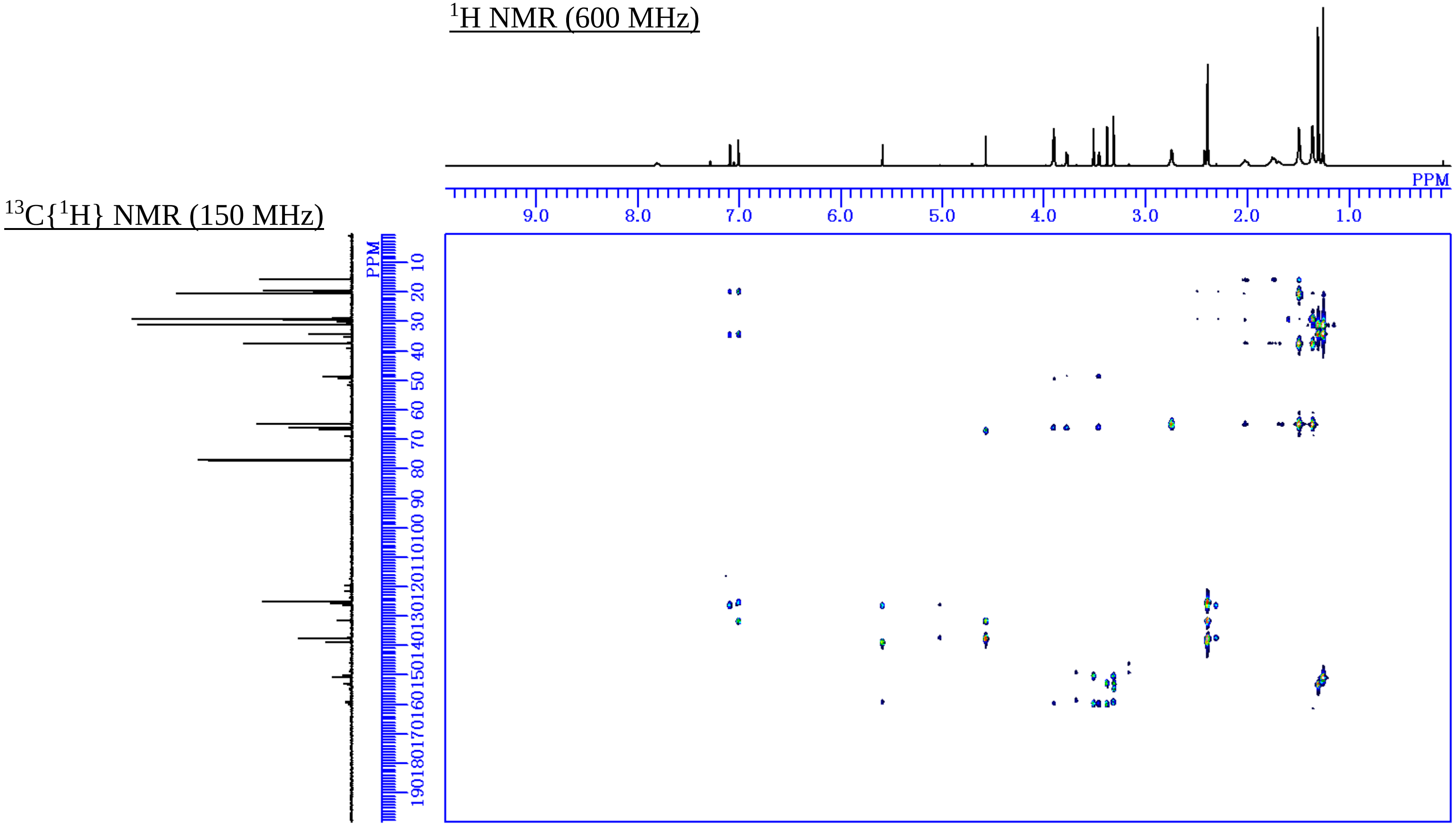

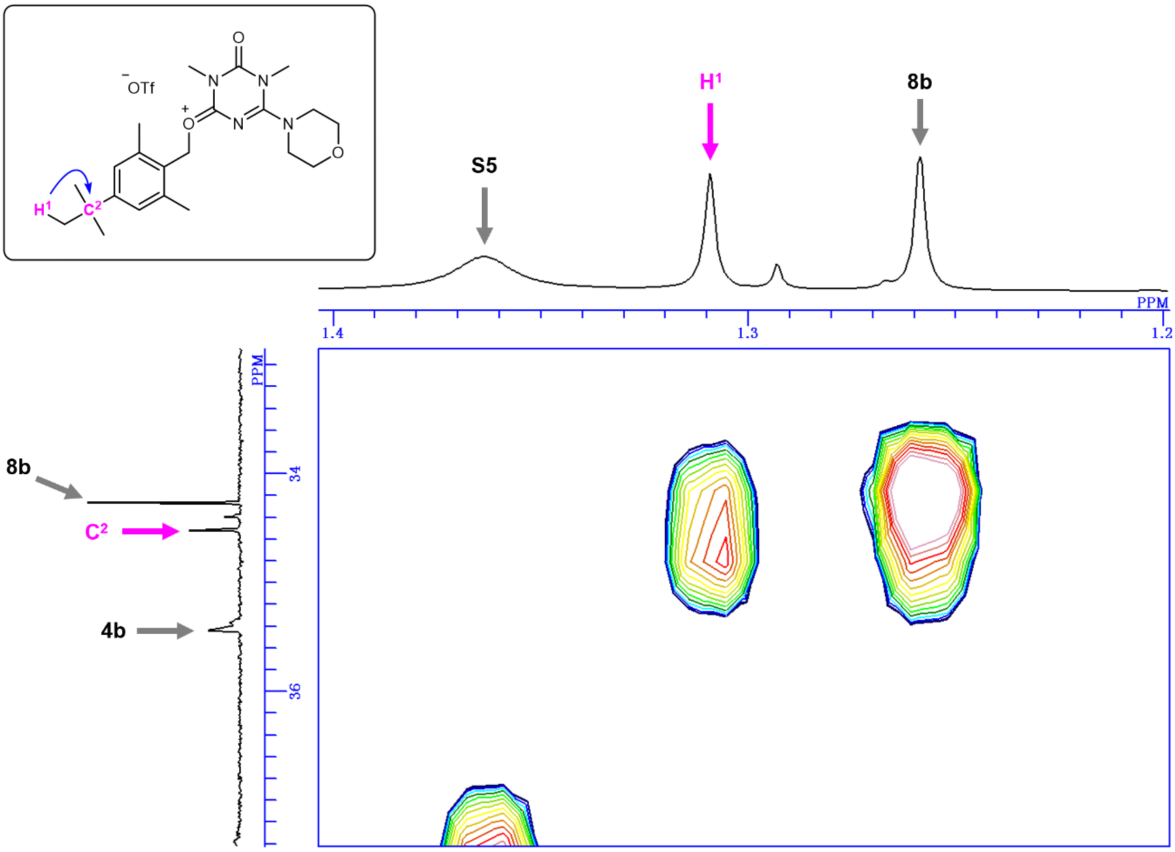

OTf
H1
C2
S5
H1
8b
PPM
1.4
1.3
1.2
8b
C2
4b
34
36

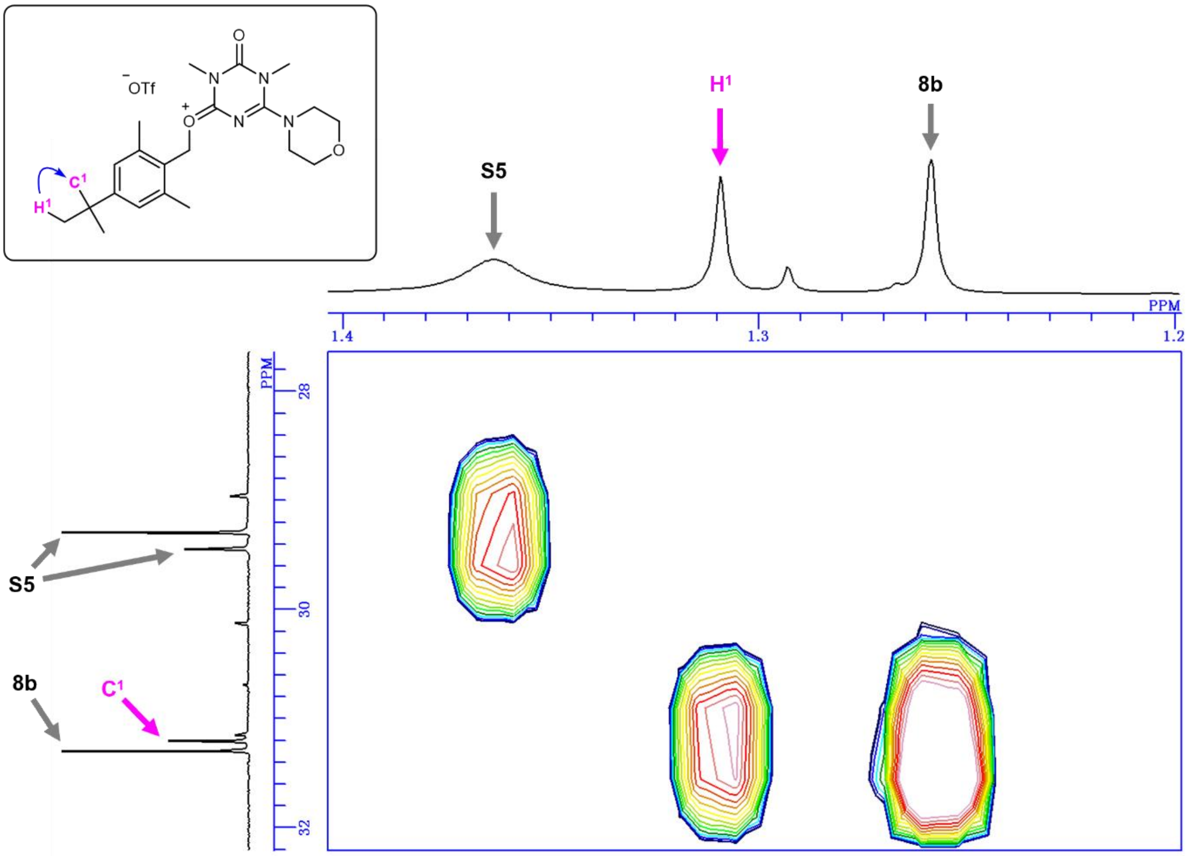

OTf
C1
H1
S5
H1
8b
PPM
1.4
1.3
1.2
28
30
32
S5
8b
C1

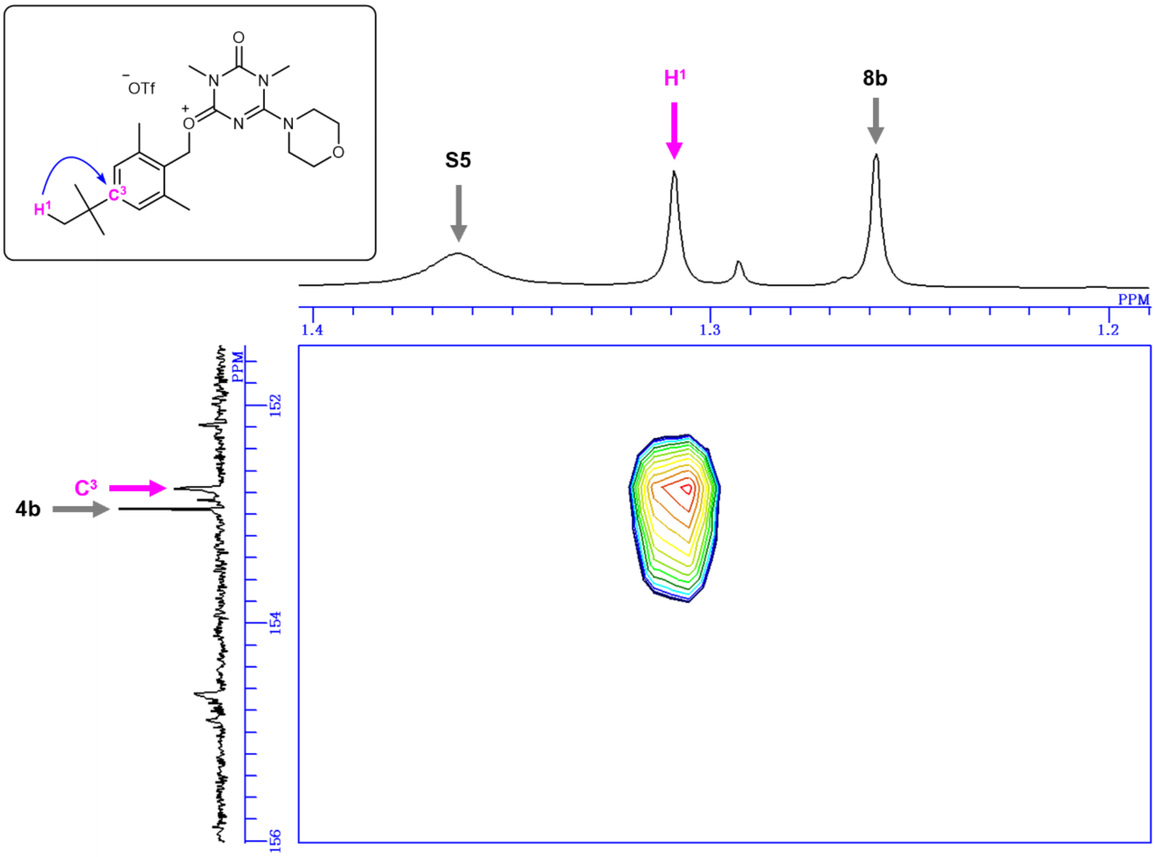

OTf
H1
C3
S5
8b
PPM
1.4
1.3
1.2
152
154
156
4b

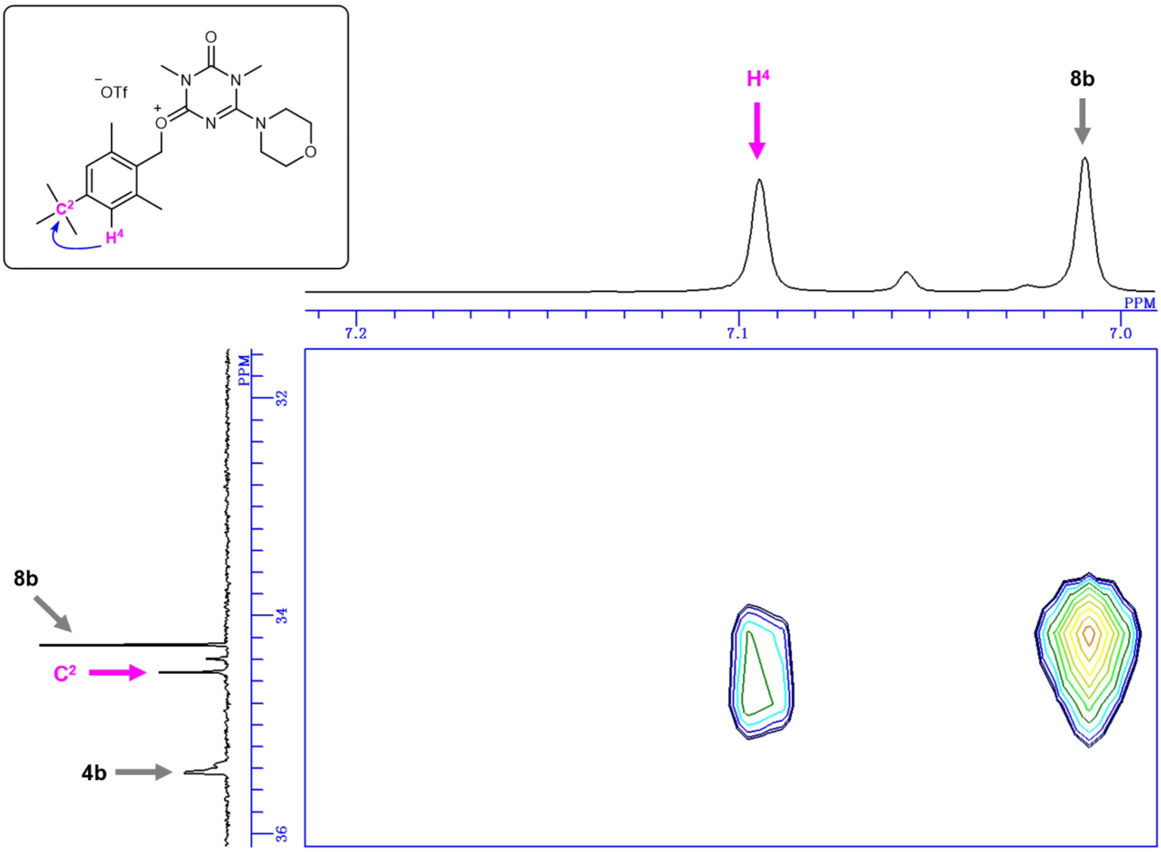

OTf
C2
H4
8b
PPM
7.2
7.1
7.0
32
34
36
4b

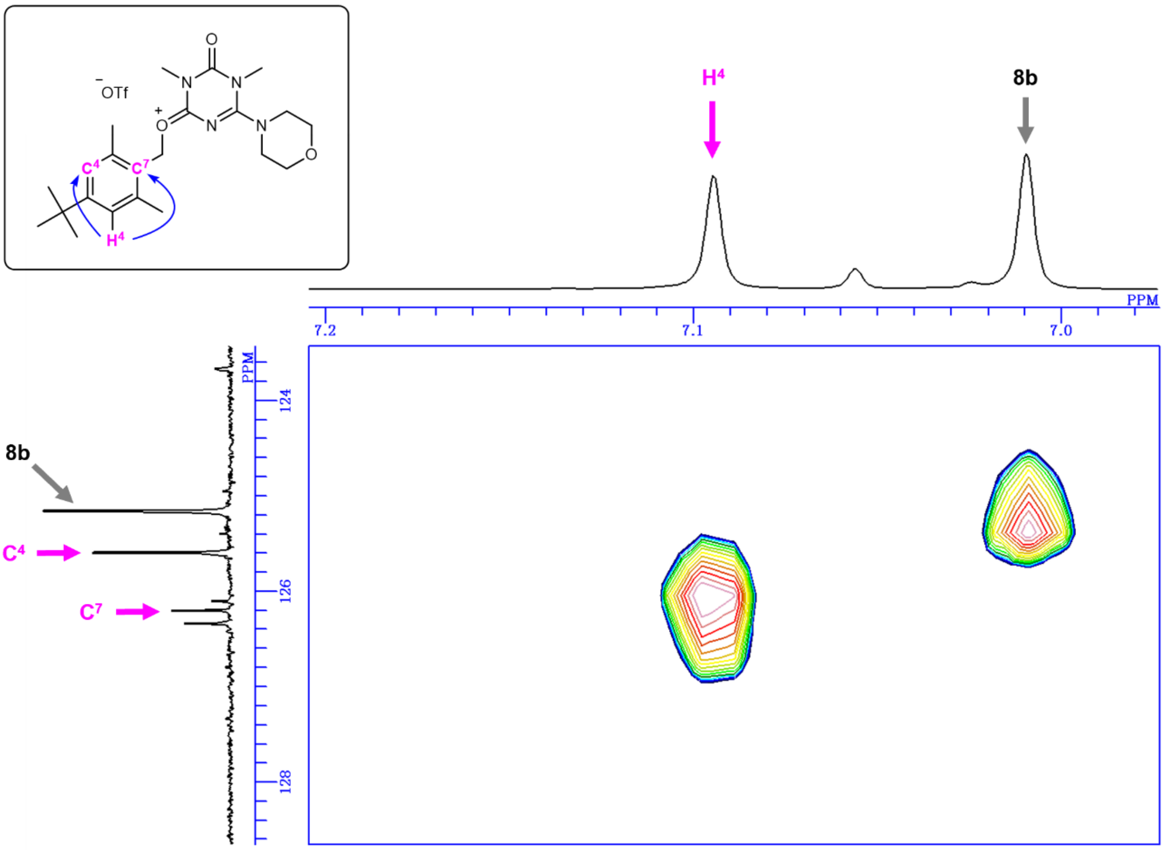

−OTf
H4
C4
C7
8b
PPM
7.2
7.1
7.0
124
126
128

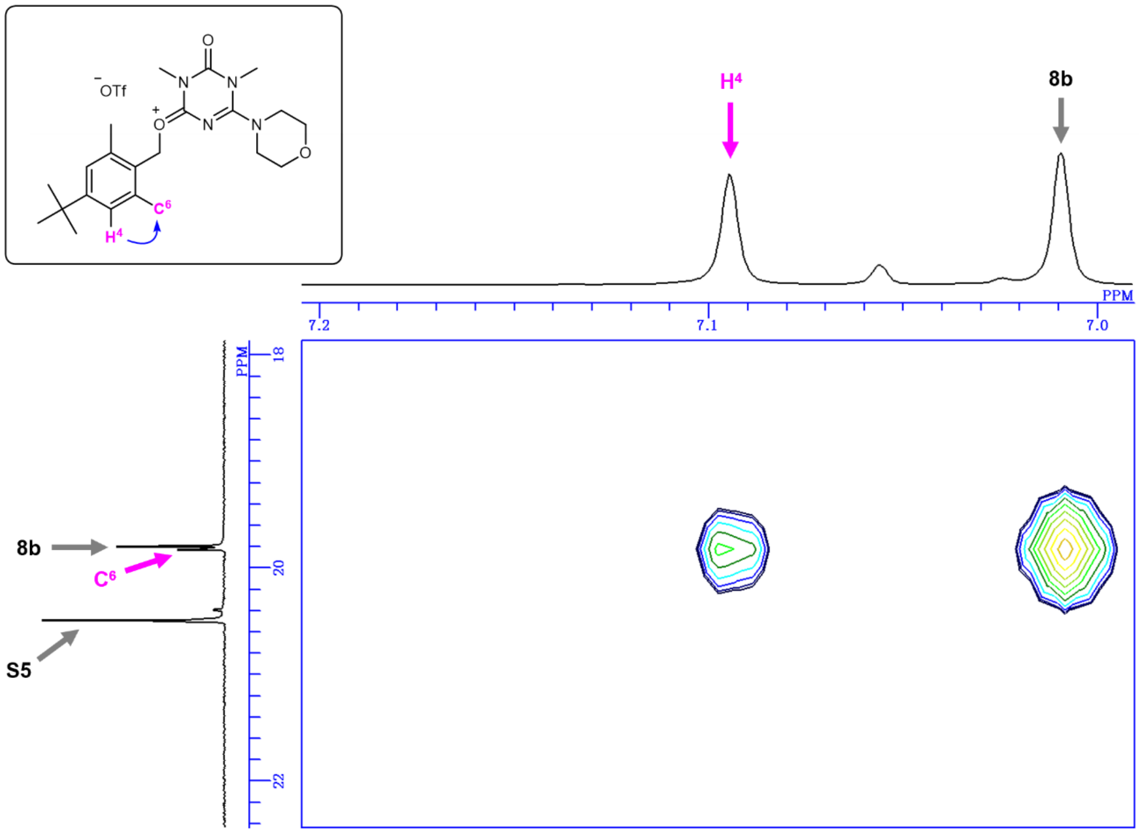

−OTf
H4
C6
8b
S5
PPM
7.2
7.1
7.0
18
20
22

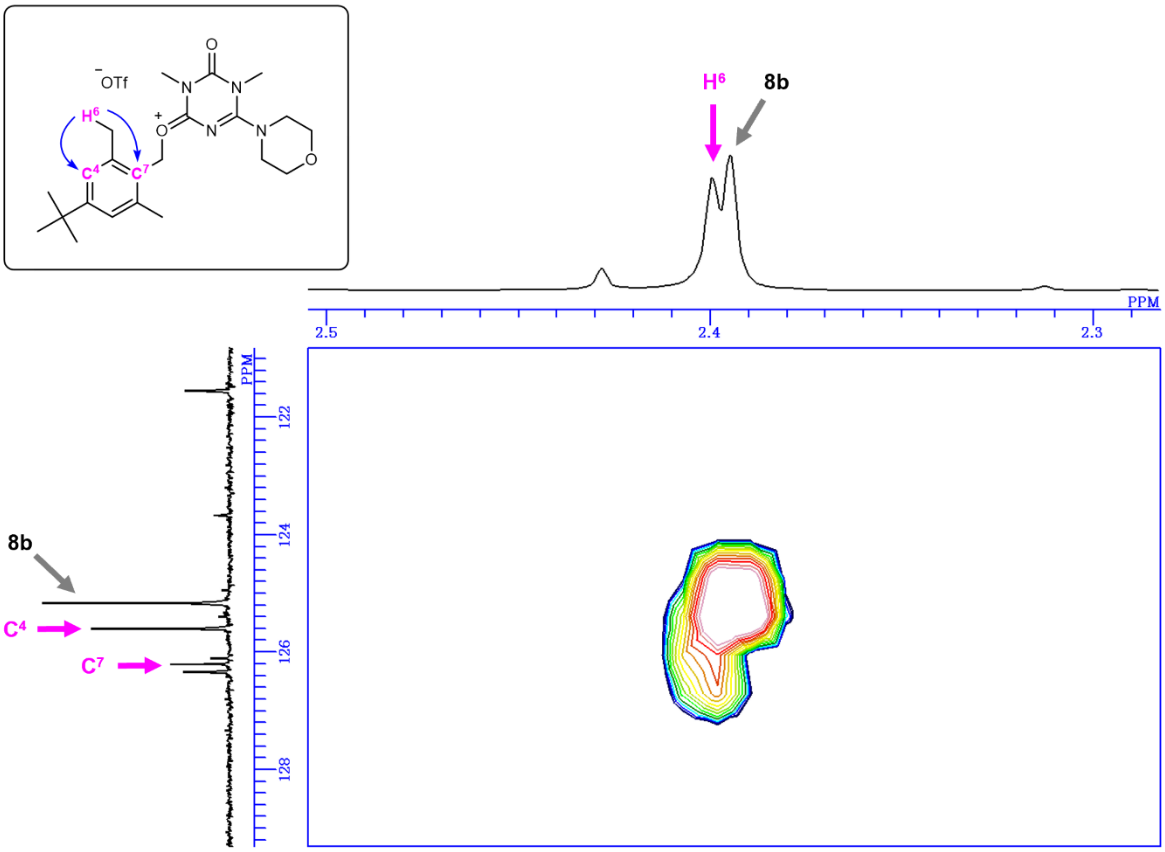

OTf
O
N
N
N
N
O
O
H6
C4
C7
H6
8b
PPM
2.5
2.4
2.3
PPM
122
124
126
128
8b
C4
C7

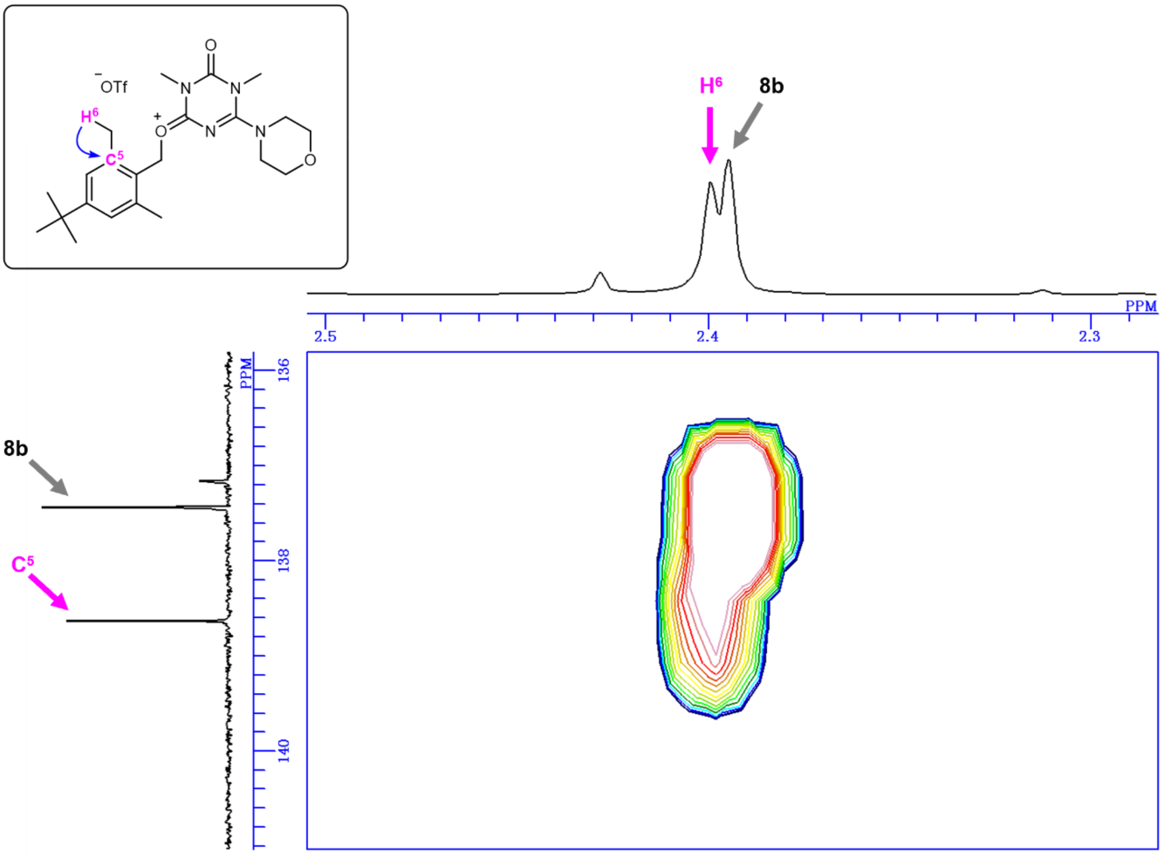

OTf
O
N
N
N
N
O
O
H6
C5
H6
8b
PPM
2.5
2.4
2.3
PPM
136
138
140
8b
C5

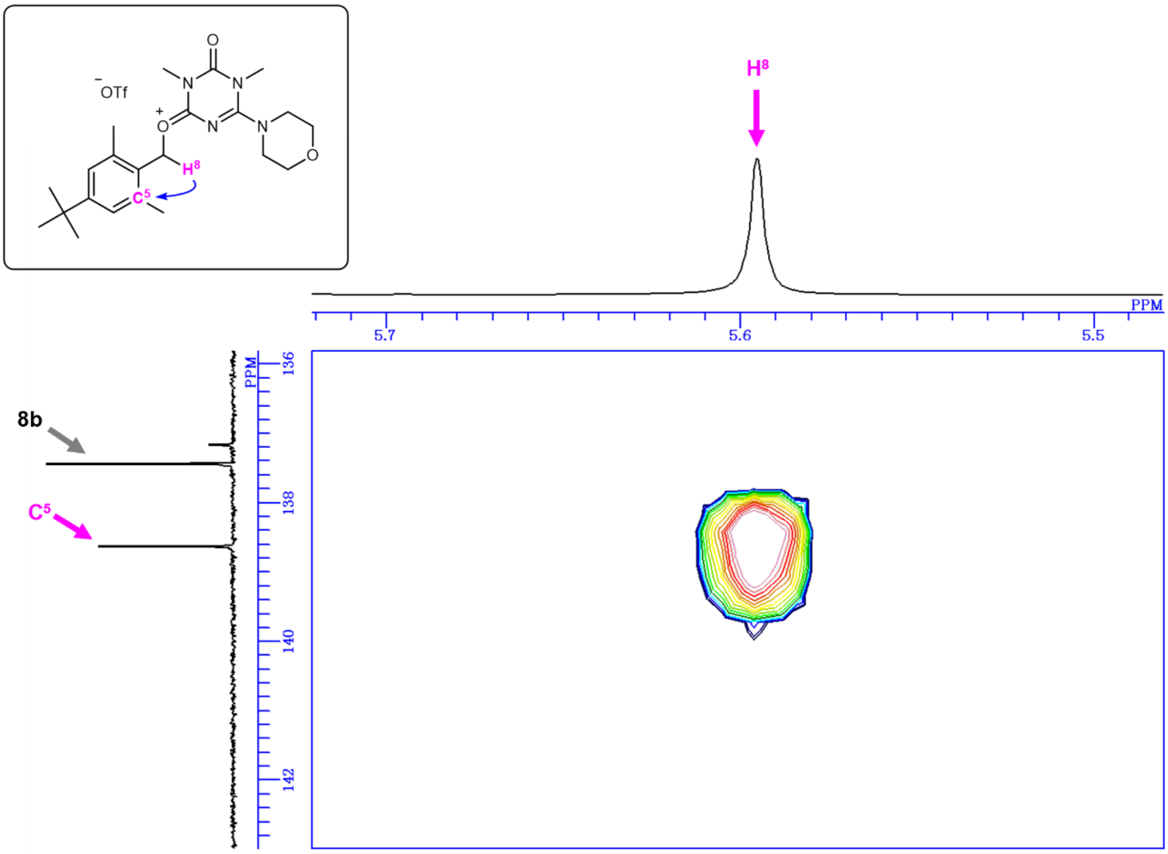

OTf
H8
C5
8b
PPM
5.7
5.6
5.5
136
138
140
142

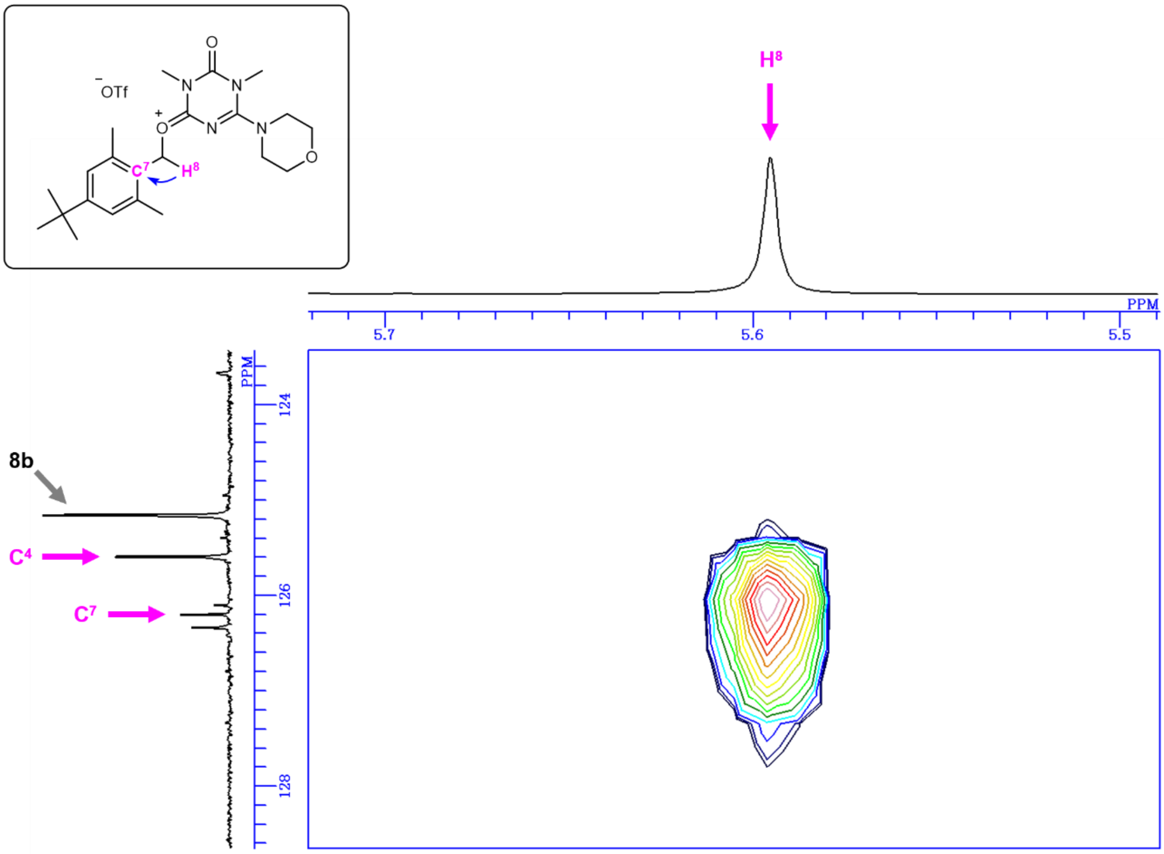

OTf
H8
C7
8b
C4
PPM
5.7
5.6
5.5
124
126
128

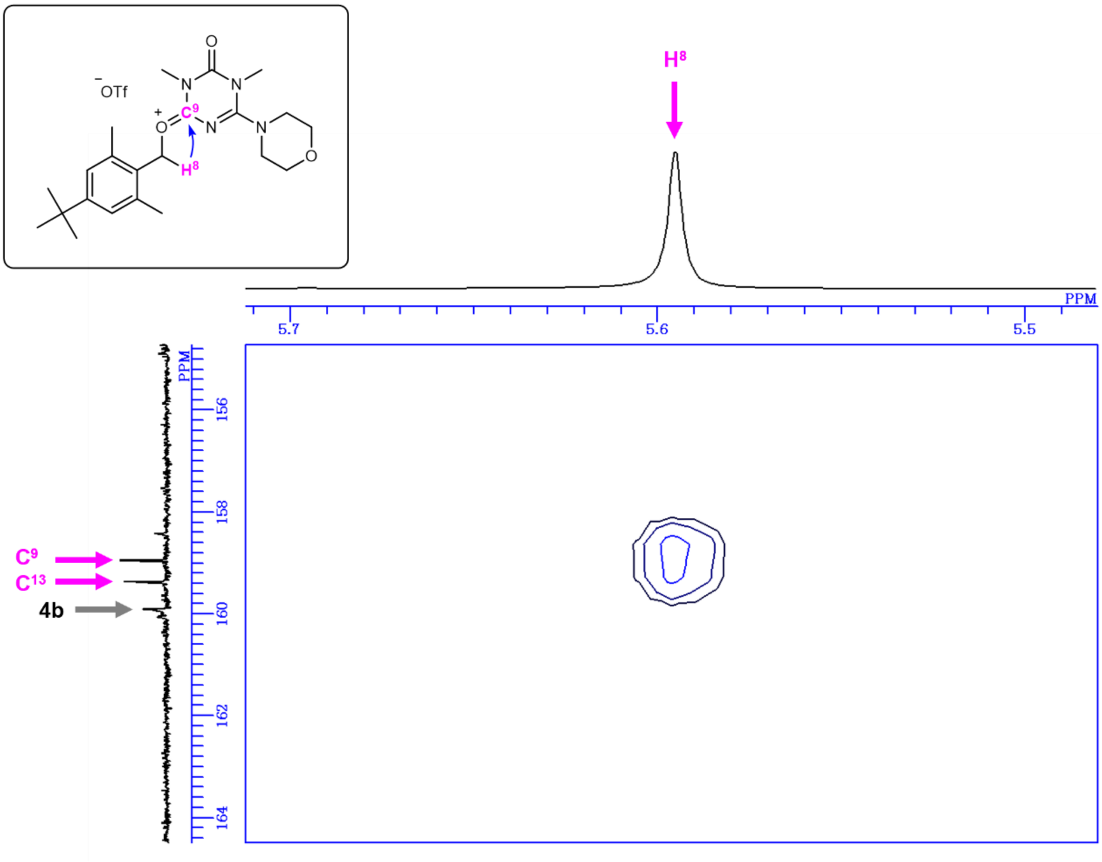

OTf
H8
C9
C13
4b
PPM
5.7
5.6
5.5
156
158
160
162
164

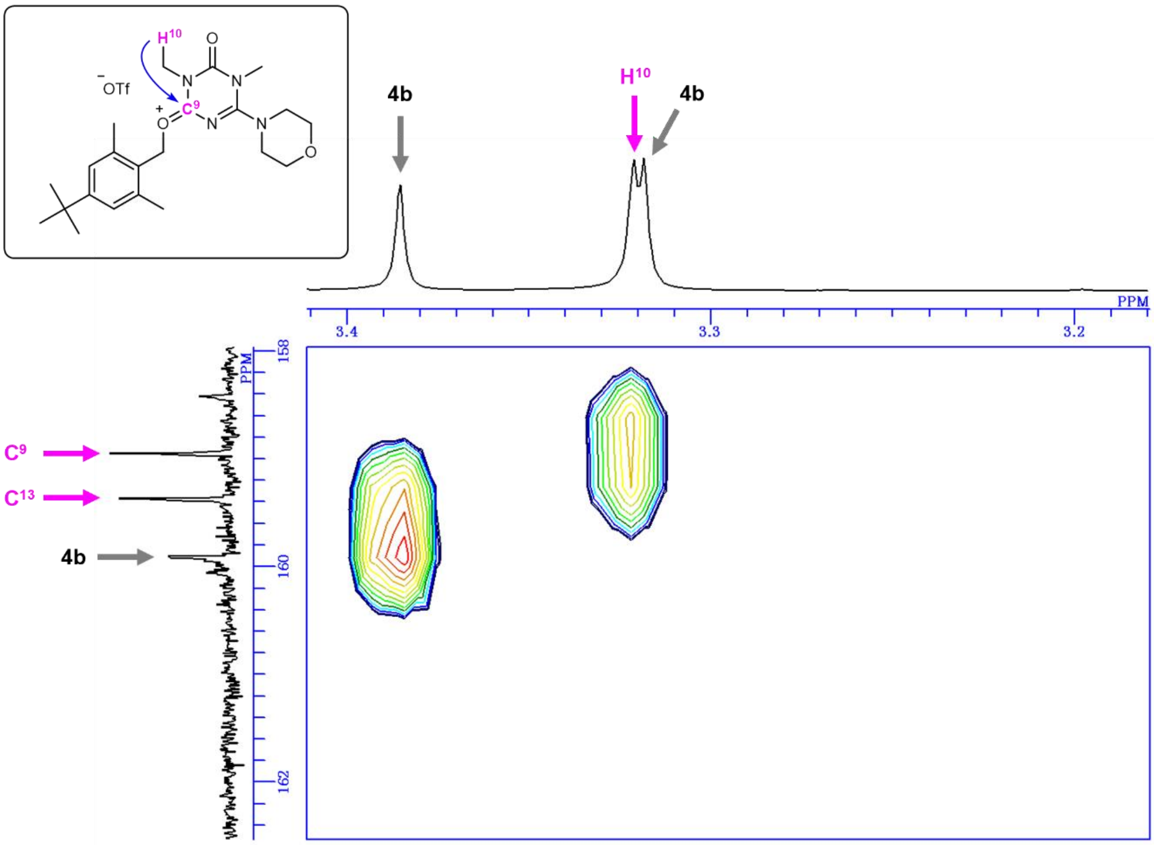

OTf
H10
C9
C13
4b
PPM
3.4
3.3
3.2
158
160
162

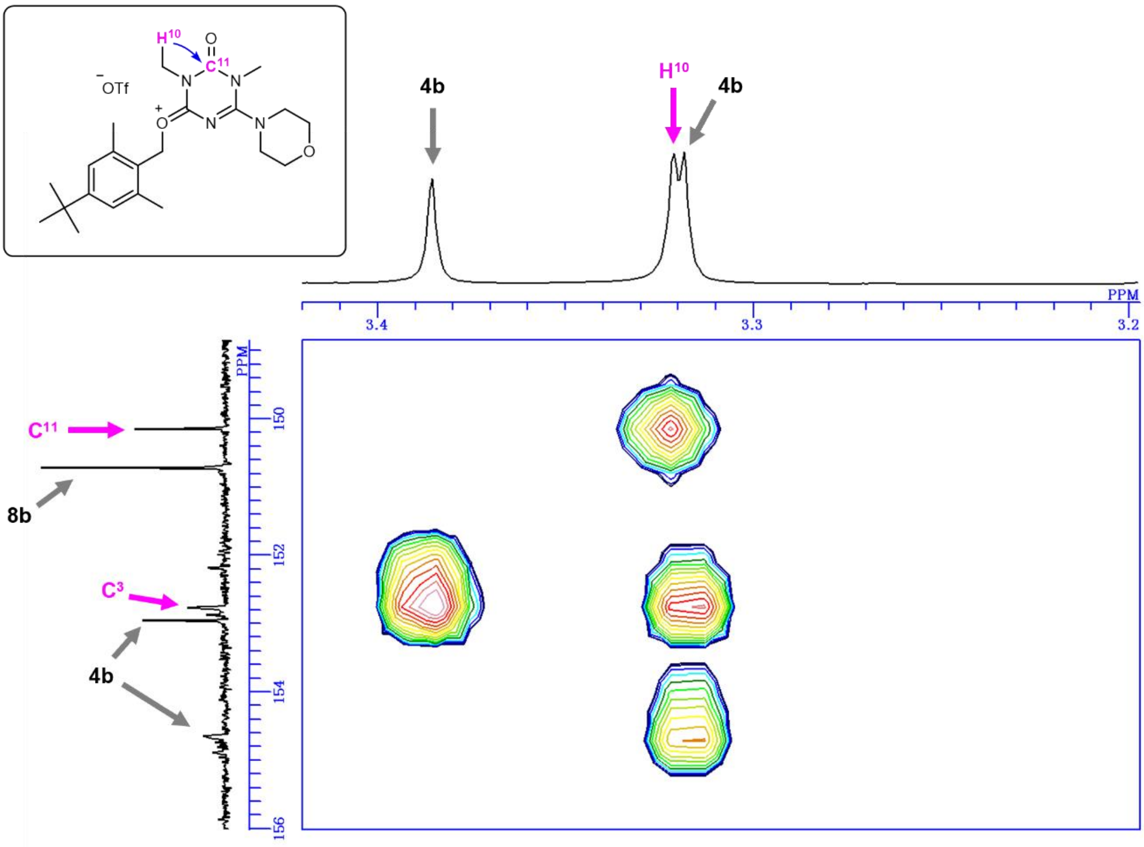
H10
C11
OTf
4b
H10
4b
PPM
3.4
3.3
3.2
C11
8b
C3
4b
150
152
154
156

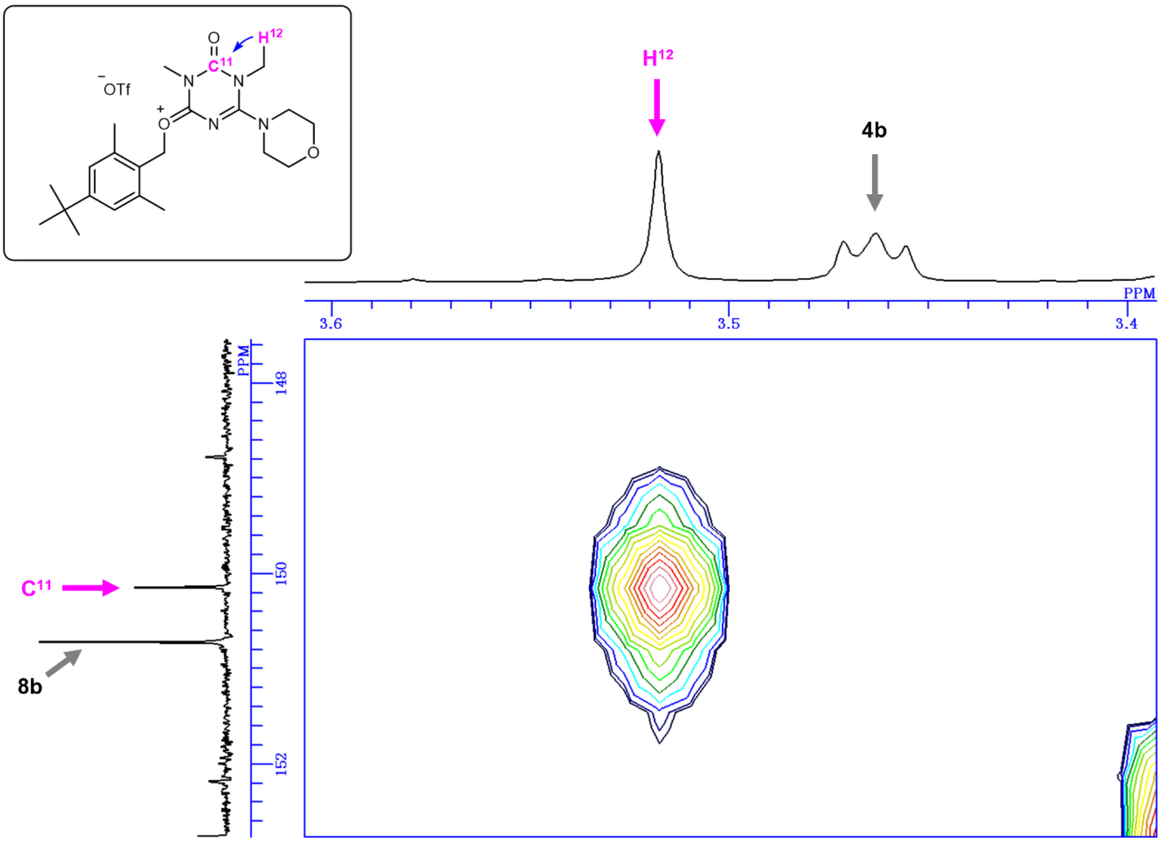
H12
C11
OTf
H12
4b
PPM
3.6
3.5
3.4
C11
8b
148
150
152

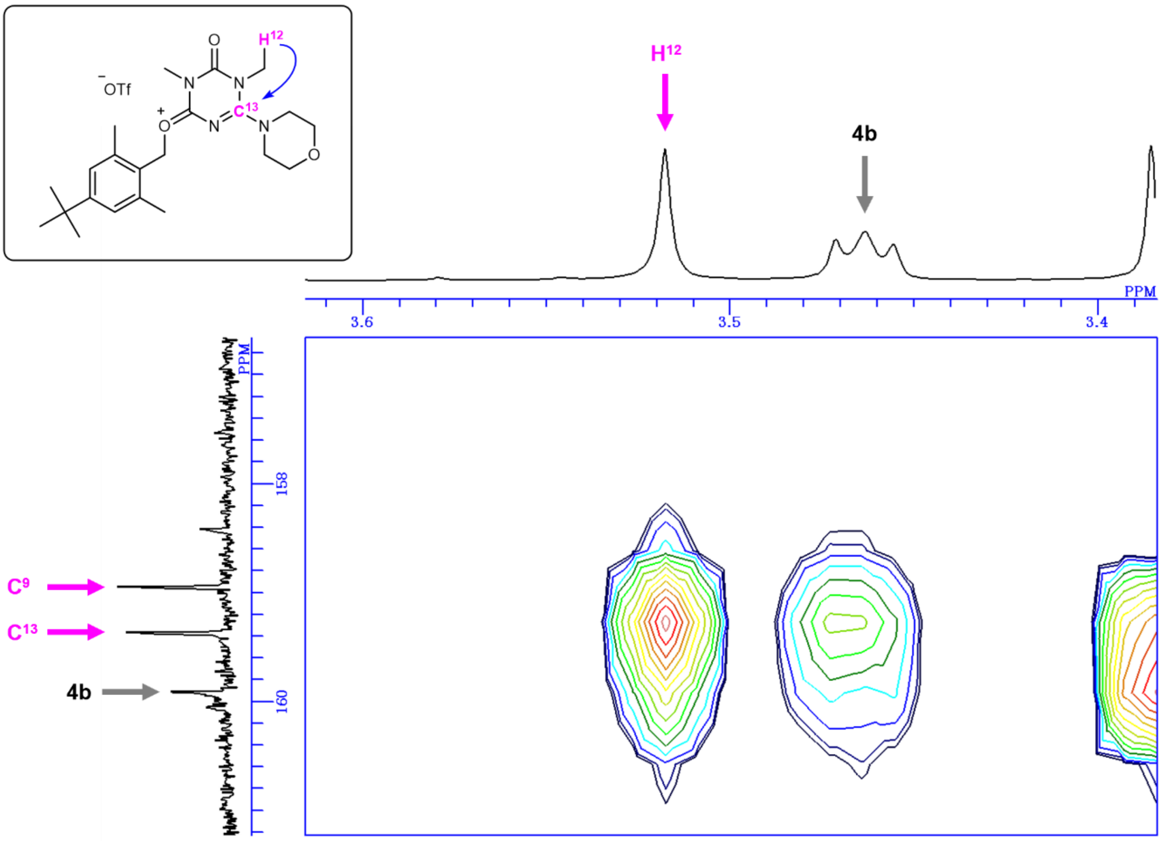

OTf
H12
C13
H12
4b
PPM
3.6
3.5
3.4
158
160
C9
C13
4b

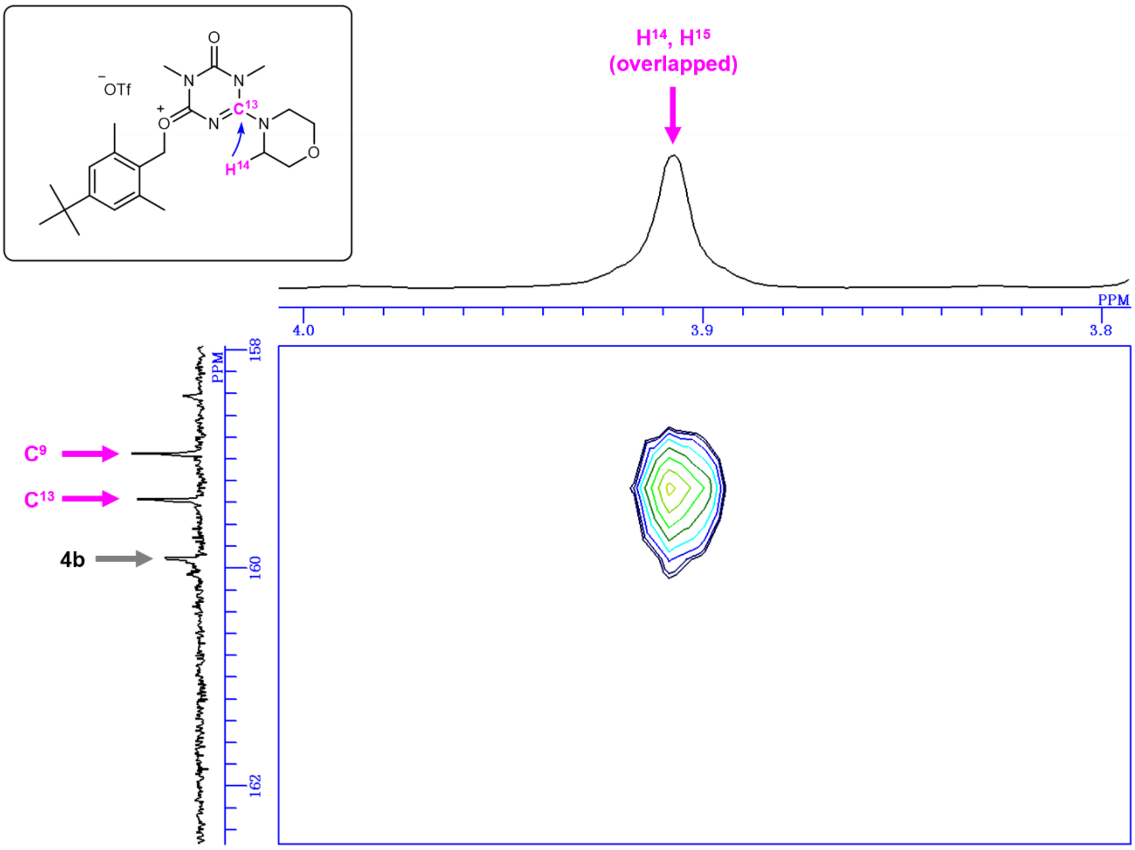

OTf
C13
H14
H14, H15
(overlapped)
PPM
4.0
3.9
3.8
158
160
162
C9
C13
4b

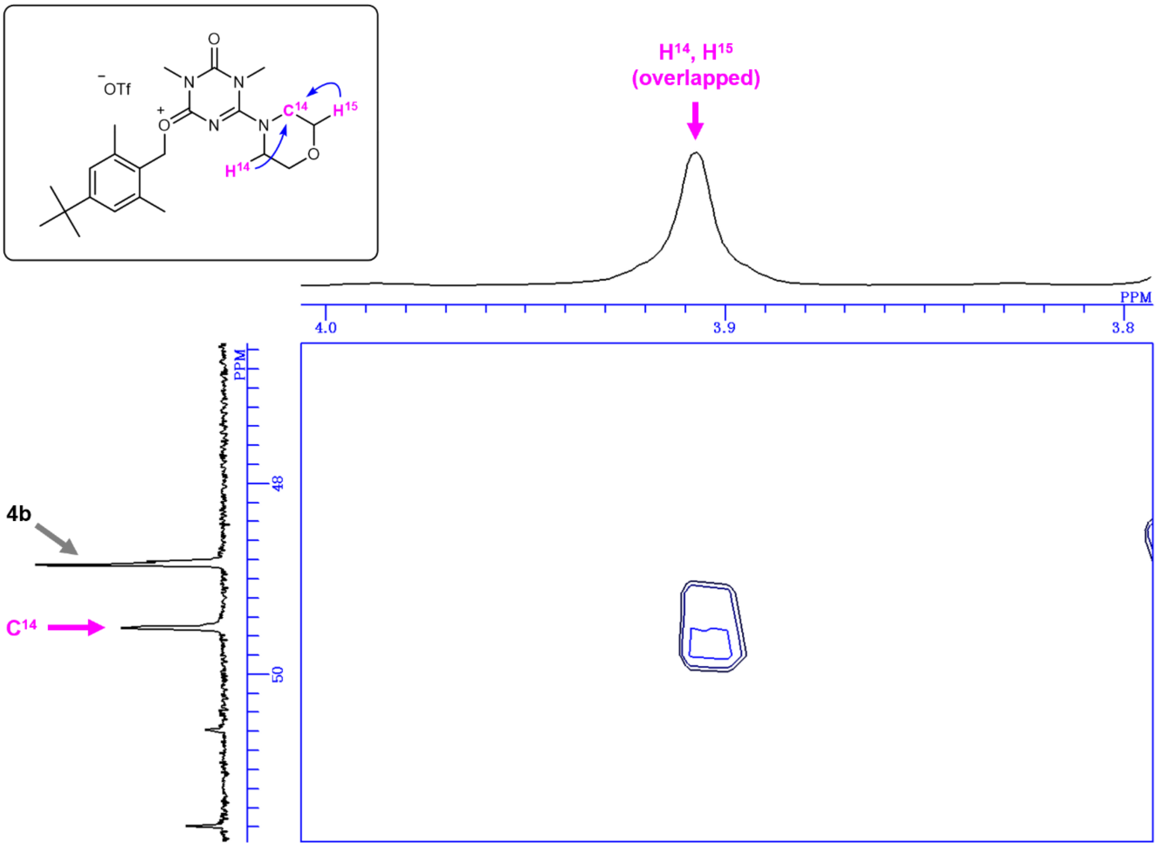
OTf
H14, H15
(overlapped)
4b
C14
PPM
4.0
3.9
3.8
48
50

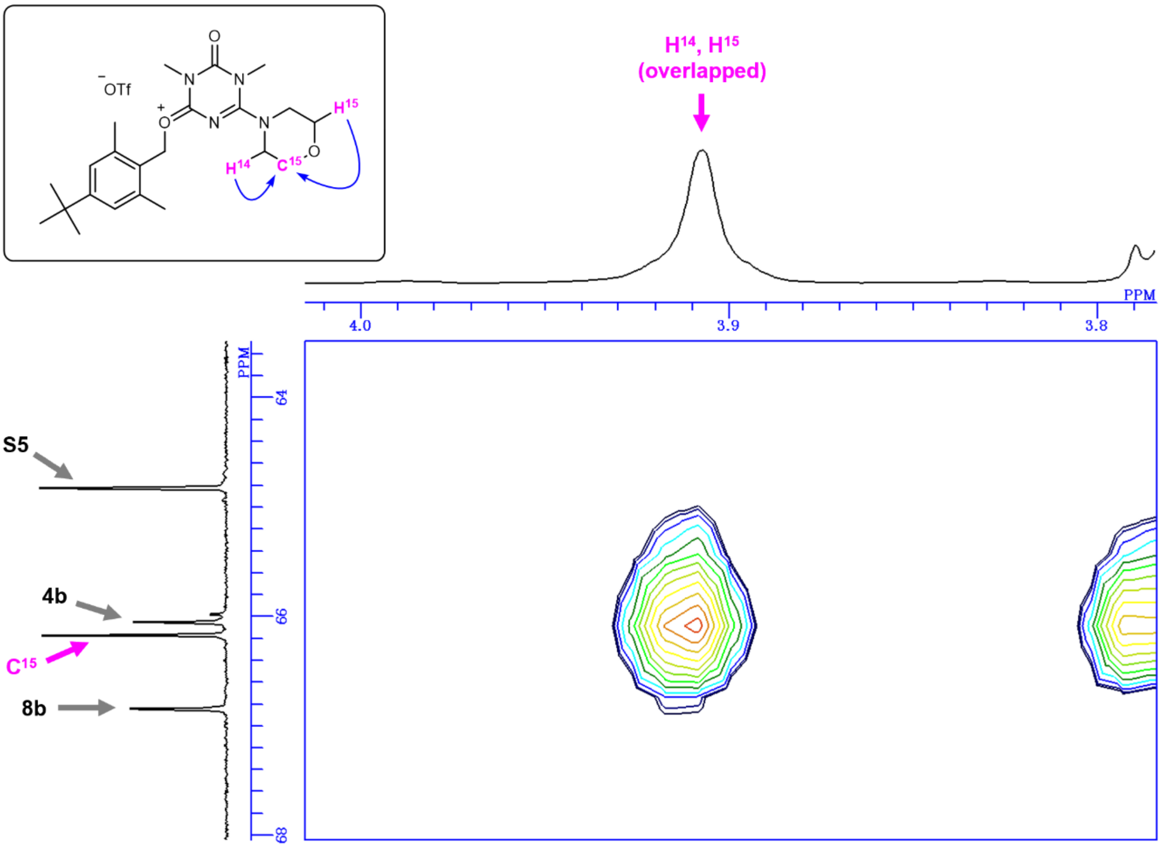
OTf
H14, H15
(overlapped)
S5
4b
C15
8b
PPM
4.0
3.9
3.8
64
66
68
